# Species Distribution Models and Abundance Estimates Enhance Breeding Bird Atlas Data

**DOI:** 10.1002/ece3.73808

**Published:** 2026-06-08

**Authors:** Nicholas G. Walton, Edmund J. Zlonis, Péter Sólymos, Alexis R. Grinde, Gerald J. Niemi

**Affiliations:** ^1^ Natural Resources Research Institute University of Minnesota Duluth Duluth Minnesota USA; ^2^ Wetland Wildlife Population and Research Group, Division of Fish and Wildlife, Minnesota Department of Natural Resources Bemidji Minnesota USA; ^3^ Department of Biological Sciences University of Alberta Edmonton Canada; ^4^ Departments of Biology and Integrated Biosciences University of Minnesota Duluth Duluth Minnesota USA

**Keywords:** breeding bird atlas, generalized linear models, Maxent, point counts, population estimates, species distribution models

## Abstract

Breeding bird atlases play a crucial role in understanding bird species distribution and abundance during the breeding season. This information is essential for creating accurate species distribution maps, which are fundamental for understanding the geographic range of species and identifying areas of high conservation value. Our primary goal was to develop species distribution models (SDMs) for as many Minnesota breeding bird species as possible using data from the Minnesota Breeding Bird Atlas (MNBBA; 2009–2013). The MNBBA combined volunteer atlas observations with systematic point‐count surveys, resulting in datasets that varied in structure and quality across species. Recognizing this variability, we applied multiple modeling approaches tailored to the available data, which also led to differing ecological interpretations. To maximize species coverage given heterogeneous data characteristics, we used three modeling strategies to maximize the number of species we modeled: (1) bootstrapped Poisson generalized linear models with a detectability offset to predict species' density and population size, (2) bootstrapped Poisson generalized linear models to predict a species' point count index of abundance, and (3) Maxent models to predict a species' index of environmental suitability. We applied the first strategy to 73 species, the second to 30, and the third to 33 species each (136 species in total). We also produced statewide population estimates for the 73 species using the first strategy. Our framework demonstrates that linking model choice to data structure significantly increases the number of species that can be modeled compared to a single‐model approach. While these results serve as a foundation for broad‐scale distribution and abundance hypotheses, we suggest this adaptable methodology be tested in other regions to maximize the utility of diverse atlas datasets.

## Graphical abstract

Our primary goal was to leverage the large amount of systematic and volunteer‐based data produced by the Minnesota Breeding Bird Atlas to develop species distribution models and population estimates for as many breeding bird species as possible. We utilized three complementary techniques to develop species distribution models and maps for 136 breeding bird species in Minnesota. We also produced statewide population estimates for 73 of these species.

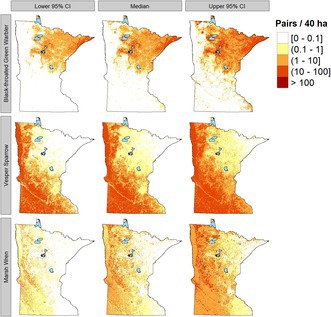

## 1 Introduction

Understanding the distribution and abundance of species is a fundamental goal of ecologists and essential for effective conservation management. Numerous studies and reviews have highlighted the alarming changes to species distributions as well as extinctions of populations across the planet (Wilson 2002; Cardinale et al. 2012). For instance, Mace et al. (2018) summarized that vertebrate population sizes have declined by 58% since 1970 and current species extinction rates are 100–1000 times higher than background rates. Rosenberg et al. (2019) estimated that bird populations in North America had declined by three billion birds since 1970 (29%).

Species ranges are shifting, contracting, expanding, and fragmenting in response to many natural and human‐related environmental perturbations (Chen et al. 2011). Species distribution models (SDMs) are an important tool for quantifying the potential causes for these changes to distribution (Franklin 2009) and abundance (Sólymos et al. 2020). SDMs combine observations of species occurrence or abundance with environmental variables to derive spatially explicit predictions of environmental suitability (Elith and Leathwick 2009; Guisan et al. 2013; Grinde and Niemi 2016). The use of SDMs can be particularly valuable when predicting the potential impacts of environmental change and are often used to inform conservation decisions across a range of spatial scales (Elith and Leathwick 2009; Howard et al. 2014; Niemi et al. 2016; Grinde et al. 2017; Zlonis et al. 2017).

Many approaches have been used to survey species across large areas; one common method includes breeding bird atlases (BBAs), which have been widely used in the United States, Canada, and many other jurisdictions across the globe (Donald and Fuller 1998; Mitchell‐Jones et al. 1999; Underhill and Gibbons 2002). Historically, most U.S. states and Canadian provinces have completed BBAs, as have many entire countries. The substantial amount of data gathered by these atlases provides a critical opportunity to improve our understanding of environmental characteristics associated with breeding birds, as well as population estimates and detailed spatial distributions. These data and analyses not only enhance our ecological knowledge, but are also essential for improving management and conservation decisions.

Furthermore, BBAs provide a unique opportunity to use field data gathered by a wide range of individuals possessing a broad spectrum of expertise, from amateur naturalists to professional ornithologists. BBAs have historically relied on citizen science volunteers for data gathering; however, the utility of these data is even more powerful when combined with systematic point count methods (Ralph et al. 1995; Matsuoka et al. 2014). When coupled with environmental covariates to construct SDMs (Donald and Fuller 1998; Mitchell‐Jones et al. 1999; Underhill and Gibbons 2002), these data provide high‐resolution insights into species‐habitat relationships. Recently, many BBA efforts [e.g., British Columbia (Davidson et al. 2015), Minnesota (Pfannmuller et al. 2017, 2024), and Pennsylvania (Wilson et al. 2012)] have included point count methodologies that improve the statistical use of these data because random sampling and survey attributes such as time of sampling, distance estimation, and estimates of species' detectability can be included (Sólymos, Matsuoka, et al. 2018).

Because BBAs capture a wide range of species, from the ubiquitous to the rare, the resulting data often exhibit varying levels of density and detection probability. Consequently, a single modeling approach may not be appropriate for an entire community. Our overall goal was to apply tailored modeling approaches that maximize the usefulness of BBA data to improve our understanding of covariates associated with the ecological distribution and abundance of bird species over a large region. Our primary goal was to produce SDMs for as many of Minnesota's breeding bird species as possible, while acknowledging that data characteristics of each species demand different modeling strategies and ecological interpretations. Our specific objectives were to: (1) Develop a framework that assigns species to one of three specific SDM strategies based on model assumptions, data quality; and distribution: (i) bootstrapped Poisson generalized linear models (GLMs) with a detectability offset to predict species’ density and population size, (ii) bootstrapped Poisson GLMs to predict a species’ point count index of abundance, and (iii) Maxent models to predict a species’ index of environmental suitability, (2) Illustrate the utility of this framework by presenting results for representative species. To exemplify the breadth of results across all 136 species with usable SDMs, we selected three species for each of the three modeling strategies to highlight the varying levels of ecological inference provided by each approach, (3) Suggest improvements to BBA sampling methodology that would enhance the usefulness and applications of future BBA and SDM goals.

## 2 Methods

### 2.1 Study Area

**FIGURE 1:**
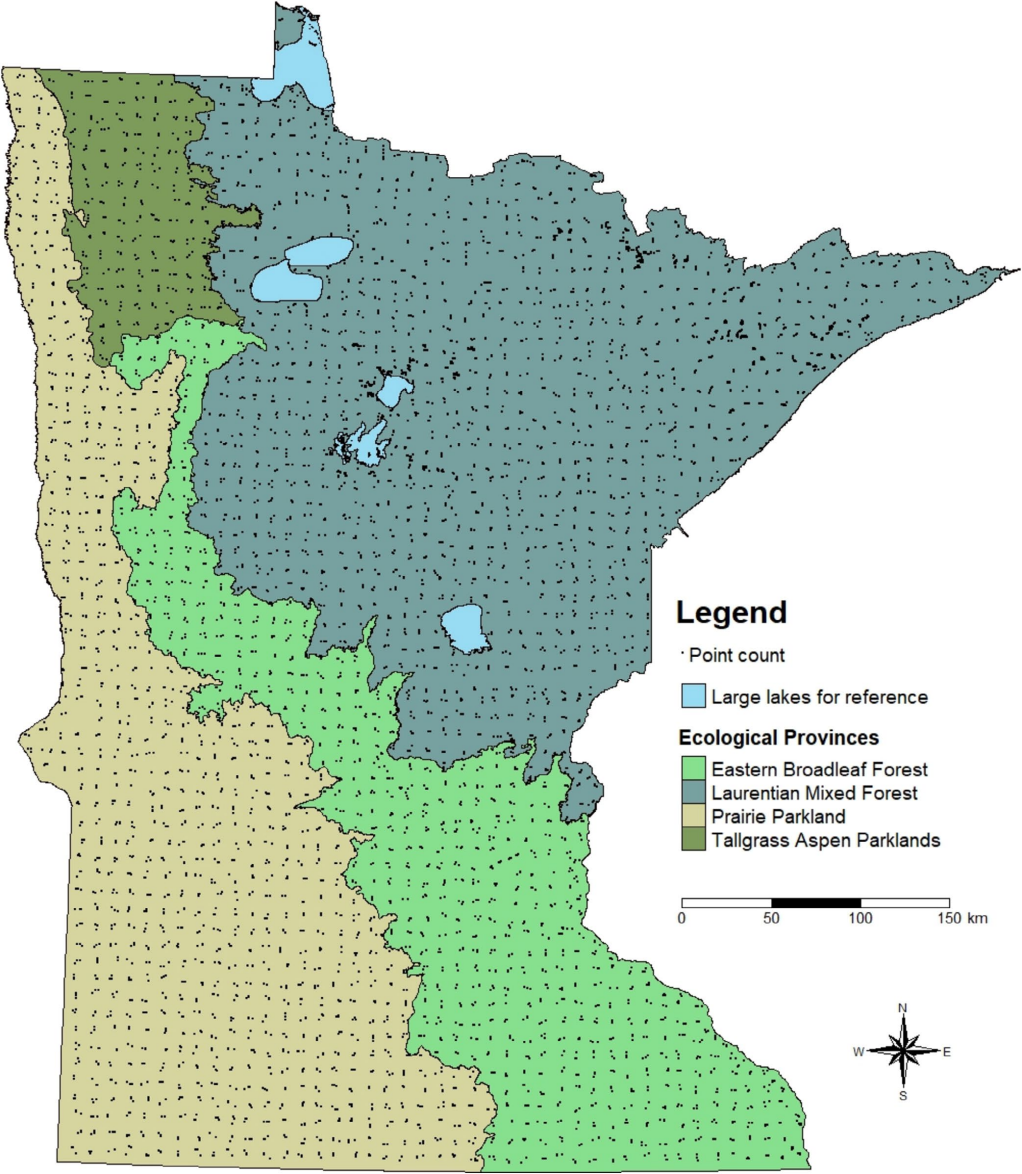
The state of Minnesota with the four major ecological provinces (Cleland et al. 1997, modified from Minnesota Department of Natural Resources 2014a). Also included are the 7882 point‐count locations surveyed during the MNBBA and supplementary point‐count data (Niemi et al. 2016).

To examine our goal and objectives, we used the BBA data for the state of Minnesota, USA, an area of 225,161 km^2^, elevation that ranges from 184 to 701 m, and a population of over 5.6 million people. Geographically, Minnesota lies in the center of the North American continent and on the ecotone among four major ecological provinces, the Laurentian Mixed Forest in the northeast, the Eastern Broadleaf Forest in the southeast, the Prairie Parkland in the west, and the Tallgrass Aspen Parklands in the northwest (Figure 1, Cleland et al. 1997). Minnesota has a humid continental climate with cold winters and mild to hot summers. Temperatures can range from −51°C in winter to 45°C in summer. Average annual precipitation ranges from 890 mm in the southeast to 510 mm in the northwest (Seeley 2015).

### 2.2 Breeding Bird Site Selection

The Minnesota Breeding Bird Atlas (MNBBA) data were gathered over a five‐year period (2009–2013) across the entire state of Minnesota and are summarized by Pfannmuller et al. (2017, 2024) and Le Tortorec et al. (2023). The atlas used the Public Land Survey System (PLSS) as a geographic frame of reference. The PLSS divides land into grid cells or townships that represent approximately 100 km^2^ (9.65 km x 9.65 km). A total of 2443 PLSS townships are found in Minnesota and because townships are a large area to sample, the northeastern quadrant (4.8 km x 4.8 km) was randomly selected among the four quadrants in each township as the focus for bird sampling. Many townships were < 100 km^2^ due to lakes, rivers, or state borders. If the northeastern quadrat was < 20 km^2^, then additional quadrats were assessed in clockwise order until a quadrat exceeded 20 km^2^. Ninety‐one townships were eliminated because no quadrats in the township met the > 20 km^2^ requirement. Hereafter, these remaining 2352 quadrants are referred to as “priority blocks”, representing the primary region of bird sampling; however, bird detections were allowed and included anywhere in the state during the Atlas sampling period (2009–2013).

We used two complementary methods for gathering breeding bird data for the MNBBA: (1) point count detections by trained and tested individuals, and (2) detections by citizen scientist volunteers. We used point counts conducted via a modified random approach to sample the breeding birds throughout Minnesota. We identified three a priori considerations affecting sampling design: (1) financial, (2) logistical, and (3) geographical. First, a calculation of available funding estimated that we could sample three point counts in each of the 2352 priority blocks over the five‐year MNBBA period, given they had an adequate road network. Hence, roadside counts were employed using secondary roads and did not include federal interstates or highways, Minnesota state highways, or any road deemed to have extensive traffic that would inhibit sampling due to safety, excessive activity, or noise. Roadside point counts were gathered throughout the majority of the state. Second, logistical constraints in roadless areas such as the Boundary Waters Canoe Area Wilderness, the Red Lake Peatland, and many other priority blocks that lacked roads constrained sampling to trails, portages, or other navigable routes using a combination of foot travel, bike, canoe, boat, or, in the case of extensive peatlands, amphibious vehicle. Approximately 5% of townships (120) lacked adequate roads and were sampled in this manner.

**FIGURE 2:**
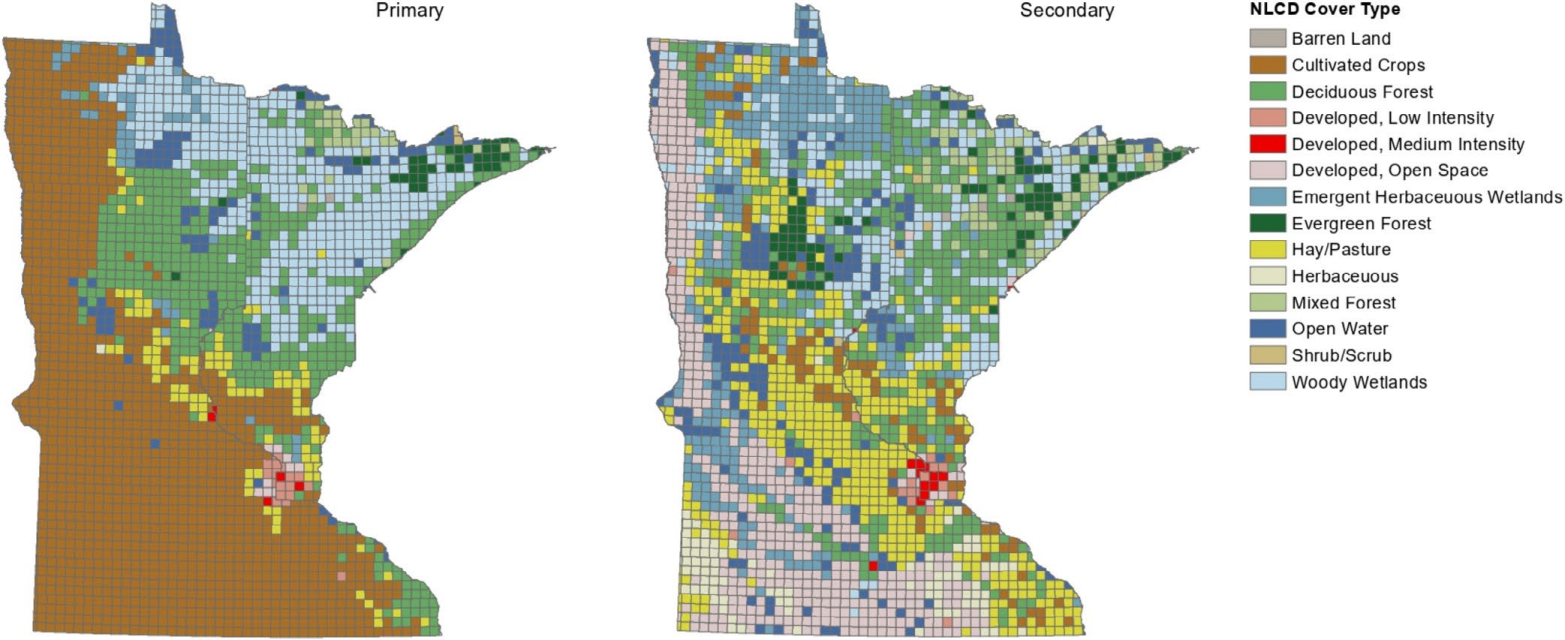
Dominant and subdominant National Land Cover Database (NLCD; version 1999–2003; Homer et al. 2004, 2007) cover types by township (9.65 × 9.65 km) in Minnesota used for the selection of point count samples.

Third, to sample each road‐accessible priority block in a township, three random point‐count locations were generated. The first point count was a randomly generated location on any secondary road within the priority block. The second and third points to be sampled within the priority block were identified within the most abundant and second most abundant cover type in the township, hereafter “dominant” and “subdominant” cover types. This consideration was included to maximize coverage of the most important habitats in the state and reduce the confounding effects of edges for subsequent statistical analysis. We used the National Land Cover Database (NLCD) (1999–2003) to identify the dominant and subdominant cover types in each priority block (Table 1, Figure 2; Homer et al. 2004, 2007). An expanding spiral search pattern from the first point was used to locate the nearest (straight‐line distance) points to each of the dominant and subdominant cover types. Land cover was filtered so that a point was only considered within the target habitat if > 40% of the land cover pixels in a 100 m radius were of the target cover type. The selected points in a priority block were required to be a minimum of 250 m apart to avoid overlapping bird detections among points (Pfannmuller et al. 2017, 2024). All points were identified a priori and recorded with a GPS in the field. Overall, depending on the density of the road network, between 2 and 5 townships (6–15 point counts) could be sampled by one counter in a given morning.

**TABLE 1:** Thirteen cover types and their percent area in the state of Minnesota (22,516,100 ha) identified using the National Land Cover Database (1999–2003; Homer et al. 2004, 2007).

Cover type	Area (%)	Description
Barren Land (Rock/Sand/Clay)	0.2	Bedrock, glacial debris, sand dunes, strip mines, gravel pits, and other earthen material. Vegetation < 15% of total cover
Cultivated Crops	38.8	Annual crops (e.g., corn, soybeans) or perennial woody crops (e.g., orchards, vineyards). Crops > 20% of total vegetation
Deciduous Forest	11.3	Dominated by trees > 5 m tall and > 20% of total vegetation cover. > 75% of the tree species deciduous
Developed, Low Intensity	1.2	Mixture of constructed materials and vegetation. Impervious surfaces are 20%–49% of cover
Developed, Medium or High Intensity	0.7	Mixture of constructed materials and vegetation to highly developed. Impervious surfaces account for 50%–100% of cover
Developed, Open Space	3.0	Mixture of some constructed materials, but mostly vegetation in the form of lawn grasses. Impervious surfaces are < 20% of cover
Emergent Herbaceous Wetlands	8.6	Perennial herbaceous vegetation > 80% of vegetative cover and substrate is periodically saturated or covered by water
Evergreen Forest	2.4	Dominated by trees > 5 m tall and > 20% of total cover. > 75% of the tree species are evergreen
Grassland/Herbaceous	1.4	> 80% graminoid or herbaceous vegetation. No intensive management (e.g., tilling), but can be grazed
Mixed Forest	4.9	Dominated by trees > 5 m tall and > 20% of total cover. Neither deciduous nor evergreen species > 75% of total cover
Pasture/Hay	6.1	Pasture/hay vegetation (e.g., grasses, legumes) > 20% of total vegetation
Shrub/Scrub	0.9	Dominated by shrubs or trees less than 5 m tall, with shrub canopy > 20% of total vegetation
Woody Wetlands	14.8	Forest/shrub vegetation > 20% of vegetative cover and substrate periodically saturated or covered by water

Inaccessible townships were sampled by identifying the most convenient access point within a priority block. The first point was the closest access point where a counter could initiate their morning survey (e.g., by foot along a trail or portage). Dominant and subdominant cover types were defined and sampled in the same way as described above. In most cases, only one inaccessible priority block could be sampled by a counter each day.

Prior to beginning field sampling, point count locations in both accessible and inaccessible priority blocks were examined using aerial imagery to check for public accessibility and to confirm point counts were located in dominant and subdominant cover types (e.g., in case of outdated or inaccurate NLCD classification). In addition, all points were field‐verified for accessibility (e.g., safety, noise, or road construction) and confirmed as the appropriate cover type. If unsatisfactory, an a priori process was in place for the field counter to randomly move the point to an alternative location (see Pfannmuller et al. 2024).

Separately from point counts described above, citizen scientists volunteered to document evidence of breeding birds in priority blocks on a first‐come, first‐served basis. These volunteers spent at least 25 h in their assigned priority block documenting the presence and breeding behavior of all bird species detected during the breeding season (Pfannmuller et al. 2017, 2024). All data gathered during the MNBBA were vetted by a technical committee of state bird experts. After observations passed the vetting process, volunteer detections were used in the Maxent analyses (described below) if they included geographic coordinates.

### 2.3 Bird Sampling

Point count sampling followed national and regional standards (Ralph et al. 1995; Matsuoka et al. 2014); ten‐minute, unlimited distance point counts were conducted between the last week in May in southern Minnesota and the second week in July in northern Minnesota (Hanowski and Niemi 1995; Pfannmuller et al. 2017, 2024). Each counter was required to pass an auditory test of 86 bird songs and any counter with less than five field seasons of point‐count sampling experience in Minnesota participated in a three‐day training session prior to collecting field data each spring. Counters also were required to pass a hearing test to ensure they were within the normal ranges as deemed by audiologists (Niemi et al. 2016). Point count data were gathered from approximately 0.5 h before to 4.0 h after sunrise on days with little or no wind (< 15 km/h) or precipitation. We extended the count period to 6.0 h after sunrise in western Minnesota because of the high proportion of excessively windy days in this region limited the number of appropriate sampling days. All birds heard or seen from a point were recorded (singing, calling, or visual), and their distance from observer estimated within 0–25 m, 25–50 m, 50–100 m, and > 100 m distance bins (Grinde et al. 2017). The minute of first detection was recorded for each individual detected during a point count, with the first and second minutes combined (i.e., 0–2 min, 2–3 min, 3–4 min, etc.). Detections of fly‐over individuals were recorded, but excluded from all analyses. A stratified‐random process was used to determine which townships were sampled in each of the 5 years of the MNBBA. The randomization was used to ensure sampling occurred in all areas of the state each year and that sampling occurred in regional blocks to minimize travel distances (Pfannmuller et al. 2017, 2024). Most of the 6921 point‐count locations were sampled once, but a random selection of 409 (5.9%) locations were sampled two or three times across years; no sites were sampled more than once in a given year.

### 2.4 Supplementary Point Count Data

In addition to the MNBBA, we included supplementary point count data from the Minnesota National Forest Breeding Bird Program in the models (Niemi et al. 2016), a long‐term monitoring program in the Chippewa and Superior National Forests of Minnesota. These data included 961 point‐count locations surveyed contemporaneously with the MNBBA each year from 2009 to 2013 and used the same 10‐min point count protocol (Etterson et al. 2009; Niemi et al. 2016).

All point counts in this dataset were conducted off‐road in remote, roadless areas, and many within forest cover types that were under‐sampled in other parts of Minnesota, thus complementing the roadside MNBBA point‐count data. These data represented 12.2% (961 of 7882) of point‐count locations and 38.6% (4616 of 11,951) of the point count data used in this analysis and added high quality roadless data to these analyses.

### 2.5 SDM Models

#### 2.5.1 Model Variables

We utilized a suite of 57 biophysical covariates to construct the species distribution models (Table 2). Unless otherwise noted, all variables had an initial spatial resolution of 30 X 30 m and were summarized at 200, 500, and 1000 m radii around each point‐count location to account for varying species‐specific habitat requirements. These covariates were grouped into five primary functional categories: land cover (1), disturbance (2), cover‐type structure (3), landscape metrics (4), and climate (5).

**TABLE 2:** List of potential variables used in species distribution modeling for the Minnesota Breeding Bird Atlas.

Group	Source	Biophysical attribute	Buffer radius (m)	Area (%)	GLM	Maxent
Land use/land cover	LANDFIRE Existing Vegetation Type^a^	Boreal coniferous	200, 500, or 1 k	4.6	×	×
		Boreal deciduous	200, 500, or 1 k	7.9	×	×
		Boreal lowland grass	200, 500, or 1 k	2.0	×	×
		Boreal shrub swamp	200, 500, or 1 k	3.2	×	×
		Cropland	200, 500, or 1 k	35.5	×	×
		Developed‐high intensity	200, 500, or 1 k	2.8	×	×
		Developed‐low intensity	200, 500, or 1 k	1.9	×	×
		Developed‐medium intensity	200, 500, or 1 k	0.3		×
		Lowland coniferous forest	200, 500, or 1 k	7.6	×	×
		Lowland deciduous forest	200, 500, or 1 k	0.9	×	×
		Lowland herbaceous	200, 500, or 1 k	1.1	×	×
		Northern hardwood forest	200, 500, or 1 k	8.0	×	×
		Oak forest	200, 500, or 1 k	1.9	×	×
		Oak savannah	200, 500, or 1 k	0.1	×	×
		Open water	200, 500, or 1 k	6.2	×	×
		Parkland deciduous forest	200, 500, or 1 k	0.1	×	×
		Pine forest	200, 500, or 1 k	0.8	×	×
		Pine‐oak barrens	200, 500, or 1 k	0.9	×	×
		Quarries‐strip mines‐gravel pits	200, 500, or 1 k	0.2		×
		Rural developed forest	200, 500, or 1 k	0.6		×
		Shrub swamp	200, 500, or 1 k	1.1	×	×
		Upland grassland	200, 500, or 1 k	10.4	×	×
		Upland native grassland	200, 500, or 1 k	0.8		×
		Upland shrub	200, 500, or 1 k	0.6	×	×
		Urban developed forest	200, 500, or 1 k	0.3	×	×
	National Wetlands Inventory^b^	Bog	500	6.4		×
		Marsh	500	3.5		×
		Riverine	500	0.8		×
		Shrub wetland	500	4.2		×
		Woody wetland	500	2.6		×
		Wet Meadow	500	1.8		×
Disturbance	Insect disturbance^c^	Eastern larch beetle	200, 500, or 1 k	0.3	×	
		Forest tent caterpillar	200, 500, or 1 k	2.6	×	
		Spruce budworm	200, 500, or 1 k	0.6	×	
	Forest loss^d^	Forest loss	200, 500, or 1 k	2.0	×	×
	Road density^e^	Road density	500	—	×	
	Census^e^	Census	500	—	×	×
Cover structure	LANDFIRE Existing Vegetation Cover^a^	Herb Cover > = 10 and < 40%	200, 500, or 1 k	2.0	×	
		Herb Cover > = 40 and < 70%	200, 500, or 1 k	7.9	×	
		Herb Cover > = 70 and < = 100%	200, 500, or 1 k	10.0	×	
		Shrub Cover > = 10 and < 40%	200, 500, or 1 k	0.2	×	
		Shrub Cover > = 70 and < = 100%	200, 500, or 1 k	1.1	×	
		Tree Cover > = 10 and < 40%	200, 500, or 1 k	6.3	×	
		Tree Cover > = 40 and < 70%	200, 500, or 1 k	20.7	×	
		Tree Cover > = 70 and < = 100%	200, 500, or 1 k	6.7	×	
	LANDFIRE Existing Vegetation Height^a^	Forest Height 0 to 10 m	200, 500, or 1 k	2.5	×	
		Herb Height 0 to 0.5 m	200, 500, or 1 k	11.2	×	
		Herb Height > 0.5 m	200, 500, or 1 k	8.7	×	
		Shrub Height 0 to 1 m	200, 500, or 1 k	0.4	×	
Landscape metrics	LANDFIRE Exisitng Vegetation Type^a^	Number of patches	500	—	×	
		Patch richness	500	—	×	×
Climate	Climate^f^	Average annual precipitation	0 (point)	—	×	×
		Average annual temperature		—		×
		Average June precipitation	0 (point)	—	×	
		Minimum June temperature C	0 (point)	—	×	
	Ecological Province^g^	Ecological province	0 (point)	—	×	
		X coordinate (UTM15)	0 (point)	—	×	

*Note:* “Groups” were used during model selections for GLMs (with and without a QPAD offset), but not Maxent models. GLMs used a single buffer radius chosen based on BIC of the first bootstrap iteration (using the full training dataset). Maxent always used the 500 m buffer radius. Area (%) is the percent of the attribute found across the state of Minnesota (22,516,100 ha).

^a^ Zhu et al. (2006), http://landfire.cr.usgs.gov/viewer/.

^b^ USFWS (2016).

^c^ USDA (2014).

^d^ Hansen et al. (2013).

^e^ US Census Bureau (2012).

^f^ https://prism.oregonstate.edu.

^g^ https://gisdata.mn.gov/.

The first category, land cover (1), comprised 31 cover type classifications derived from LANDFIRE existing vegetation type version LF 2010/LF 1.2.0 (Table 2, Appendix S1; Rollins et al. 2006, Zhu et al. 2006, LANDFIRE 2013) and the National Wetland Inventory (Table 2; USFWS 2016). Second, disturbance (2) metrics included six variables: defoliation by eastern larch beetle ( *Dendroctonus simplex* ), tent caterpillar (*Malacosoma* spp.), and spruce budworm ( *Choristoneura fumiferana* ; USDA 2014); forest loss (Hansen et al. 2013), and anthrogopoengic stressors including road (U.S. Census Bureau 2012) and human population density (U.S. Census Bureau 2012) calculated at the 500 m radius (Table 2). Third, cover‐type structure (3) was represented by 12 variables derived from LANDFIRE existing vegetation height and cover, including eight classes of vegetation cover (tree, shrub, and herbaceous cover ranging from 10% to 100%) and four height categories (forest height < 10 m, shrub height < 1 m, herbaceous height < 0.5 m, and herbaceous height > 0.5 m) (Table 2; Rollins et al. 2006; Zhu et al. 2006; LANDFIRE 2013). Fourth, we calculated landscape metrics (4) using Fragstats (McGarigal et al. 2012): cover type patch abundance and richness. Both metrics were processed using a 500 m moving window and summarized within a 500 m radius around the point count locations (Table 2; McGarigal et al. 2012). Finally, the climate and location category (5) included three PRISM‐based attributes (average annual and June precipitation; minimum June temperature (Daly et al. 1997, PRISM Climate Group 2013)), the Universal Transverse Mercator X coordinate (UTM zone 15 N) of each point‐count location, and ecological province (Table 2; Cleland et al. 1997, Minnesota Department of Natural Resources 2014a).

#### 2.5.2 Species Distribution Modeling Approaches

**FIGURE 3:**
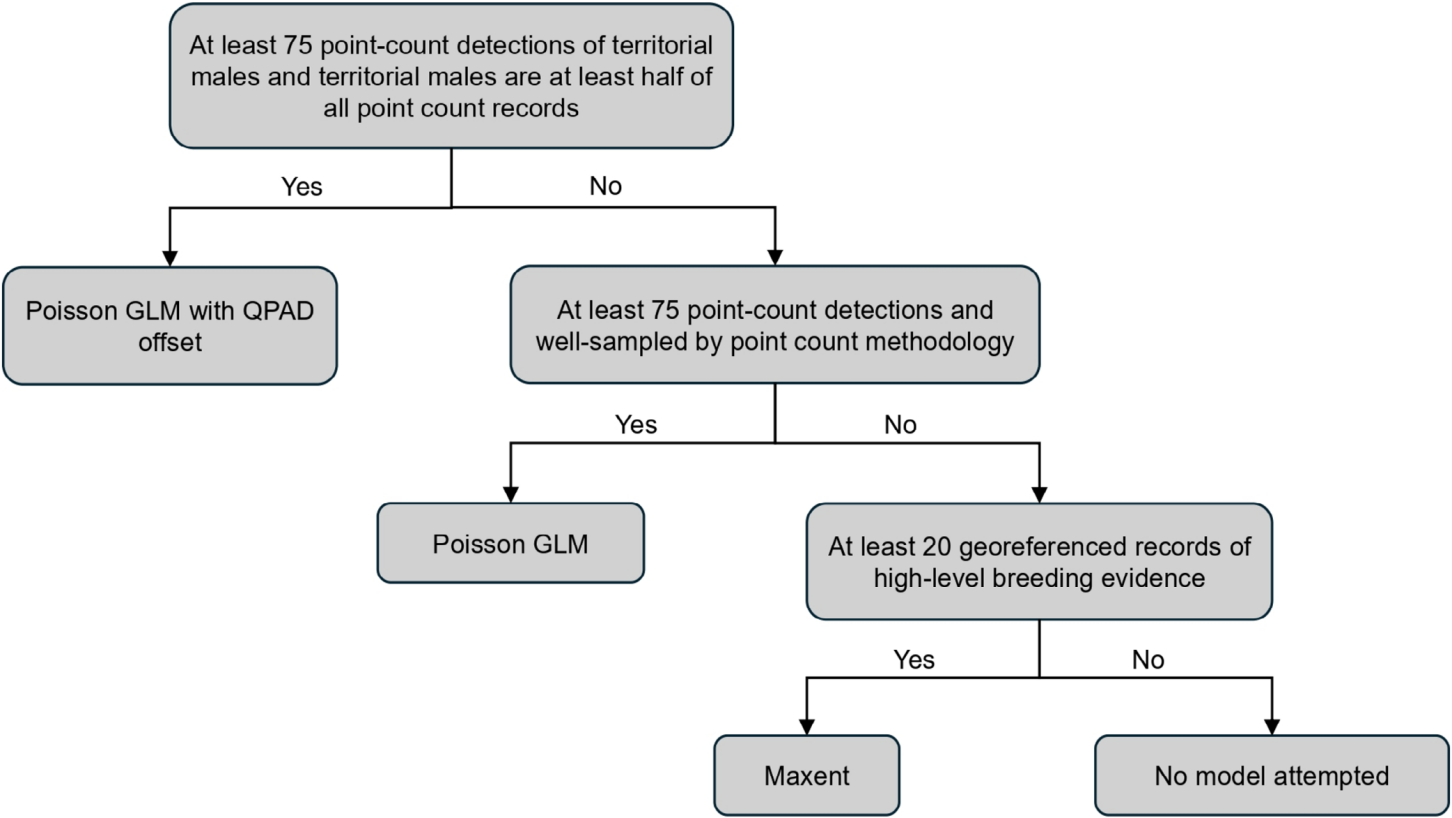
Flowchart identifying how specific modeling strategies were determined for each species modeled from the Minnesota Breeding Bird Atlas. Each species and the associated model type are identified in Appendix S2.

We identified three modeling approaches to generate habitat‐based SDMs tailored to the varying data types and species distributions within the MNBBA. These included (1) bootstrapped Poisson generalized linear models (GLMs) with a detectability offset to predict species' density and population size (e.g., Ball et al. 2016; Edwards et al. 2023), (2) bootstrapped Poisson GLMs to predict a point count index of abundance (e.g., Niemi et al. 2016), and (3) Maxent models to predict an index of environmental suitability (e.g., Zlonis et al. 2017). Rather than comparing these approaches for each species, we assigned a single method to each bird species based on its life history, total number of observations, and the overarching goal of maximizing the scope of ecological inference. Our objective was to model as many breeding bird species as possible while adhering to the data constraints. Thus, we developed a decision‐making flowchart that helped identify the appropriate modeling method for each species (Figure 3; Appendix S2).

The level of ecological inference varied widely between these methods. The GLM with a detectability offset provided the highest level of inference, yielding density estimates that are comparable across different studies and providing a mechanistic understanding of covariate effects on bird populations. This approach resulted in statewide maps of predicted density and total population estimates. The GLM without a detectability offset produced an index of abundance (expected count per point count). While this method similarly identified mechanistic covariate effects and generated statewide distribution maps, the results represent an index of relative abundance rather than absolute density, limiting its comparability to other studies using different protocols (D. H. Johnson 2008).

Finally, Maxent models provided the most restricted ecological inference. The resulting index of environmental suitability cannot be reliably compared between species or external datasets (Merow et al. 2013). However, Maxent is a robust tool for identifying environmental associations and generating statewide distribution maps for rare, uncommon, or under‐surveyed species with limited data, often collected by opportunistic volunteer observations. While numerous alternative SDM methodologies exist (Franklin 2009), we determined that these three approaches best addressed the varying extent, resolution, and quality of data available in the MNBBA.

#### 2.5.3 Generalized Linear Models With QPAD Detectability Offset

For this modeling effort, we used GLMs combined with the QPAD approach to account for variation in availability and effective detection radius (EDR; Sólymos et al. 2013; Sólymos, Moreno, and Lele 2018, Zlonis et al. 2019, Edwards et al. 2023). We included only species that had a minimum of 75 point‐count detections of territorial males (Sólymos et al. 2013; Bayne et al. 2016; Sólymos, Matsuoka, et al. 2018) with > 50% of detections being singing males (Figure 3). We used bootstrapped (Breiman 1996; Efron 2014) GLMs (McCullagh and Nelder 1989) with a Poisson error structure and a log link to derive ensemble models of species distribution and abundance. Ensemble models used 46 of the biophysical attributes (Table 2) as potential covariates to our response variable, which was number of singing males per point count. No variables with correlation > 0.8 were included. Model selection and predictions were conducted using program R version 3.6.1 (R Core Team 2019). A stratified random (region and year) holdout dataset of 10% was used to validate models.

QPAD combines a removal model of detection availability (Farnsworth et al. 2002) with a distance model of detection probability (Buckland et al. 2001) into a location‐specific offset that can be used in any model framework that supports the use of offsets (Sólymos et al. 2013; Edwards et al. 2023). Variables we considered for the removal model were time since local sunrise, ordinal day, and their quadratic terms. Variables considered for the distance model included proportion of tree cover and land cover composition (LCC). Proportion of tree cover was a continuous variable indicating the proportion of forest cover within a 500 m radius of each count location. We derived forest cover from the Minnesota Land Use and Cover dataset, downloaded from the Minnesota Geospatial Commons (Minnesota Department of Natural Resources 2014b). LCC consisted of five land‐cover categories: open habitats (e.g., developed, herbaceous), shrublands and developed forests, open forests (10%–40% cover), moderately dense forests (40%–70% cover), and dense forests (> 70% cover). Land‐cover categories were derived from LANDFIRE version LF 2010/LF 1.2.0, by combining the existing vegetation type and existing vegetation cover classifications (Rollins et al. 2006; Zhu et al. 2006; LANDFIRE 2013). For example, locations classified as forests based on the LANDFIRE type and classified as having > 70% vegetation cover based on the LANDFIRE cover, would be considered the ‘dense forest’ LCC. Point count locations were assigned the most common LCC category within a 500 m radius. The data used to estimate offsets included point counts from the MNBBA and Minnesota National Forest Breeding Bird Program; a single point count was selected at random from locations with multiple visits. QPAD offsets were derived using R packages detect (Sólymos, Moreno, and Lele 2018) and QPAD (Sólymos et al. 2013, Edwards et al. 2023).

During model selection we used *β* = 240 bootstrap iterations, where data for the first iteration consisted of the entire training dataset (i.e., the full dataset minus a 10% holdout set), and all other iterations used a spatio‐temporal stratified random selection with replacement from the training dataset following Ball et al. (2016). Resampling allowed us to estimate non‐parametric confidence intervals on our predictions (described in more detail below). A preliminary analysis was conducted to determine the scale of biophysical attributes to be used for each species (i.e., 200, 500, or 1000 m). We fit the full training dataset to the first category of biophysical attributes (land cover) at each of the three scales. The scale with the lowest Bayesian information criterion (BIC; Schwarz 1978) was selected as the scale for the remainder of the model selection process for a given species.

To build the GLMs we applied “branching” forward selection (*sensu* Ball et al. 2016, Westwood et al. 2019) to the five categories of biophysical attributes (Table 2). First, we started with a null model (intercept only), and conducted stepwise forward selection on the first group of variables (i.e., land cover; Table 2). Selection for this group stopped when no additional variables improved the model (based on BIC) or all variables from the current group were added to the model. This model was then used as a starting model for forward stepwise selection on the next category of biophysical attributes (i.e., disturbance). The stepwise selection process continued for each of the remaining three biophysical categories (Table 2). Variables were not removed once selected within each category of this branching process. The order of these categories was chosen a priori based on hierarchical habitat selection principles (Ball et al. 2016; D. H. Johnson 1980). We used bootstrap aggregation (Breiman 1996; Efron 2014) to estimate variable importance, and parameter and prediction confidence intervals. Variable importance was the frequency with which a variable was selected in the *β* bootstrapped models (Efron 2014). The combination of bootstrap and variable selection provides non‐parametric confidence intervals that incorporates model‐related (covariate selection) uncertainty, thus is more robust against model‐misspecification related biases compared to bootstrapping a model with a fixed set of covariates.

We tested for overdispersion (Cox 1983) using the first model (i.e., the first bootstrap iteration using the full training dataset) for each species. We considered a model to be overdispersed if the deviance from the first model was greater than the degrees of freedom from the null model. Overdispersion can be caused by many sources including excess zero observations (zero‐inflation), missing important covariates, and excessive heterogeneity (Payne et al. 2017). When overdispersion was detected, we refit the models using a negative binomial error structure. These models were fit using the glm.nb function from the MASS package (Venables and Ripley 2002).

The resulting models were projected statewide on a 90 × 90 m grid. Thus, each grid cell had 240 predicted values (one for each bootstrap iteration described above) and the estimate for a given cell was the median predicted value for that cell (Breiman 1996, Efron 2014). Similarly, the lower and upper ends of the 95% bootstrap confidence interval were the 2.5 and 97.5 percentiles for each cell. We assumed a 1:1 ratio between territorial males and females so our mapping unit was pairs (i.e., singing males × 2) per 40 ha.

Using the QPAD offsets described above, allowed us to derive statewide population estimates from these predictions. The population estimate for a given species was the median statewide prediction multiplied by two (i.e., assuming the 1:1 ratio of males to females). Additionally, the *β* bootstrap predictions allowed us to calculate a 95% bootstrap CI of the population estimate. The bootstrap process and predictions were computationally intensive; we used Minnesota Supercomputing Institute's (MSI; www.msi.umn.edu) high performance computing systems to run the analyses in parallel using R packages doParallel (Microsoft Corporation and Weston 2020) and Rmpi (Yu 2002). We compared our population estimates to those developed by Partners in Flight (PIF; 2020) for Minnesota.

Predictions were generated for the 10% holdout dataset using the 240 models derived from the training dataset. We used R package pROC (Robin et al. 2011) to calculate the area under the receiver operating curve (AUC; Fawcett 2006) from the observed (> 0 counts coded as 1; e.g., Stralberg et al. 2025) and expected values for the holdout dataset. The proportion of non‐zero counts that were one (versus greater than one) was 68% for species modeled without the QPAD offset and 71% for those with the QPAD offset. AUC can range from 0 to 1 and an AUC of > 0.50 indicates that a model is better than random at classifying an observation (Fawcett 2006). This was used as an indicator of the reliability of the models for a given species when applied to out‐of‐sample data and allowed us to gauge the usefulness of models across all three of the modeling strategies. We also calculated Root Mean Square Error (RMSE) for the models based on count data, GLM with and without QPAD offsets.

#### 2.5.4 Generalized Linear Models Without Detectability Offset

The second modeling approach was applied to species having at least 75 point‐count detections but not primarily detected as territorial males (Figure 3). In this case, the response variable was total detections per point count (excluding detections of fly‐over individuals) and not necessarily singing males. Model selection and bootstrap aggregation were the same as in the previous method, but without an offset. Map predictions were also the same, except the mapping unit was expected birds per 10‐min point count, which we treated as an index of abundance. Since we did not account for detection probability, we were not able to estimate density and thus derive population estimates for species using this approach.

#### 2.5.5 Maxent

The Maxent machine‐learning method (Phillips et al. 2006; Elith et al. 2011) was used as a tertiary option to model distributions of species that did not meet the criteria for GLMs, but had at least 20 georeferenced detections of high‐level breeding evidence (i.e., probable or confirmed nesting records; Pfannmuller et al. 2017, 2024; Figure 3). Maxent is known to handle small sample sizes well (Phillips and Dudík 2008) and accurate models can be developed with 20, or possibly even fewer, presence records (Anderson and Gonzalez Jr 2011; van Proosdij et al. 2016). Though Maxent provided a solution for modeling additional species, it's important to note that the scope of ecological inference is narrower when compared to our GLM methods. We did not utilize the logistic output from Maxent as a probability of occurrence (Yackulic et al. 2013), instead we interpreted raw Maxent output as an index of environmental suitability (Merow et al. 2013; Merow and Silander Jr. 2014).

The detections used were primarily from volunteer‐based sampling within priority blocks of a township, as opposed to point counts. Approximately 7% out of more than 25,000 of the potentially available detections included geographic coordinates that allowed for habitat modeling of many species that would have otherwise not have had sufficient data to model using GLMs. Eighteen of the species modeled with the GLM strategy also had suitable numbers of georeferenced breeding records from volunteer data: for these species (see Appendix S2), we attempted both Maxent and GLM models and reported results of the model with the highest AUC value.

Maxent models used 36 of the biophysical covariates (Table 2) and all species used the 500 m scale. Only variables with a correlation < 0.8 were used; we included a slightly different subset of potential biophysical covariates because Maxent model selection evaluates all variables at the same time (i.e., no branching selection) and is less sensitive to collinearity (Feng et al. 2019). There was no stepwise variable‐selection procedure; all variables were considered simultaneously. Model development and selection were implemented using the ENMeval package (Muscarella et al. 2014) in program R version 3.2.3 (R Core Team 2016). Maxent relies on several transformations of covariates and a regularization multiplier to do internal model selection (Phillips et al. 2006). However, varying the types of transformations and the regularization multiplier values has been shown to create more useful models that are more generalizable (i.e., less over fit; Warren and Seifert 2011, Muscarella et al. 2014). To simplify interpretation of results, we limited analyses to linear or linear and quadratic transformations of covariates (Merow et al. 2013). Regularization multiplier values ranged from 1 to 10 at increments of one. Thus, we compared 20 different combinations of regularization multiplier values and covariate transformations for each species. Each model was run using five‐fold cross‐validation (e.g., Zlonis et al. 2017).

To account for potential bias in volunteer reporting locations, we used a targeted background approach (Phillips and Dudík 2008) where the range of covariate values available to each species were characterized from the locations of all volunteer records, as opposed to random locations. In preliminary modeling, we assessed this by comparing Maxent models utilizing random background locations to those using targeted‐background locations. The former models developed predictions of environmental suitability that were positively biased towards regions with higher volunteer sampling effort, often near population centers and developed areas. Whereas, the targeted‐background approach, which has been shown to effectively address sampling bias (Barber et al. 2022), developed more plausible results that were based on a given species' landcover associations as opposed to higher‐intensity sampling effort.

The top model for a given species was determined by comparing Akaike's Information Criterion (AICc; Burnham and Anderson 2002) values among the 20 competing models. Model fit was evaluated using the average AUC values from the five‐fold cross‐validation process. We assessed variable importance and effect using Maxent's output which includes response curves and jackknife tests of variable contributions (Phillips 2010).

#### 2.5.6 Species Selected for Presentation in Results

In order to demonstrate the breadth of results across each of the three modeling strategies (more than 100 species) and validate the framework's capacity to maximize ecological inference across disparate species and data constraints, we randomly selected three species from each of the three SDM approaches to summarize in greater detail within the Results. Species were only considered if their final model had AUC > 0.70, which generally represents a cut‐off for a useful SDM (Swets 1988; Araújo et al. 2005; Stralberg et al. 2025). To ensure these species represented the geographical and ecological diversity of the study area, we constrained the random selection to represent the two major contrasting cover types within Minnesota, forests and prairie‐grasslands, as well as statewide distributions. Consequently, for each of the three modeling strategies, we randomly selected one species with each of these general distributions. For the Generalized Linear Models with QPAD detectability offsets, the focal species selected to exemplify these distributions were Black‐throated Green Warbler ( *Setophaga virens* ), Vesper Sparrow ( *Pooecetes gramineus* ), and Marsh Wren ( *Cistothorus palustris* ). For the Generalized Linear Models without detectability offsets, the representative species included Blue‐gray Gnatcatcher ( *Polioptila caerulea* ), Willow Flycatcher ( *Empidonax traillii* ), and Red‐bellied Woodpecker ( *Melanerpes carolinus* ). Finally, the focal species for the Maxent modeling approach included Broad‐winged Hawk ( *Buteo platypterus* ), Western Kingbird ( *Tyrannus verticalis* ), and Lark Sparrow ( *Chondestes grammacus* ).

## 3 Results

**FIGURE 4:**
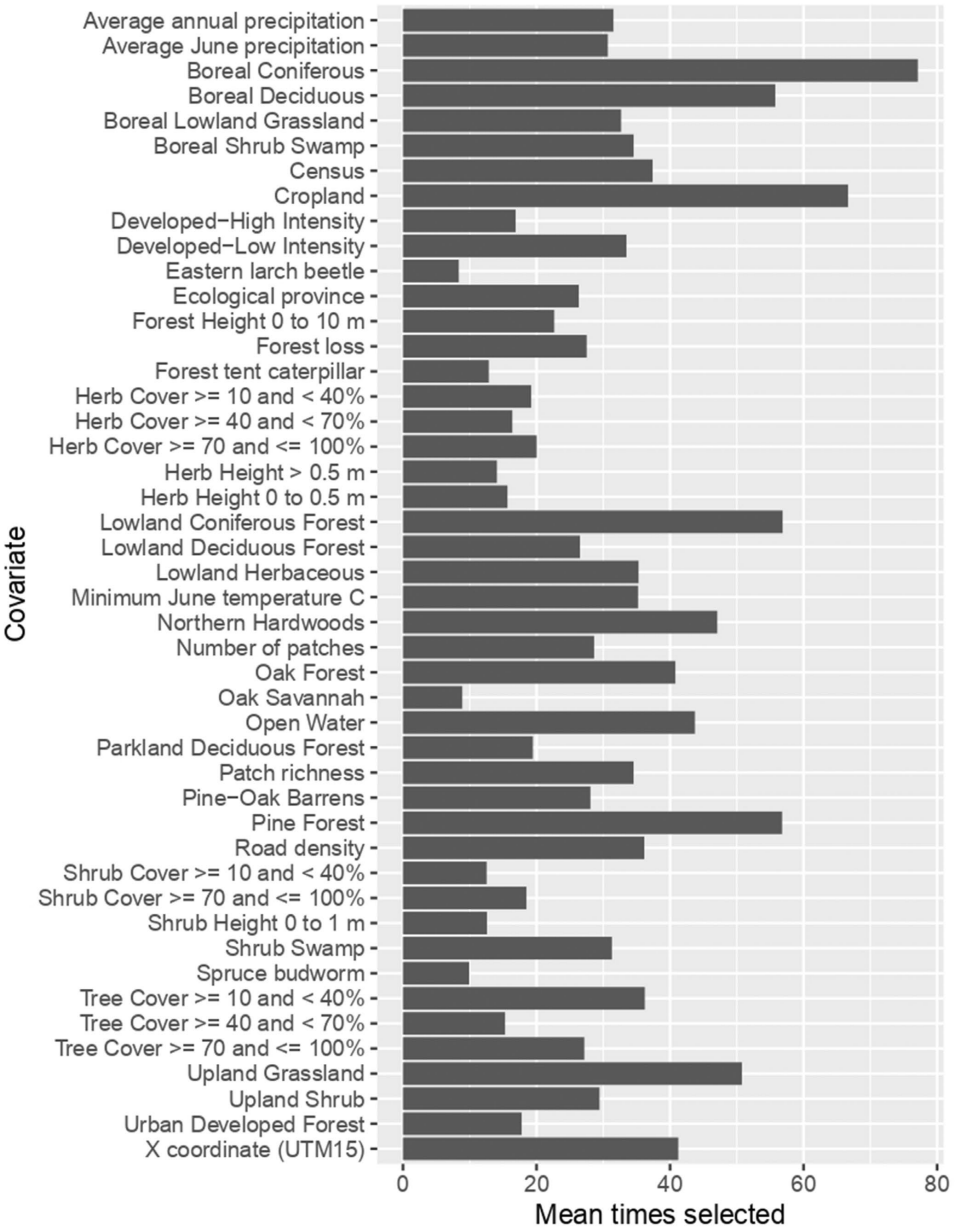
Frequency of covariate selection across 107 species modeled for the Minnesota Breeding Bird Atlas using Generalized Linear Models with or without a QPAD offset. Models for each species consisted of *β* = 240 bootstrap iterations. The mean number of times selected is the number of times a variable is selected across all species divided by 240.

The MNBBA (2009–2013), point‐count observers and citizen scientists reported 379,852 validated records from 250 bird species, with 231 of these species confirmed as nesting (Pfannmuller et al. 2017, 2024). Counters completed a total of 7335 point‐counts at 6921 locations during the MNBBA. An additional 4616 point‐counts were conducted at 961 locations during the supplementary point‐count project (11,951 point‐counts across 7882 point‐count locations in total for approximately 1992 h of sampling). Excluding flyover detections, 245,555 individuals and 233 species were detected during point‐count surveys. Based on the minimum number of detections for each of three potential models, we calculated SDM models for 163 bird species. Of these, 136 species met our modeling criteria of AUC > 0.70. Models are not presented for the 21 species that did not meet the minimal AUC criteria. Overall, AUC for the 136 species with useful models ranged from 0.70 to 0.99 (Table 3, Appendices S3 and S4). Scientific names for all bird species are included in Appendix S2. The most frequently selected variables in GLM with QPAD and GLM models were Boreal Coniferous and Cropland (Figure 4). The 1000 m radius was selected 34 times, 500 m 28 times, and 200 m 41 times (Table 3; Appendix S3). Predicted species distribution maps are available for each species as (Appendix S5).

**TABLE 3:** Nine randomly selected bird species representing the three modeling strategies for Minnesota Breeding Bird Atlas data.

Common name	Model type	Radius (m)	AUC	Detections	Individuals	Mean	SE	Range	State population estimate (lower, upper 95% CI)	PIF state population estimate (lower, upper 95% CI)
Black‐throated Green Warbler^a^	GLM/QPAD	500	0.87	1509	2143	0.18	0.0049	0–5	1,460,000 (1,270,000, 4,000,000)	270,000 (140,000, 440,000)
Vesper Sparrow^b^	GLM/QPAD	200	0.92	1432	2206	0.18	0.0052	0–6	4,810,000 (4,370,000, 5,480,000)	1,100,000 (700,000, 1,700,000)
Marsh Wren^c^	GLM/QPAD	200	0.80	248	542	0.05	0.0034	0–10	2,430,000 (1,590,000, 11,460,000)	460,000 (260,000, 680,000)
Blue‐gray Gnatcatcher^a^	GLM	500	0.93	81	95	0.01	0.0009	0–3		180,000 (28,000, 540,000)
Willow Flycatcher^b^	GLM	500	0.89	69	90	0.01	0.0010	0–4		48,000 (26,000, 77,000)
Red‐bellied Woodpecker^c^	GLM	1000	0.91	361	403	0.03	0.0018	0–3		130,000 (76,000, 210,000)
Broad‐winged Hawk^a^	Maxent	500	0.79	51						64,000 (36,000, 99,000)
Western Kingbird^b^	Maxent	500	0.88	32						17,000 (2900, 44,000)
Lark Sparrow^c^	Maxent	500	0.86	49						2300 (650, 4800)

*Note:* Each strategy had three randomly selected example species, one each with a generally forested^a^, prairie^b^, and statewide^c^ distribution in Minnesota. The number of point‐count locations with non‐flyover detections (Detections), total number of non‐flyover individuals detected (Individuals), mean (Mean) and standard error (SE) of individuals detected, range per point among all 7882 points sampled during the Minnesota Breeding Bird Atlas from 2009 to 2013, predicted state population (species modeled with the GLM/QPAD method only), and PIF state population estimates are included. Note, for GLM/QPAD models, the number of detections and individuals includes only observations of singing males. The number of detections for Maxent models refers to the number of verified high‐level breeding evidence records (probable or confirmed nesting records) used in analyses.

### 3.1 Generalized Linear Models With QPAD Detectability Offset

A total of 73 species used the GLM with QPAD offset and met the AUC cutoff of 0.7 (Appendices S3 and S5). Models for an additional four species did not meet the AUC cutoff and are not included here. Of the 11,951 total point counts, 10,753 were used to build models and 1193 were used to validate models (the remaining five counts were omitted as no QPAD offset was calculated). The number of counts with detections ranged from 52 (Palm Warbler [ *Setophaga palmarum* ]) to 5961 (Red‐eyed Vireo [ *Vireo olivaceus* ]), AUC values ranged from 0.70 (American Redstart [ *Setophaga ruticilla* ]) to 0.98 (Grasshopper Sparrow [ *Ammodramus savannarum* ]), and the median number of covariates selected in the 240 species‐specific models ranged from three (Purple Finch [ *Haemorhous purpureus* ]) to 27 (Ovenbird [ *Seiurus aurocapilla* ] and White‐throated Sparrow [ *Zonotrichia albicollis* ]; Appendix S3). The three randomly selected species (Black‐throated Green Warbler, Vesper Sparrow, Marsh Wren) were generally representative of the broader group; the number of counts with detections ranged from 248 to 1509, AUC varied from 0.87 to 0.92, and the number of covariates from 11 to 24 (Tables 3 and 4).

**TABLE 4:** Summary of Minnesota Breeding Bird Atlas models for six species using Generalized Linear Models (GLM) with and without a QPAD offset (Sólymos et al. 2013).

Common name	Model type	Number of covariates	Covariates
Black‐throated Green Warbler^a^	GLM/QPAD	24	Cropland (−6.7, −2.45), Boreal Shrub Swamp (−0.73, −0.24), Lowland Deciduous Forest (−0.74, −0.21), Pine‐Oak Barrens (−0.69, −0.23), Number of patches (0.27, 0.48), Tree Cover > = 70 and < = 100% (0.08, 0.43), Patch richness (−0.41, −0.18), Forest loss (−0.12, −0.01)
Vesper Sparrow^b^	GLM/QPAD	11	Cropland (0.97, 1.9), Boreal Coniferous (−1.79, −0.32)
Marsh Wren^c^	GLM/QPAD	19	Open Water (0.57, 1.68), Lowland Herbaceous (0.24, 0.68)
Blue‐gray Gnatcatcher^a^	GLM	6	Oak Forest (0.08, 0.61)
Willow Flycatcher^b^	GLM	5	Lowland Herbaceous (0, 0.57)
Red‐bellied Woodpecker^c^	GLM	11	Parkland Deciduous Forest (−587.74, −2.04), Boreal Coniferous (−3.66, −0.48), Pine Forest (−1.09, −0.18), Oak Forest (0.14, 0.3)

*Note:* Each strategy had three randomly selected species, one each with a generally forested^a^, prairie^b^, and statewide^c^ distribution in Minnesota. Covariates were included in this table only if they were selected in all bootstrap models and their 95% CI did not overlap zero. An exception was made for Willow Flycatcher, which had no covariates that met these criteria. For this species, covariates were included in this table only if the median parameter estimate was non‐zero and the 95% CI did not extend past zero.

**FIGURE 5:**
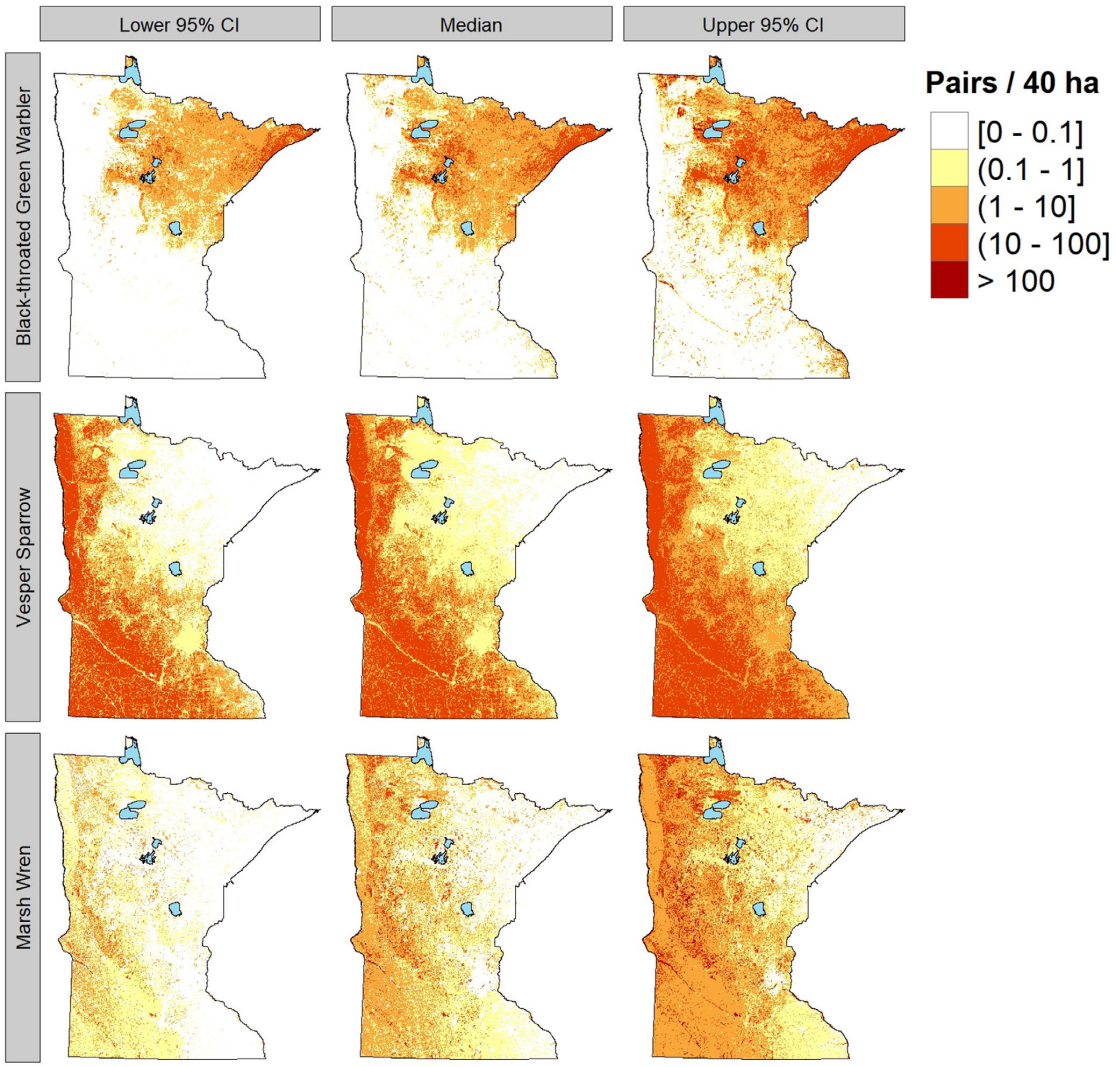
Three example species representing the Generalized Linear Model with QPAD offset strategy used for 73 bird species for the Minnesota Breeding Bird Atlas. Predicted values are in pairs per 40 ha. Also shown is the non‐parametric bootstrap 95% confidence interval.

We mapped the median predicted density (pairs/40 ha) and their corresponding 95% confidence intervals for all 73 species (e.g., Figure 5). Black‐throated Green Warbler was predicted to be most abundant in the northeastern region of the state. Cropland was the driving covariate for this species with a negative association. Other negative associations included boreal shrub swamp, lowland deciduous forest, and pine‐oak barrens. Positive associations were tree cover 70%–100% and patch richness (Table 4, Figure 5). In contrast, Vesper Sparrow was positively associated with cropland and negatively associated with boreal coniferous cover resulting in a distribution throughout the open habitats of the southern and western portions of the state (Table 4, Figure 5). Marsh Wren was predicted to be most abundant in the western half of Minnesota where it was positively associated with open water and lowland herbaceous habitats (Table 4, Figure 5).

Statewide population estimates were limited to bird species modeled with the GLM with QPAD detectability offsets and ranged from 39,200 adult Black‐billed Cuckoo to 15,600,000 for Horned Lark (Appendix S6). The corresponding estimates for Minnesota based on Partners in Flight (PIF; 2020) were 120,000 for the Black‐billed Cuckoo and 1,100,000 for Horned Lark (Appendix S6). The confidence intervals for both of these species overlapped with PIF's. Population estimates for the three randomly selected species using GLM with the QPAD offset did not overlap with the PIF (2020) population estimates; our population estimates were higher for all three species (Table 3). While several species such as Black‐billed Cuckoo, Alder Flycatcher, Eastern Phoebe, White‐throated Sparrow, and Western Meadowlark had confidence intervals that overlapped, for the majority of species (57) the MNBBA population estimates were substantially higher than those estimated by PIF (2020) (Table 3; Appendix S6).

### 3.2 Generalized Linear Models Without Detectability Offset

We attempted to model 37 species using the GLM approach. Of these, seven either did not meet the AUC cutoff or had higher AUC with the Maxent approach. Thus, 30 species were included (Appendices S3 and S5). All 11,951 point counts were used in this analysis (10,758 for model building and 1193 for validation). The number of counts with detections ranged from 24 (Bank Swallow [ *Riparia riparia* ]) to 2174 (Mourning Dove [ *Zenaida macroura* ]), AUC values varied from 0.70 (Yellow‐bellied Sapsucker [ *Sphyrapicus varius* ]) to 0.99 (Black‐billed Magpie [ *Pica hudsonia* ]), and the median number of covariates ranged from four (Ruby‐throated Hummingbird [ *Archilochus colubris* ] and Red‐tailed Hawk [ *Buteo jamaicensis* ]) to 21 (Sandhill Crane [*Antigone canadensis*]; Appendix S3). The three randomly selected species (Blue‐gray Gnatcatcher, Willow Flycatcher, Red‐bellied Woodpecker) had counts with detections ranging from 69 to 172, AUC from 0.77 to 0.93, and the number of covariates included in the best models varied from 5 to 11 (Tables 3 and 4).

**FIGURE 6:**
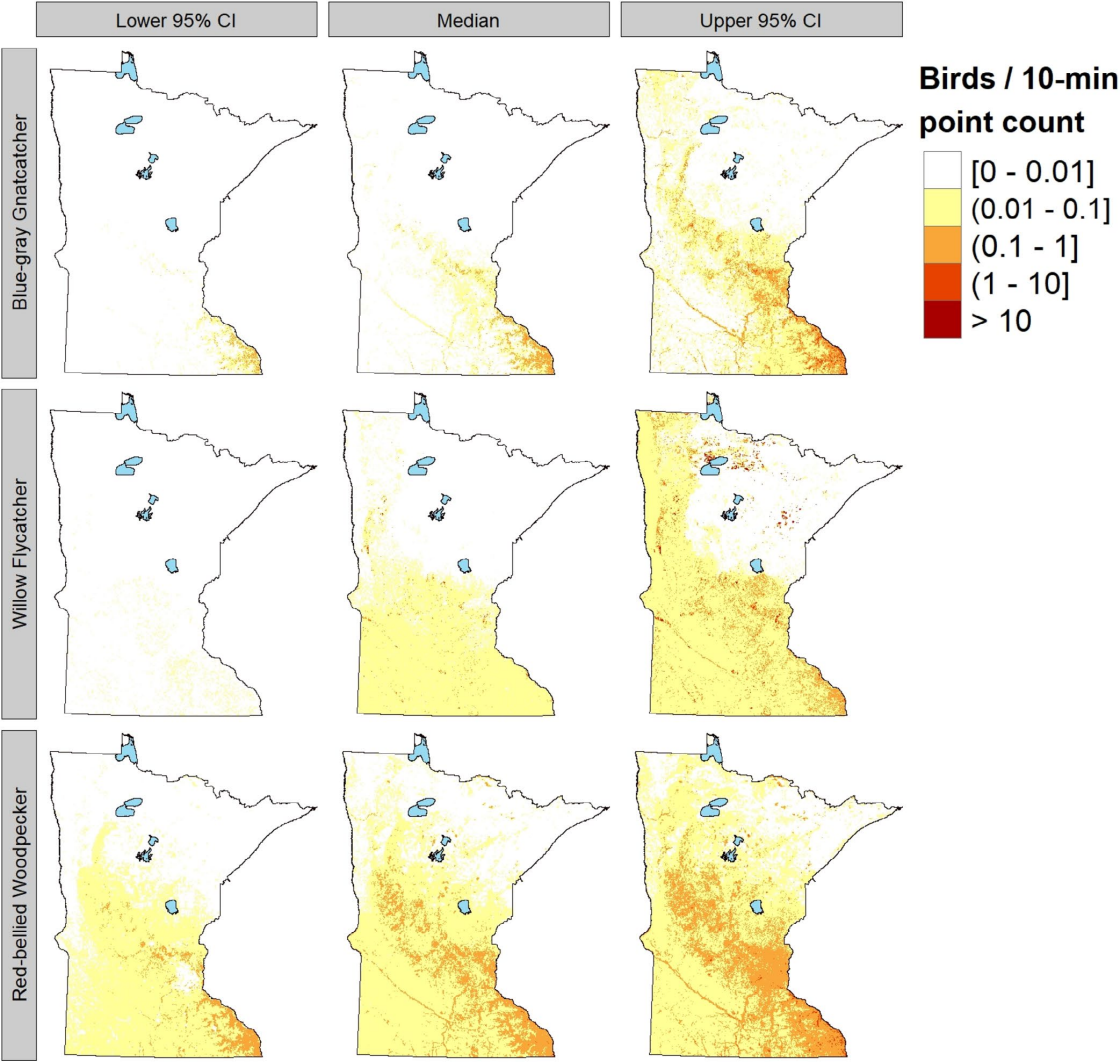
Three example species representing the Generalized Linear Model strategy used for 30 bird species for the Minnesota Breeding Bird Atlas. Predicted values are in expected individuals per 10‐min point count. Also shown is the non‐parametric bootstrap 95% confidence interval.

We mapped the median expected number of individuals detected during a 10‐min point‐count survey and the corresponding 95% confidence intervals for each of the 30 species (e.g., Figure 6). Blue‐gray Gnatcatcher was predicted to be most abundant in the southeastern portion of Minnesota where it had a positive association with oak forests (Table 4, Figure 6). Willow Flycatcher was predicted to be most abundant in the southwestern region of Minnesota where abundance was positively associated with lowland herbaceous habitats (Table 4, Figure 6). Red‐bellied Woodpecker was predicted to be distributed across much of the state, but less abundant in the northeast. This species was negatively associated with parkland deciduous, boreal coniferous, and pine forests, but positively associated with oak forest (Table 4, Figure 6).

### 3.3 Maxent

We modeled 42 species using the Maxent approach. Nine did not meet the AUC cutoff (or had higher AUC with GLM models), leaving 33 for inclusion (Appendices S4 and S5). The sample size ranged from 20 (Blue‐winged Warbler [ *Vermivora cyanoptera* ]) to 364 (Cliff Swallow [ *Petrochelidon pyrrhonota* ]) probable or confirmed breeding records. AUC values for the Maxent models ranged from 0.72 (Ring‐necked Duck [ *Aythya collaris* ]) to 0.97 (Gray Partridge [ *Perdix perdix* ]; Appendix S4). Twenty‐one of these models used linear features, and 12 used linear and quadratic features. The regularization parameter ranged from 1 to 10.

The focal species for the Maxent approach (Broad‐winged Hawk, Western Kingbird, and Lark Sparrow) had between 32 and 51 georeferenced locations of high‐level breeding evidence, AUC values ranging from 0.79 to 0.88, and models that included linear‐only or linear and quadratic features (Tables 3 and 5).

**TABLE 5:** Model summary for three species modeled with Maxent as part of Minnesota Breeding Bird Atlas.

Common name	Features	Regularization multiplier	Covariates
Broad‐winged Hawk^1^	Linear	10	(−) Cropland, (−) Upland Grassland, (+) Boreal Deciduous
Western Kingbird^2^	Linear	10	(−) Northern Hardwood Forest, (−) Lowland Coniferous Forest, (−) Marsh, (+) Cropland, (−) Oak Forest
Lark Sparrow^3^	Linear, qaudratic	7	(q) Open Water, (−) Marsh, (q) Boreal Deciduous, (−) Bog, (q) Lowland Coniferous Forest, (q) Developed‐High Intensity, (−) Boreal Shrub Swamp

*Note:* Three randomly selected species using the Maxent modeling method, one each with a generally forested^1^, prairie^2^, and statewide^3^ distribution in Minnesota. Maxent features and regularization multipliers were determined via a model selection process described in the text. All covariates with greater than 5% contribution to the final Maxent model, as well as the direction of effect for the given variable [(+) positive; (−) negative; (q) quadratic relationship], are included.

**FIGURE 7:**
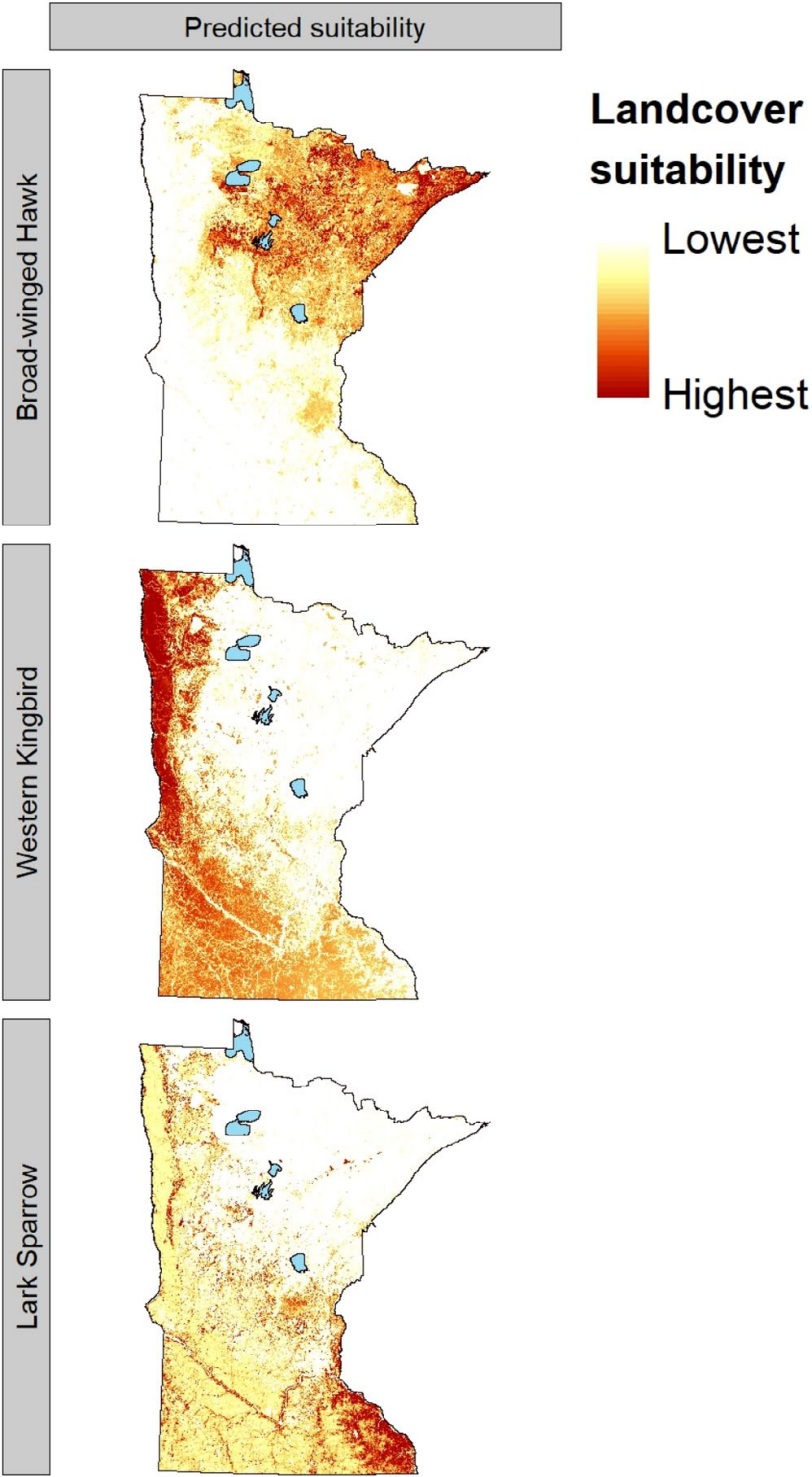
Three example species representing the Maxent strategy used for 33 bird species for the Minnesota Breeding Bird Atlas. Predicted values are in land cover suitability, from lowest suitability to highest suitability.

For the Broad‐winged Hawk, predicted environmental suitability was highest in Minnesota's forested northeastern region, driven by a strong positive association with boreal deciduous forest and a corresponding negative association with open habitats such as cropland and upland grassland (Table 5, Figure 7). In contrast, suitability for the Western Kingbird was predicted to be highest along the western and southern portions of the state, where it was positively associated with cropland and negatively associated with forest types including Northern hardwood forest and lowland coniferous forest (Table 5, Figure 7). Finally, the Lark Sparrow exhibited a more localized and dispersed distribution, with the highest environmental suitability in southeastern Minnesota and scattered areas across the central and western regions. This species’ model included more complex transformations of covariates, including a quadratic association with open water and high‐intensity development, alongside a negative association with wetland cover types such as marshes and bogs (Table 5, Figure 7).

## 4 Discussion

In this study, we developed a modeling framework to maximize the utility of MNBBA data, producing SDMs for 136 bird species breeding in Minnesota (59% of species). By assigning species to one of three modeling strategies, ranging from rigorous density‐based GLMs to suitability‐based Maxtent models, we were able to generate population estimates for 73 species and indices of abundance or suitability for an additional 63 species. Our results demonstrate that a flexible, multi‐tiered approach allows for a more comprehensive assessment of avifauna than any single method could provide. These efforts provide critical information for current population estimates for many species that can be used as a baseline to compare changes in populations as subsequent BBA efforts are completed. Furthermore, our models provide information about species‐specific relationships with biophysical variables that can help improve the understanding of distribution, habitat, and landscape requirements that can be used for habitat management and conservation efforts.

Several recent breeding bird atlases have produced SDM models, including Ontario (Cadman et al. 2007), British Columbia (Davidson et al. 2015), and Pennsylvania (Wilson et al. 2012). For example, Cadman et al. (2007) used data from 5‐min point counts to produce both SDMs and species population estimates during its second BBA in Ontario. They used both ordinary and supervised manual kriging to produce maps on a relative abundance scale. They also estimated populations of 124 species using the same methods as PIF (Blancher et al. 2007). Wilson et al. (2012) and Rodewald et al. (2016) used 6.25‐min point counts from the second Pennsylvania and Ohio BBAs, respectively, to extend regression kriging using removal (Farnsworth et al. 2002) and distance (Farnsworth et al. 2006) models. They included analysis of detectability with removal and distance models to estimate populations for 66 species.

One of the potential strengths in applications of the SDMs is their use in future management and conservation planning within state, provincial, country, or other political jurisdiction. For example, in Minnesota, more than 700 citizen scientists and experienced field ornithologists participated in the gathering of bird data. In total, they recorded 379,852 acceptable detections and point counts resulted in an additional 245,555 acceptable detections used in SDM models. If we assume point‐counts on average cover a radius of 150 m, then 7882 point counts cover approximately 557 km^2^ or about 0.25% of Minnesota. Thus, despite most areas of the state being unsampled by point counts, the systematic sampling design coupled with SDMs provided predictions on the potential suitability and abundance for bird species across all of Minnesota.

Even though there are limitations to the inferences that are possible from these analyses, an appropriate breeding bird atlas sampling and analytical framework provided a process for improvements and a wealth of management and conservation actions for implementation in the future. Many breeding bird atlases are repeated on a 15 to 20‐year cycle. For instance, the Canadian Province of Ontario has completed its 3rd atlas project in 2021–2025, following previous atlases in 1981–1985 and 2001–2005 (Birds Canada 2021). Repeated atlases, using the same methodology, can provide a means to refine SDMs, test specific a priori hypotheses on the distribution and abundance of breeding bird species, and assess distributional and population changes over time.

Point‐count surveys are the most widely used standardized method for counting terrestrial birds (Matsuoka et al. 2014). The methodology is efficient in terms of the number of species and individuals detected per unit time. For example, the mean number of species detected per 10‐min point (excluding fly‐overs) in Minnesota was 11.78 (SE 0.04, range of 1 to 28) and the mean number of individuals was 20.55 (SE 0.11, range 1 to 356). Gathering data on point counts is species dependent. For example, abundance data can be obtained for common, easily detected species, whereas presence/absence data may be the only data obtained for less common or hard to detect species. Abundance data have been shown to improve the accuracy of SDMs (Howard et al. 2014). Opportunistic records of species occurrence or breeding evidence often constitute the bulk of data collected in atlases and SDMs often rely on presence‐only or presence/absence data (Guisan and Zimmermann 2000; Brotons et al. 2004; Elith et al. 2006; Pearson et al. 2006). We successfully applied both of these types of data using three different modeling approaches. The GLM approaches with and without offsets allowed us to use point count abundance data, while Maxent allowed us to also use presence data from citizen scientists, as long as georeferenced data were available. We recommend more citizen scientists be encouraged and trained to record the locations of their observations, especially for rare species where each observation is valuable data (Sullivan et al. 2014).

Many statistical approaches have been used to develop SDMs with breeding bird data (Franklin 2009). One of the most common methods utilizes GLMs to estimate environmental covariates across space. This approach requires count or presence‐absence data to generate statistical functions to rank habitat suitability (Manel et al. 1999; Guisan and Zimmermann 2000; Brotons et al. 2004). In our GLM mapped projections, we applied them either as a density of pairs per 40 ha (GLM with offset) or as an index of abundance (bird detections per 10 min point count; GLM no offset). Moreover, the display of the mapped projections of a species or suite of species abundances can be examined at ever finer scales of spatial resolution (e.g., county, township, or sub‐township), thereby identifying specific habitats of potential importance.

Machine learning approaches, like Maxent or boosted regression trees, have been used to model avian distribution and abundance over large scales. At large spatial (e.g., continental) scales variable importance and effect sizes vary across space and machine learning algorithms tend to excel (e.g., Leston et al. 2024; Stralberg et al. 2025). However, at the scale of states or provinces, such geographic variation is not as important, of a consideration, which makes GLM approaches equally suitable (Ball et al. 2016; Westwood et al. 2019). The ease of interpretation of GLM model coefficients and portability of the results make GLMs ideal for many applications. For example, estimated model coefficients can be used in landscape simulations to test for potential effects of land use changes (Mahon et al. 2014; Cadieux et al. 2020; Leston et al. 2020).

All of our population estimates were either higher or had overlapping confidence intervals when compared to those presented by PIF (2020) for the state of Minnesota. PIF (2020) primarily uses the federal North American Breeding Bird Survey (Sauer et al. 2019) which is based on roadside counts where many attributes associated with species' detectability and distance to detection are not explicitly collected for each count. Thogmartin (2010), Sólymos, Matsuoka, et al. (2018); Sólymos et al. (2020), and Edwards et al. (2023) have all emphasized that species population estimates that quantify species detectability and distance to detection result in population estimates that are higher than those that include expert judgment‐based adjustment factors (Stanton et al. 2019). Current PIF population estimates use expert‐driven estimates of maximum detection distance for each species, which were found to drive the overall differences between distance sampling‐based versus maximum detection distance‐based population estimates (Sólymos et al. 2020). Our approach of using time‐removal models to account for availability bias is also distinct from the time‐adjustment used in PIF population estimates, which are based on fitting a smooth curve to species counts over time‐of‐day (Stanton et al. 2019). Lastly, our assumption of a 1:1 male‐to‐female ratio used to derive the population estimates may vary across species, requiring species‐specific adjustments rather than a blanket pair adjustment multiplier. Accounting for uncertainty in the pair adjustment can be done in the future using a Monte Carlo approach to propagate uncertainty using a distribution of plausible expert judgment‐based values, for example, as in Stanton et al. (2019). However, it's important to note that it is unknown whether these higher estimates are closer to the truth (Sólymos et al. 2020) or whether they are potentially overestimates due to formulation of the offsets that treats availability and perceptibility as independent processes (Martin‐Schwarze et al. 2021; Lele and Sólymos 2026).

Sólymos et al. (2020) highlighted the potential bias of population estimates derived from only roadside counts, especially in areas with expansive roadless areas. Thus, it's possible that some of the larger discrepancies between MNBBA and PIF (2020) population estimates were related to the additional counts the MNBBA completed in roadless, forested and peatland areas (as well as off‐road counts included from the Minnesota National Forest Breeding Bird Program). Two primary roadless areas include the Boundary Waters Canoe Area Wilderness (BWCAW) and the Agassiz Lowland Ecological Subsection (ALES). The BWCAW (4,411 km^2^) and ALES (50% of which is peatland or 7500 km^2^) comprise about 11,611 km^2^. Many of the species that have large discrepancies between MNBBA and PIF (2020) such as the Nashville Warbler [9,300,000 MNBBA:2,200,000 PIF (2020)], Yellow‐rumped Warbler (3,180,000:350,000), Yellow‐bellied Flycatcher (731,000: 170,000), Connecticut Warbler (488,000:47,000), and Dark‐eyed Junco (115,000:22,000) are commonly found in these regions (Appendix S6). In addition, Hanowski and Niemi (1995) found that many species associated with lowland coniferous forests, like those identified here with large estimated population discrepancies, were undercounted in a paired comparison of point counts on roads with those > 200 m from roads.

We used Maxent to model the distributions of species that were uncommon or poorly surveyed by point‐count methodology, and thus, could not be modeled with the more comprehensive GLMs. The resulting models represent an index of environmental suitability for a given species based on environmental covariates and presence locations (Merow et al. 2013). Consequently, the scale of model output does not represent a quantitative measure of abundance and cannot be readily compared among species. However, due to the nature of citizen science data collected in BBAs, Maxent provides a solution to model the distributions of many additional species and their associated habitat characteristics that would otherwise be omitted (Elith et al. 2011).

In our case, we were also able to model presence locations of higher order breeding evidence. For example, of the 268 locations used for modeling the Bald Eagle, 89% represented locations of active nests and thus the resultant suitability model predicts the suitability of areas as nesting habitat for the species, as opposed to more general “breeding habitat”. When sequential BBAs have been conducted across time, the distributions of a given species can be compared to identify potential losses or gains in suitable habitat (e.g., Khanum et al. 2013).

Overall, we recommend that BBAs and other large‐scale citizen‐science datasets consider diverse modeling strategies, including presence‐only modeling such as Maxent. However, users must be aware of and adhere to guidelines for using these techniques. With Maxent in particular, addressing sampling bias, tuning of model parameters, and careful interpretation of outputs are of considerable importance (Warren and Seifert 2011; Kramer‐Schadt et al. 2013; Merow et al. 2013; Muscarella et al. 2014).

## 5 Conclusions

Region‐wide point counts and BBA data provide powerful inputs to statistical models for predicting the distribution and abundance of multiple avian species. We found that a combination of GLM with an offset for detectability, GLM, and Maxent provided an opportunity to model most breeding bird species in Minnesota. We recommend using multiple approaches that support the inclusion of systematic point counts and volunteer observations when attempting to create SDMs for many species across a relatively large geographic range. Further, the use of multiple approaches allowed us to assess important environmental variables for both common species and uncommon species. Breeding bird atlas surveys provide an opportunity to engage citizens in the scientific process and to promote public awareness and advancements in bird conservation. Identifying modeling approaches that incorporate volunteer data is an important consideration for BBA efforts and can help encourage individuals to take action to protect bird habitats in their communities. Systematic, replicable survey methodology is also important for providing comprehensive baseline data on the distribution, abundance, and breeding status of bird species. These datasets provide an opportunity to assess how birds are responding to changes in habitat and climate over time. Overall, BBAs and SDMs are critical tools for monitoring, conserving, and improving our ecological understanding of bird populations and their habitats. When combined, they can enhance data used for conservation planning, future research, and policy development, ultimately contributing to the preservation of avian biodiversity worldwide.

## Author Contributions

**Nicholas G. Walton:** conceptualization (supporting), data curation (lead), formal analysis (lead), writing – original draft (lead). **Edmund J. Zlonis:** conceptualization (equal), data curation (equal), formal analysis (lead), writing – original draft (equal). **Péter Sólymos:** formal analysis (supporting), methodology (supporting), writing – review and editing (supporting). **Alexis R. Grinde:** funding acquisition (supporting), writing – review and editing (equal). **Gerald J. Niemi:** conceptualization (lead), funding acquisition (lead), methodology (equal), writing – original draft (equal).

## Funding

This work was supported by the Minnesota Environment and Natural Resources Trust Fund, ML 2010, Chap.362, Sec.2, Subd.3c.

## Conflicts of Interest

The authors declare no conflicts of interest.

## Supporting information


**Appendix S1:** LANDFIRE Existing Vegetation Type (EVT version LF 2010/LF 1.2.0) reclassification crosswalk. Each LANDFIRE cover type has multiple attributes associated with it.
**Appendix S2:** Name and model type applied to 136 bird species modeled as part of the Minnesota Breeding Bird Atlas. Species with an asterisk* were modeled with both Maxent and GLM. The model with the highest AUC was reported.
**Appendix S3:** Summary of parameter estimates for all species modeled with a GLM (with or without QPAD). Only covariates that were selected in all bootstrap iterations (*b* = 240) are shown here. The exception is for species that had no variables meeting this criteria; in this case only the most frequent variable(s) is shown here. See supplemental material for a complete listing of model parameters. Detections refers to the number of point counts with non‐flyover observations for a given species. For GLM/QPAD species, this only includes observations of singing males.
**Appendix S4:** Model summary for bird species modeled with MaxEnt as part of Minnesota Breeding Bird Atlas, 2009–2013. Only species with useful models (AUC > 0.70) are included. Twenty combinations of MaxEnt features and regularization multipliers were compared via a model selection process implemented with the R package ENMeval (Muscarella et al. 2014). All covariates with greater than 5% contribution to the final MaxEnt model, as well as the direction of effect for the given variable [(+) positive; (−) negative; (q) quadratic relationship], are included. Variables are listed in descending order of importance. The final models and all associated MaxEnt outputs (e.g., response curves, crossvalidation results) are available in supplemental material. Records refers to the number of verified high‐level breeding evidence records (probable or confirmed nesting records) used in analyses.
**Appendix S6:** Statewide population estimates for 73 bird species using MNBBA point count data, their 95% confidence interval, and PIF population estimates.

**Appendix S5:** Species distribution maps for 136 species modeled as part of the Minnesota Breeding Bird Atlas. Species are grouped by model type.

## Data Availability

Data and statistical code are permanently archived in the Data Repository for the University of Minnesota (DRUM) and are available via this permalink: https://hdl.handle.net/11299/277686.
