# Supplementary material for: Species Distribution Models and Abundance Estimates Enhance Breeding Bird Atlas Data
Source: Ecol Evol. 2026 Jun 8;16(6):e73808. doi: 10.1002/ece3.73808 (PMC13244075; doi:10.1002/ece3.73808)

Journal name: Ecology and Evolution
Title: Species Distribution Models and Abundance Estimates enhance Breeding Bird Atlas Data
Nicholas G. Walton, Edmund J. Zlonis, Péter Sólymos, Alexis R. Grinde, Gerald J. Niemi

Appendix S5. Species distribution maps for 136 species modelled as part of the Minnesota Breeding Bird Atlas. Species are grouped by model type.

GLM without QPAD offset (30 species)

Barn Swallow *Hirundo rustica*

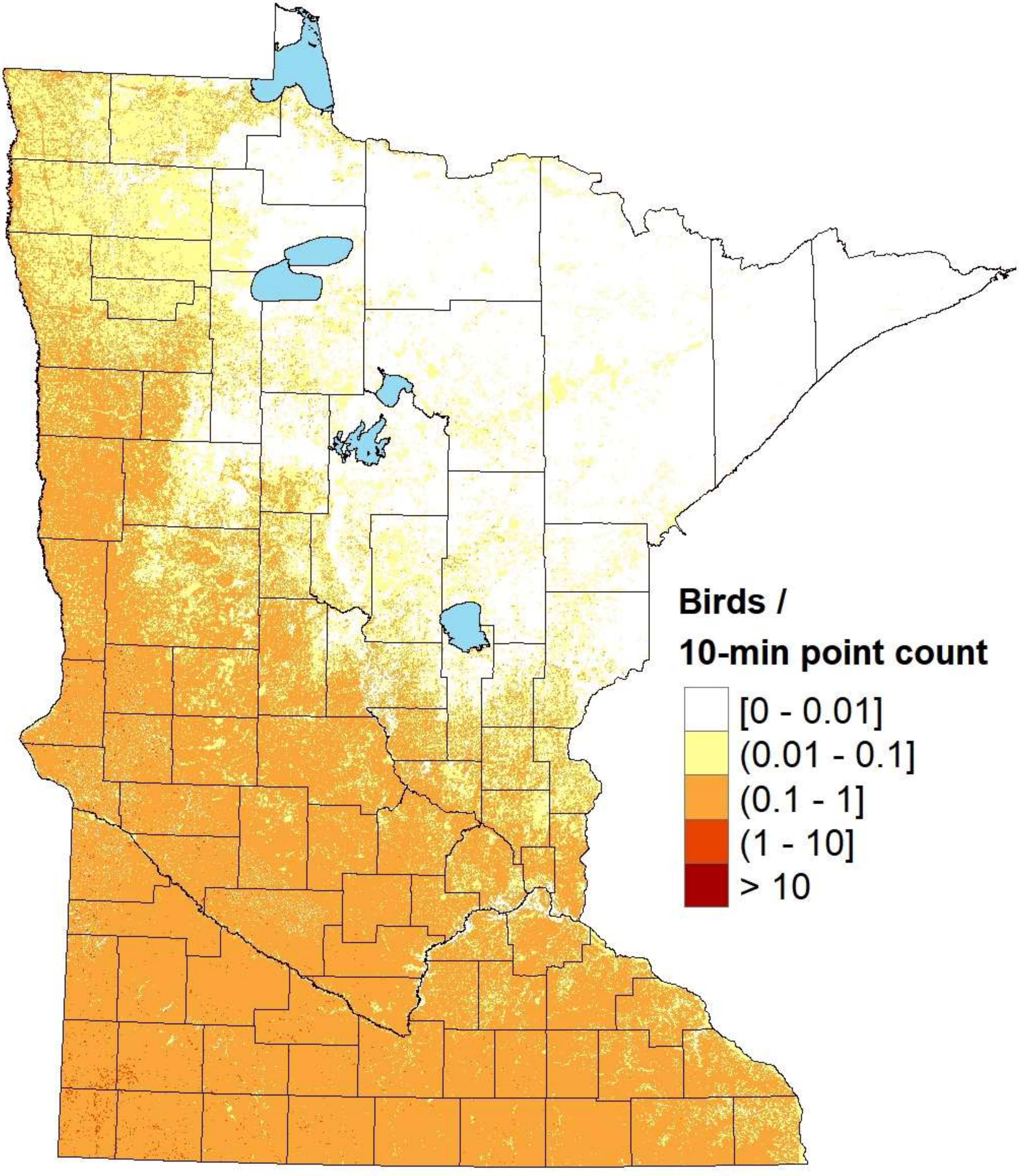

Black-billed Magpie *Pica hudsonia*

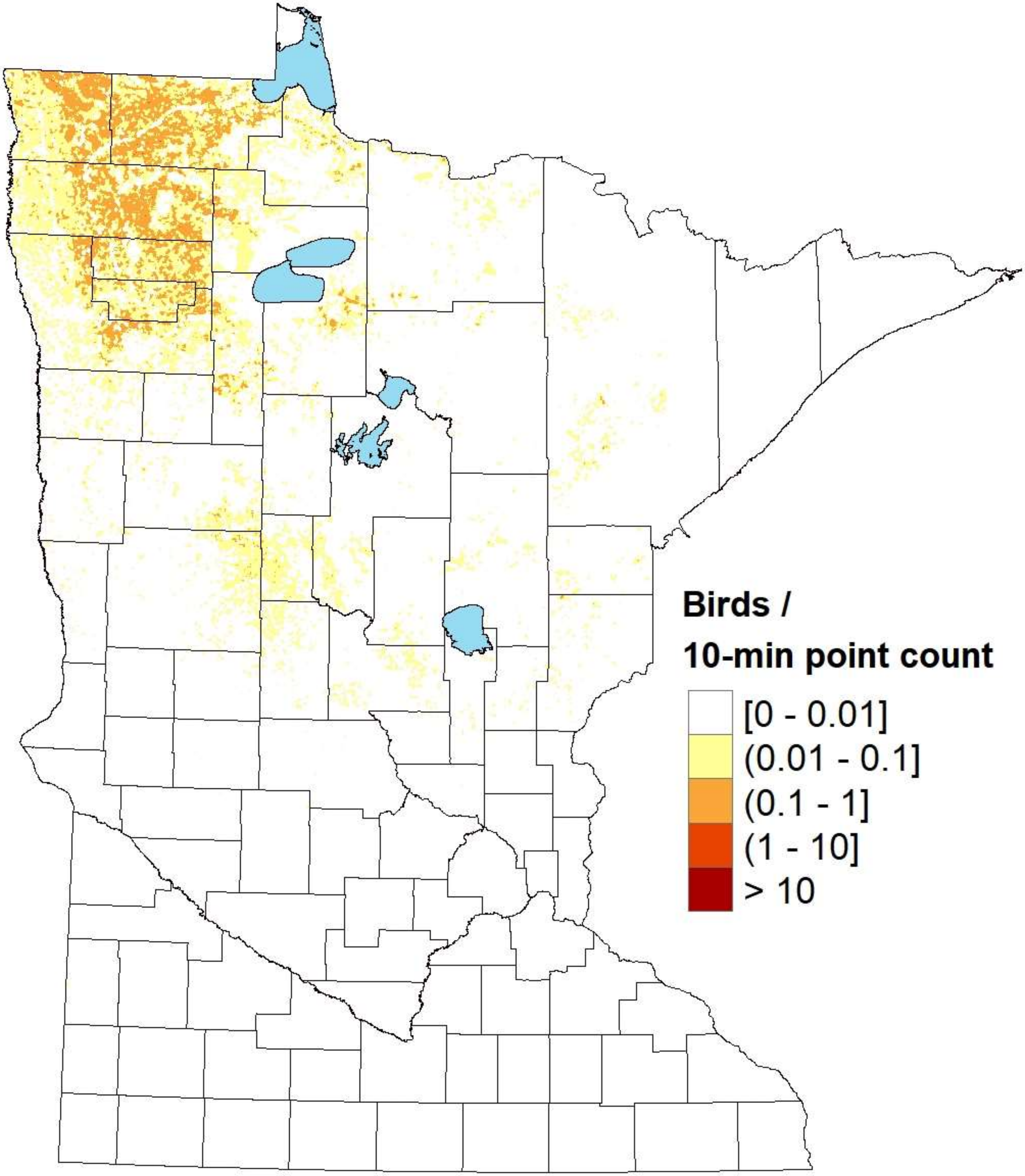

Blue-gray Gnatcatcher *Polioptila caerulea*

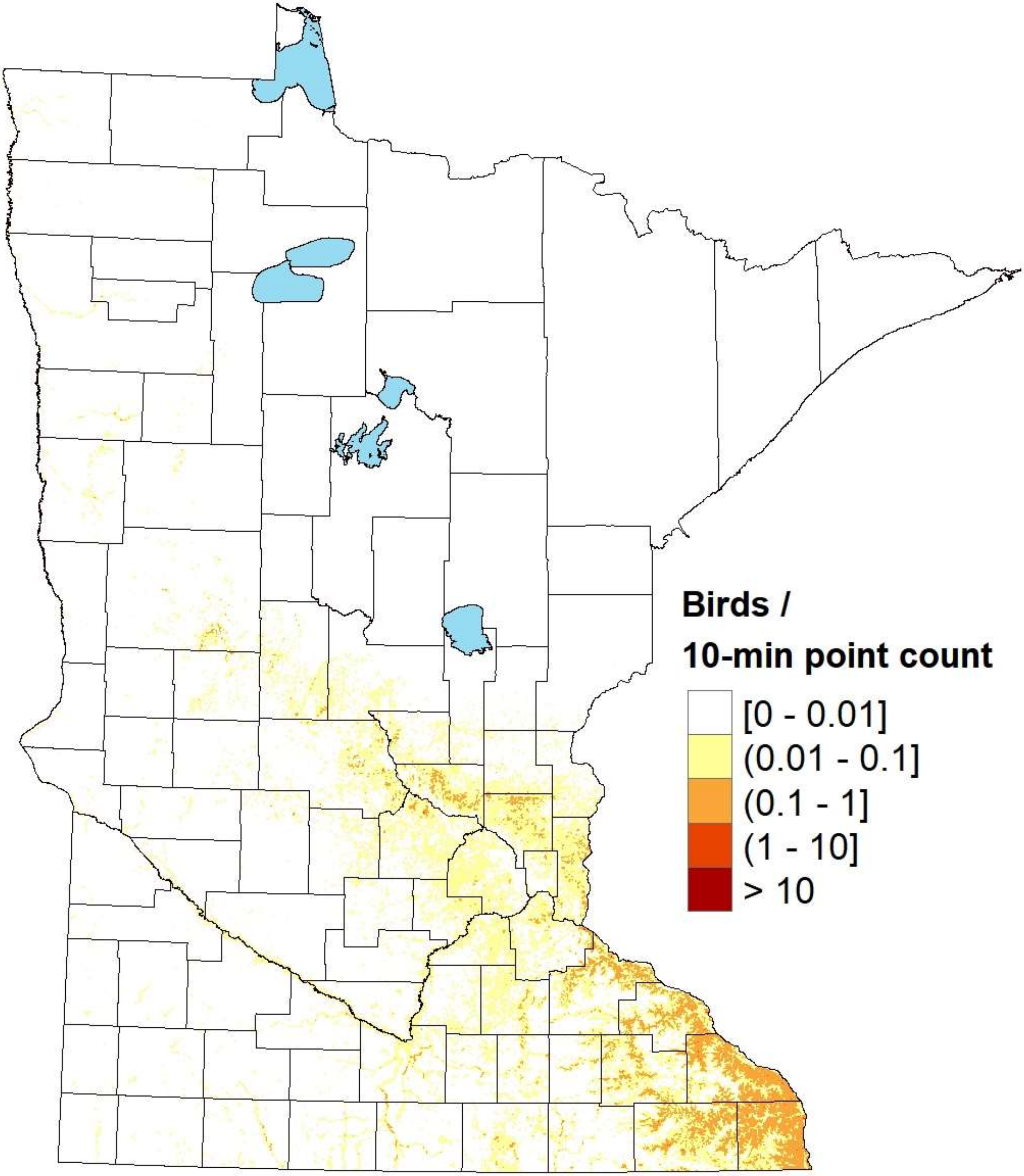

Blue-winged Teal *Spatula discors*

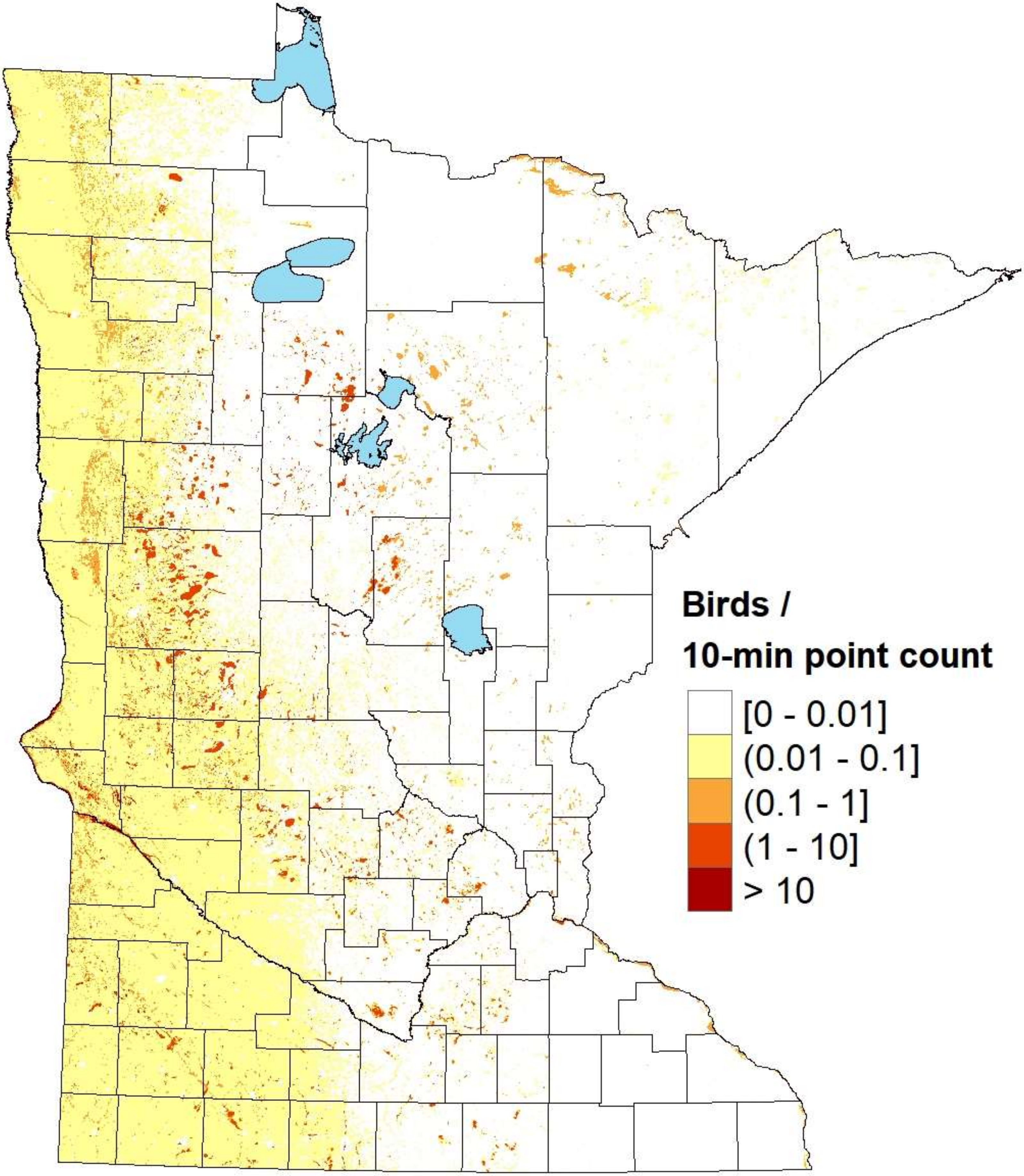

Boreal Chickadee *Poecile hudsonicus*

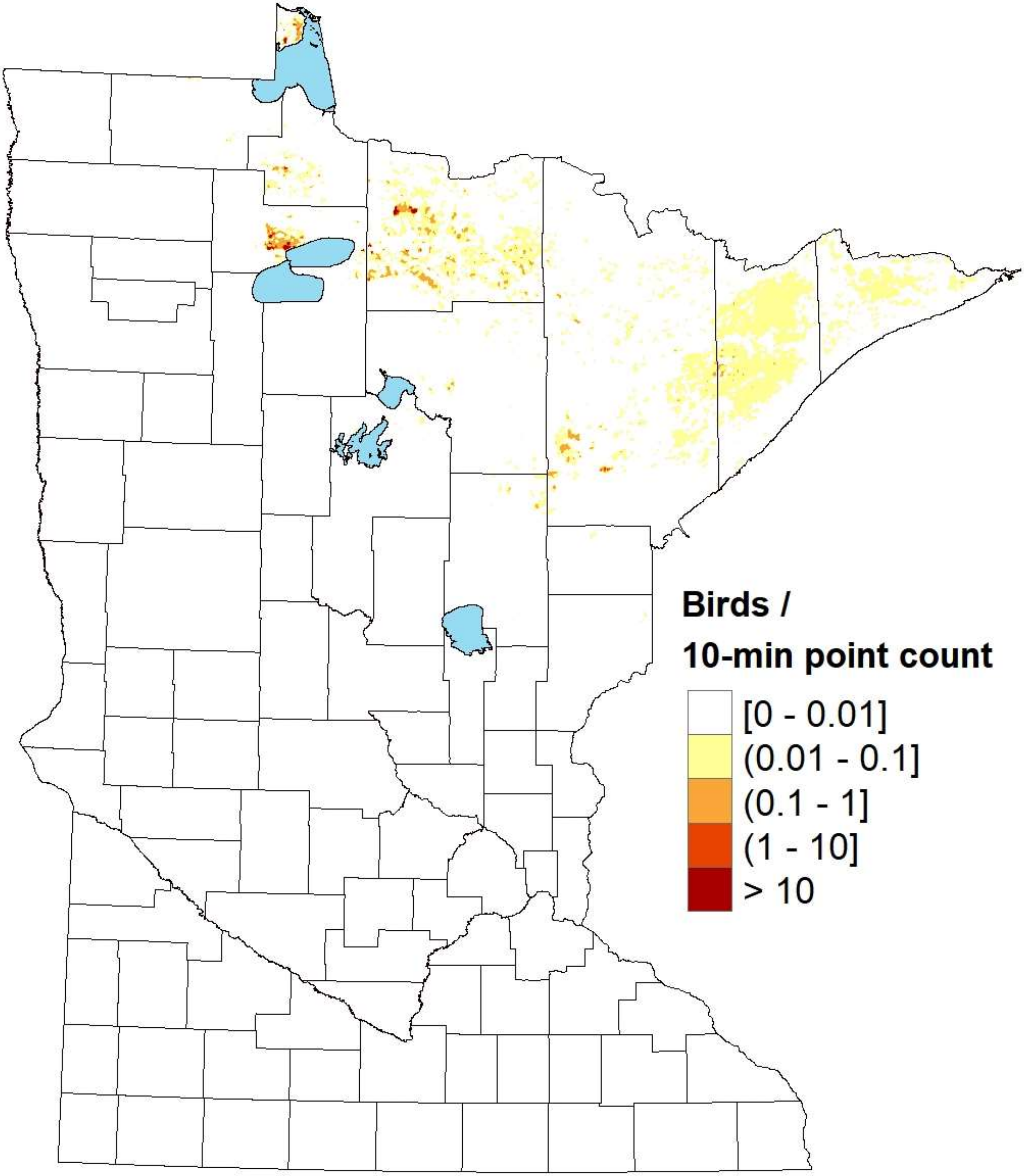

Brewer's Blackbird *Euphagus cyanocephalus*

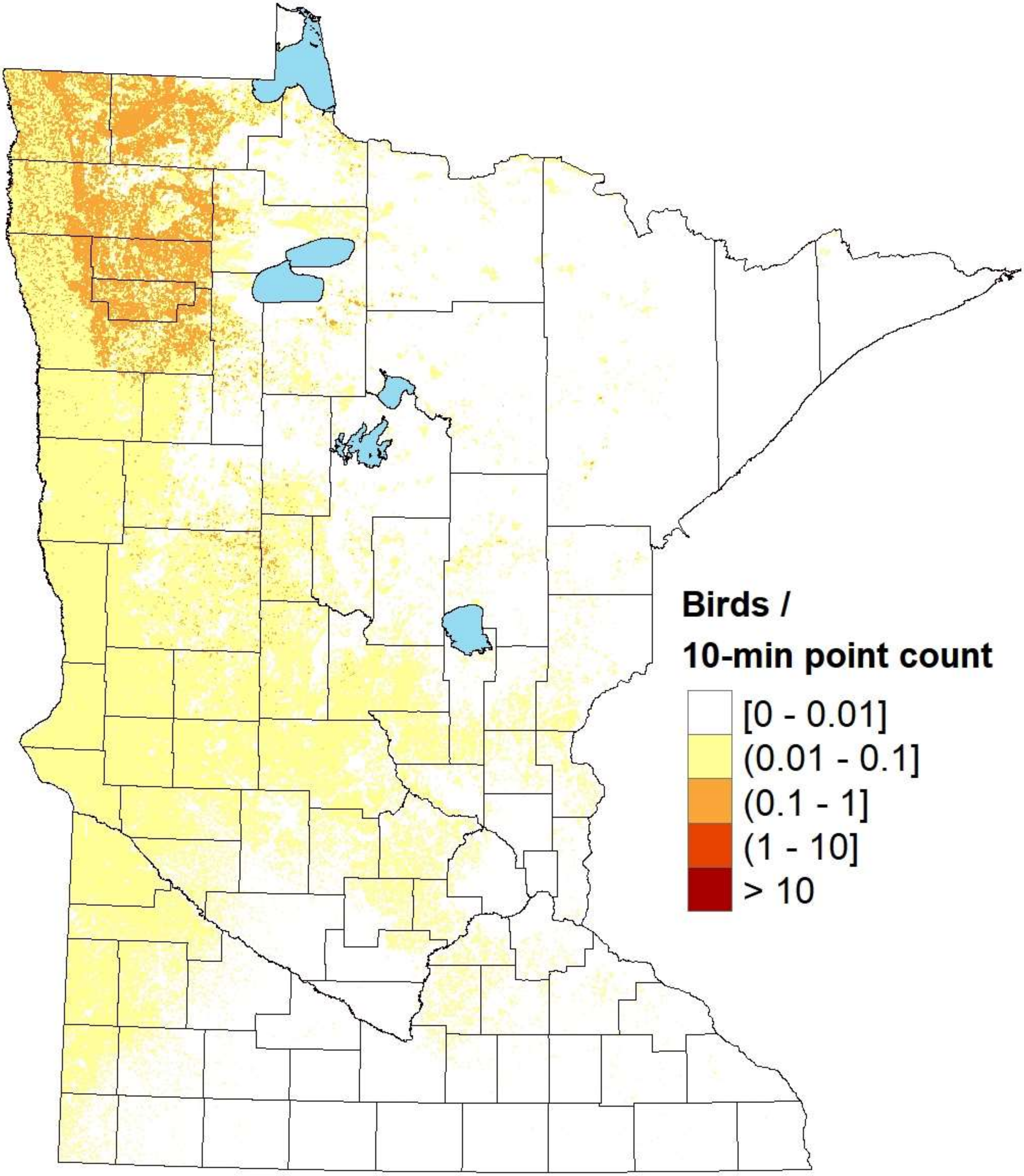

Canada Jay *Perisoreus canadensis*

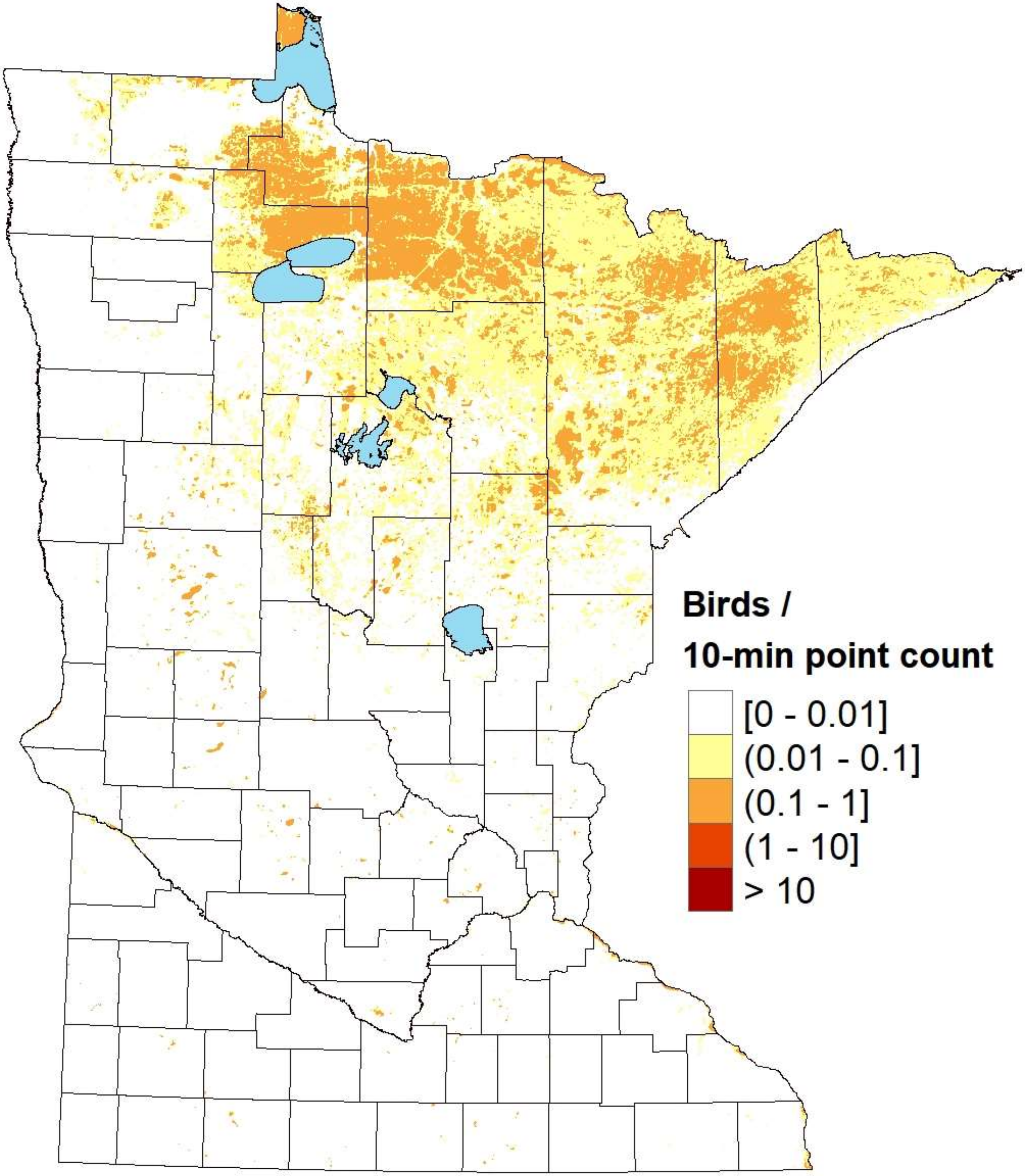

Chimney Swift *Chaetura pelagica*

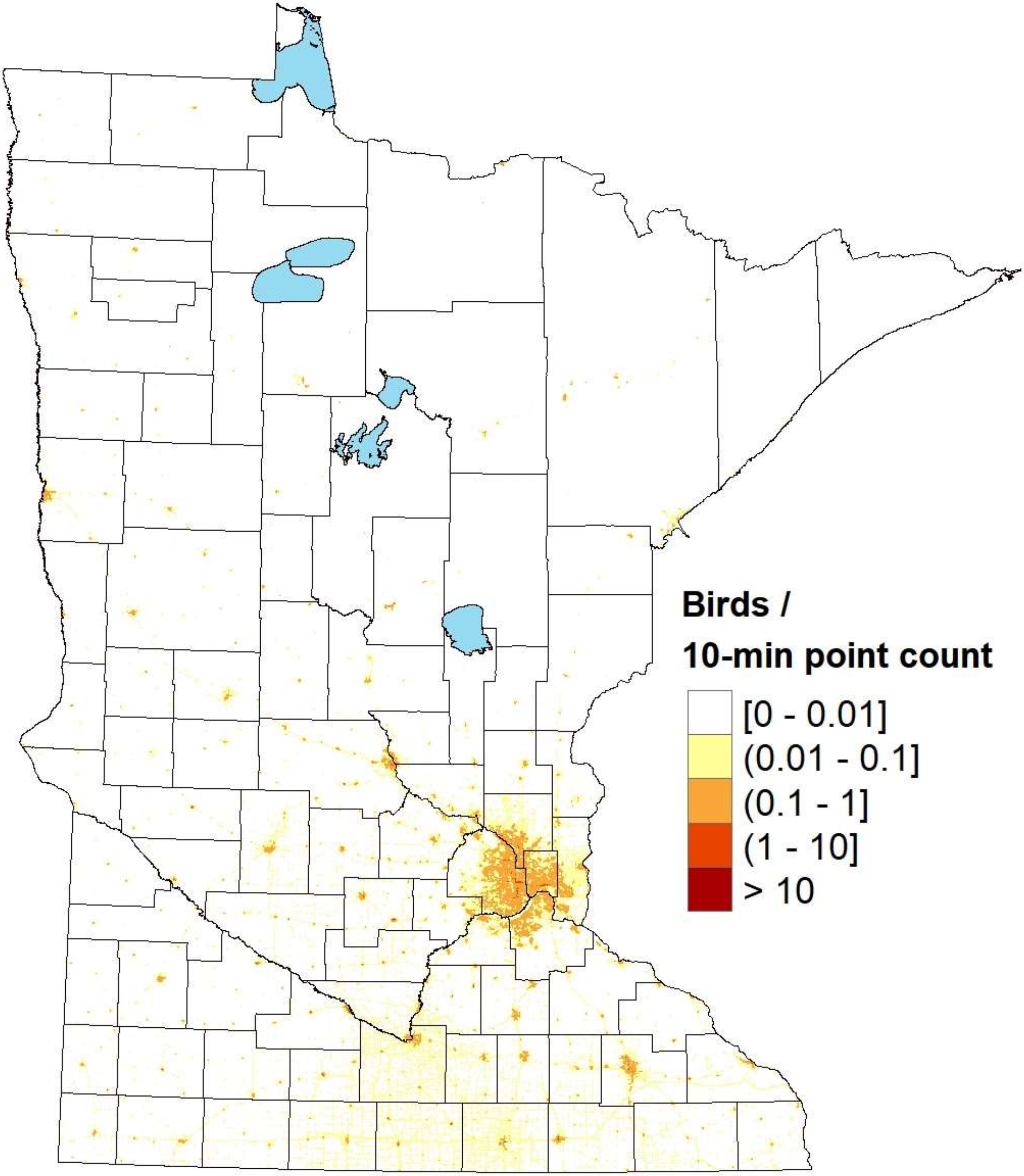

Eastern Kingbird *Tyrannus tyrannus*

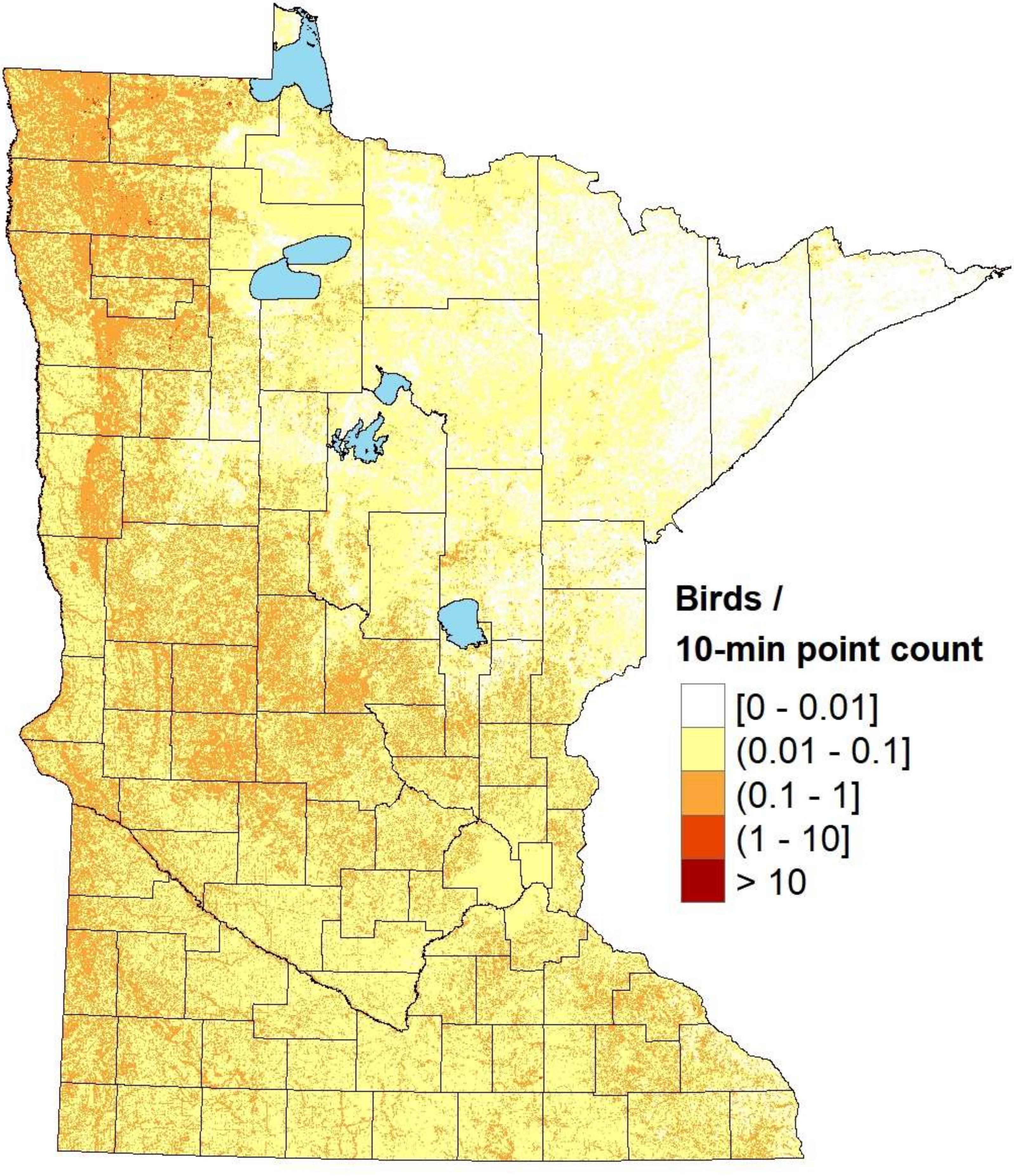

European Starling *Sturnus vulgaris*

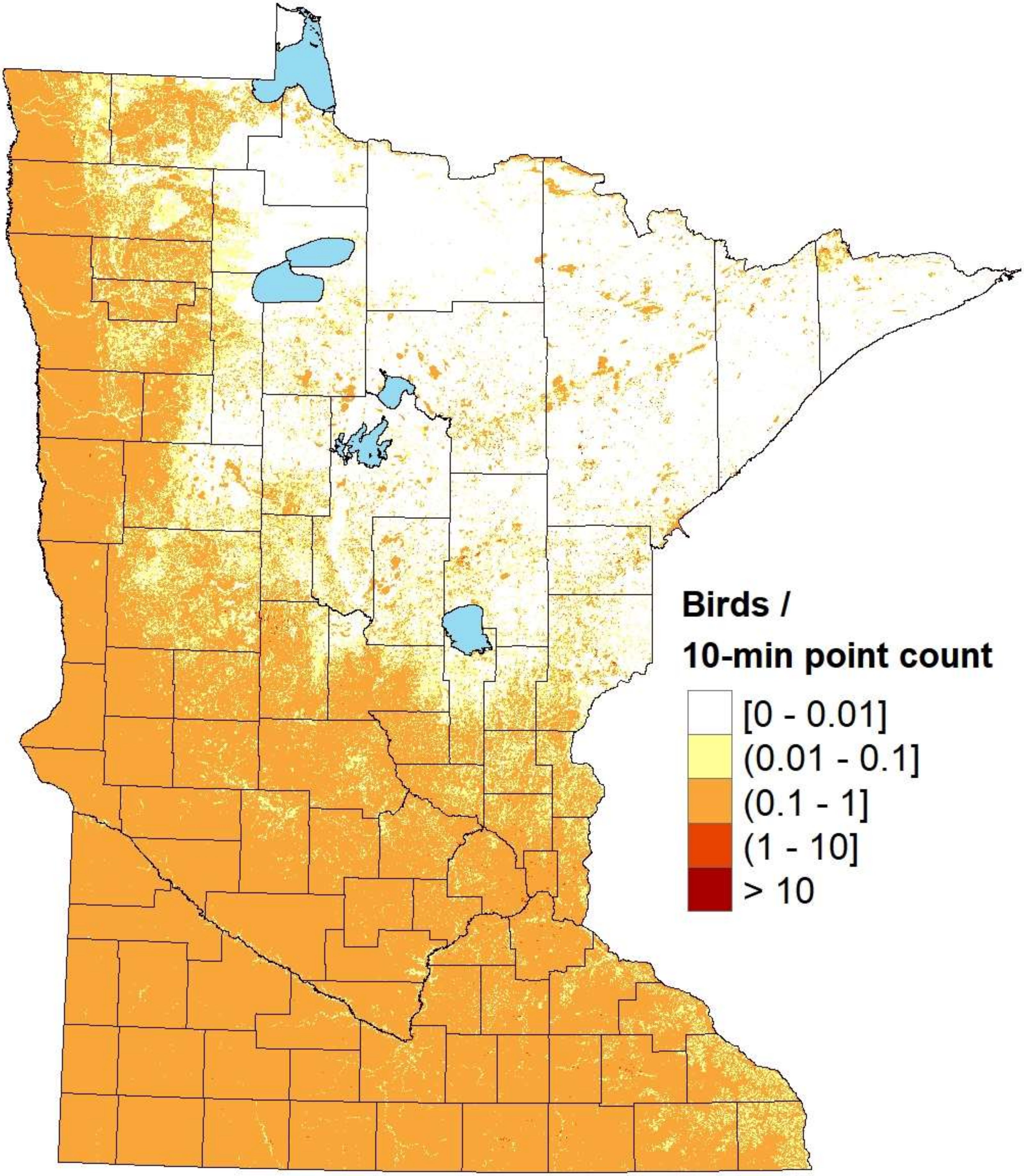

Evening Grosbeak *Coccothraustes vespertinus*

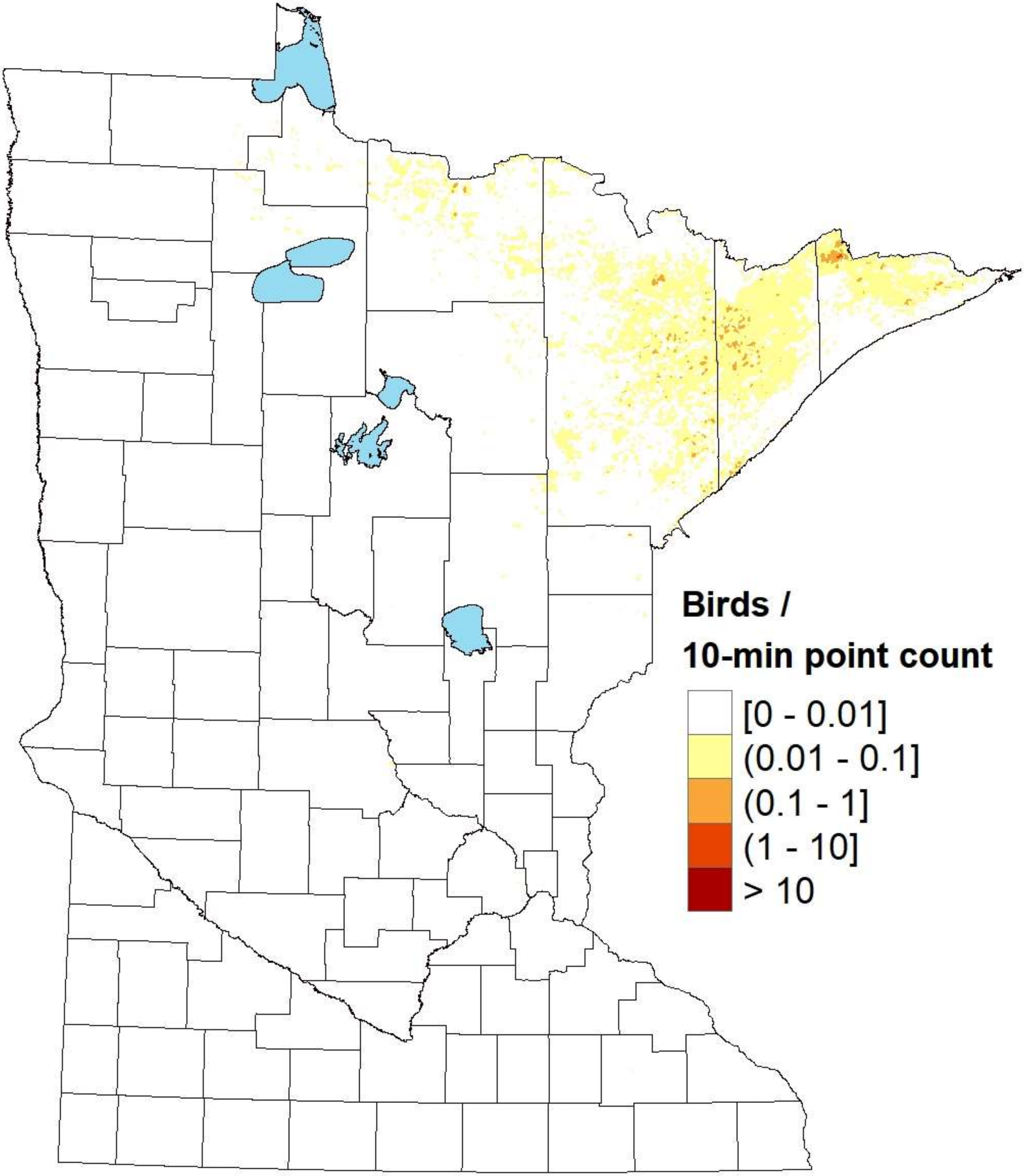

House Sparrow *Passer domesticus*

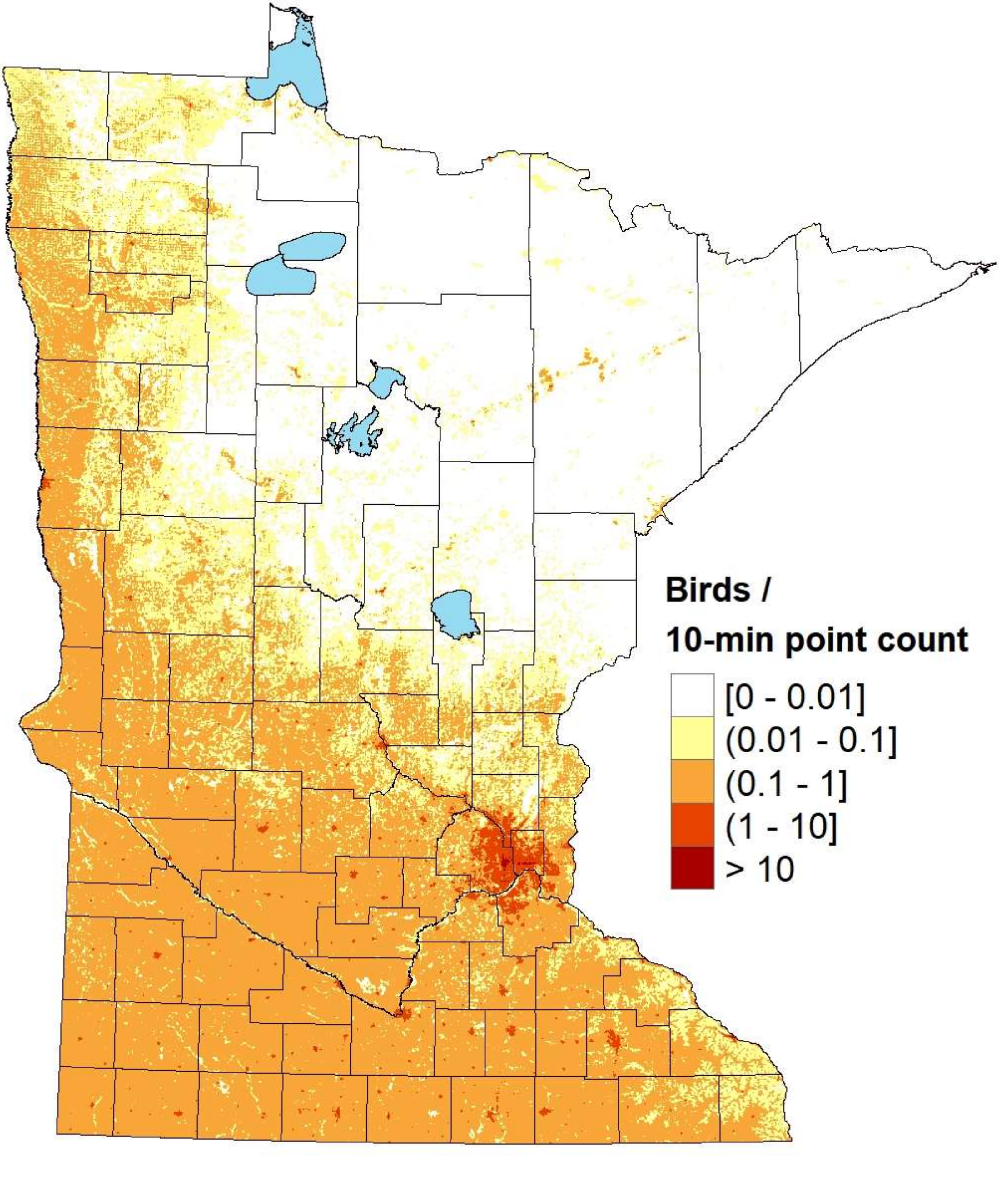

Killdeer *Charadrius vociferus*

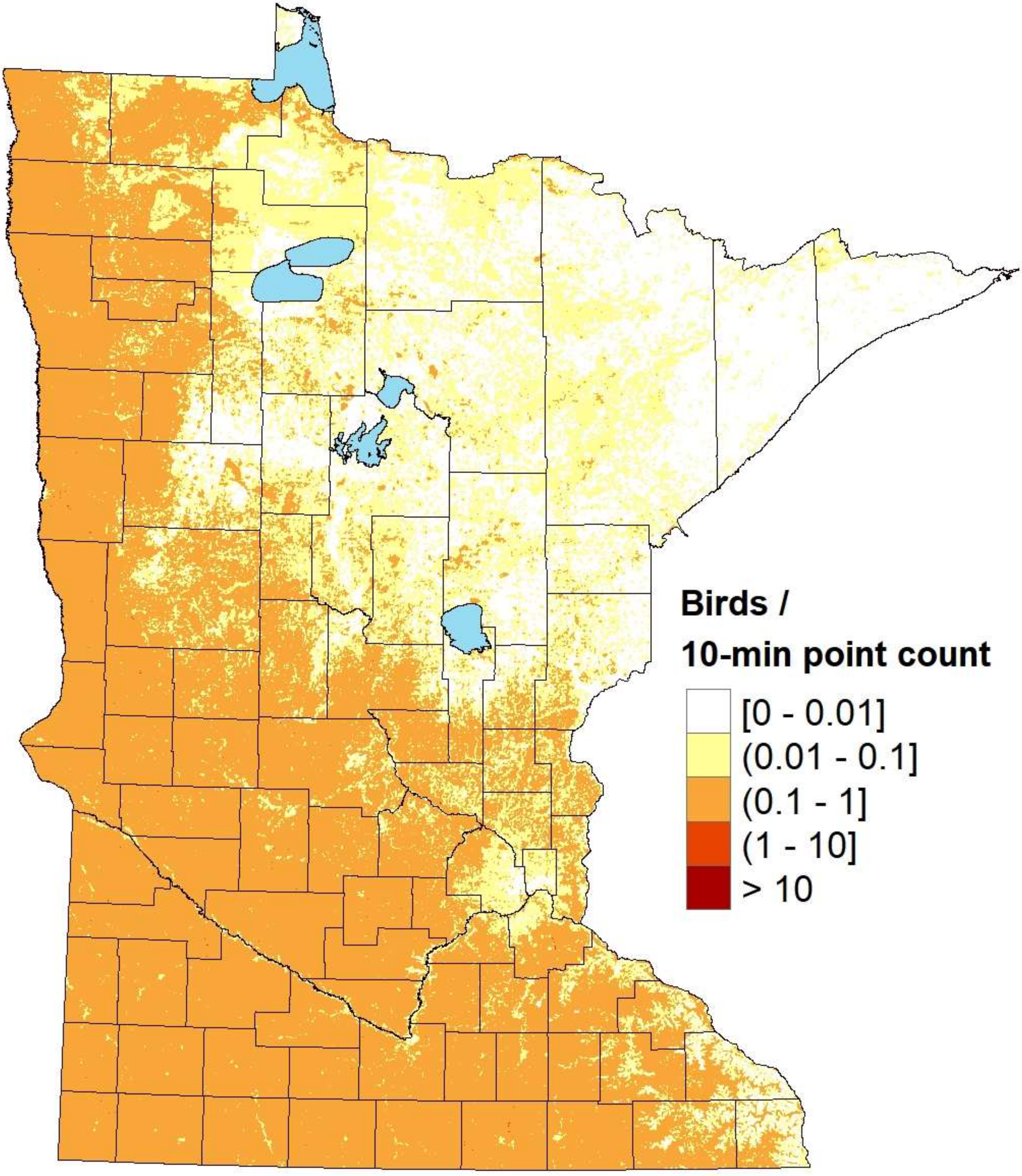

Mallard *Anas platyrhynchos*

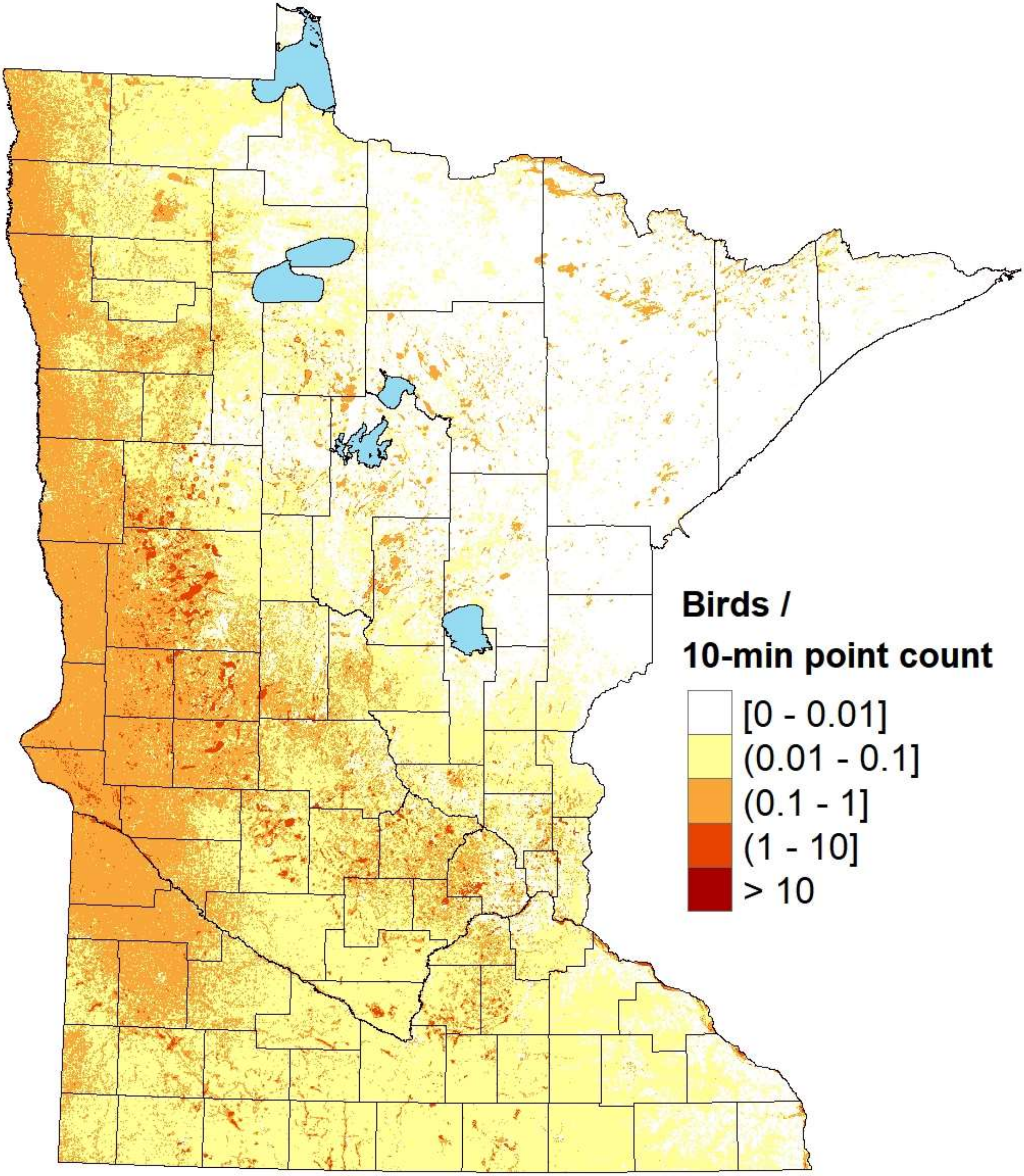

Mourning Dove *Zenaida macroura*

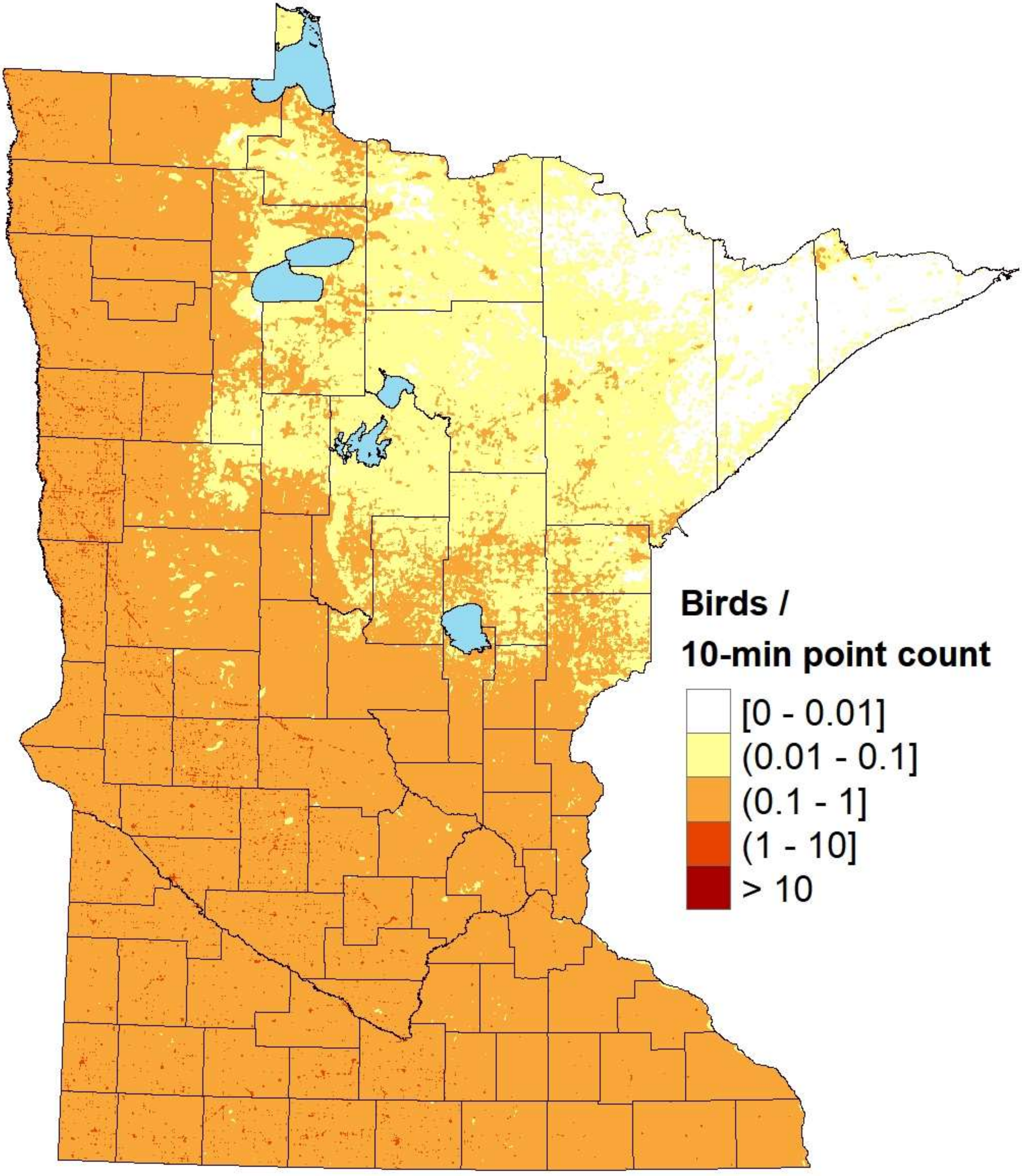

Northern Harrier *Circus hudsonius*

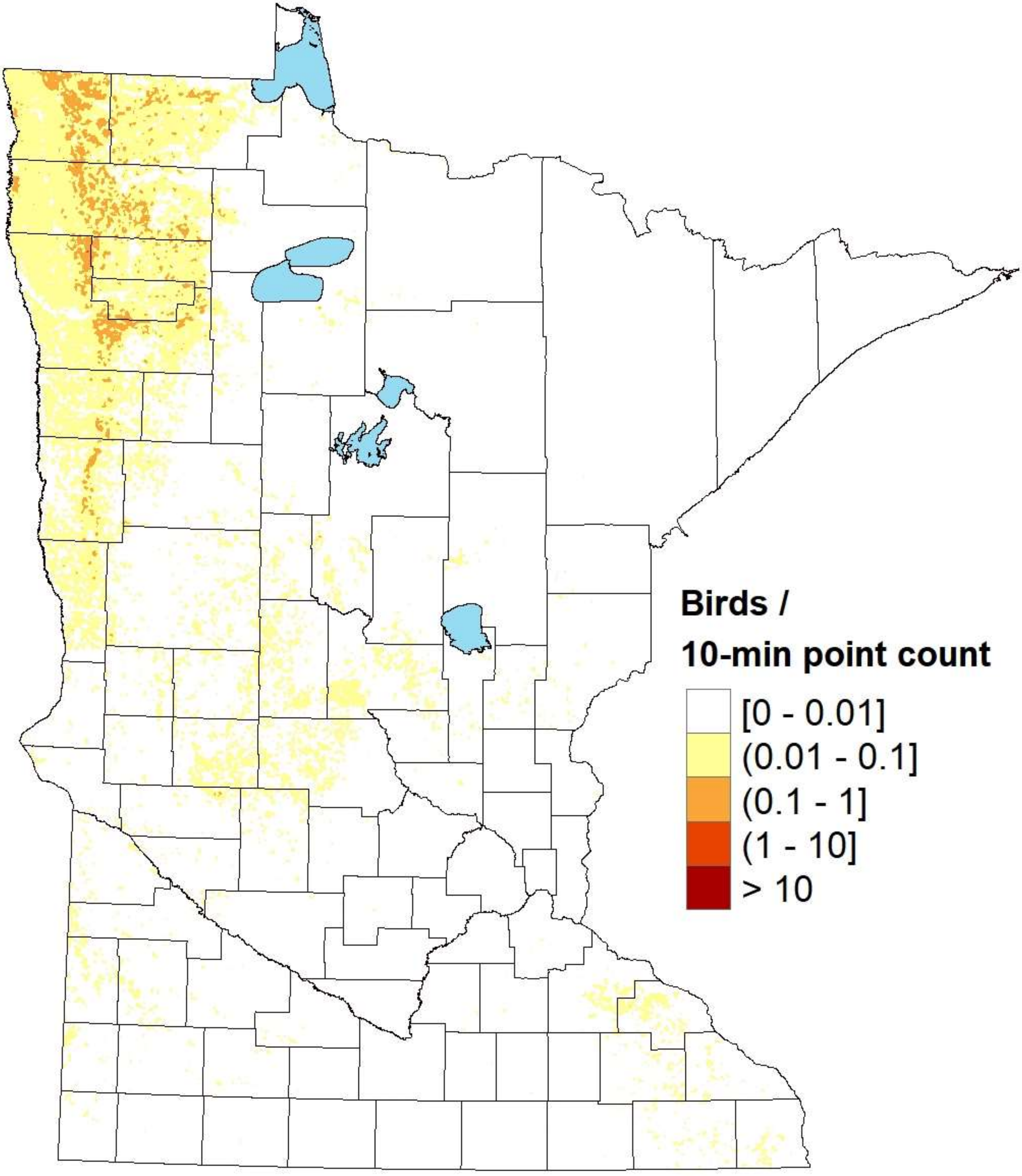

Pied-billed Grebe *Podilymbus podiceps*

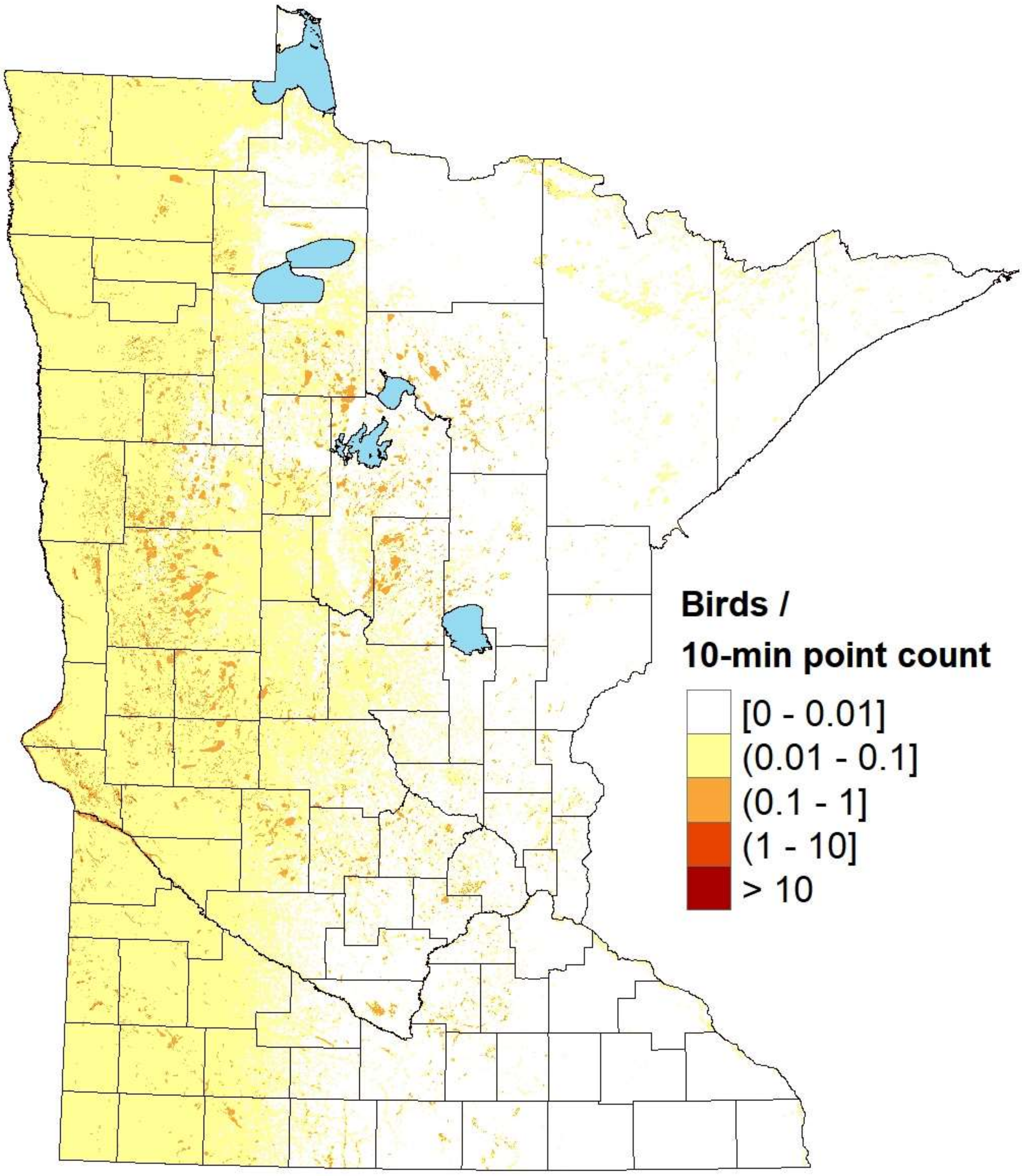

Pine Siskin *Spinus pinus*

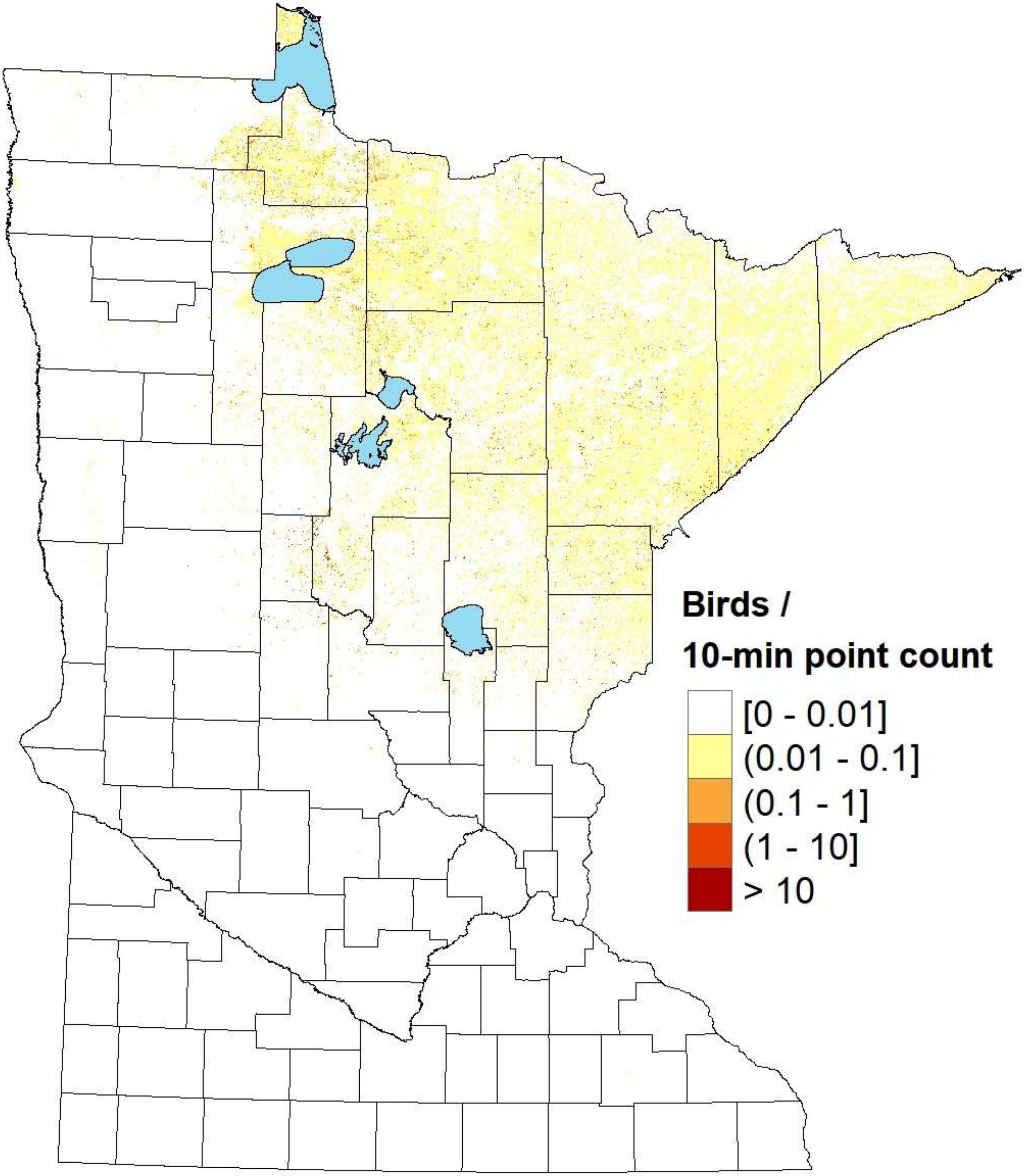

Red-bellied Woodpecker *Melanerpes carolinus*

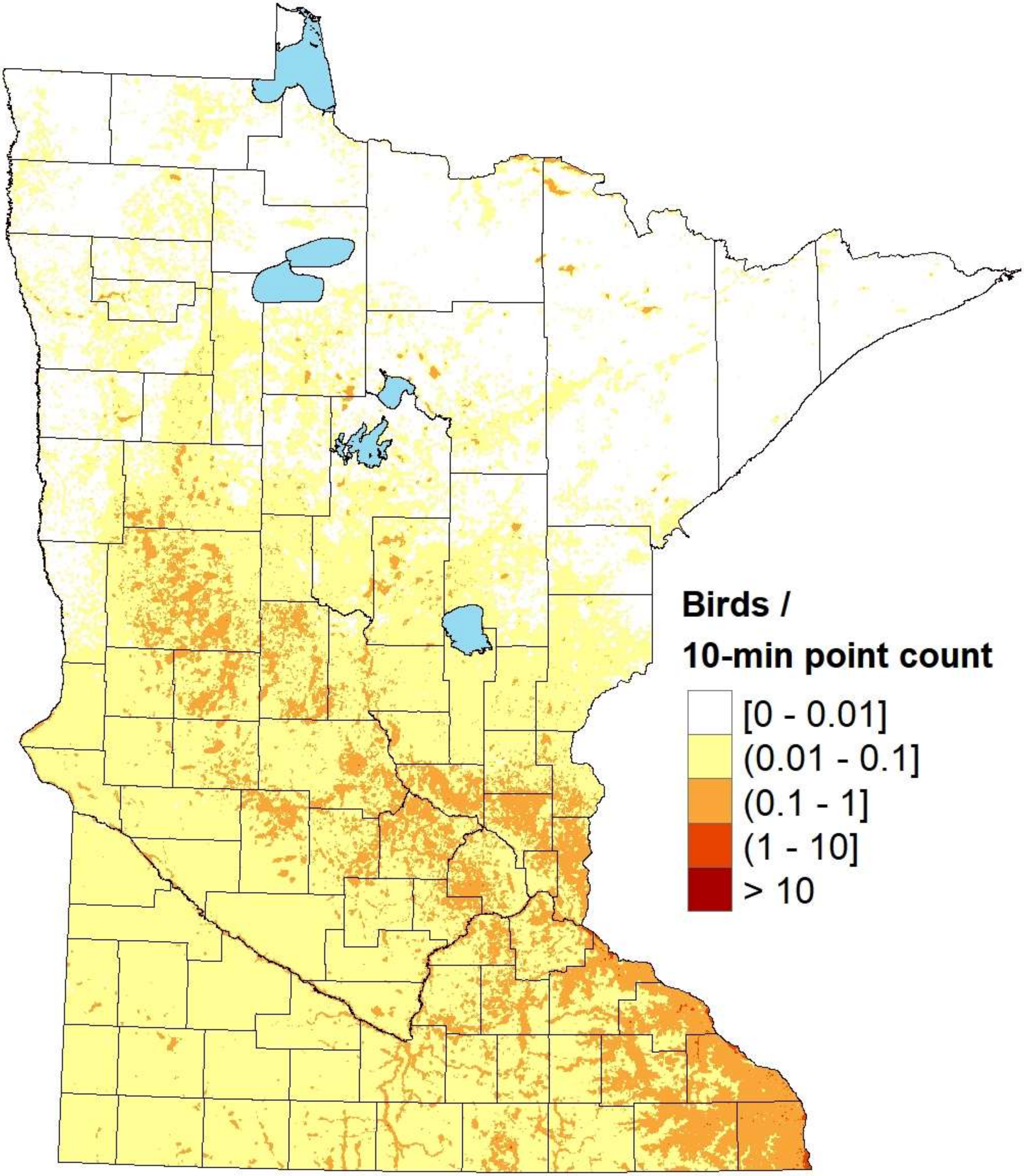

Red-breasted Nuthatch *Sitta canadensis*

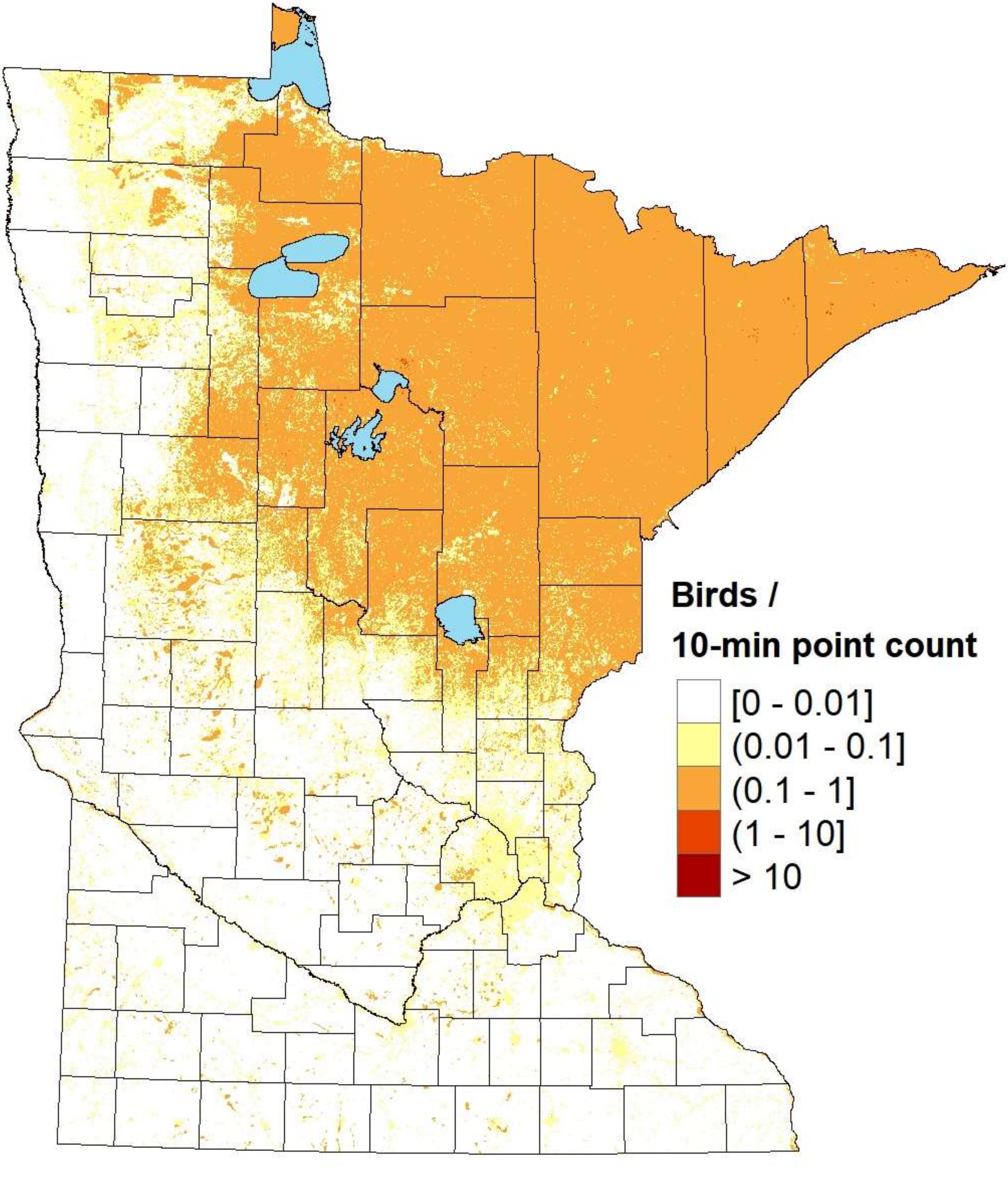

Red-tailed Hawk *Buteo jamaicensis*

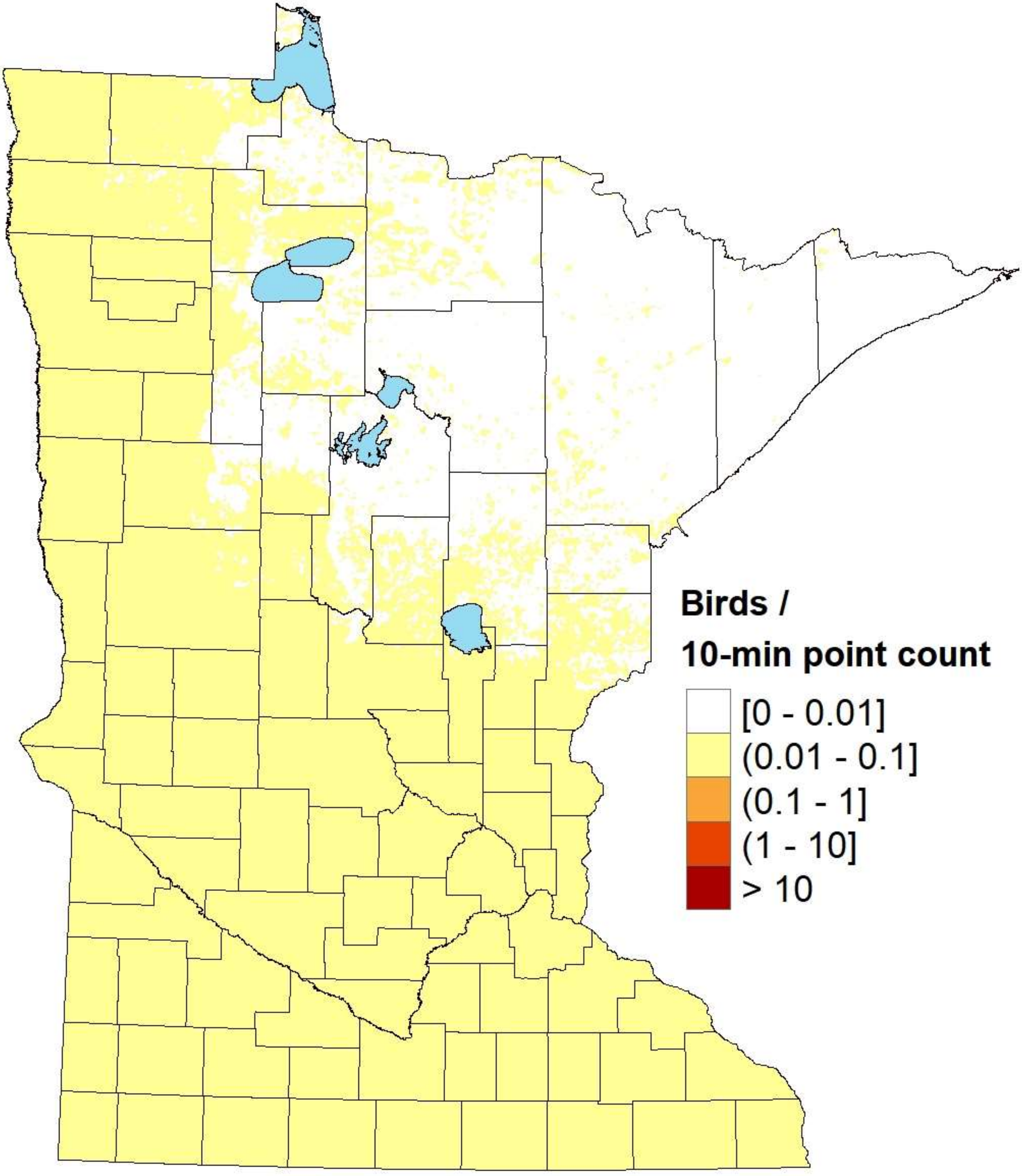

Ring-necked Pheasant *Phasianus colchicus*

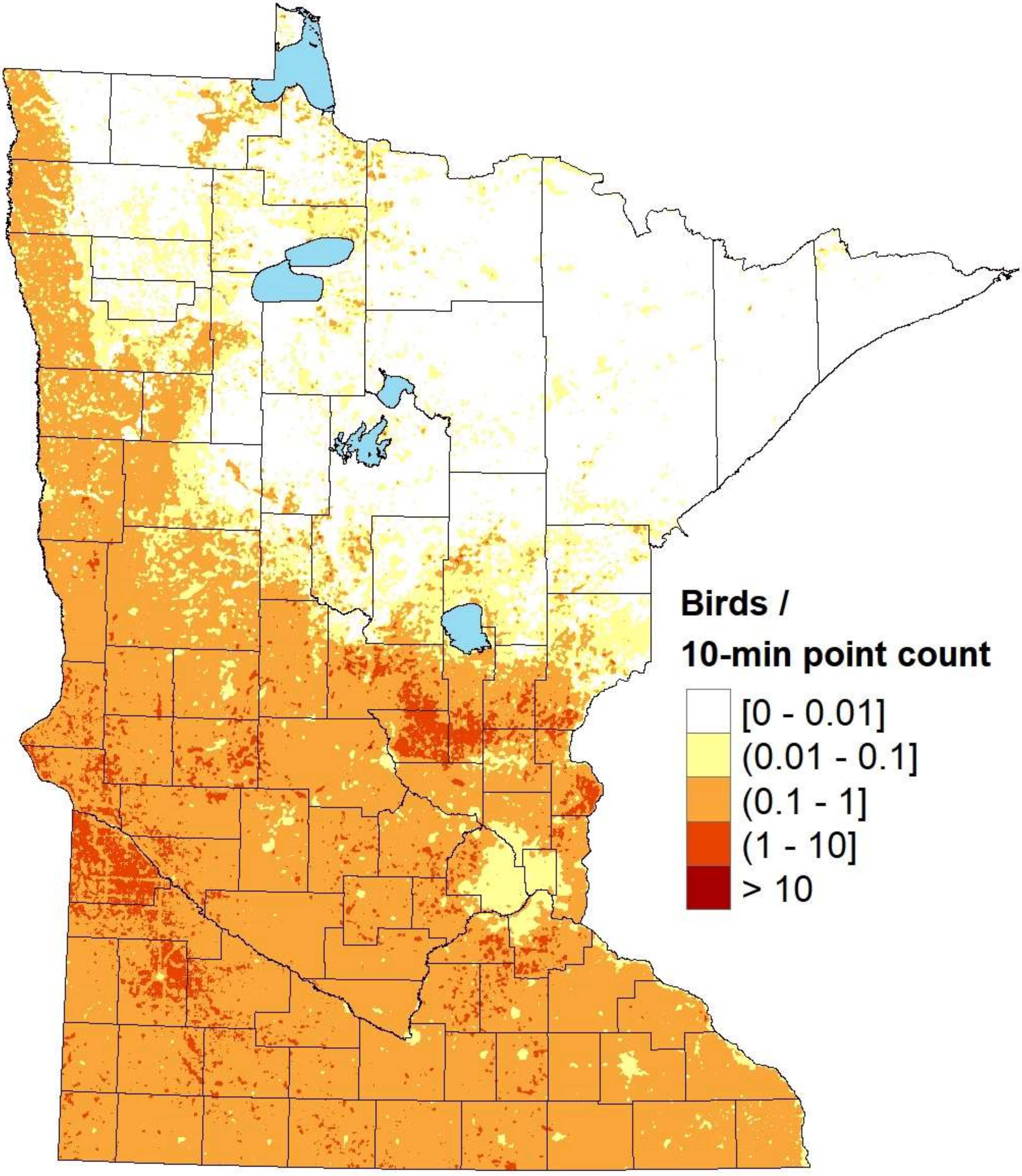

Ruby-throated Hummingbird *Archilochus colubris*

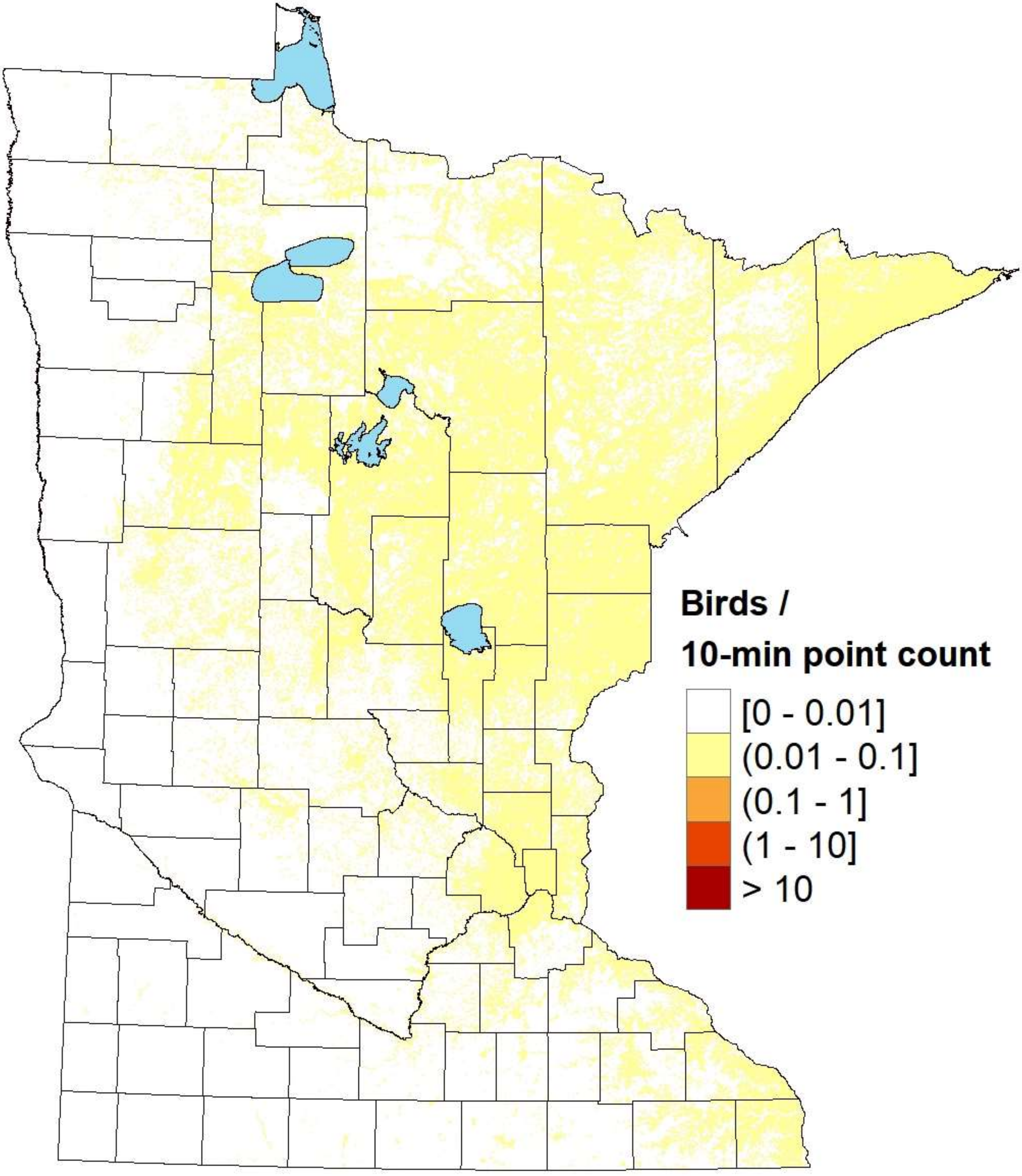

Sandhill Crane *Antigone canadensis*

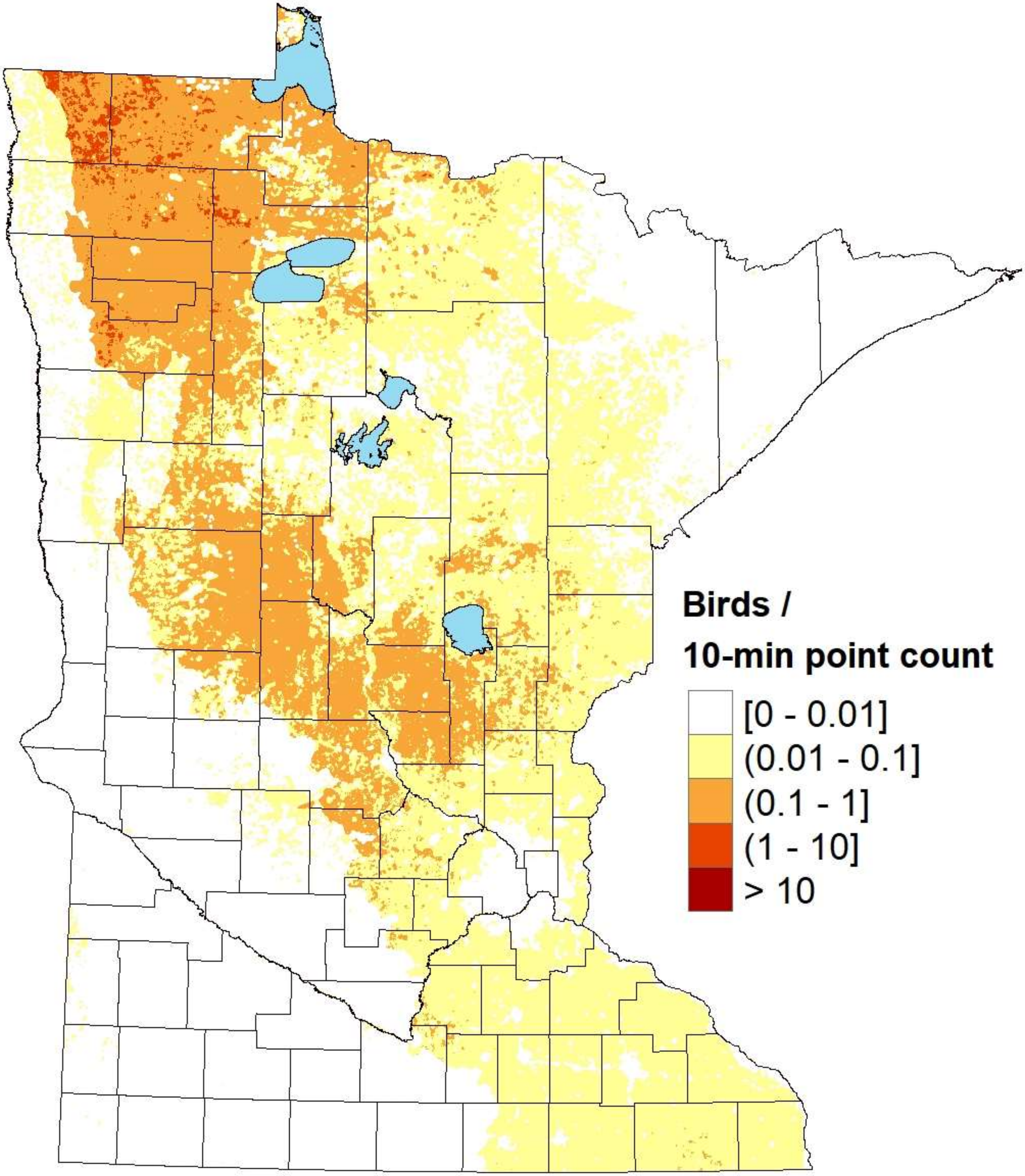

Sora *Porzana carolina*

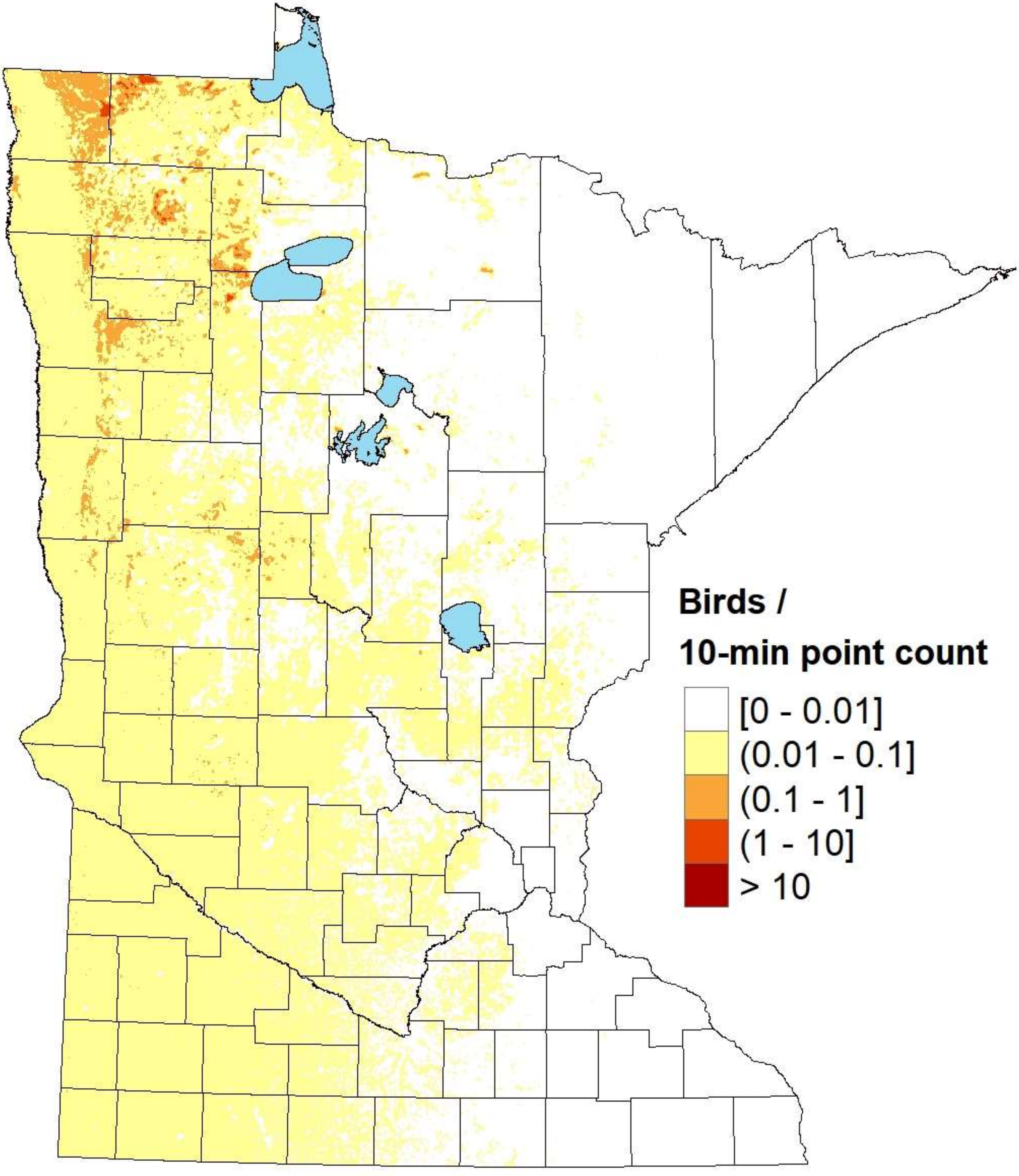

Tree Swallow *Tachycineta bicolor*

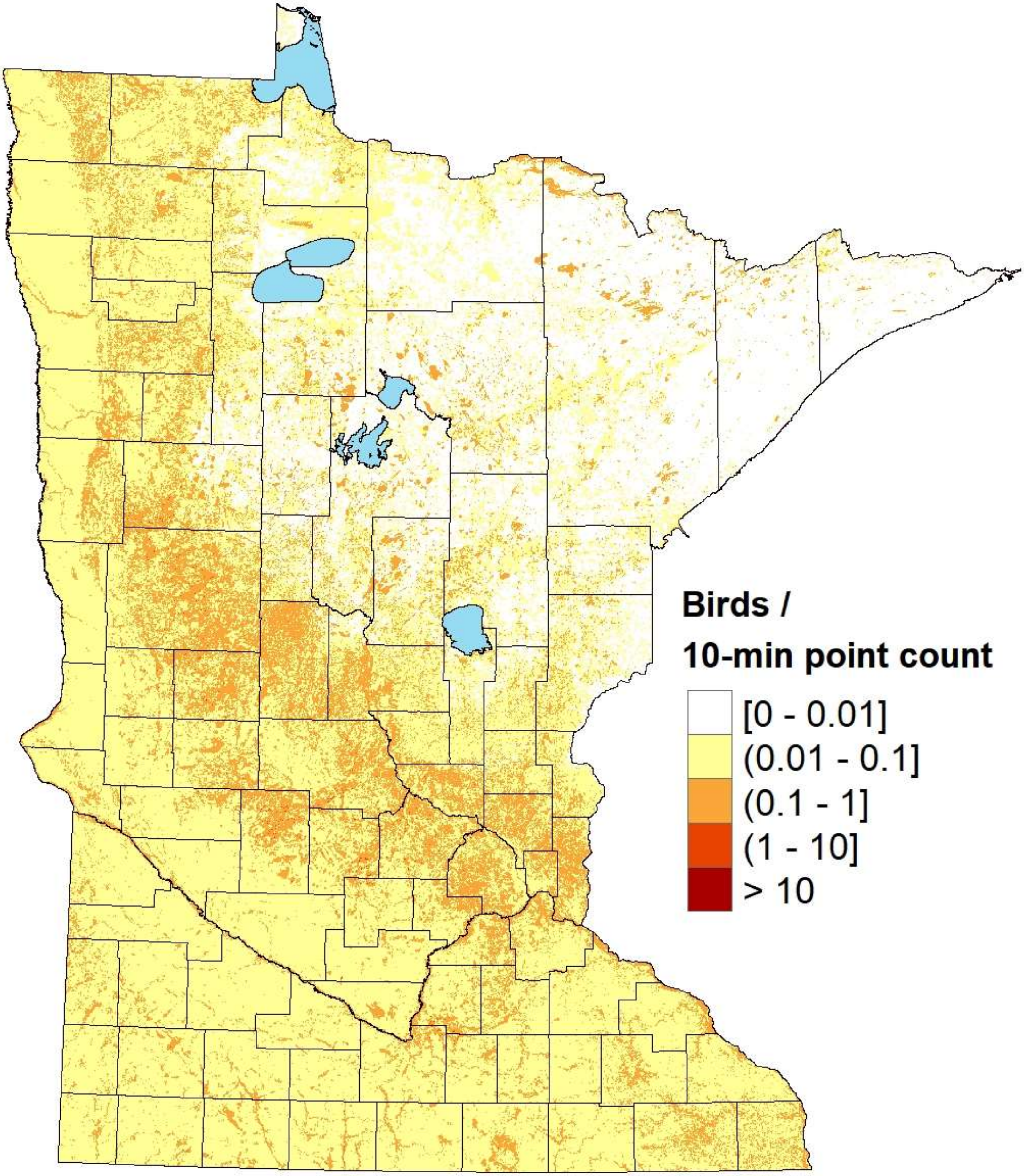

Upland Sandpiper *Bartramia longicauda*

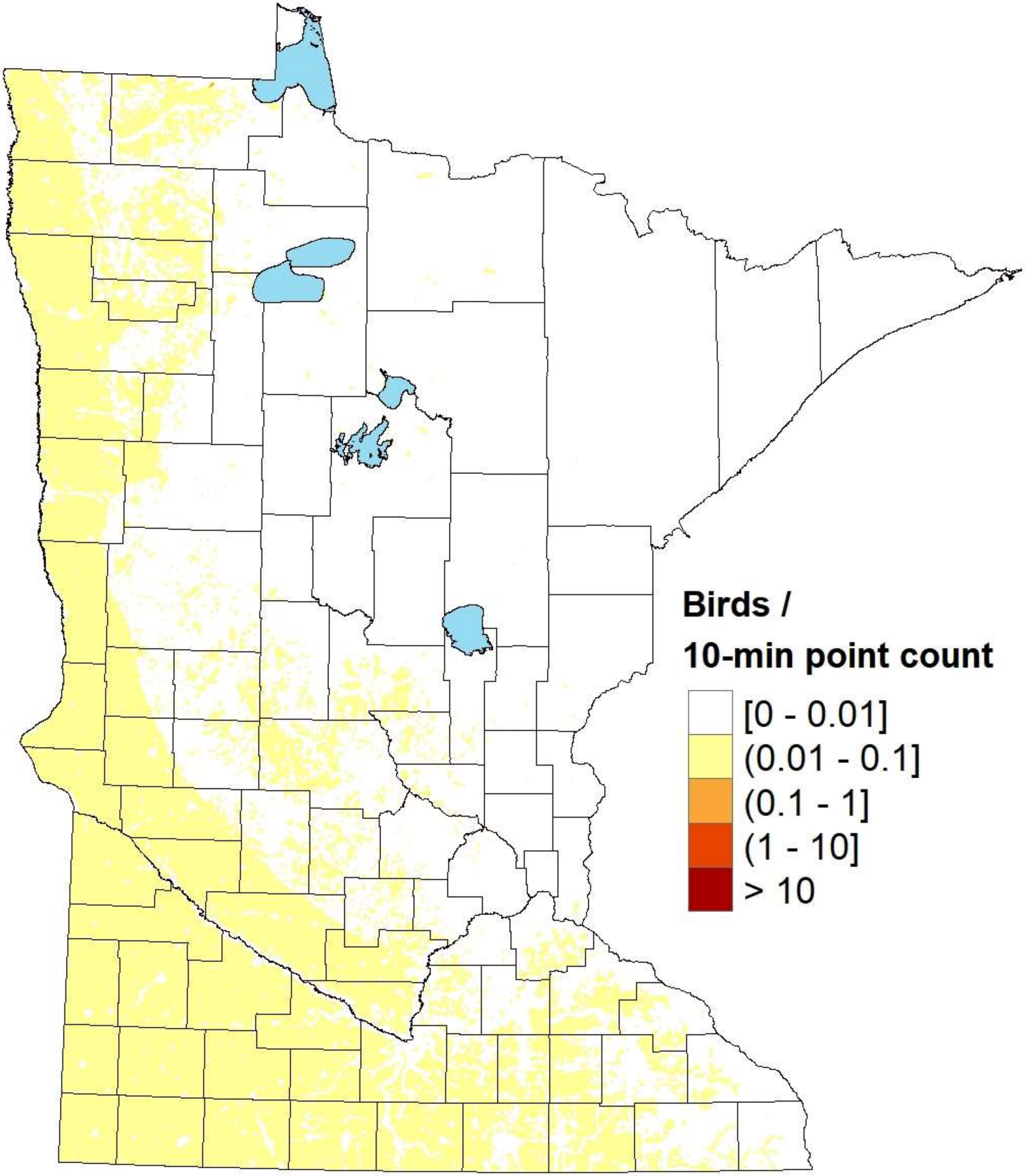

Willow Flycatcher *Empidonax traillii*

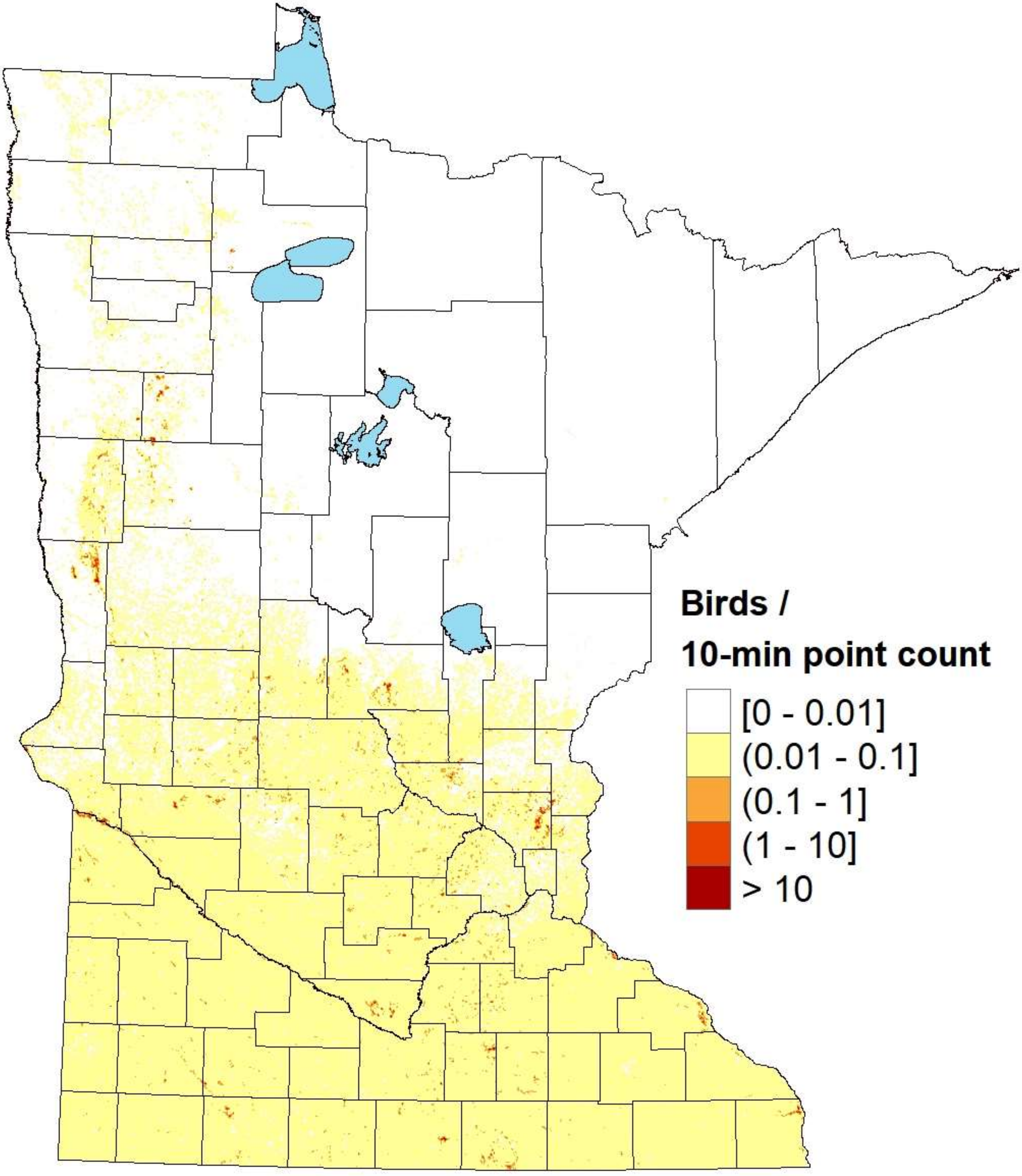

Wood Duck *Aix sponsa*

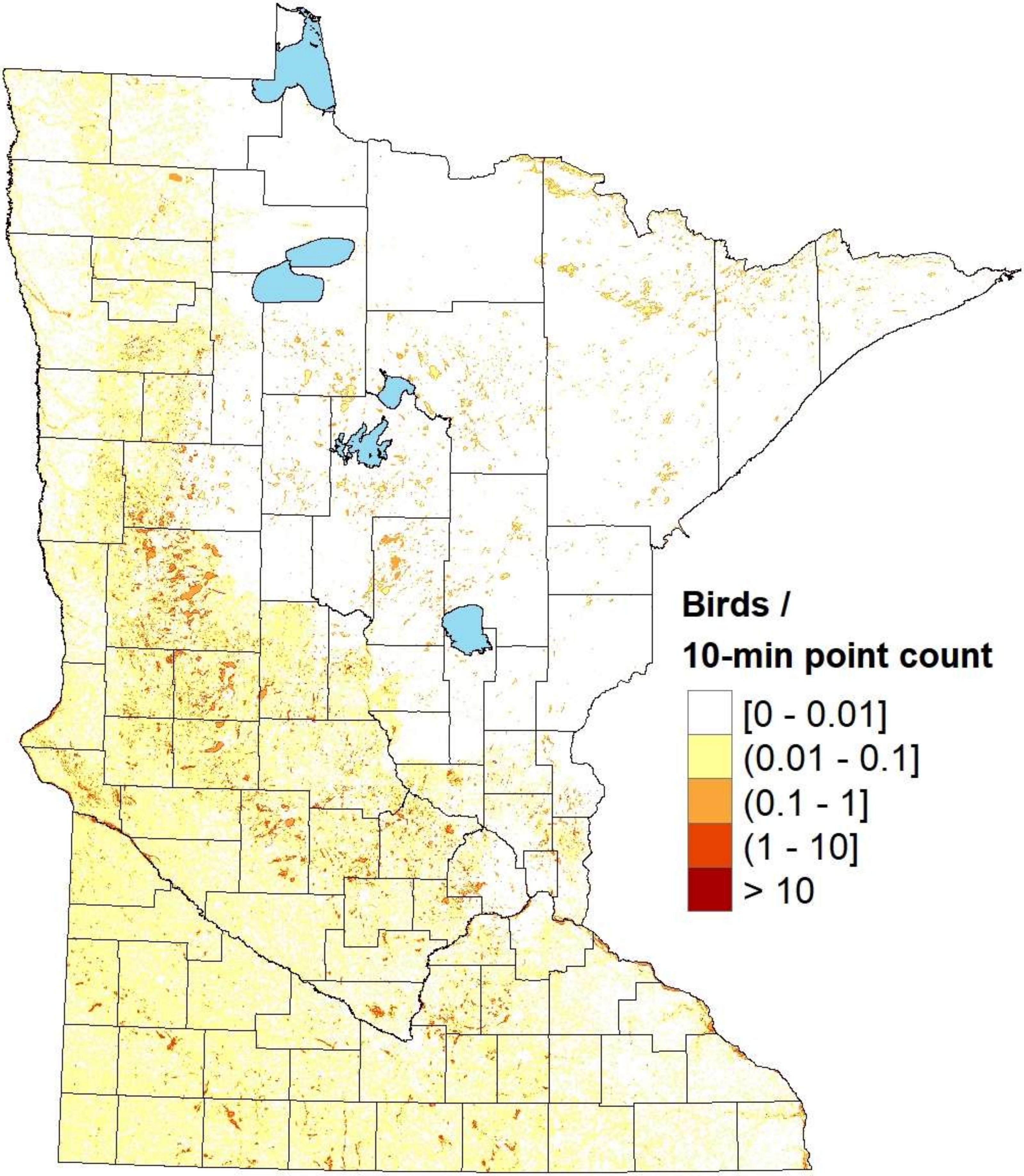

Yellow-bellied Sapsucker *Sphyrapicus varius*

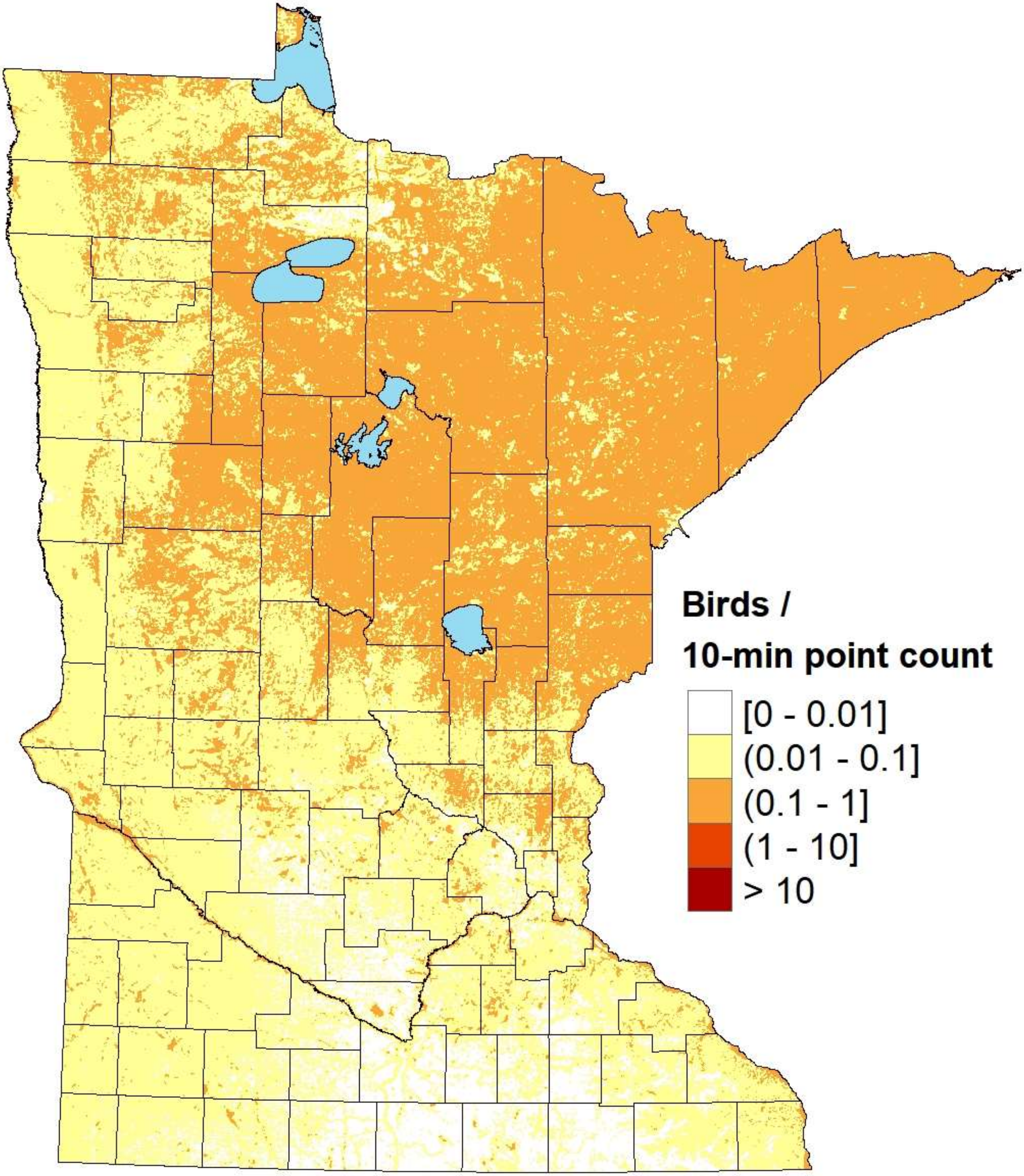

GLM with QPAD offset (73 species)

Alder Flycatcher *Empidonax alnorum*

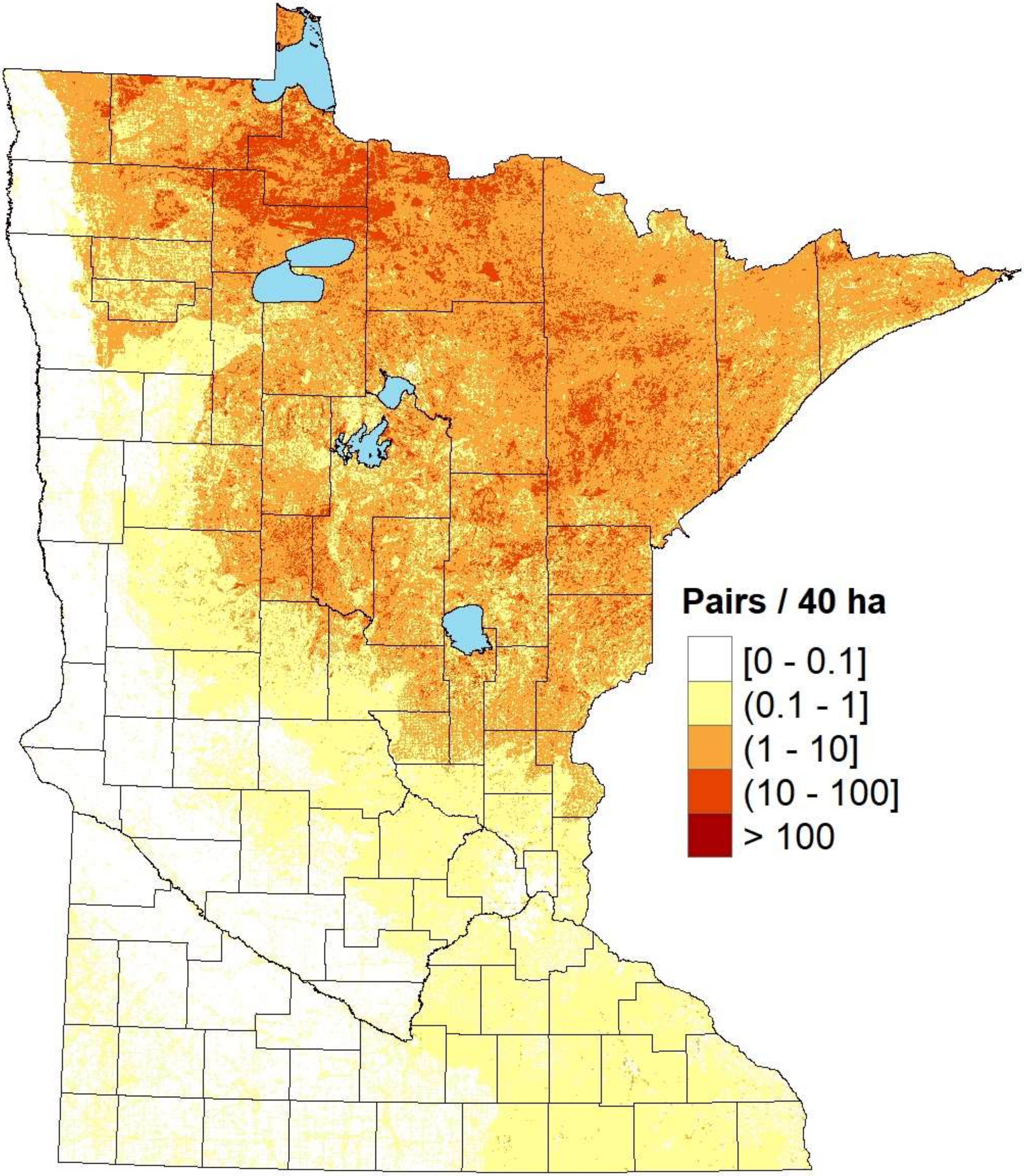

American Goldfinch *Spinus tristis*

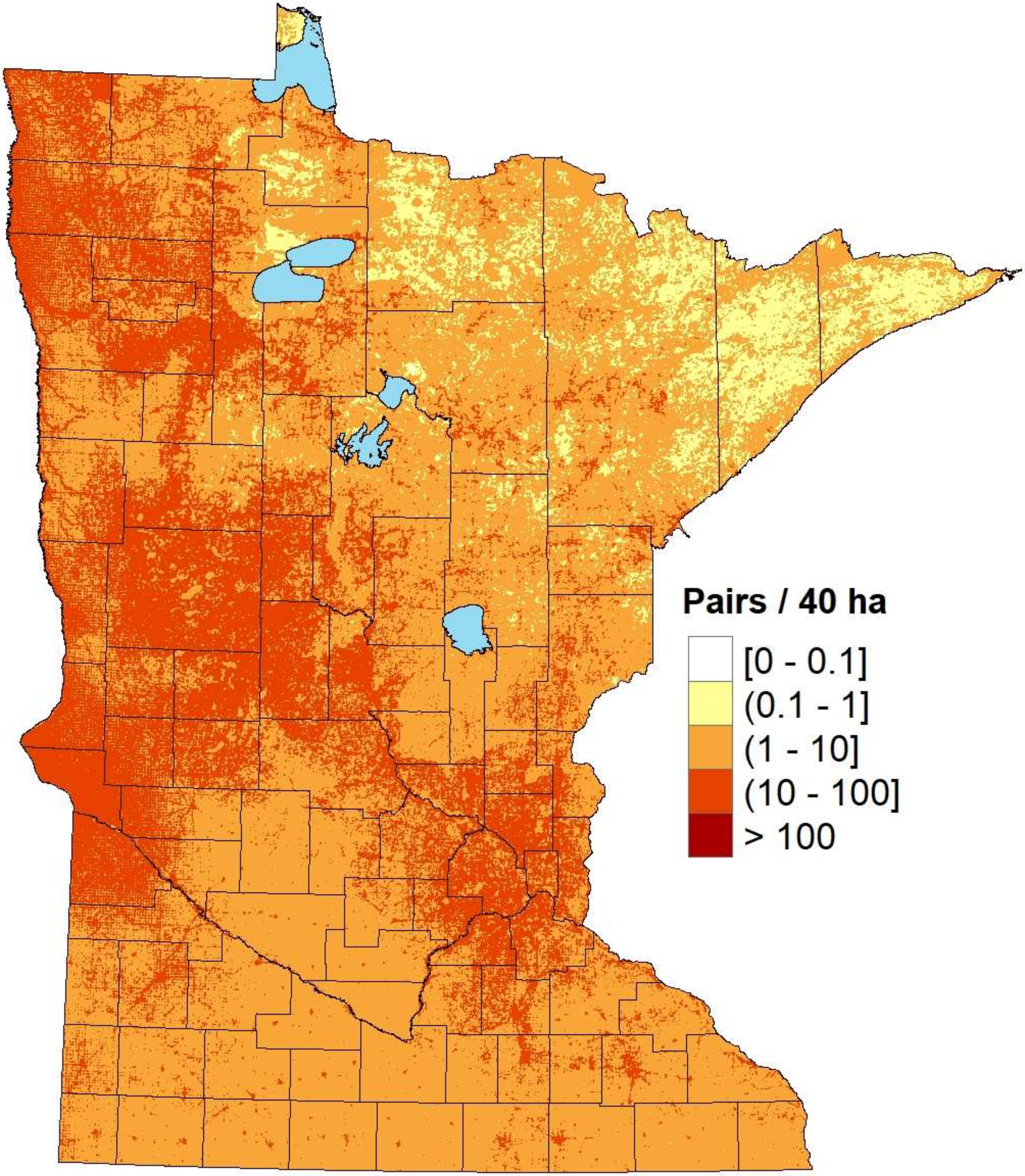

American Redstart *Setophaga ruticilla*

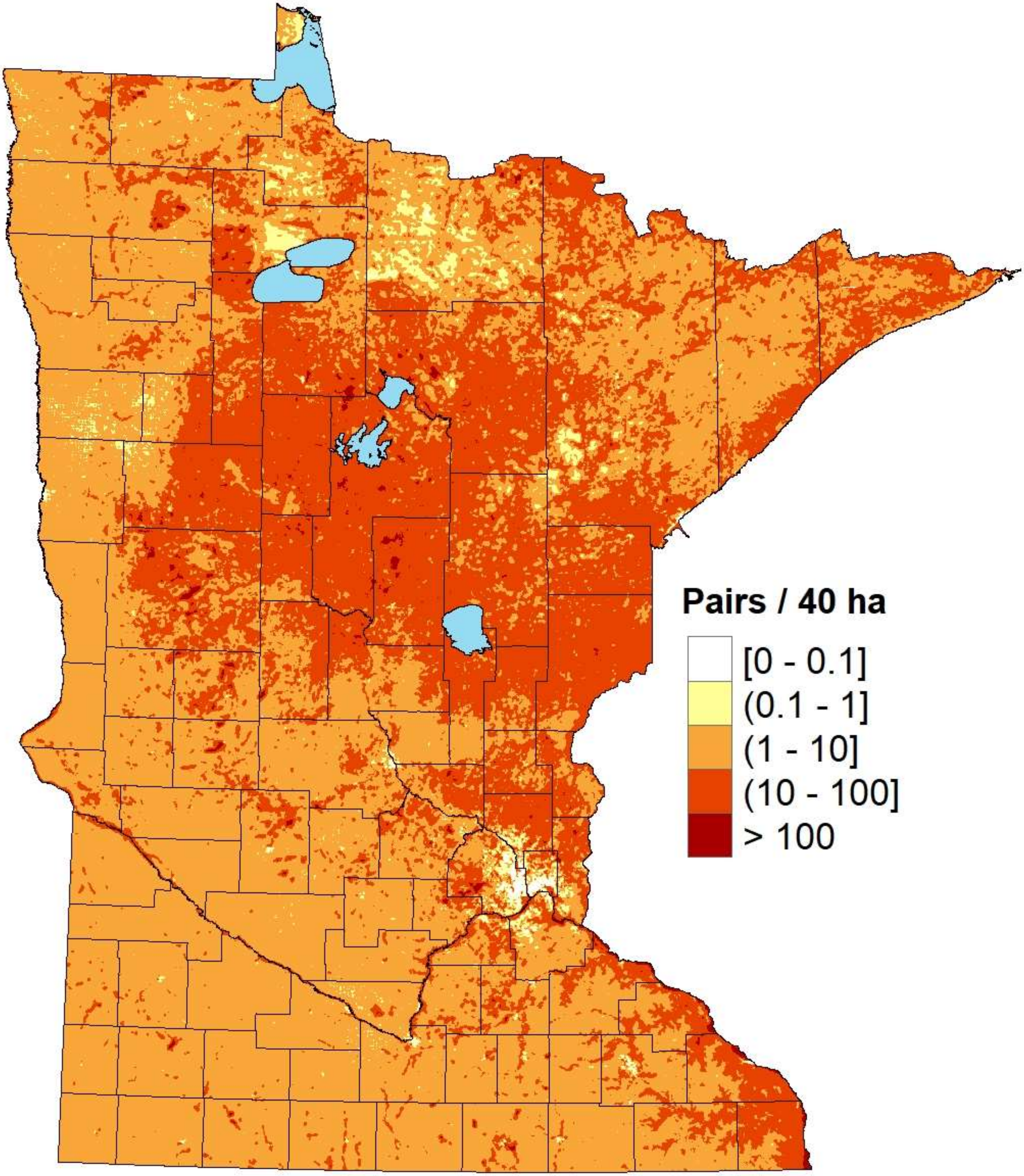

Baltimore Oriole *Icterus galbula*

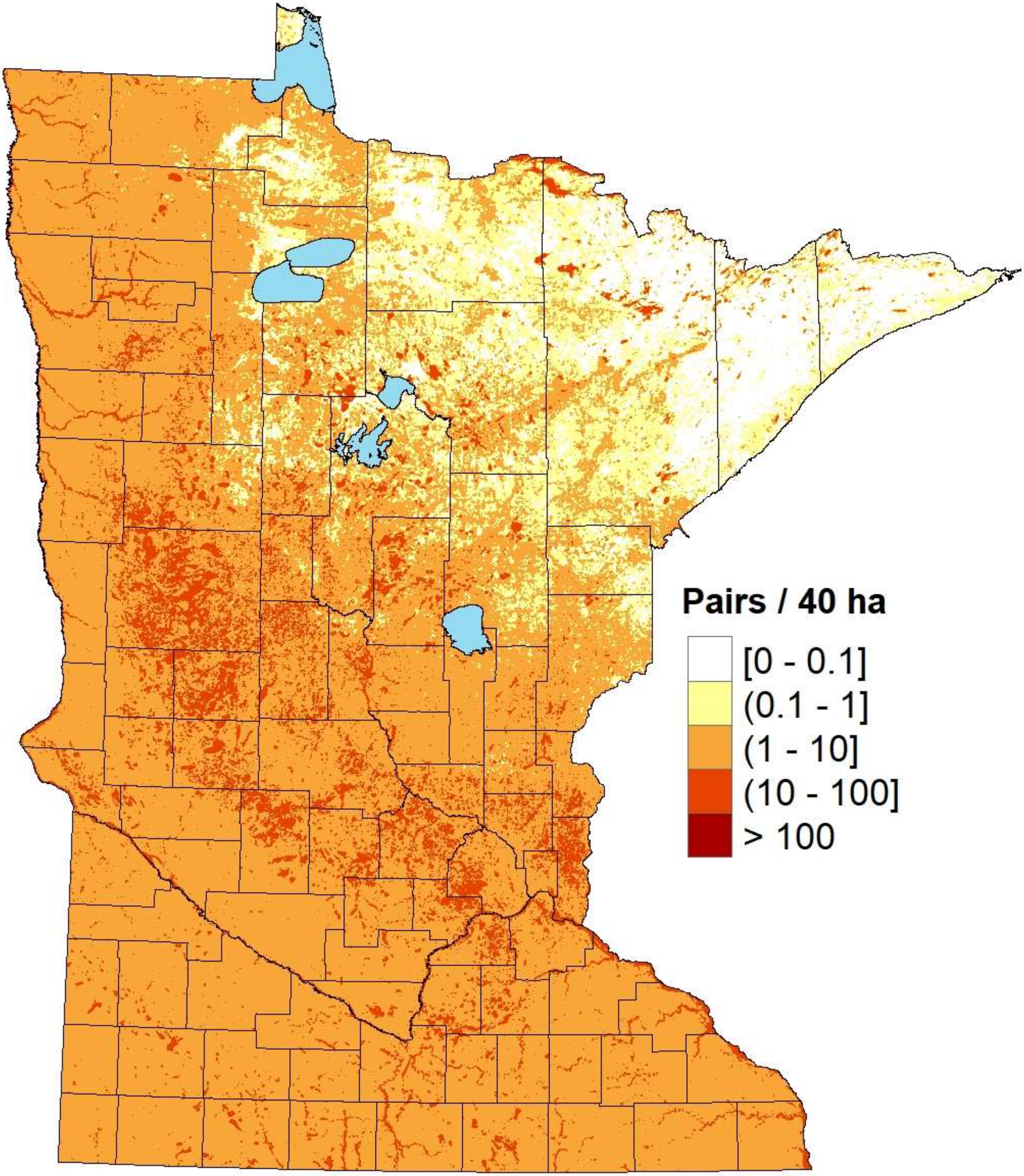

Black-and-white Warbler *Mniotilta varia*

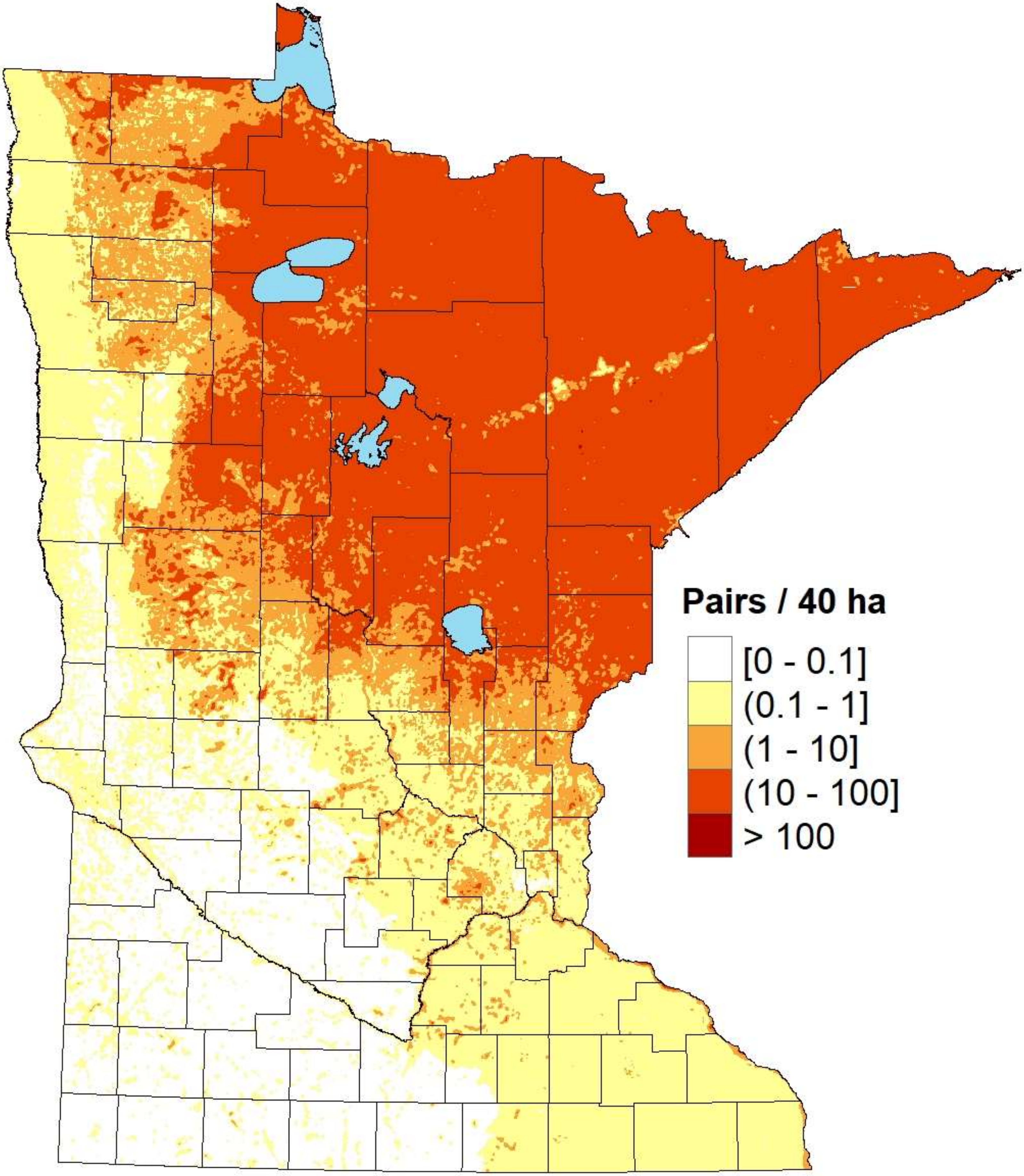

Black-billed Cuckoo *Coccyzus erythropthalmus*

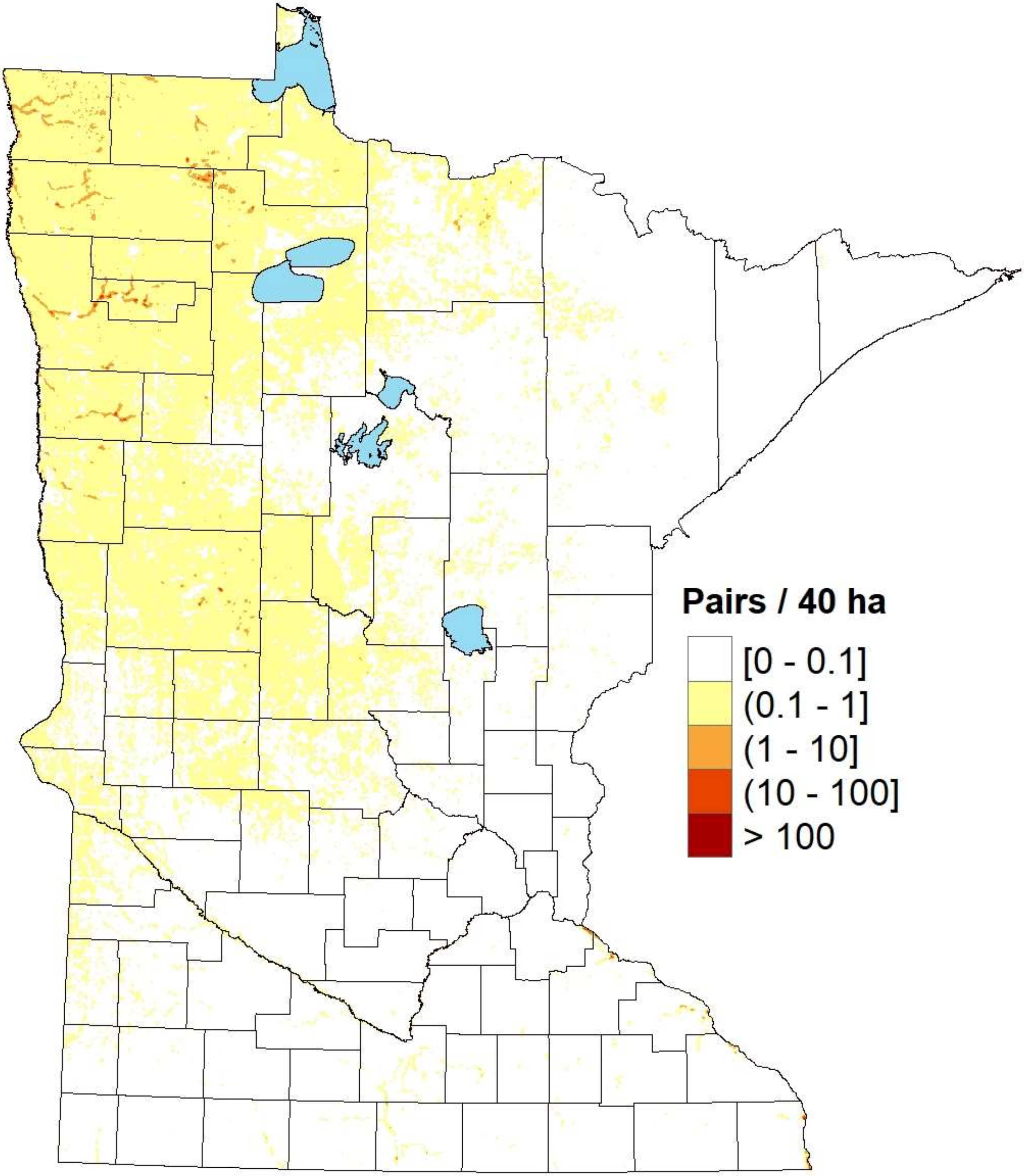

Blackburnian Warbler *Setophaga fusca*

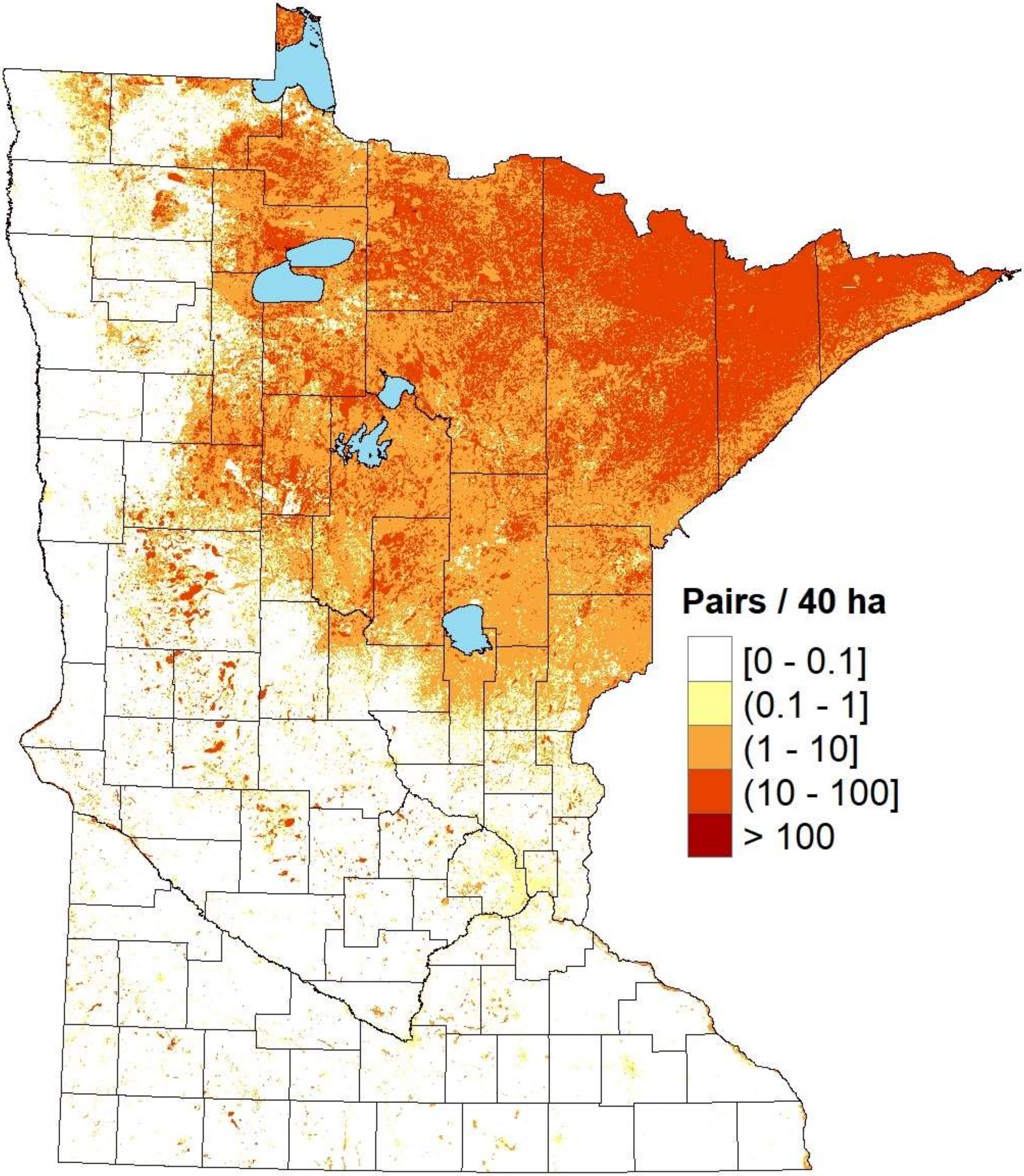

Black-throated Blue Warbler *Setophaga caerulescens*

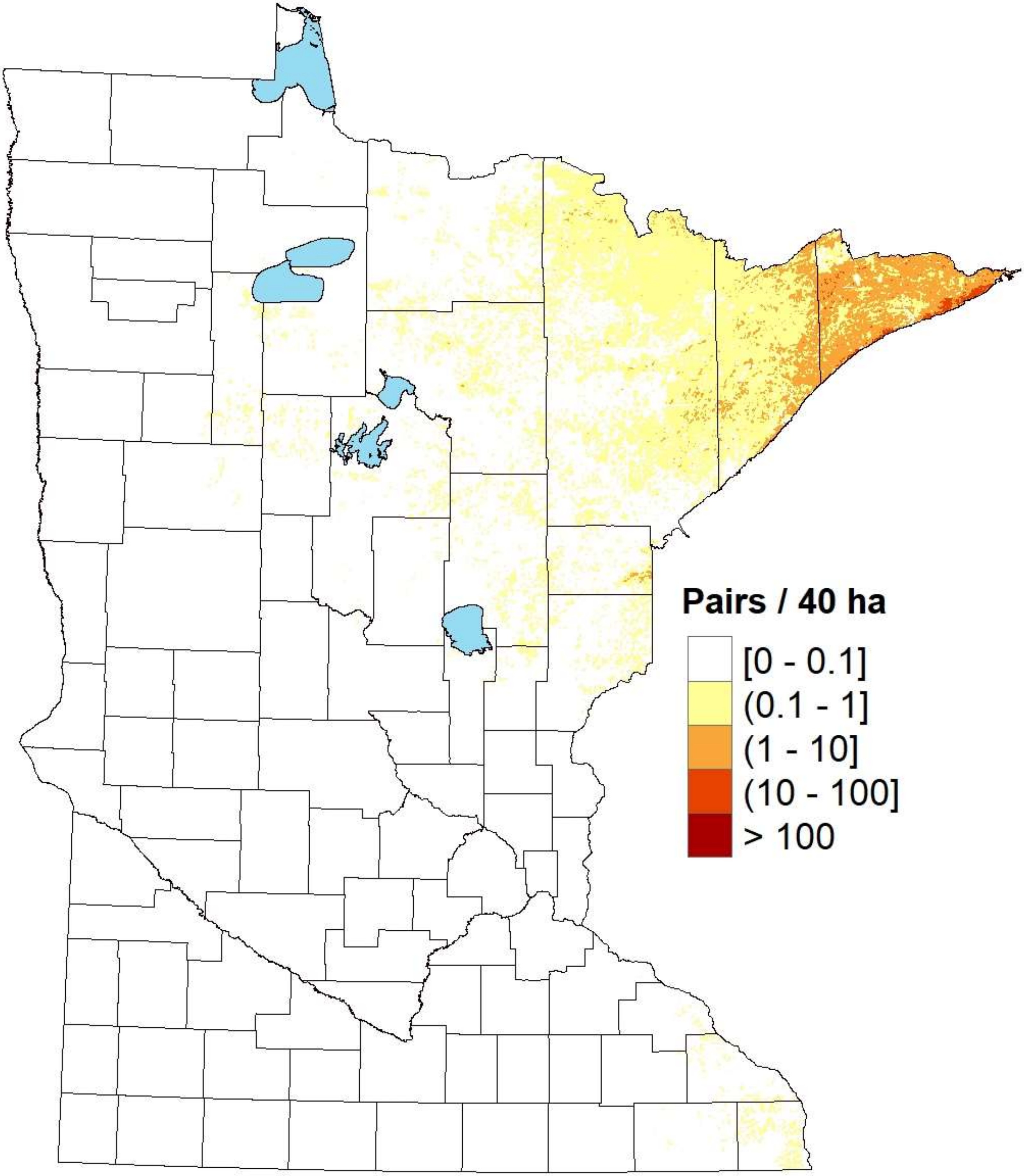

Black-throated Green Warbler *Setophaga virens*

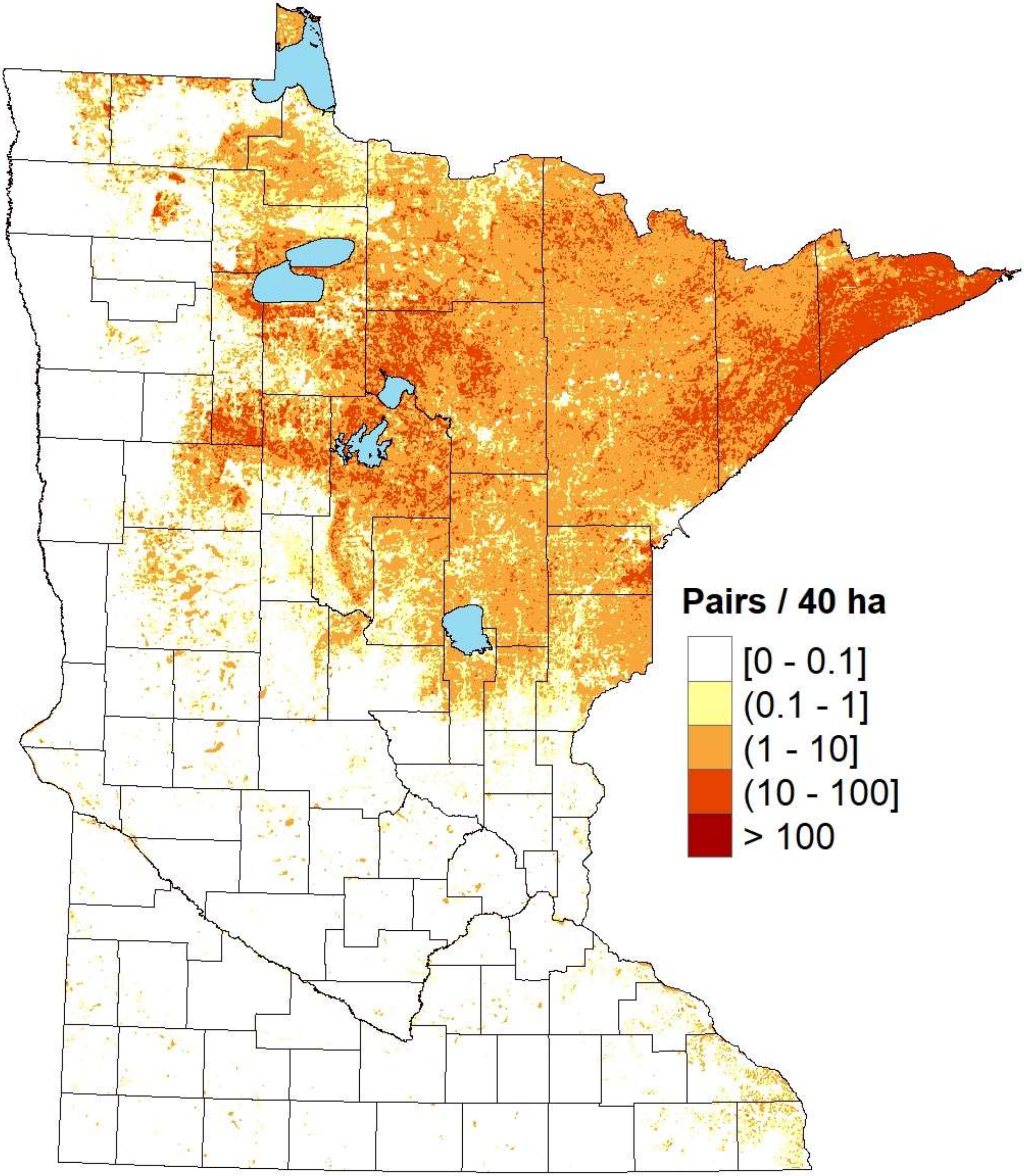

Blue-headed Vireo *Vireo solitarius*

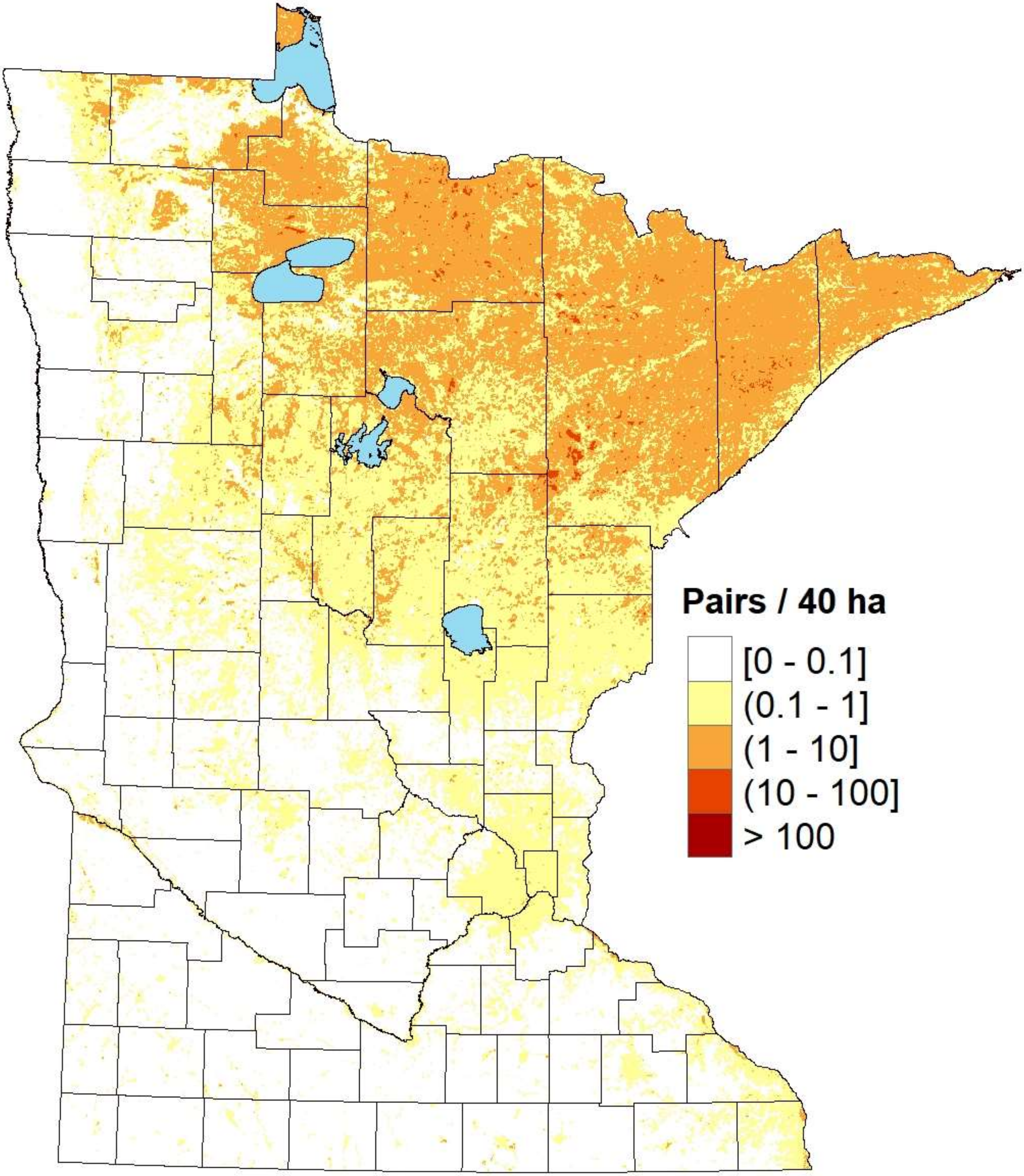

Bobolink *Dolichonyx oryzivorus*

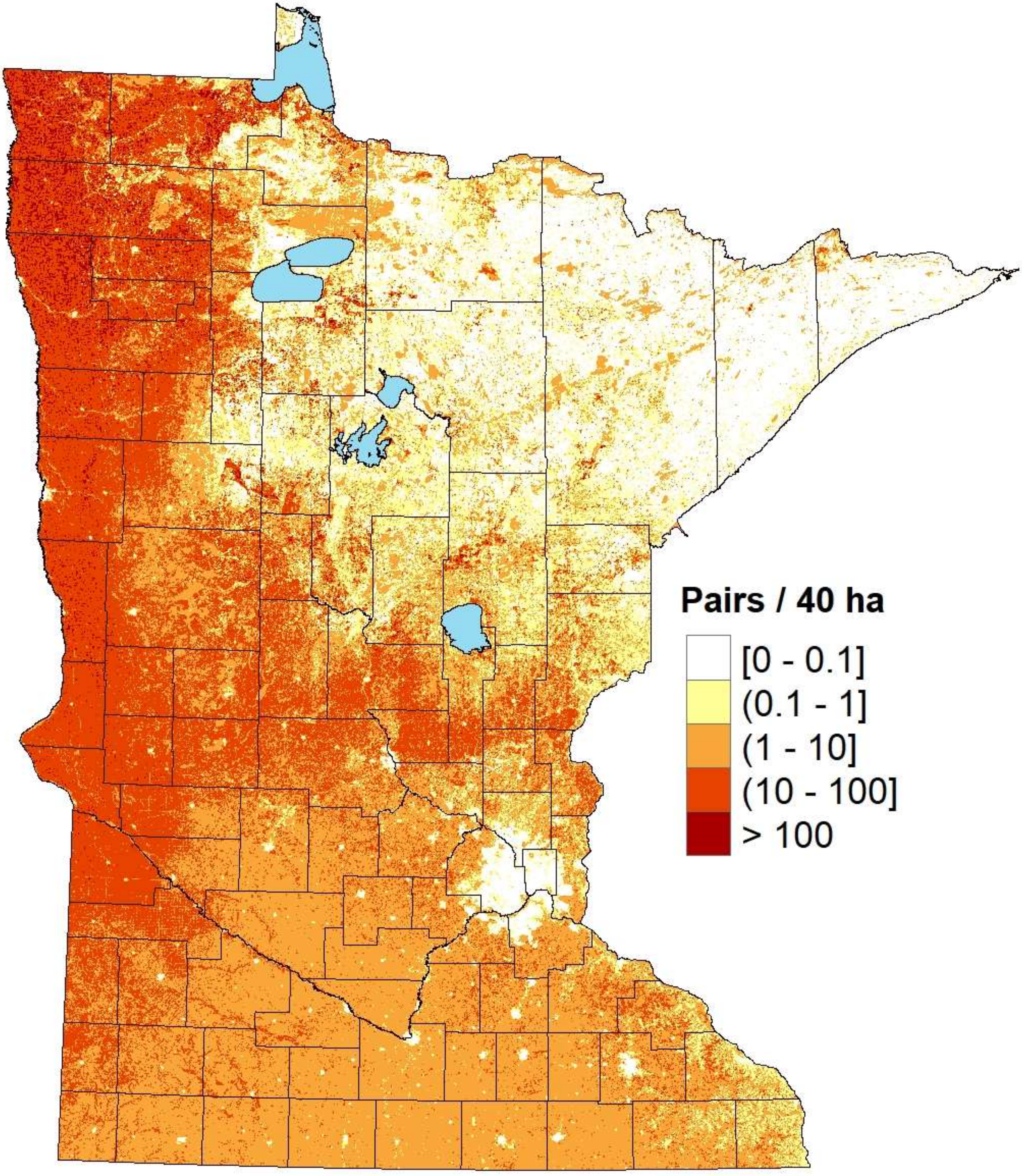

Brown Creeper *Certhia americana*

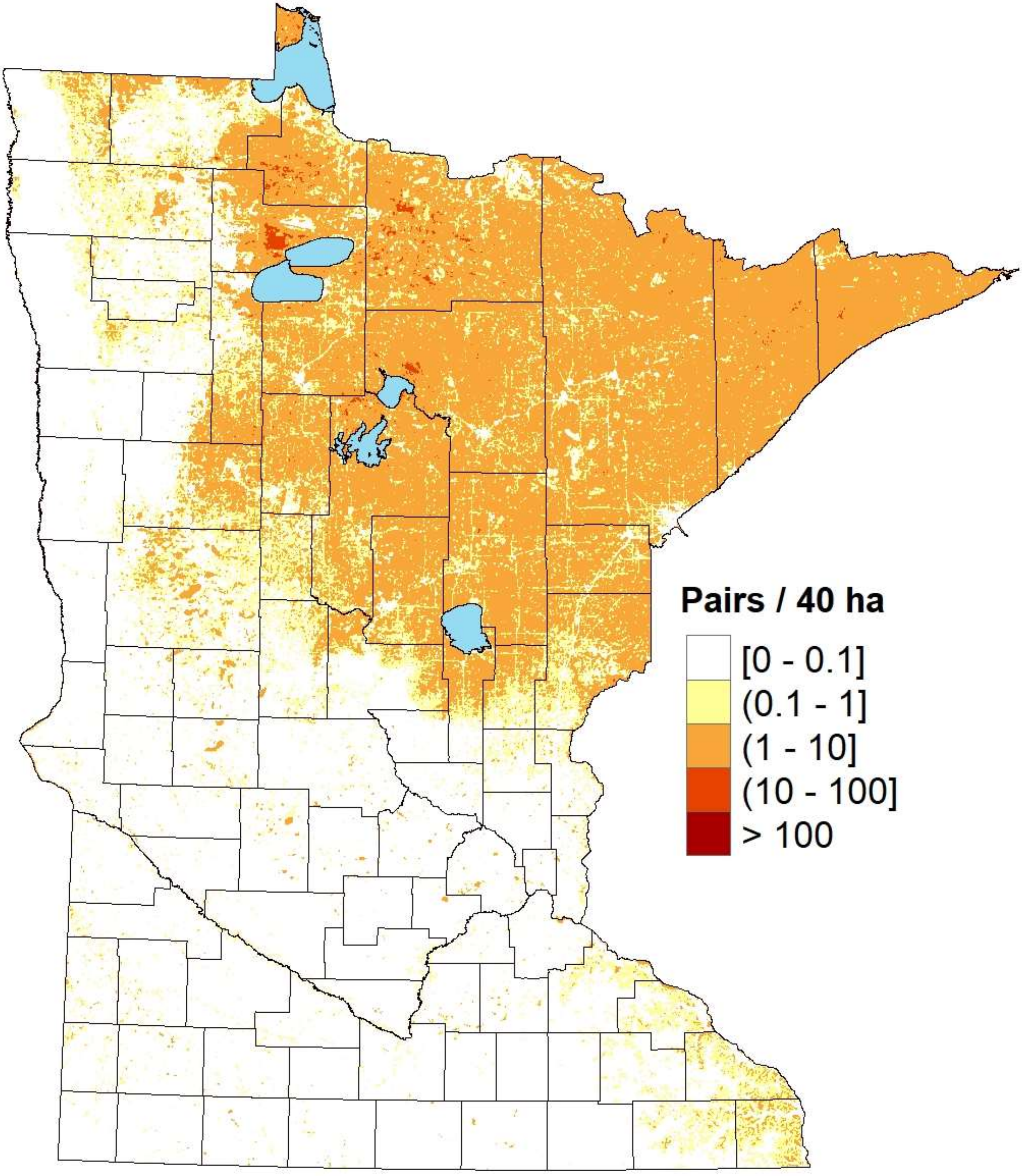

Brown Thrasher *Toxostoma rufum*

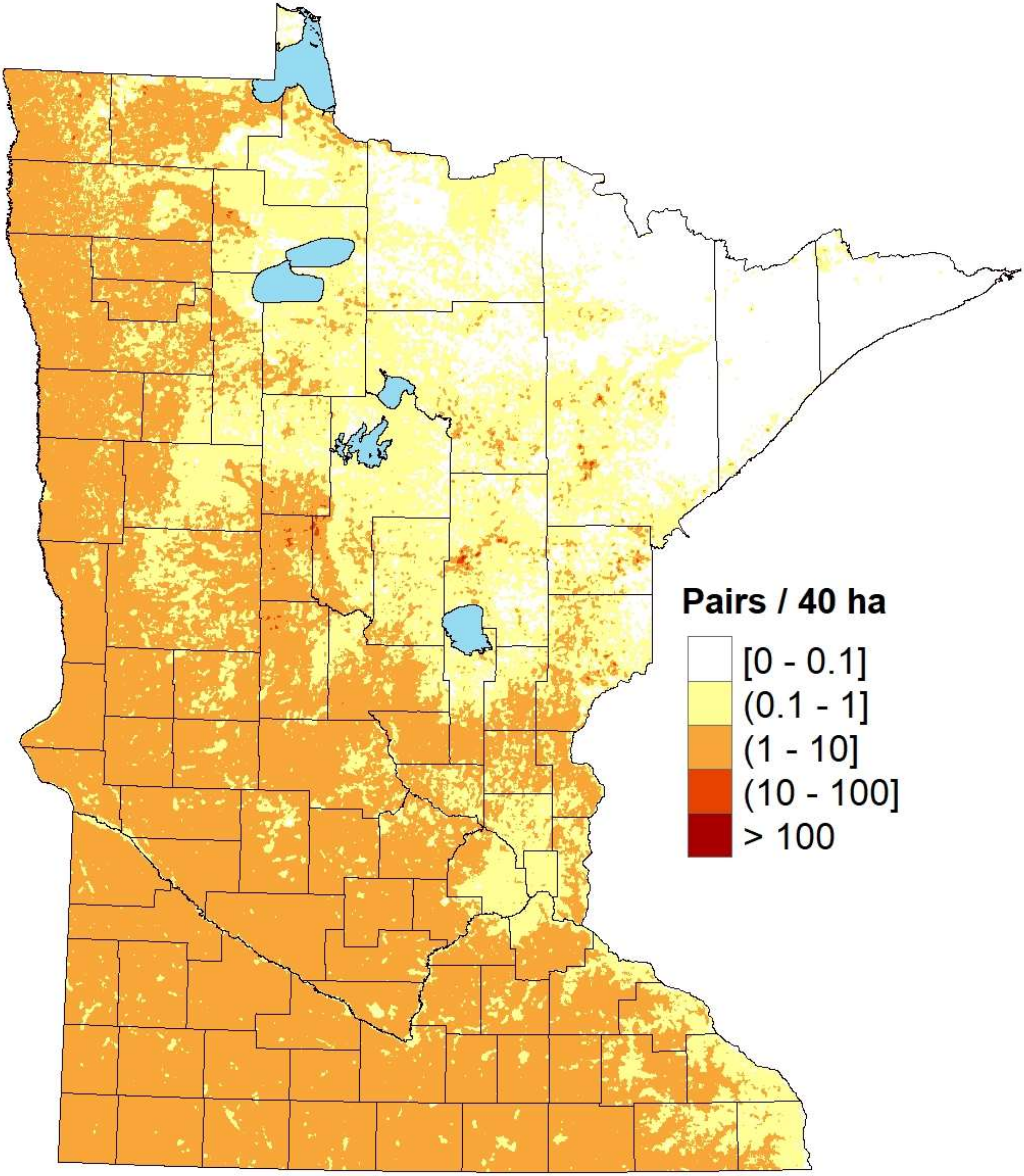

Canada Warbler *Cardellina canadensis*

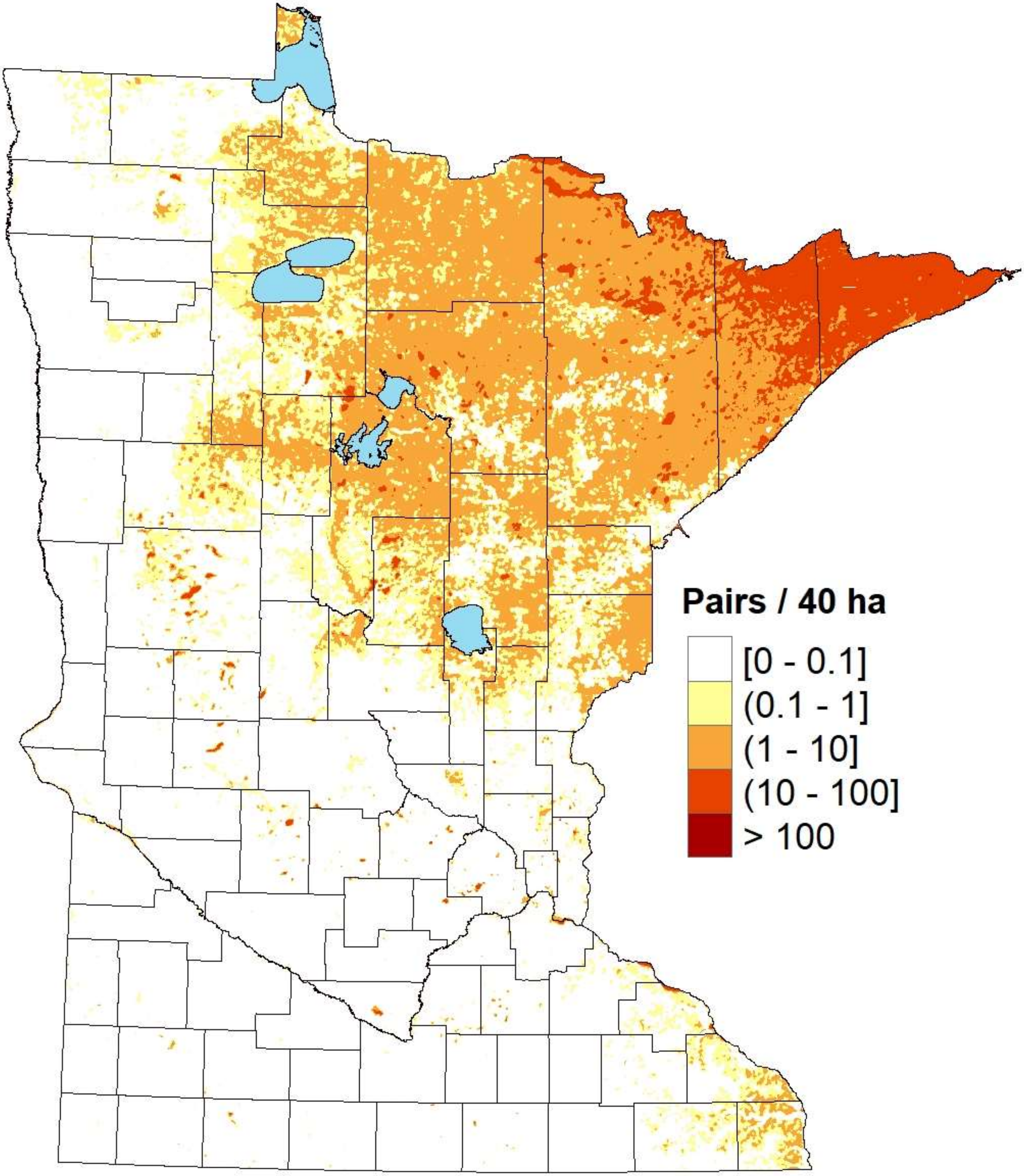

Cape May Warbler *Setophaga tigrina*

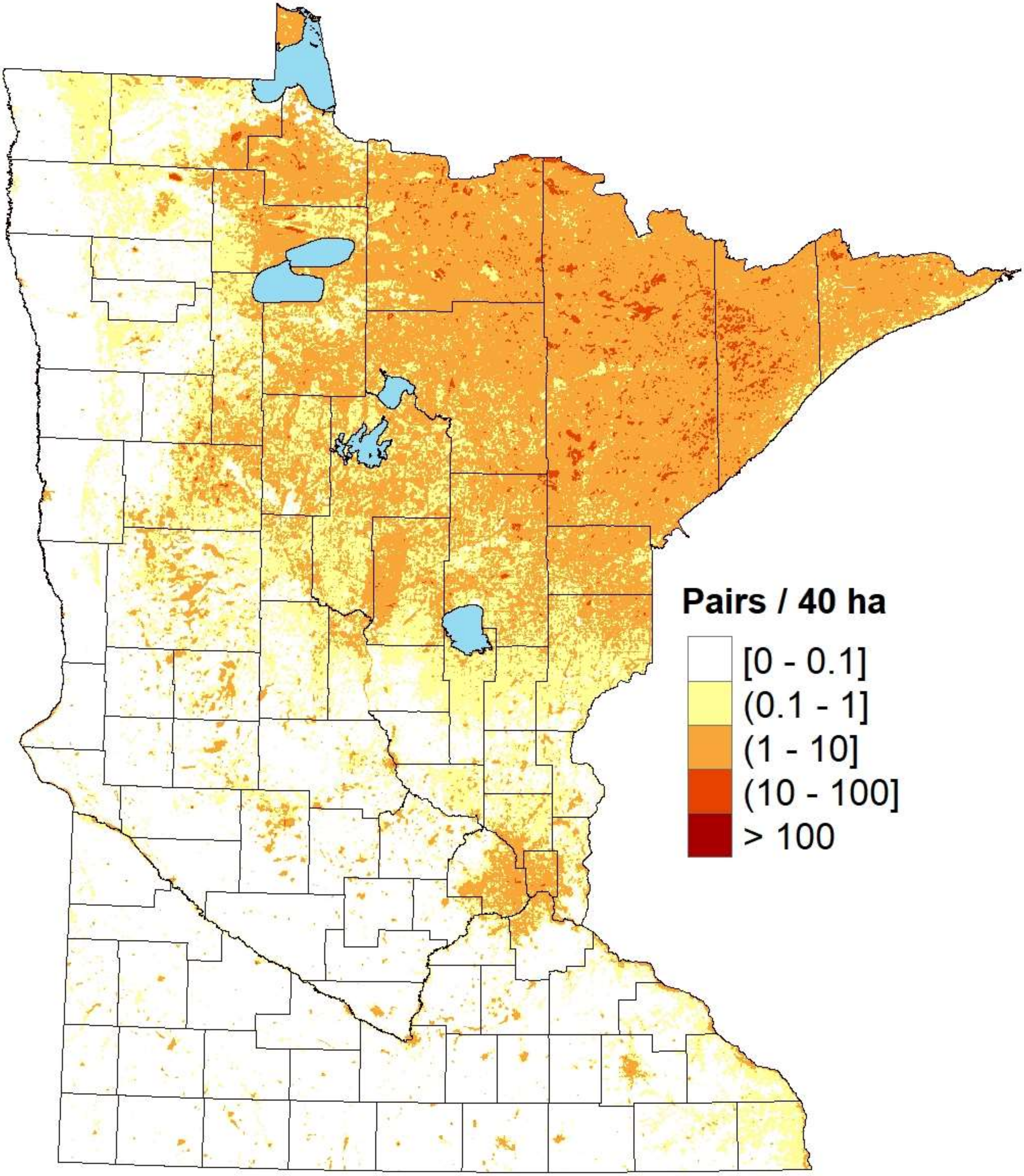

Chestnut-sided Warbler *Setophaga pensylvanica*

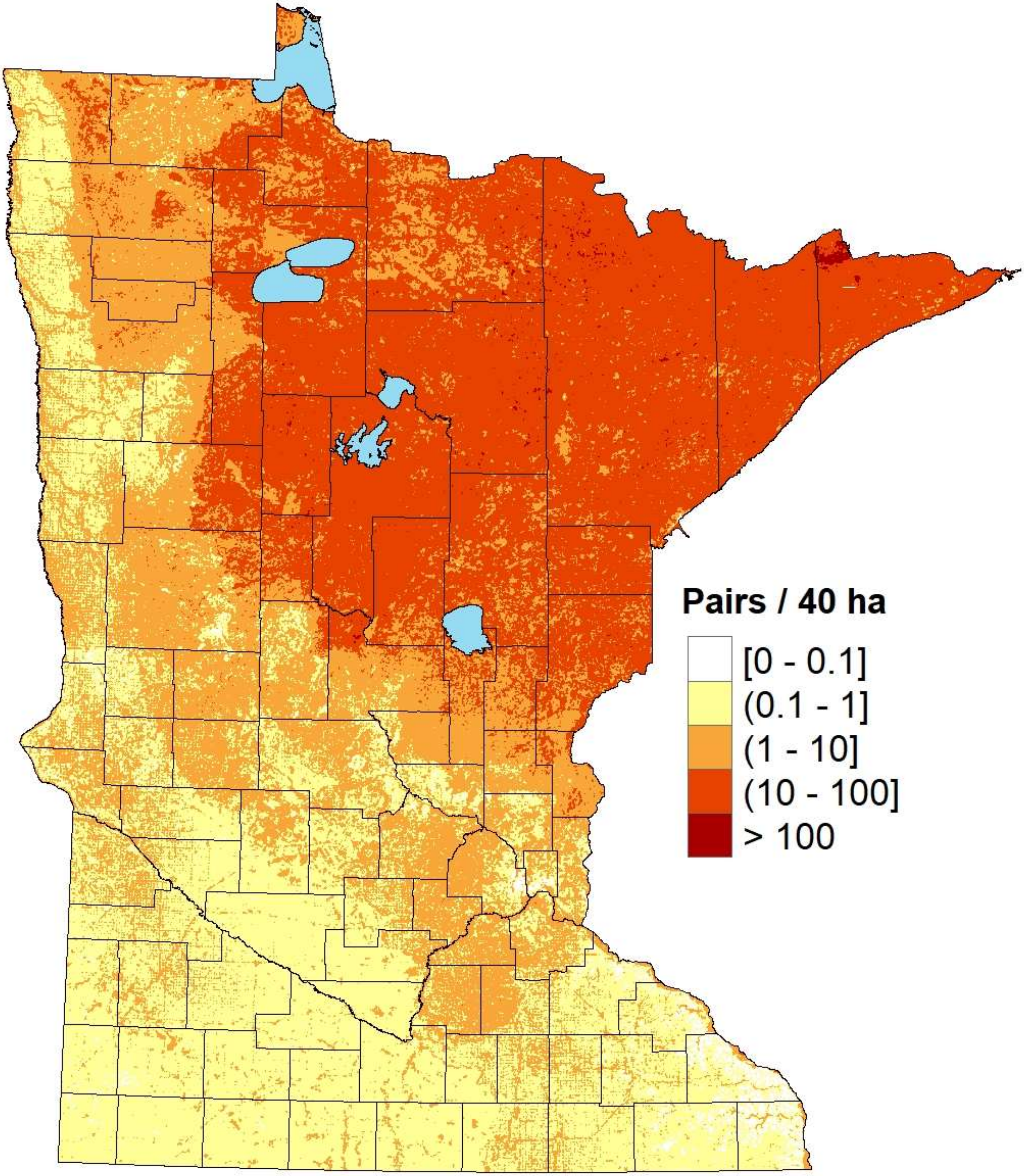

Chipping Sparrow *Spizella passerina*

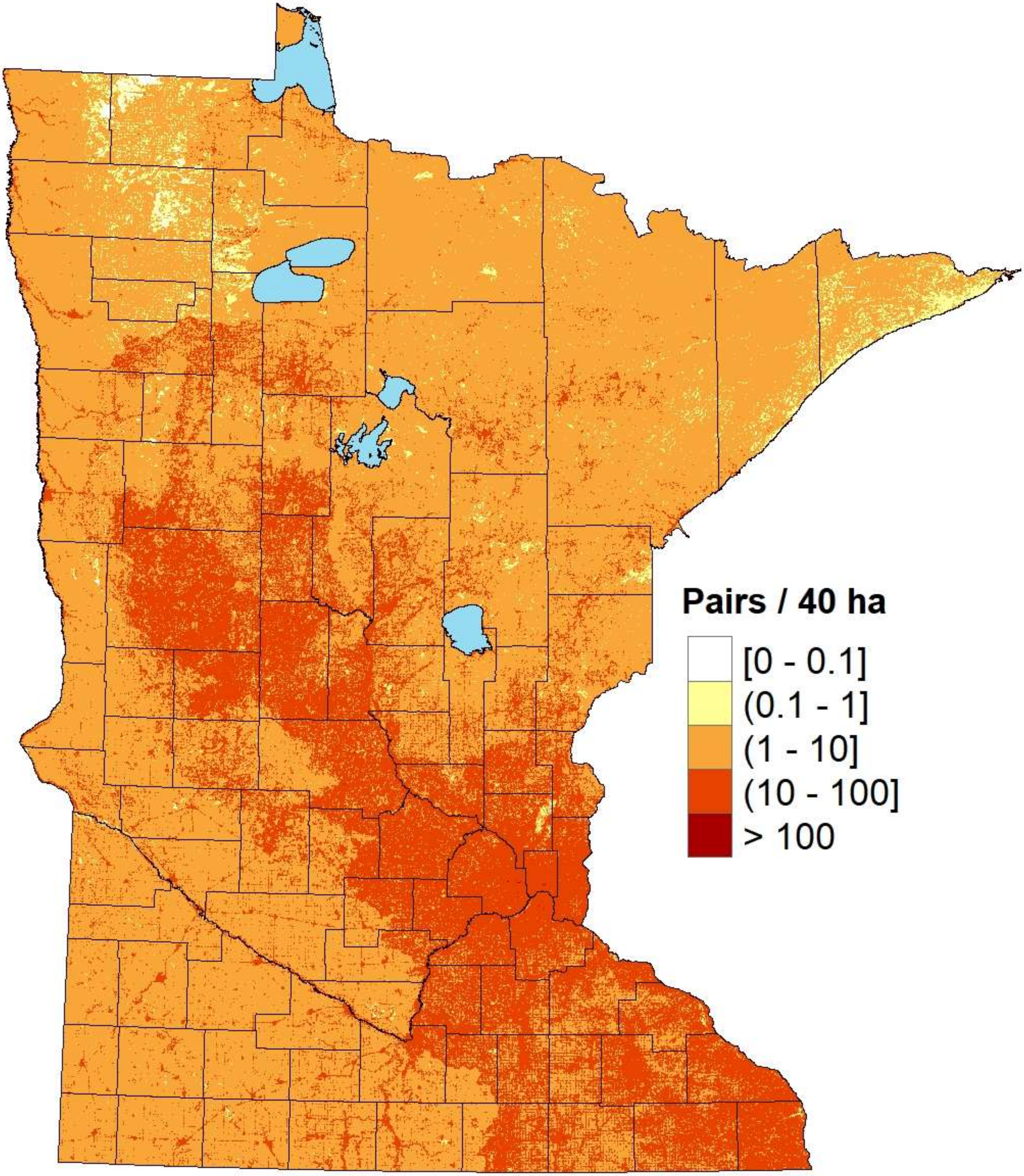

Clay-colored Sparrow *Spizella pallida*

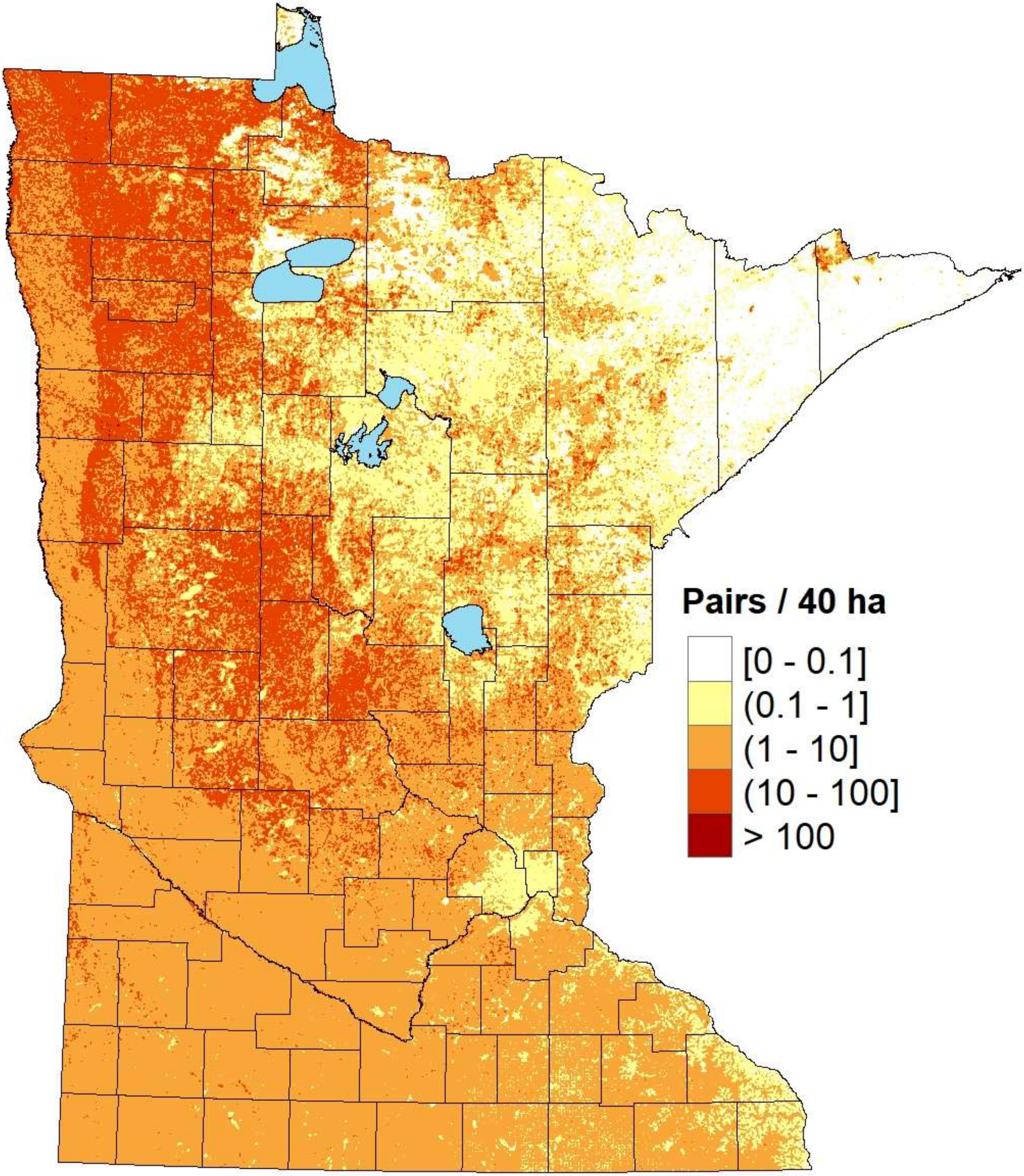

Common Yellowthroat *Geothlypis trichas*

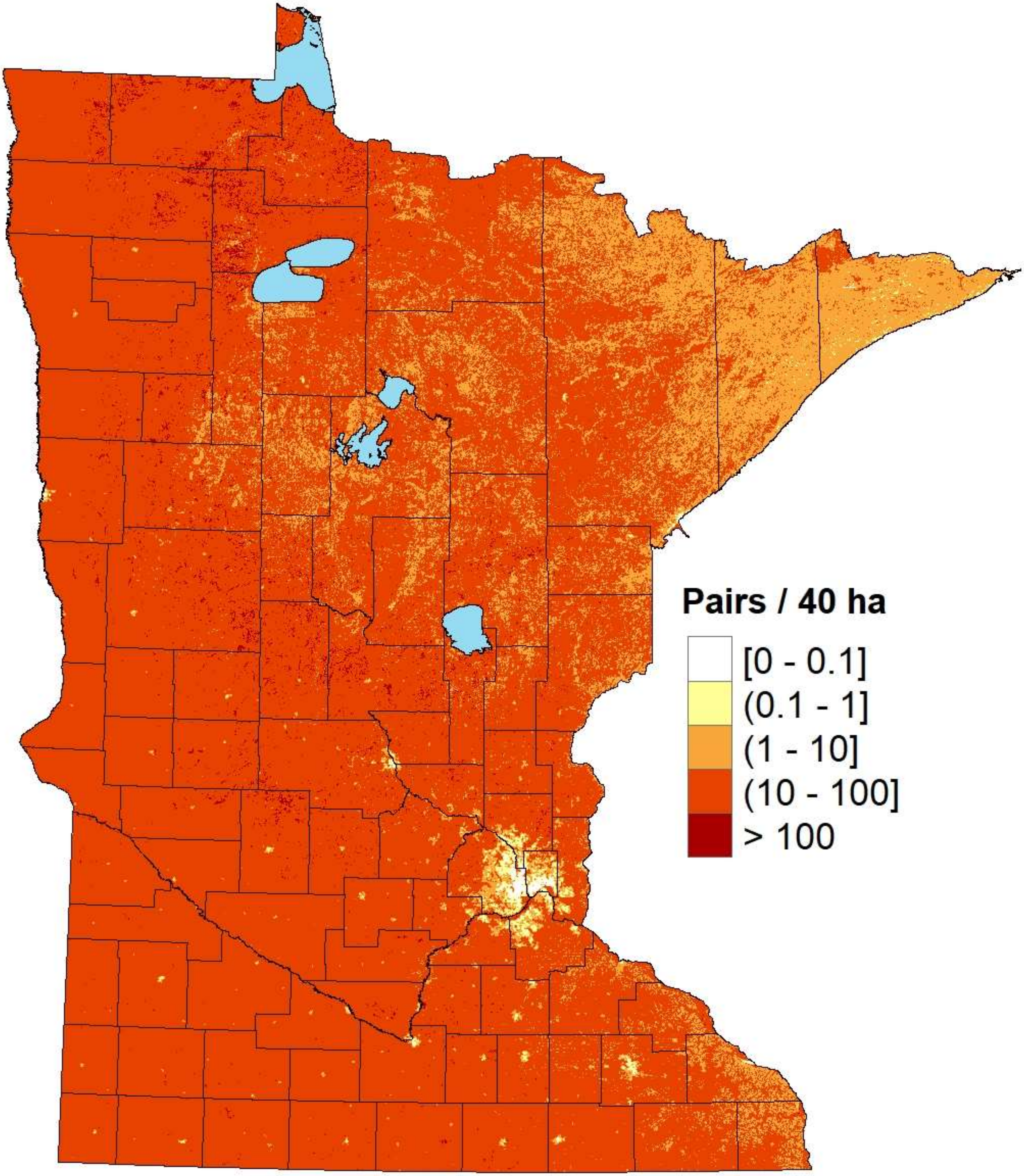

Connecticut Warbler *Oporornis agilis*

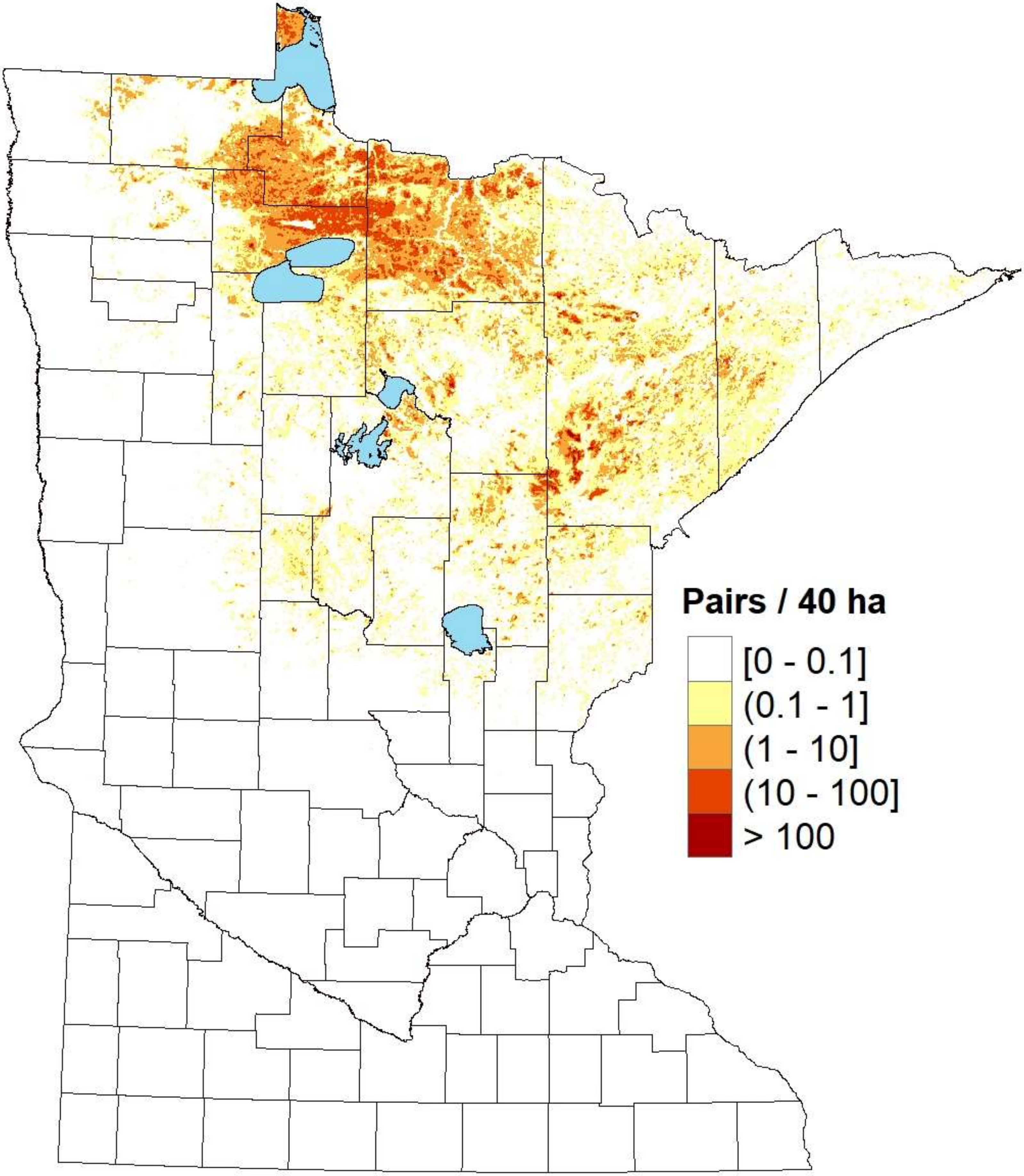

Dark-eyed Junco *Junco hyemalis*

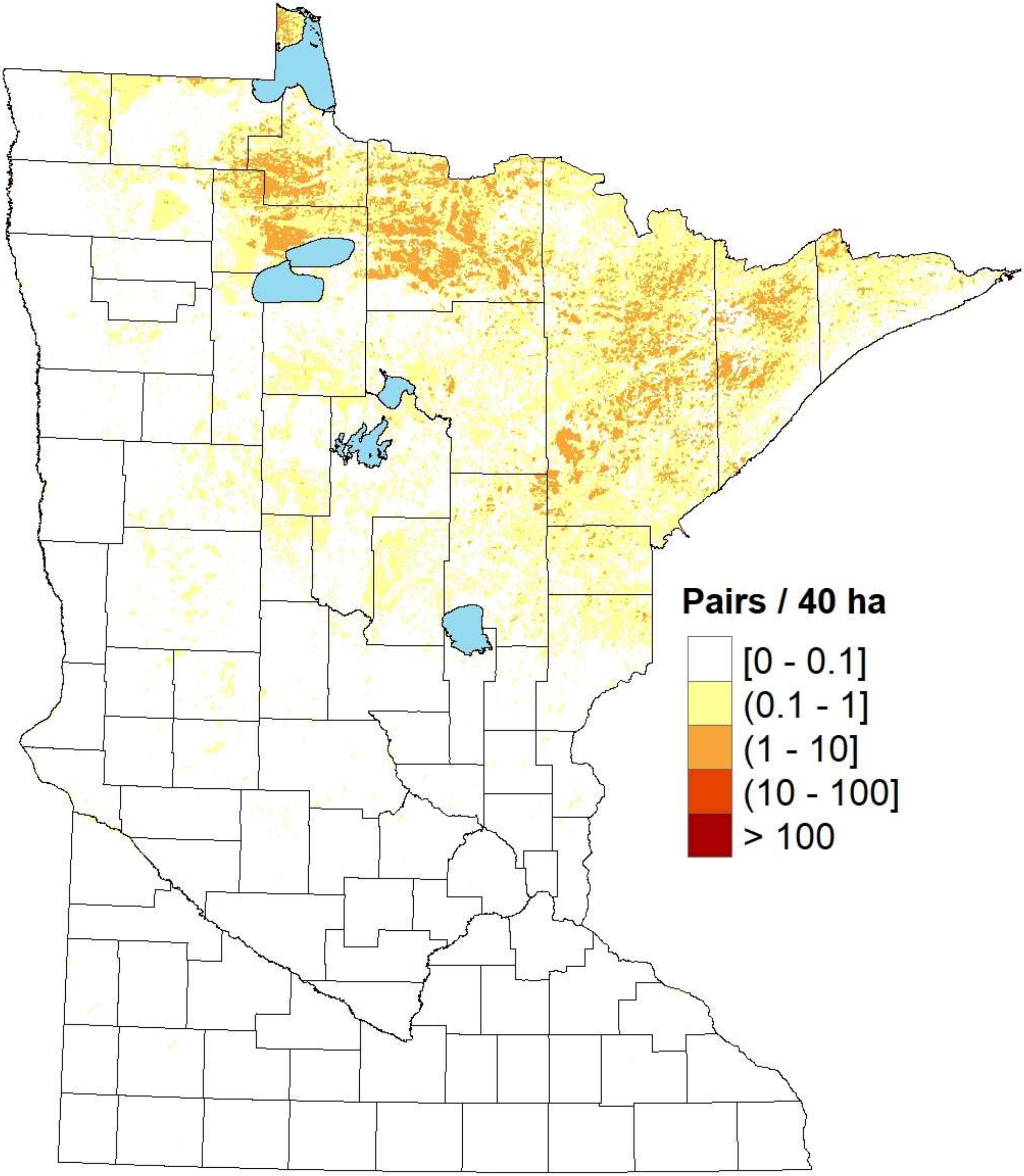

Dickcissel *Spiza americana*

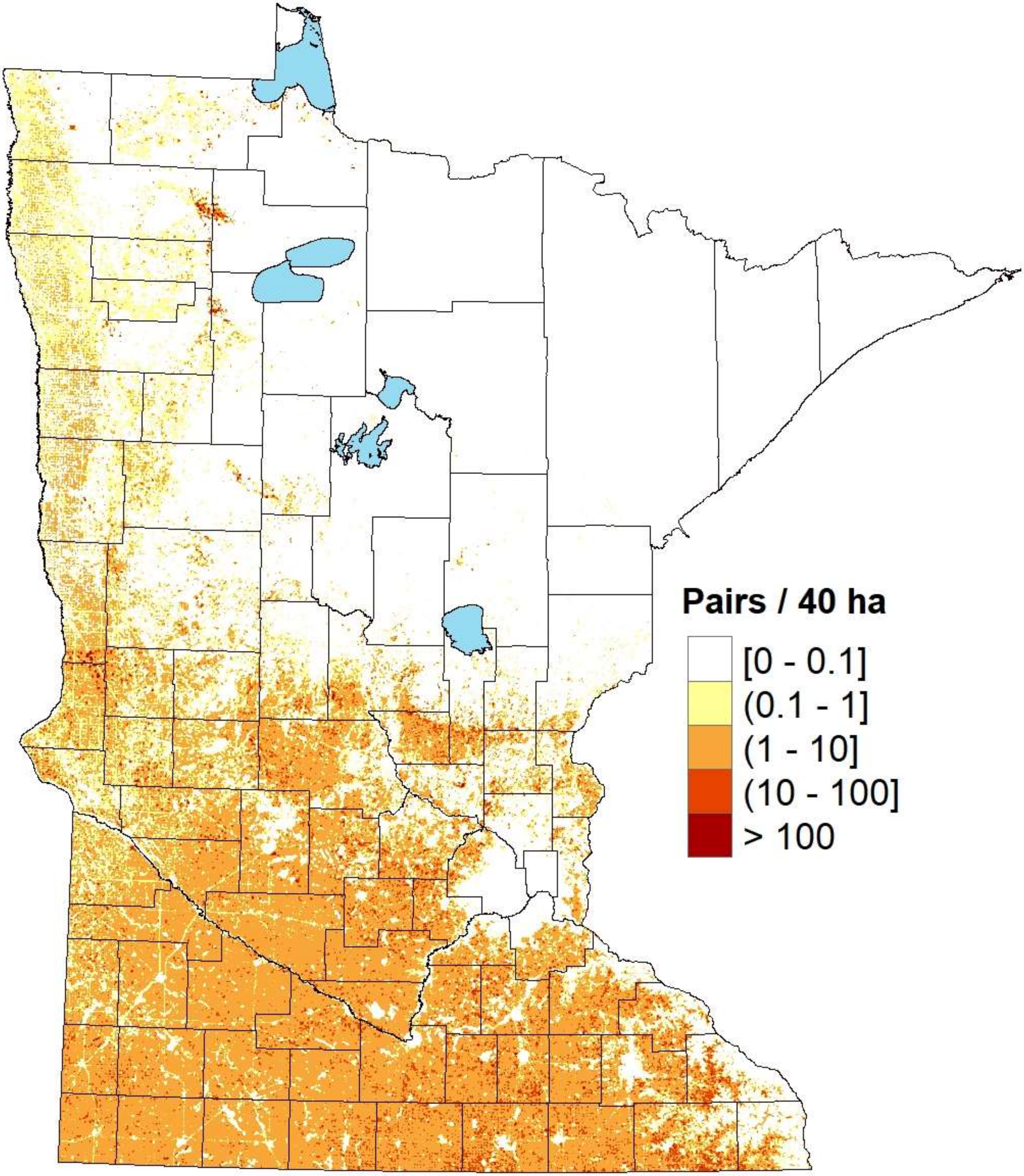

Eastern Bluebird *Sialia sialis*

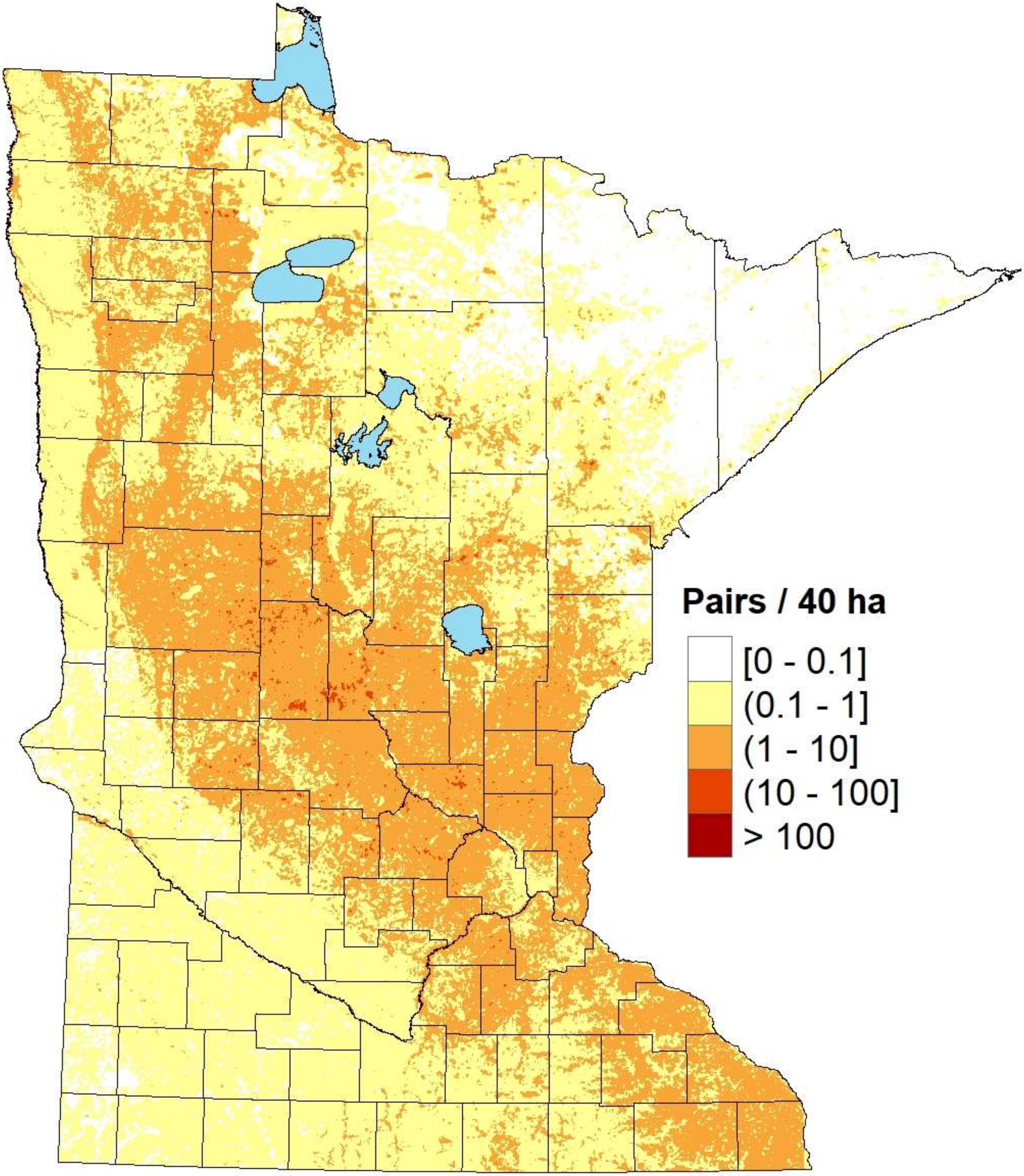

Eastern Meadowlark *Sturnella magna*

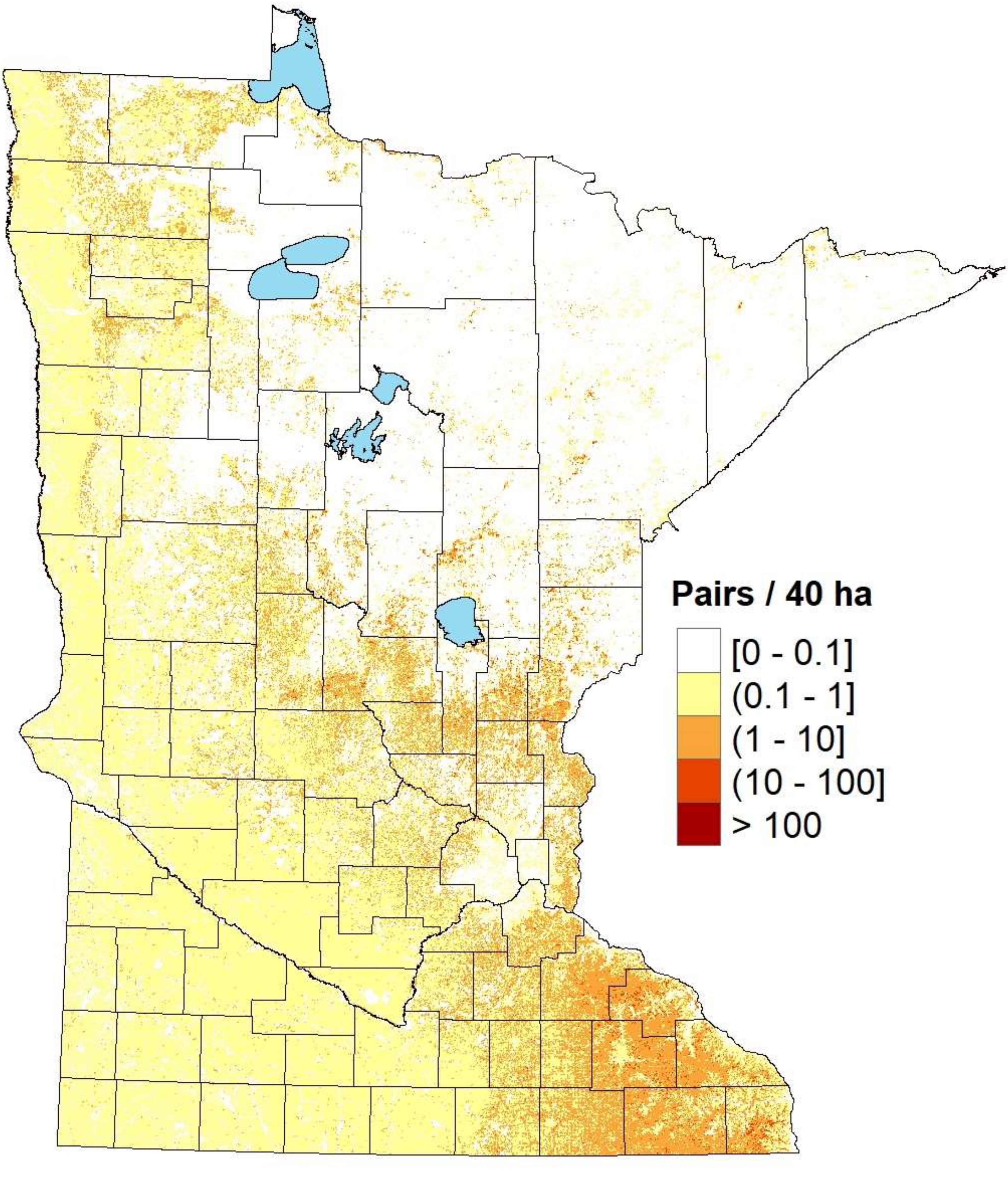

Eastern Phoebe *Sayornis phoebe*

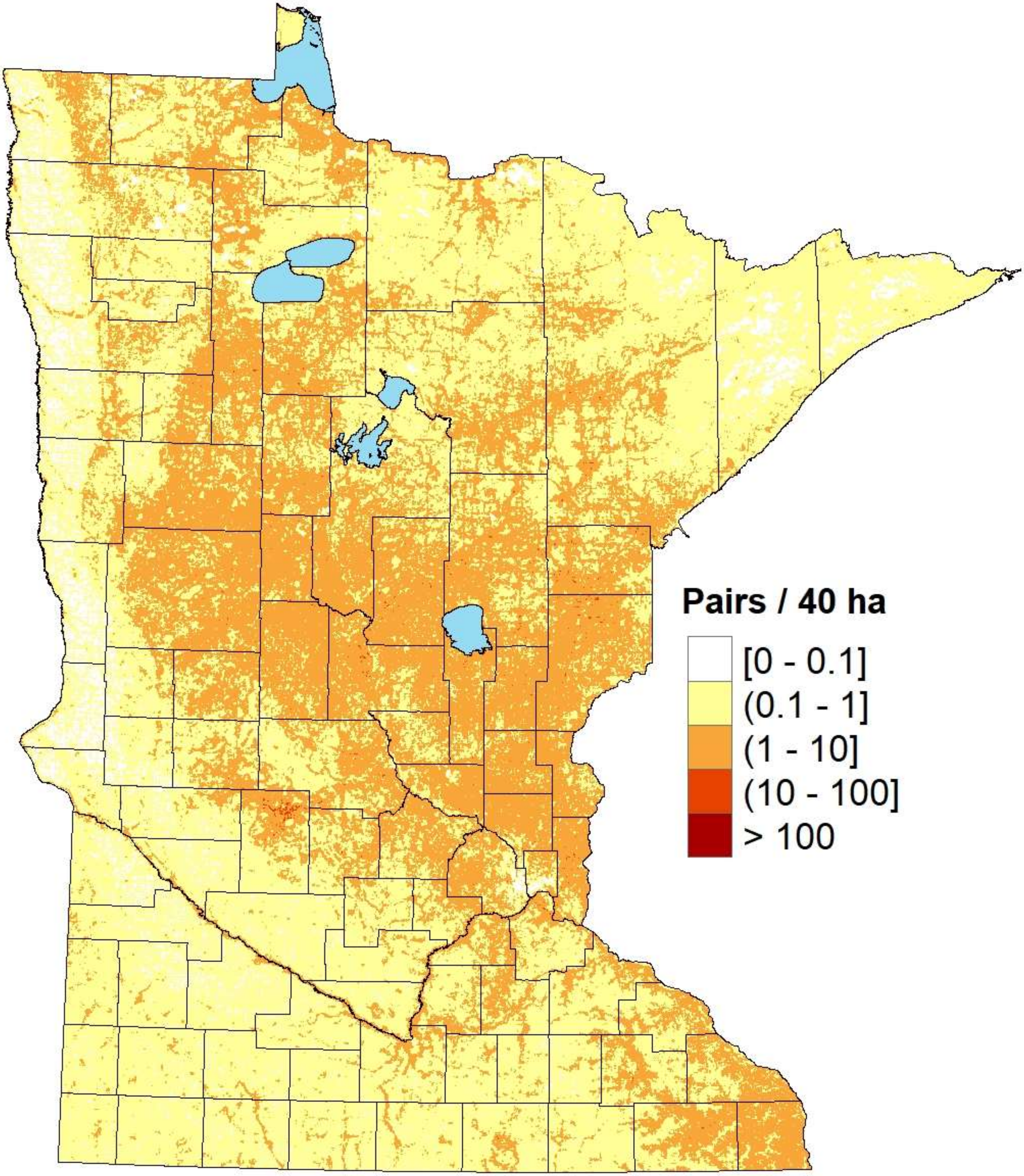

Eastern Wood-Pewee *Contopus virens*

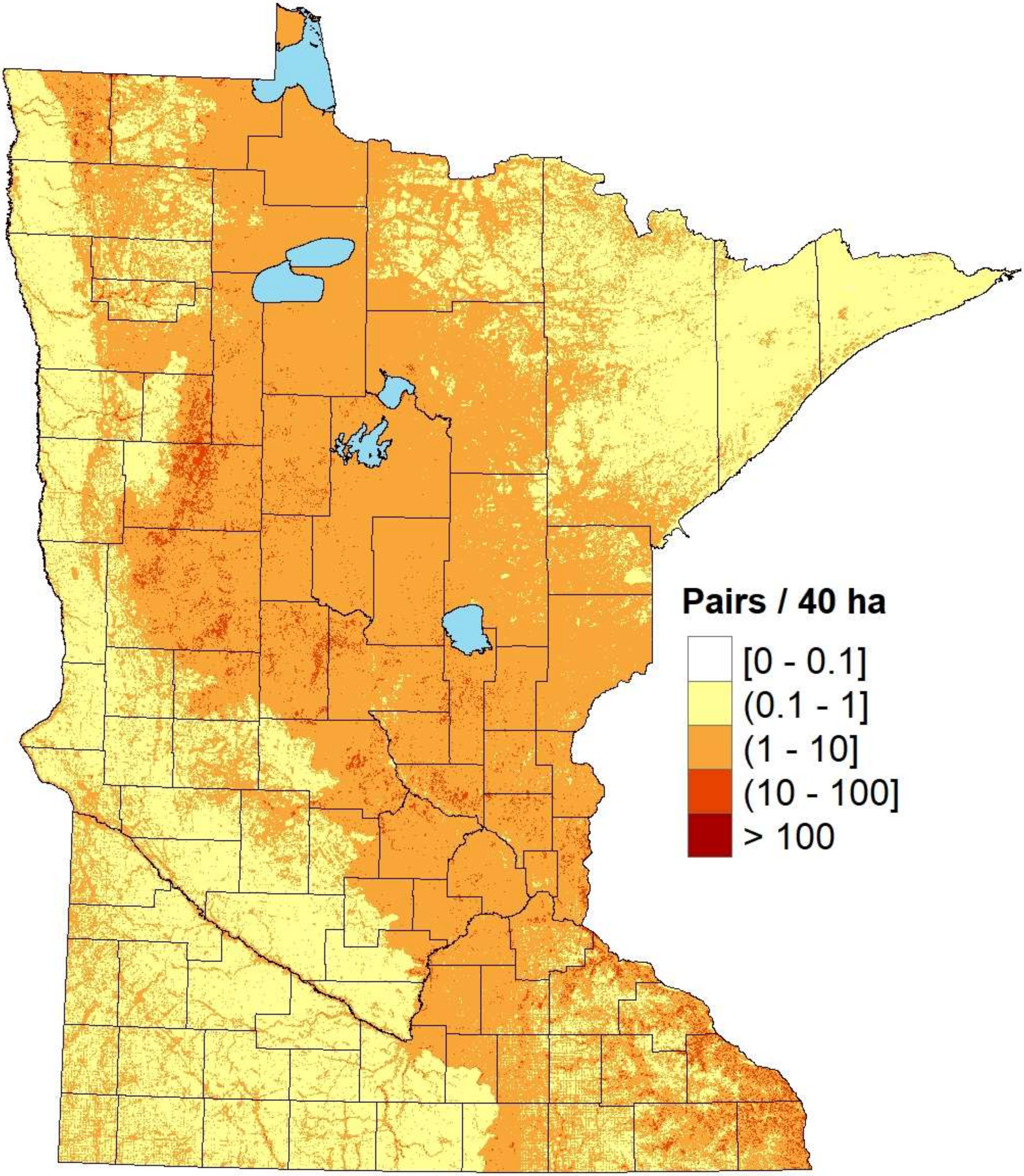

Field Sparrow *Spizella pusilla*

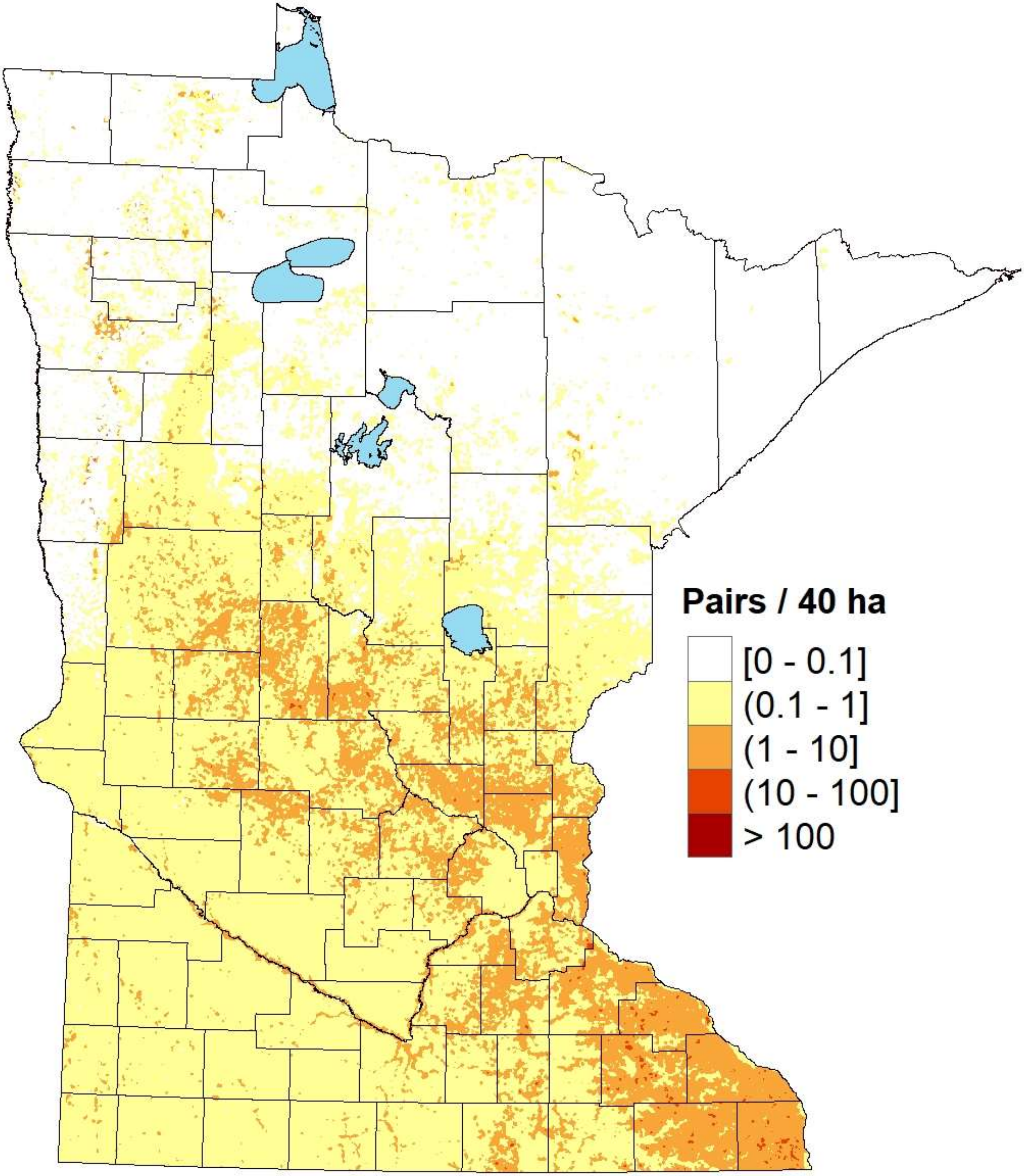

Golden-crowned Kinglet *Regulus satrapa*

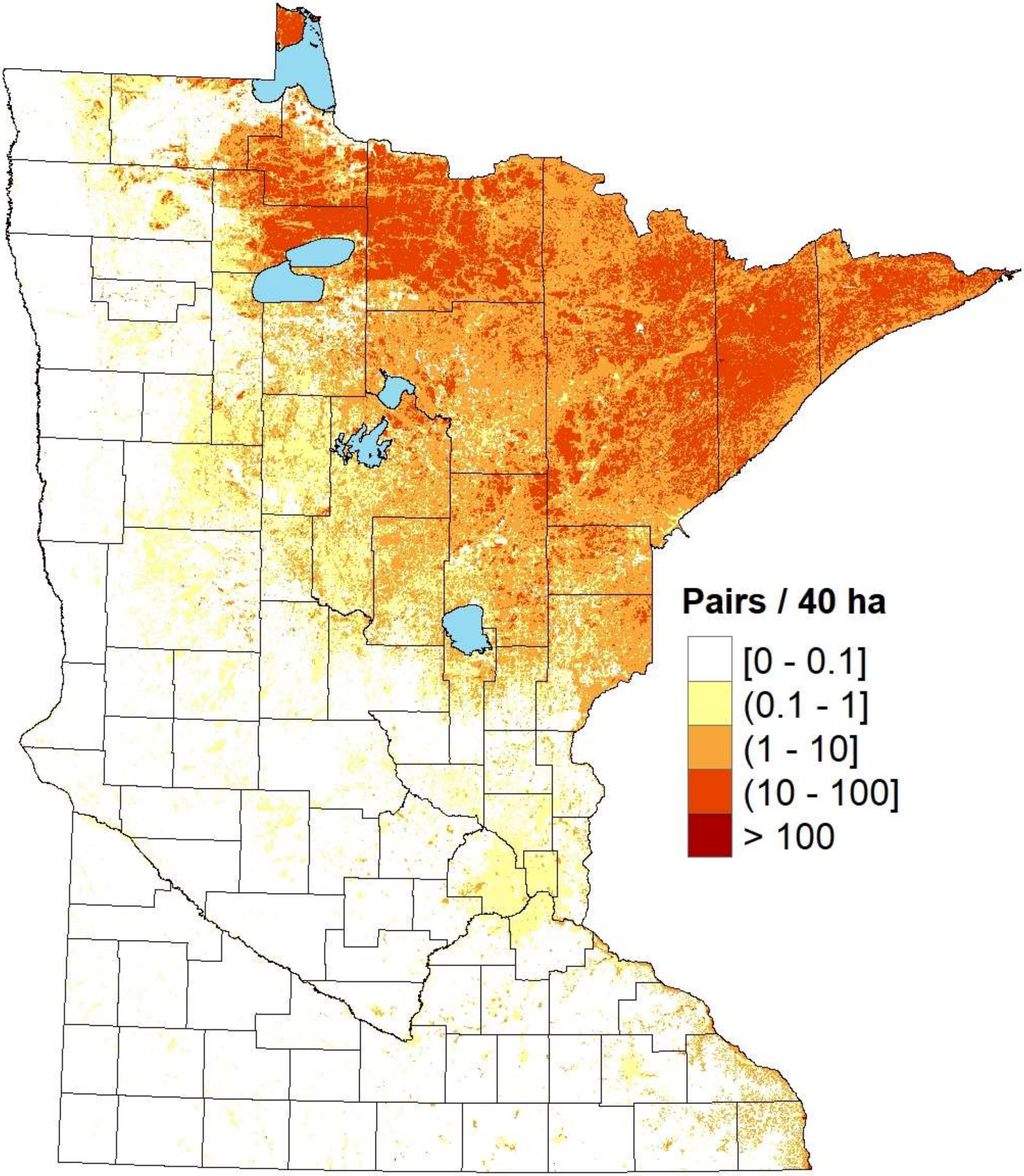

Golden-winged Warbler *Vermivora chrysoptera*

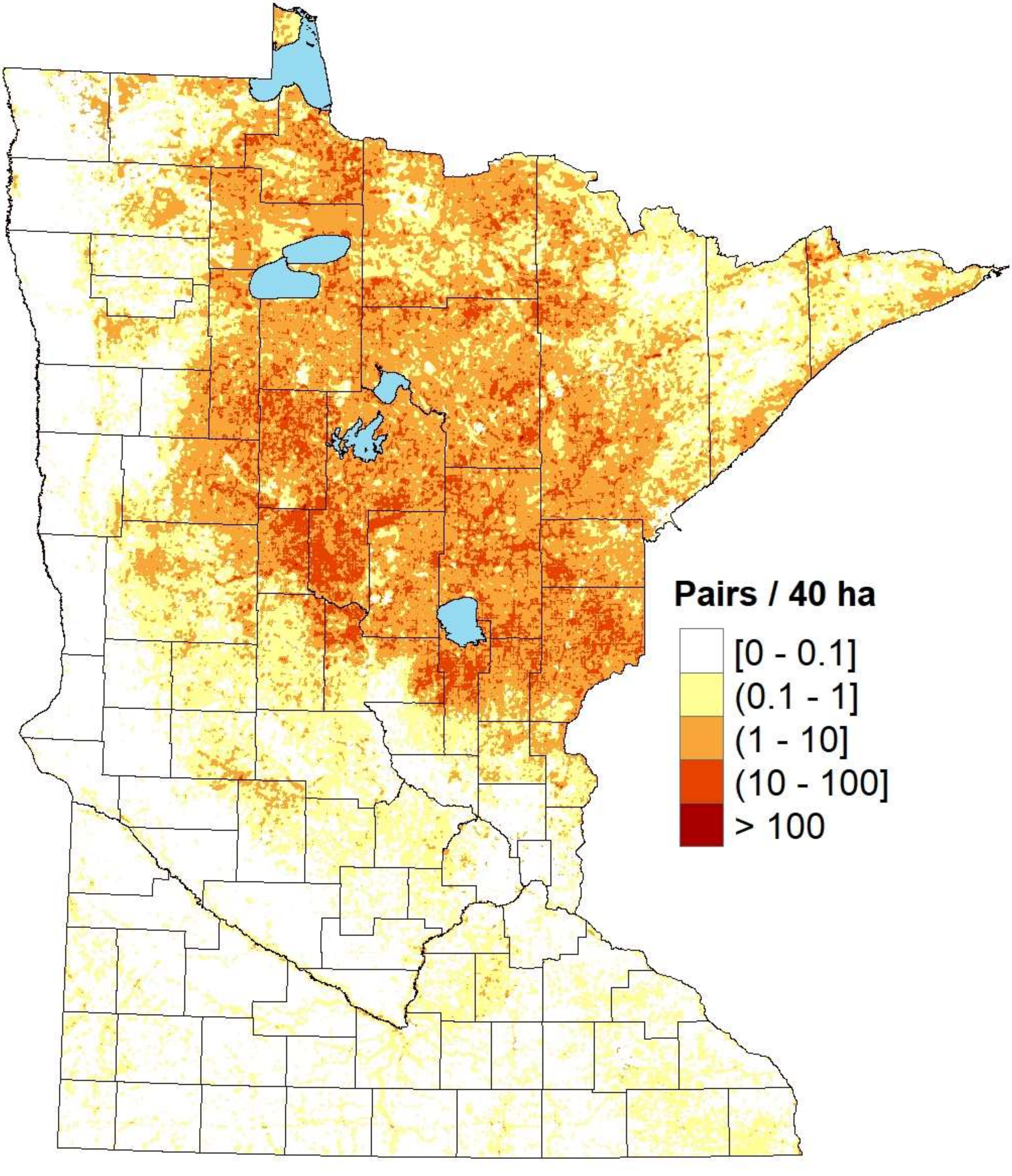

Grasshopper Sparrow *Ammodramus savannarum*

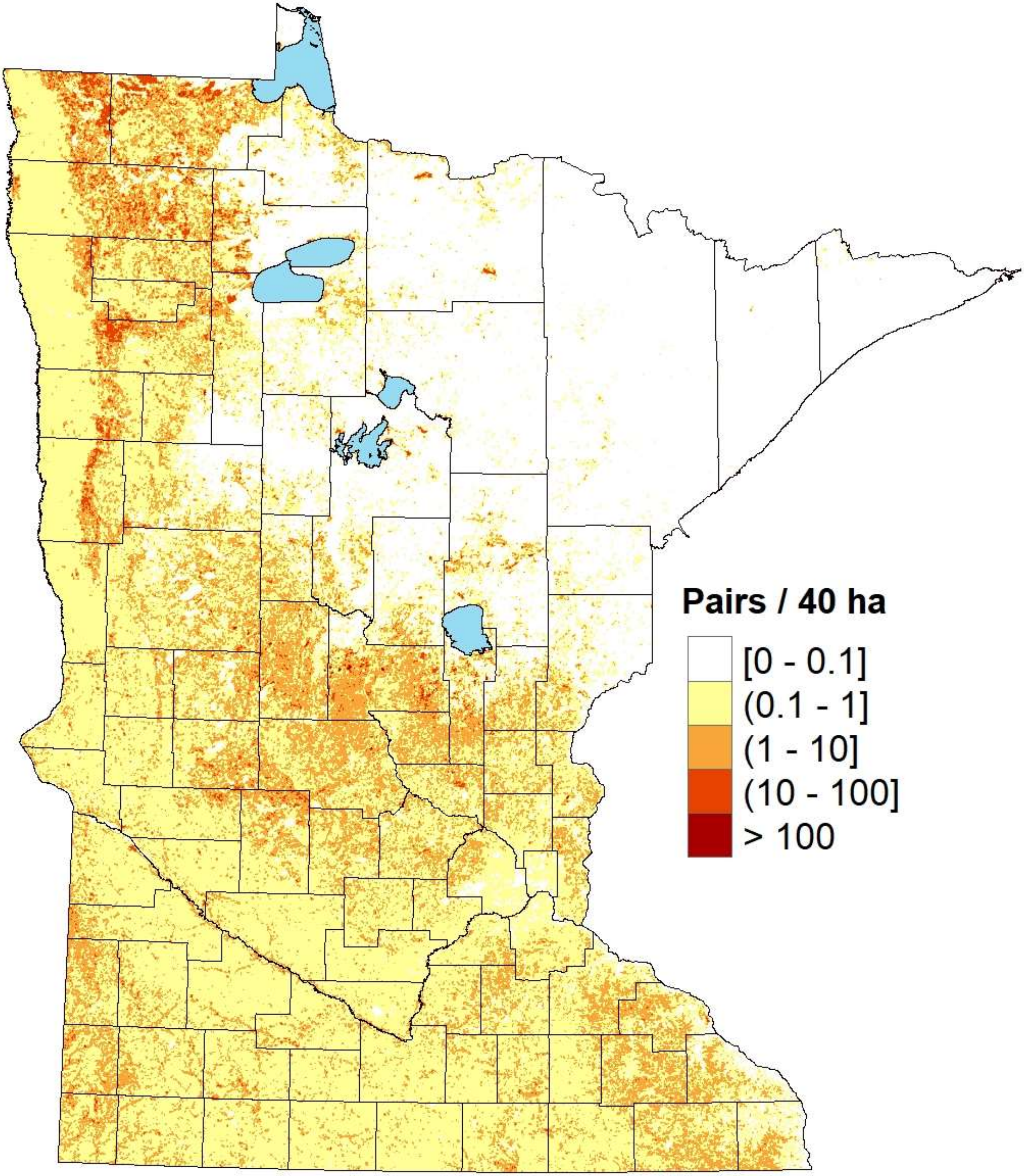

Gray Catbird *Dumetella carolinensis*

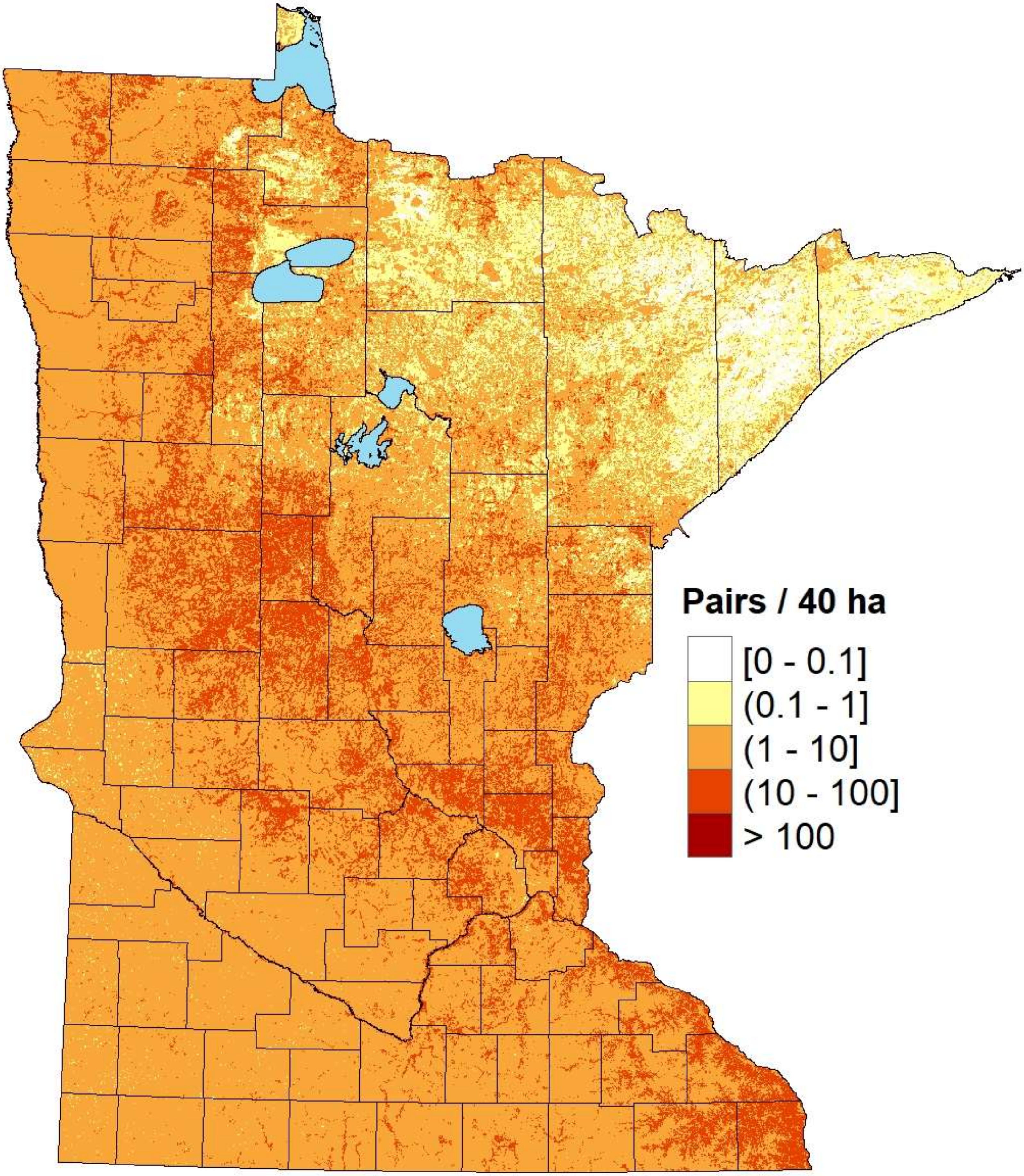

Great Crested Flycatcher *Myiarchus crinitus*

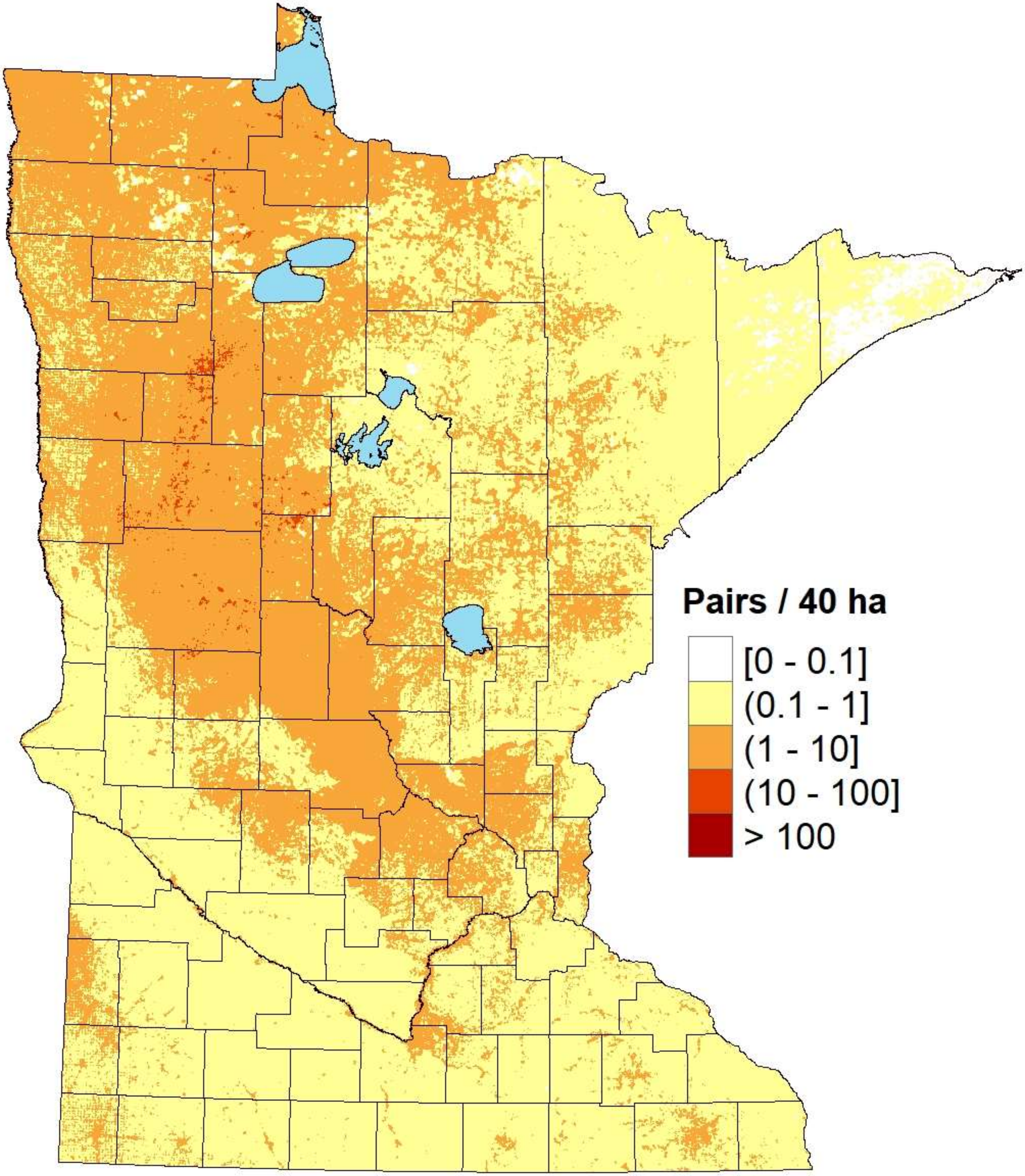

Hermit Thrush *Catharus guttatus*

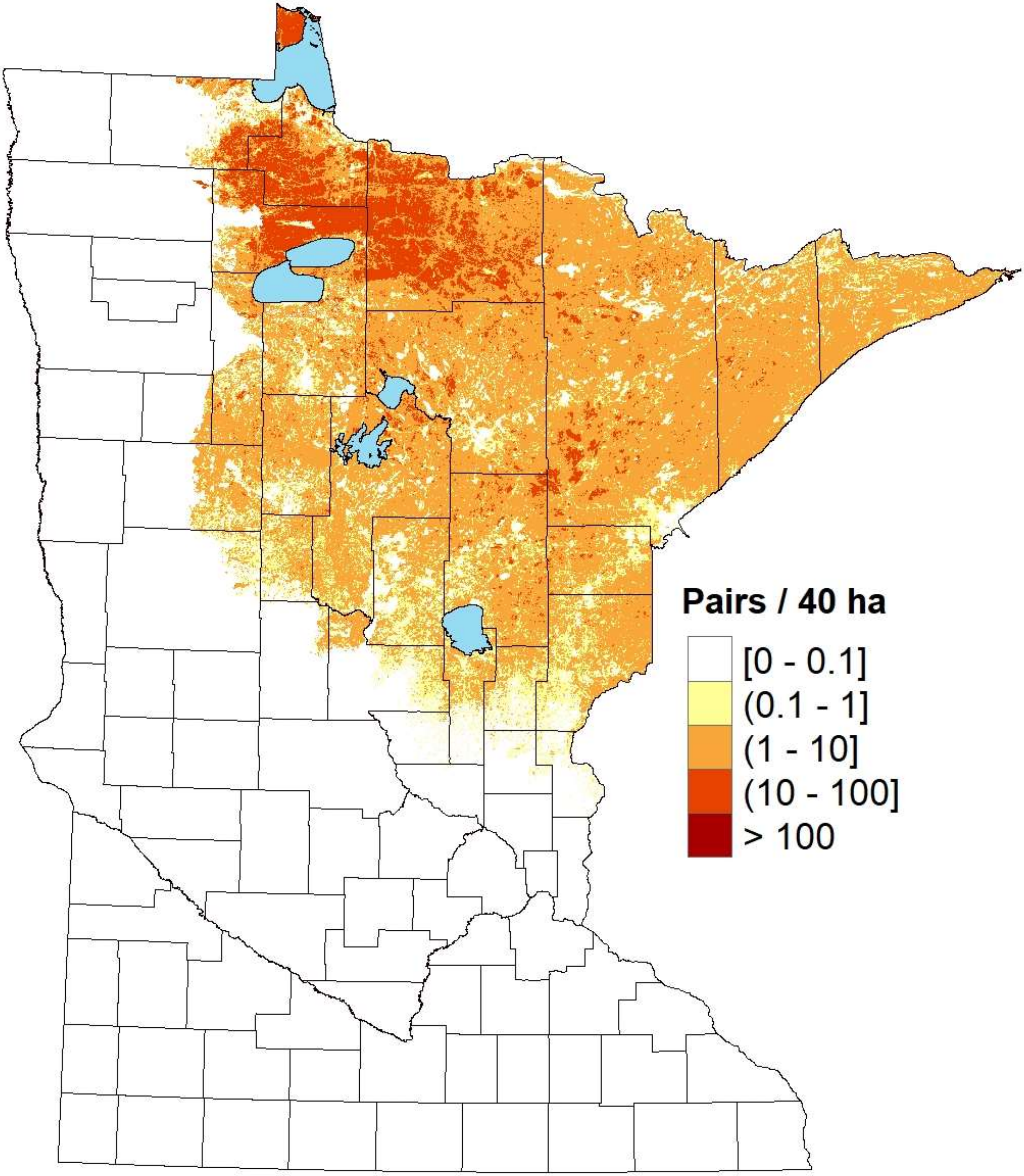

Horned Lark *Eremophila alpestris*

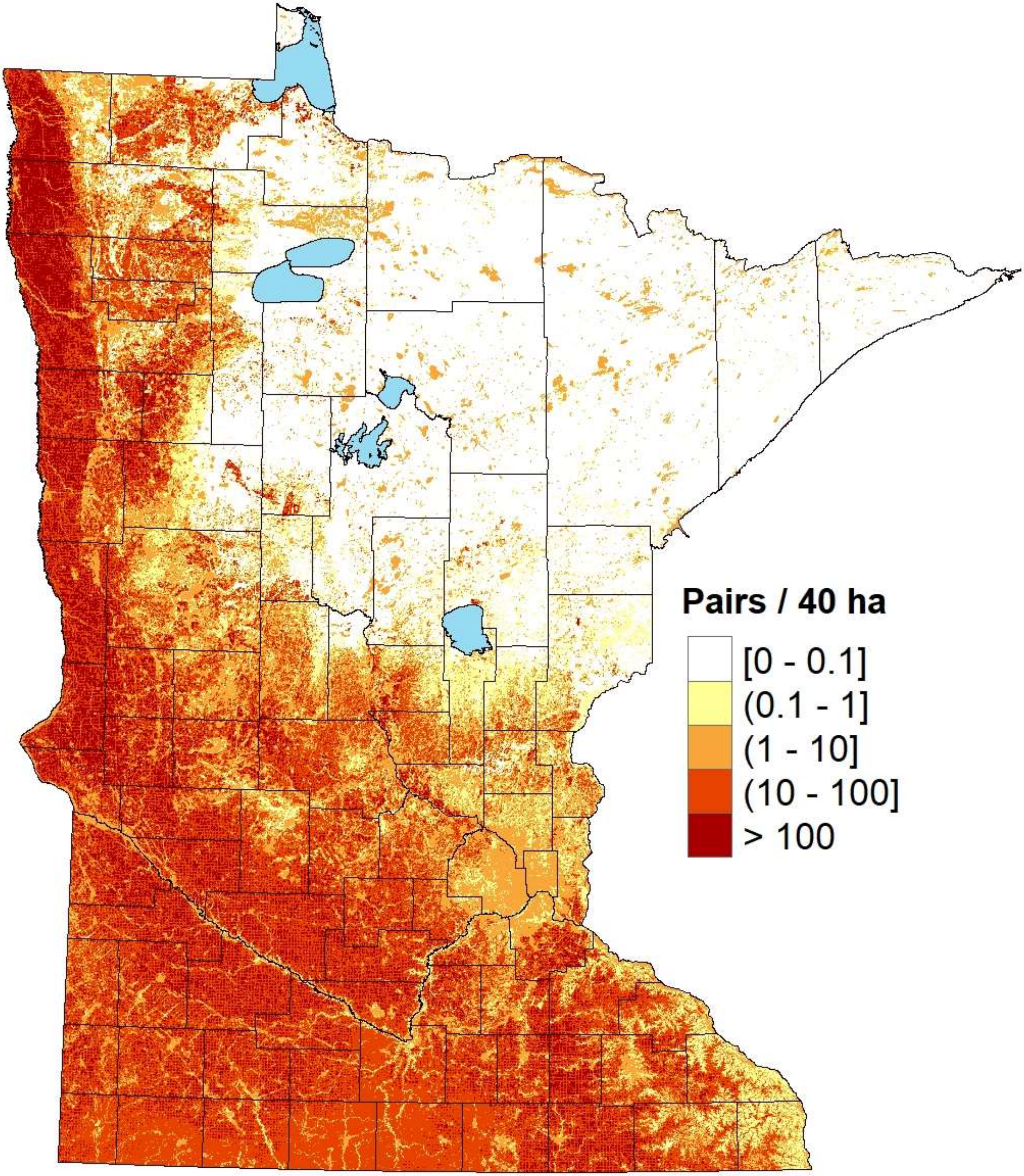

House Finch *Haemorhous mexicanus*

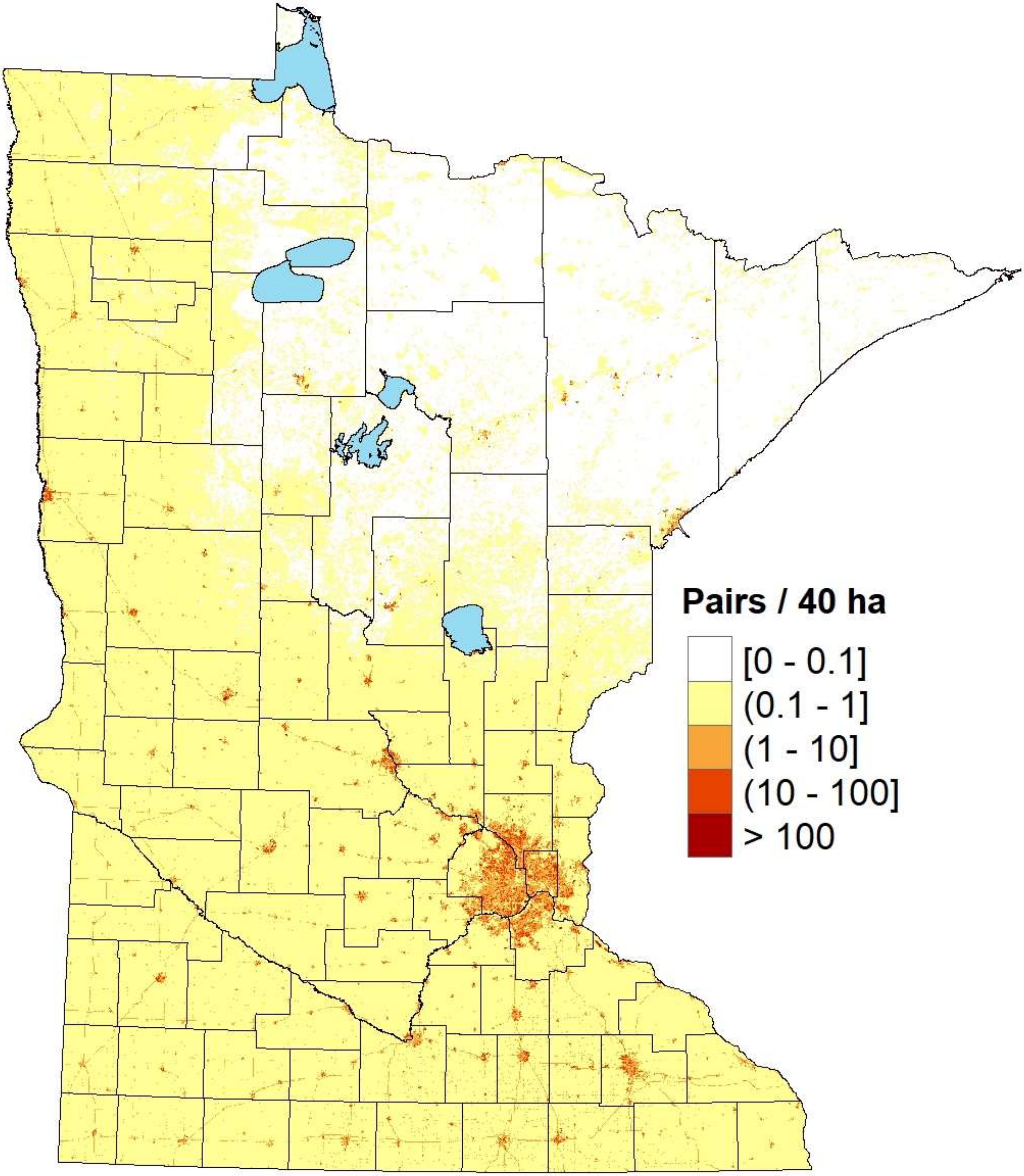

House Wren *Troglodytes aedon*

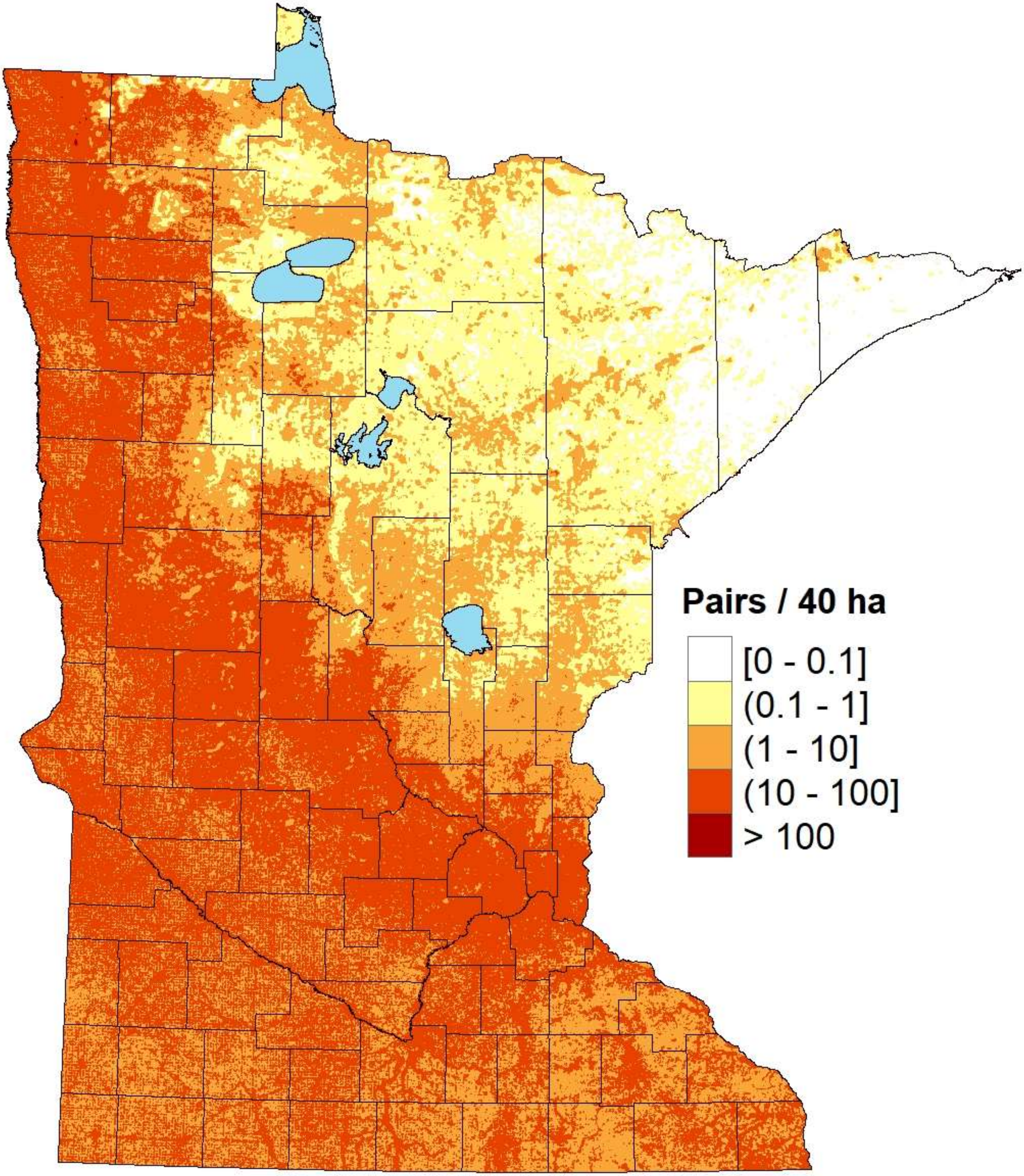

Indigo Bunting *Passerina cyanea*

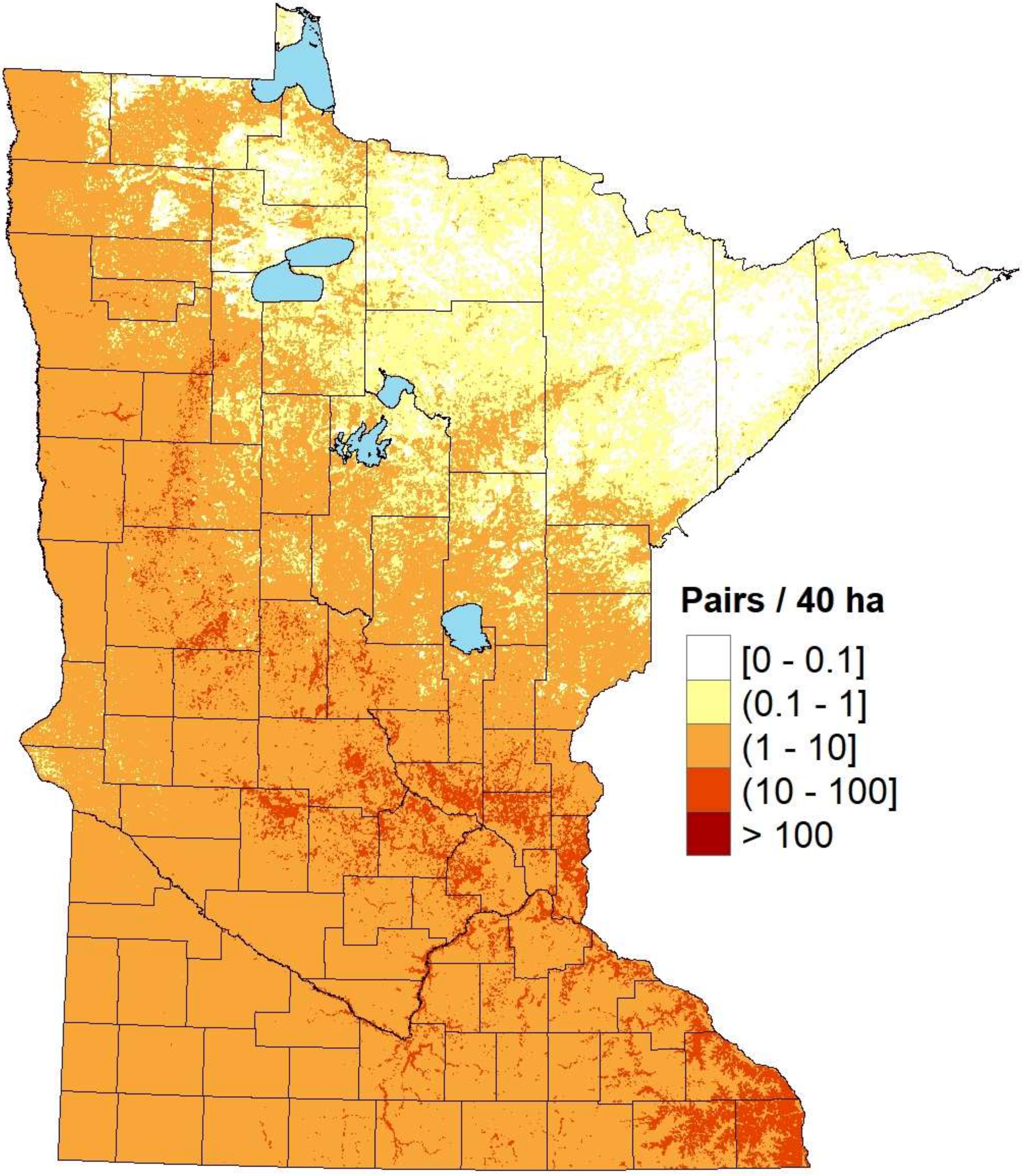

Least Flycatcher *Empidonax minimus*

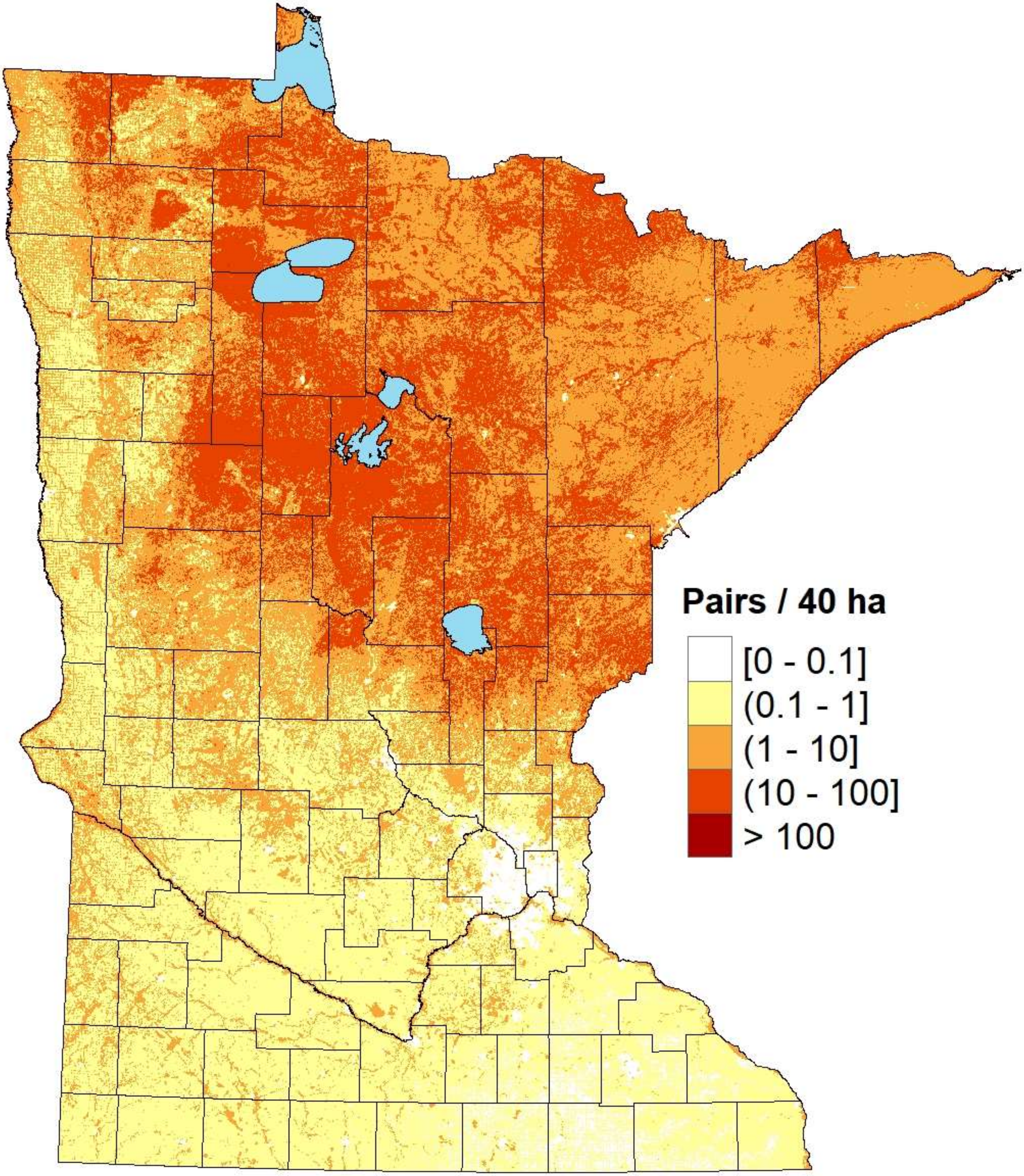

LeConte's Sparrow *Ammospiza leconteii*

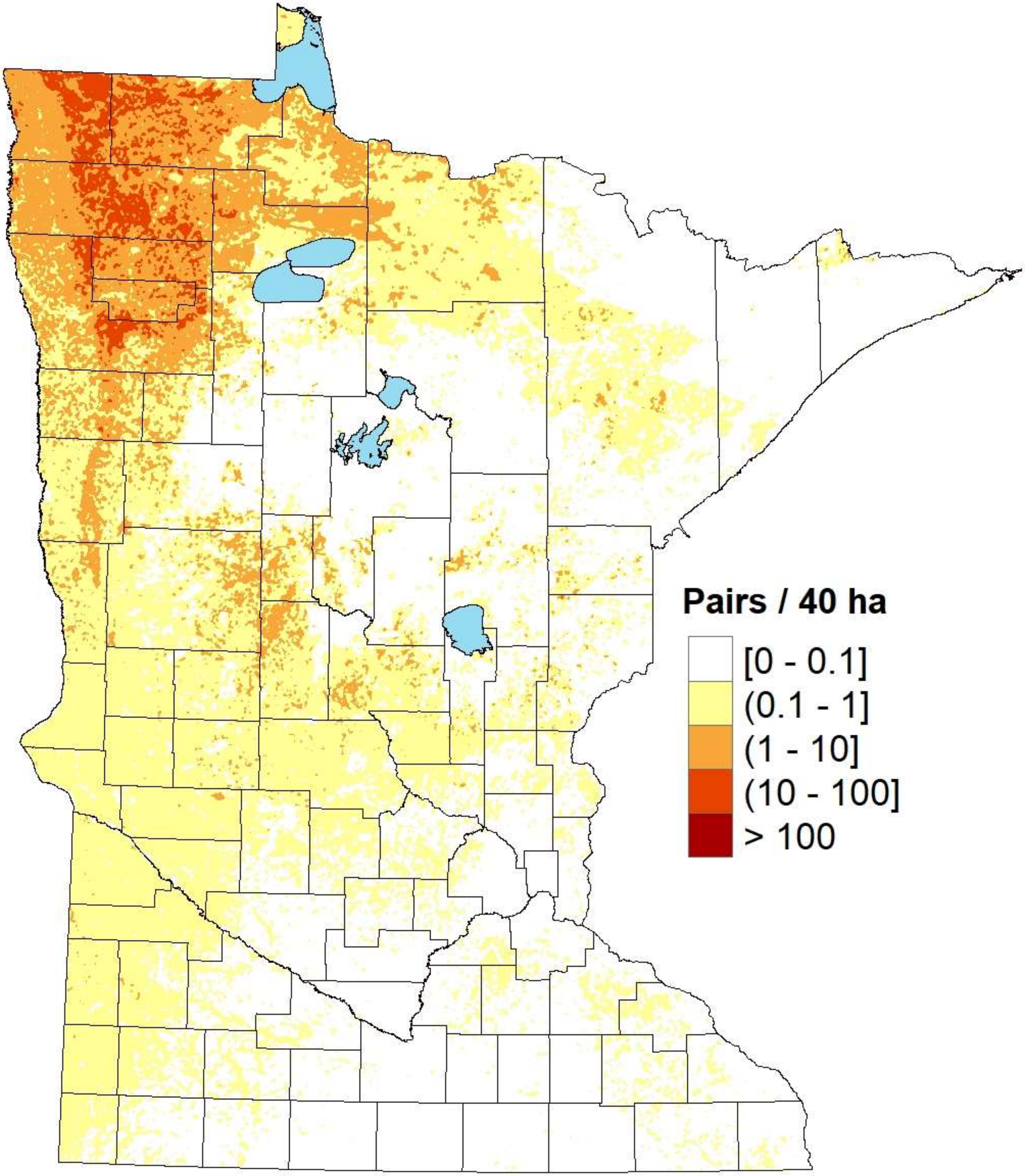

Lincoln's Sparrow *Melospiza lincolnii*

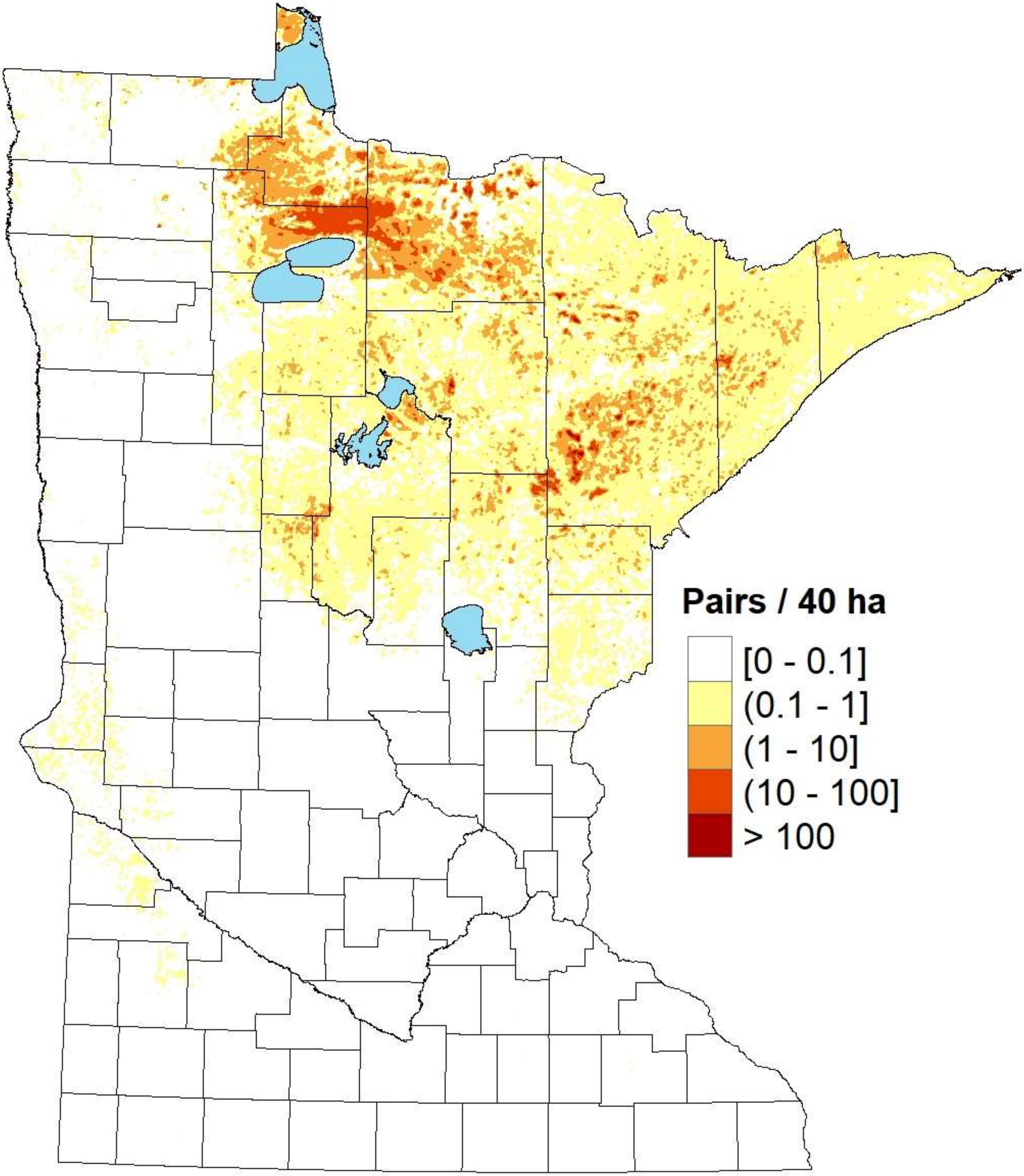

Magnolia Warbler *Setophaga magnolia*

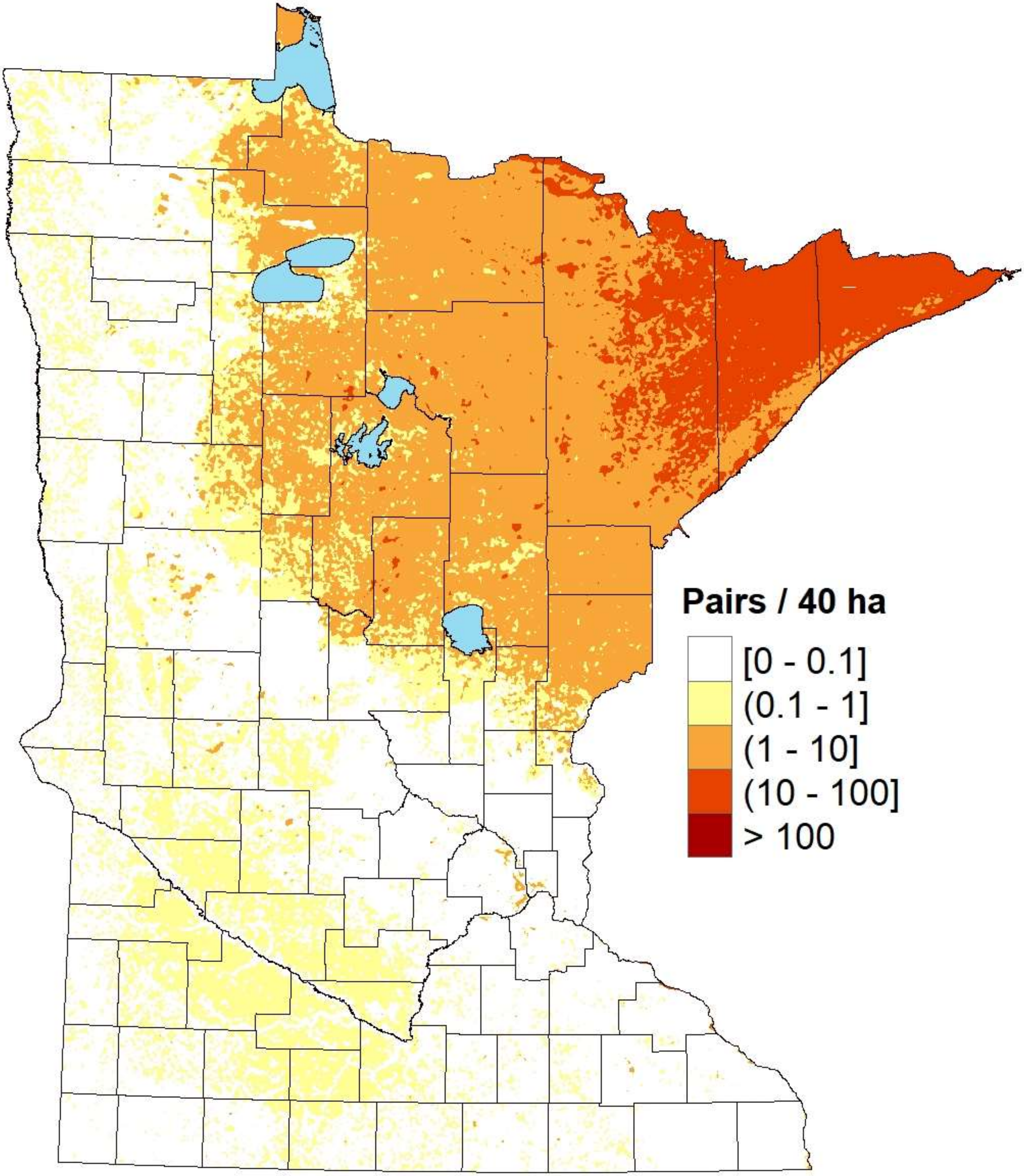

Marsh Wren *Cistothorus palustris*

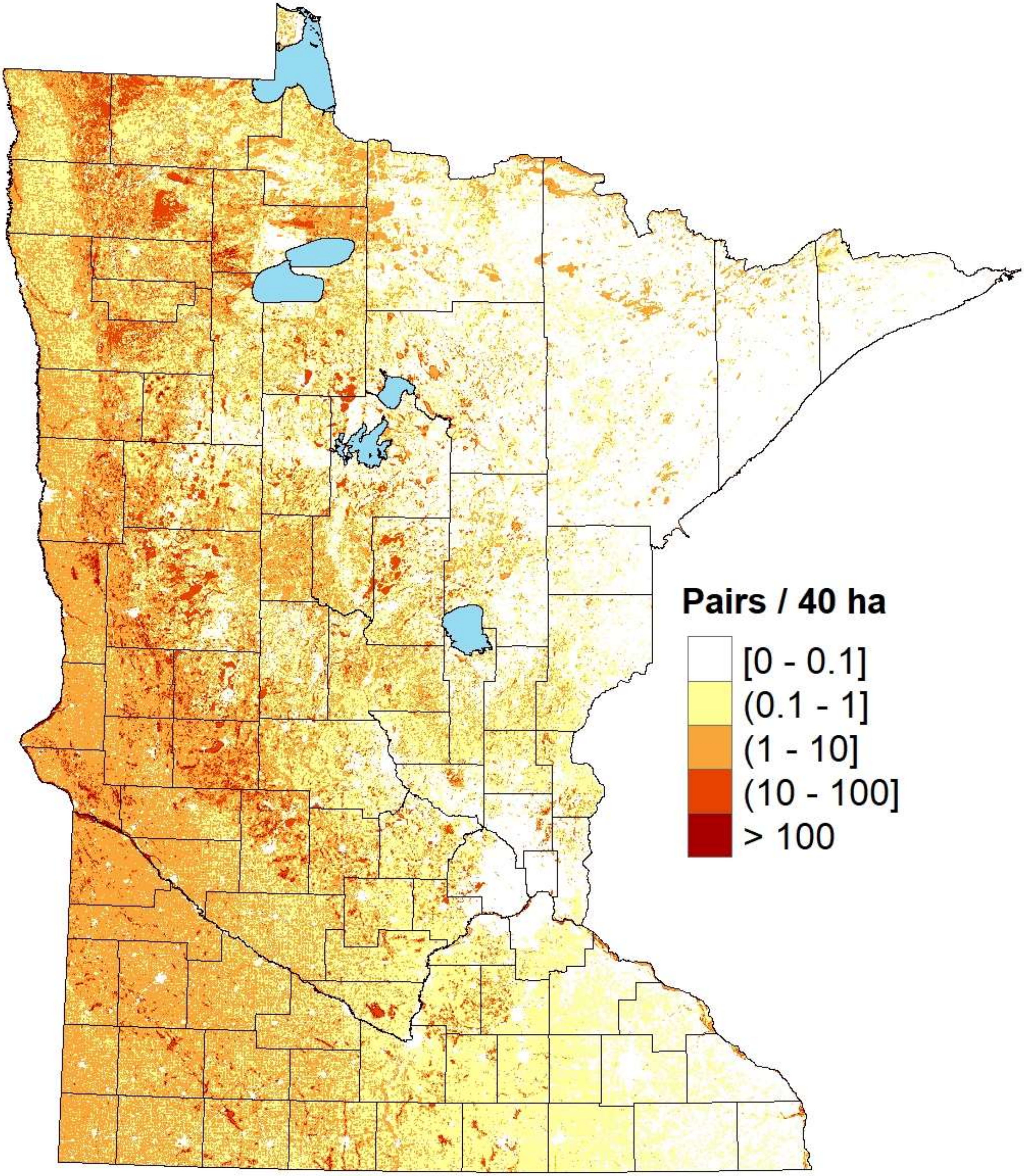

Mourning Warbler *Geothlypis philadelphia*

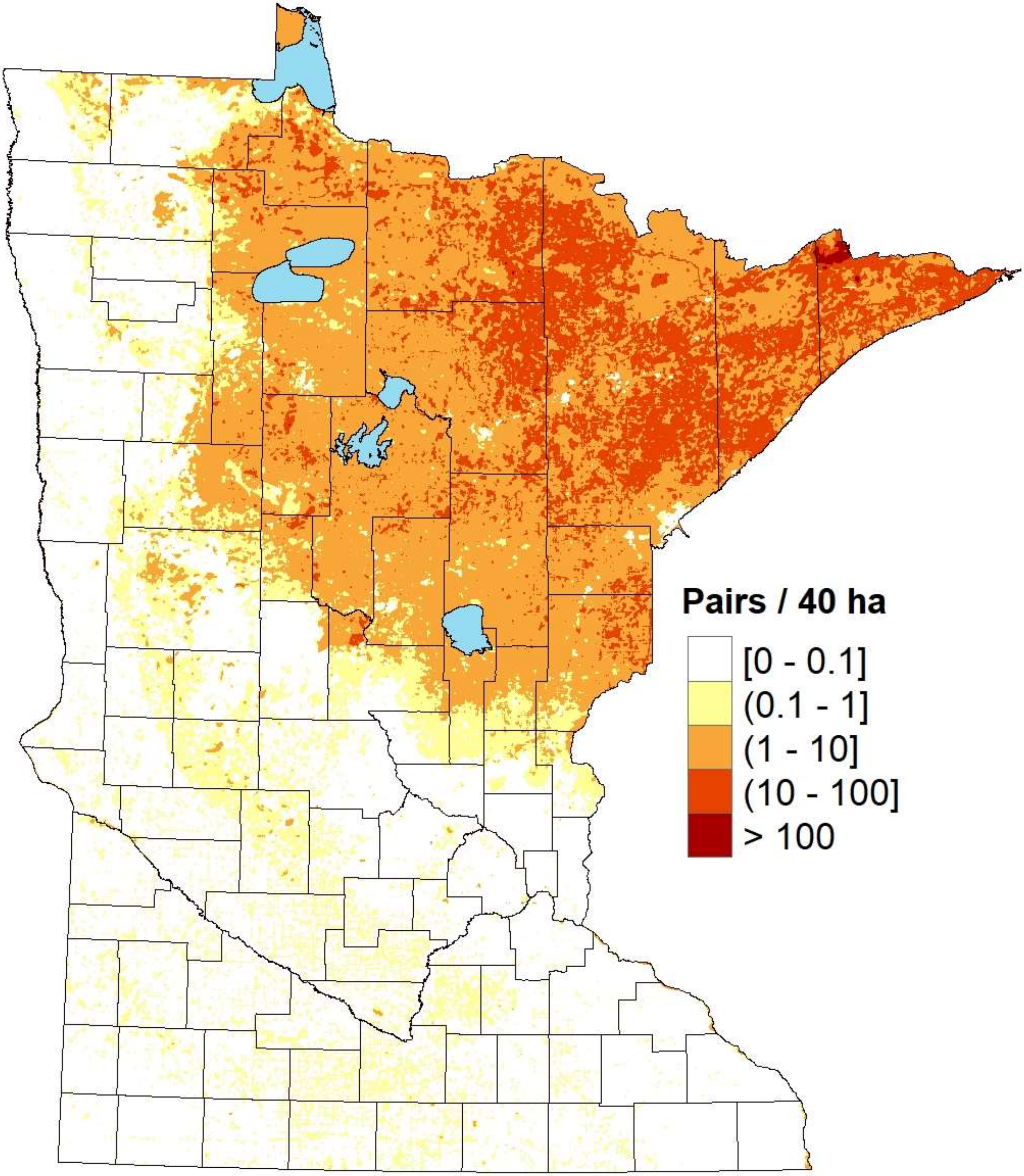

Nashville Warbler *Leiothlypis ruficapilla*

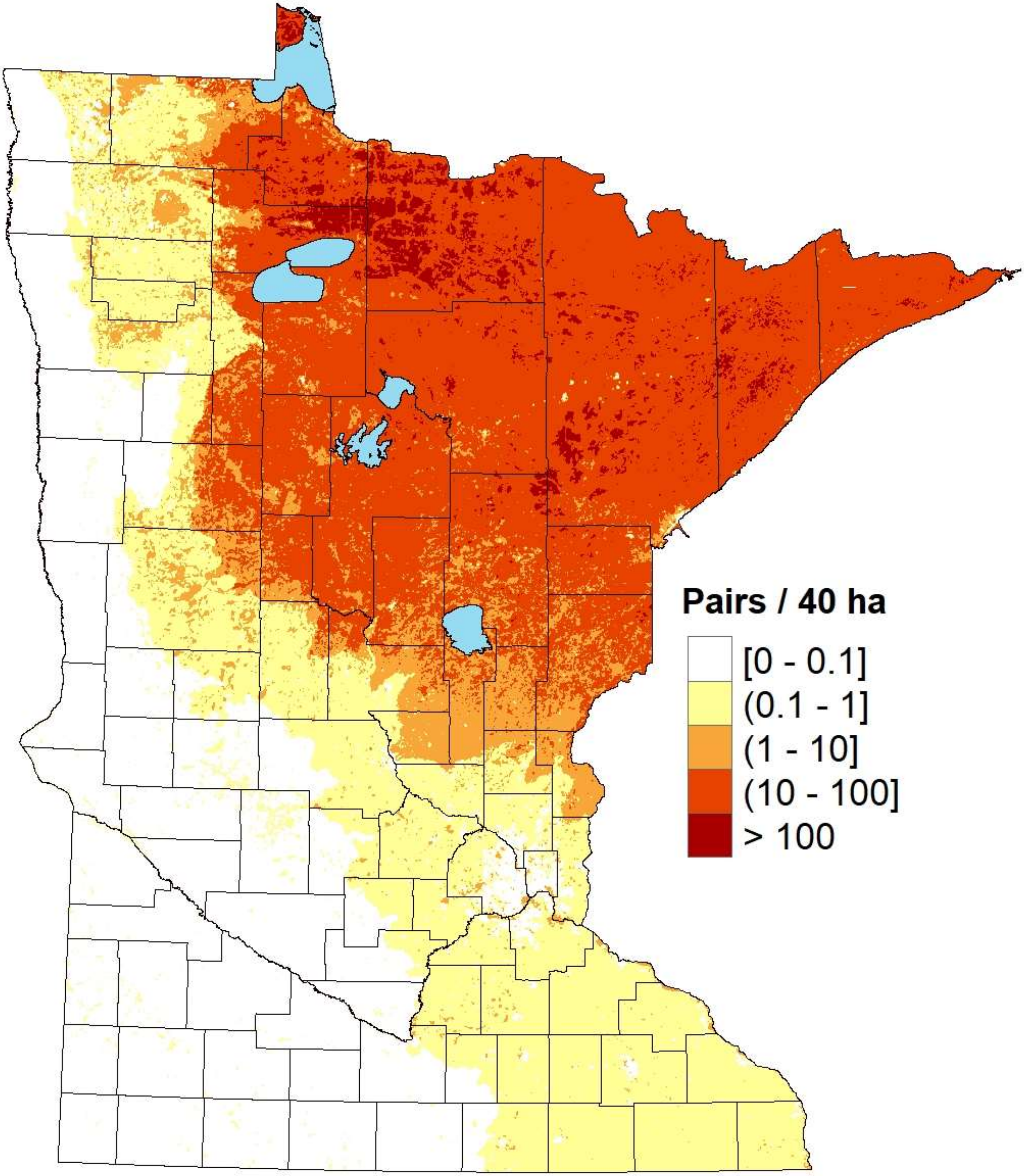

Northern Cardinal *Cardinalis cardinalis*

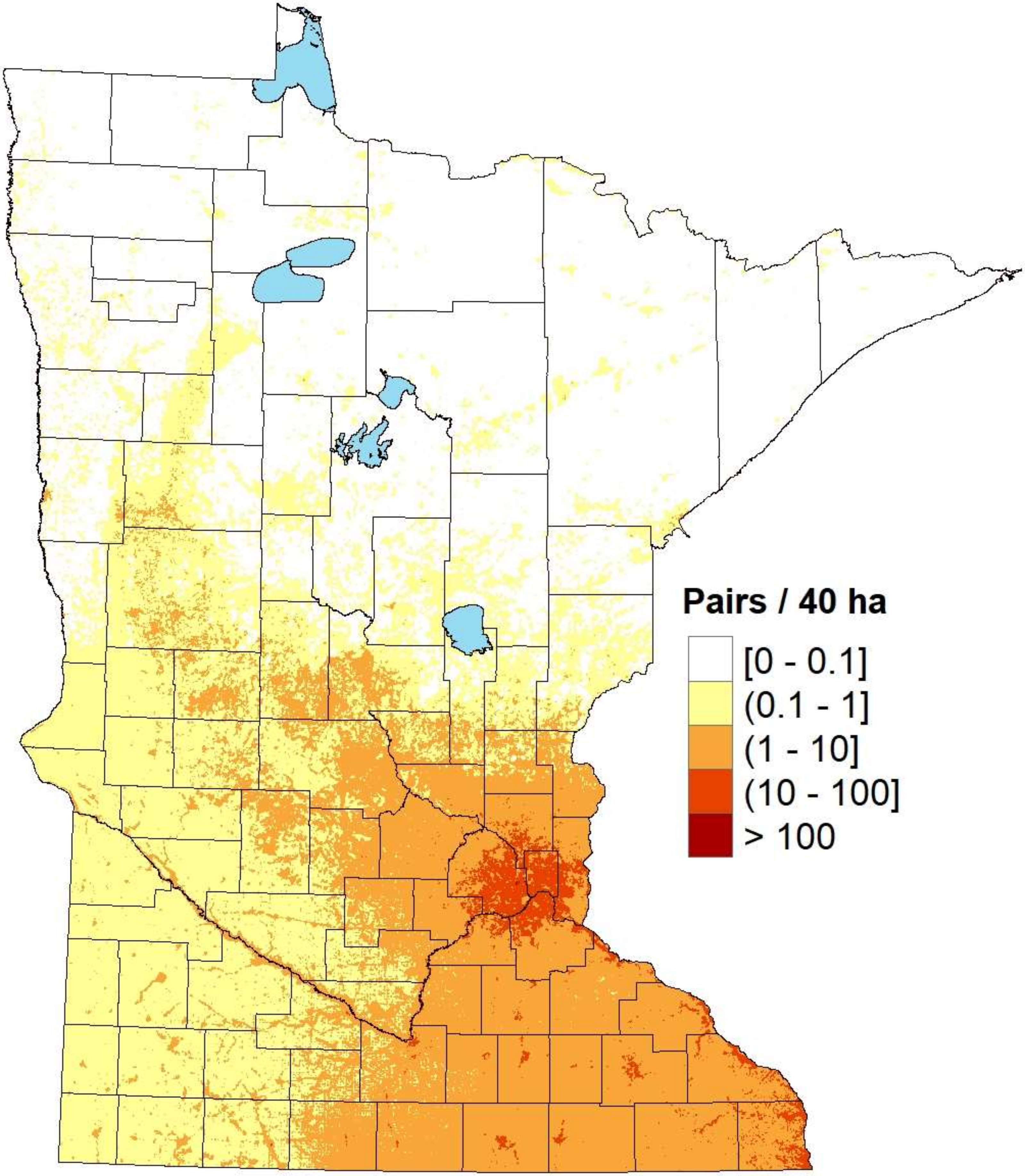

Northern Parula *Setophaga americana*

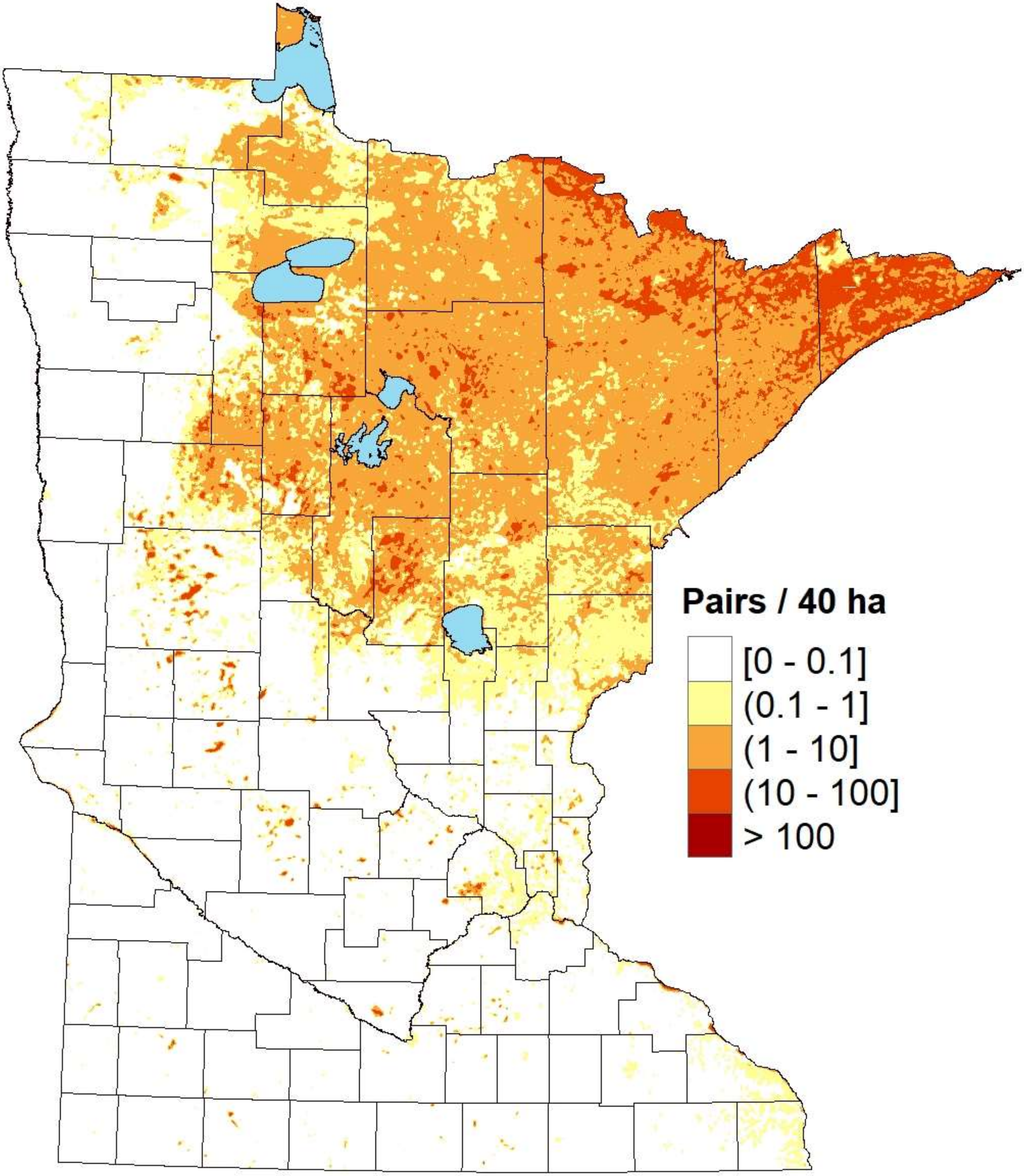

Northern Waterthrush *Parkesia noveboracensis*

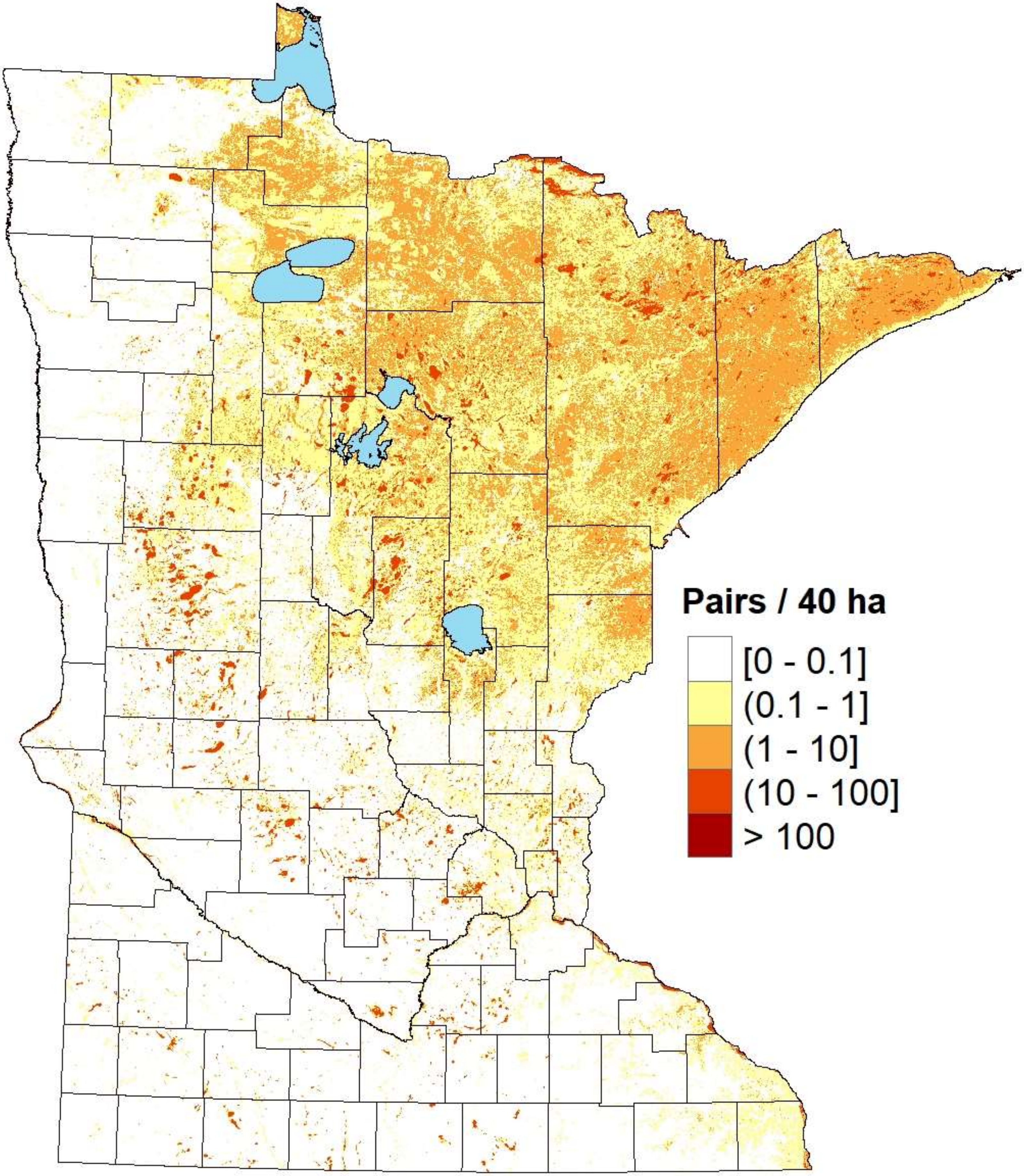

Olive-sided Flycatcher *Contopus cooperi*

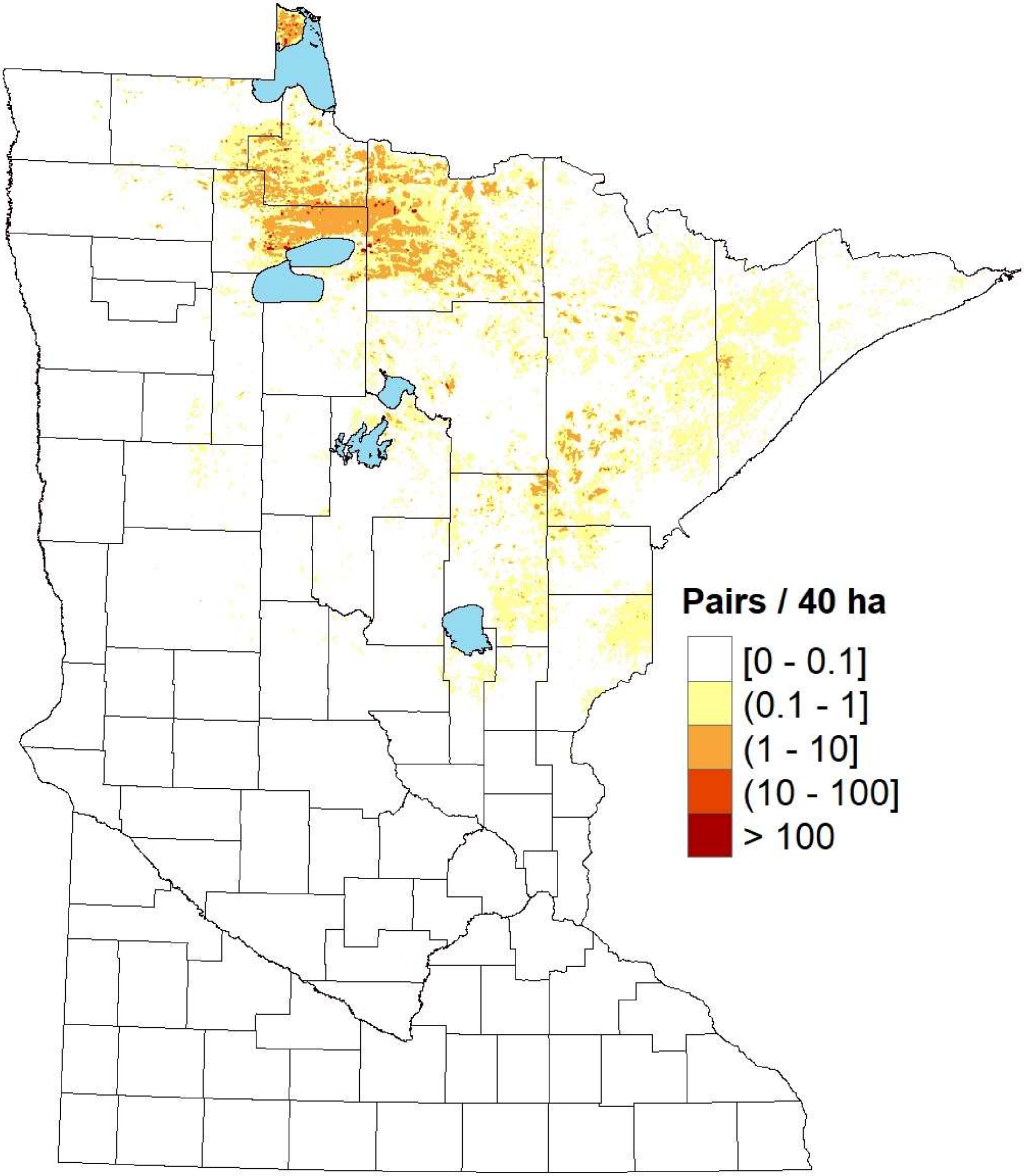

Ovenbird *Seiurus aurocapilla*

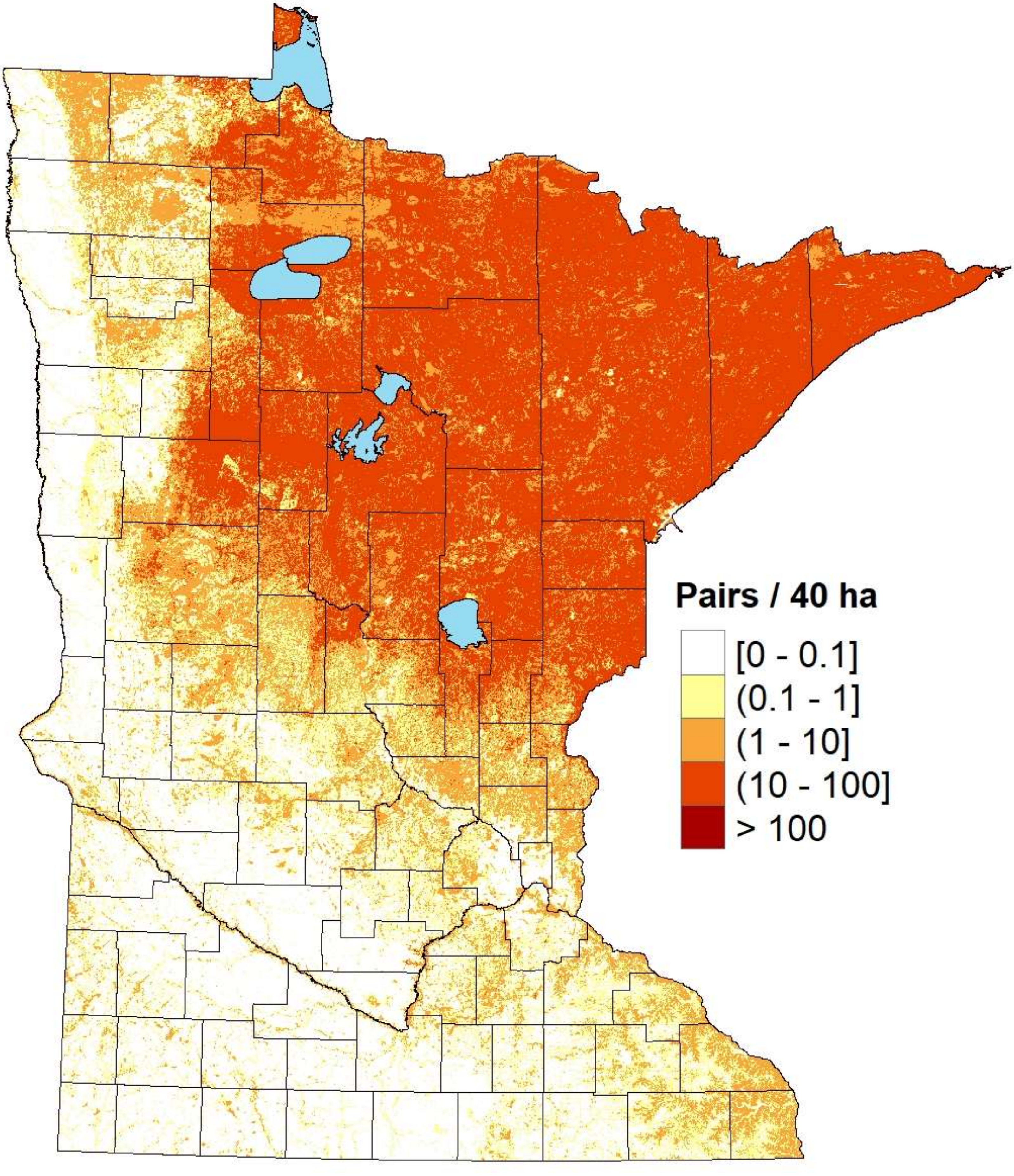

Palm Warbler *Setophaga palmarum*

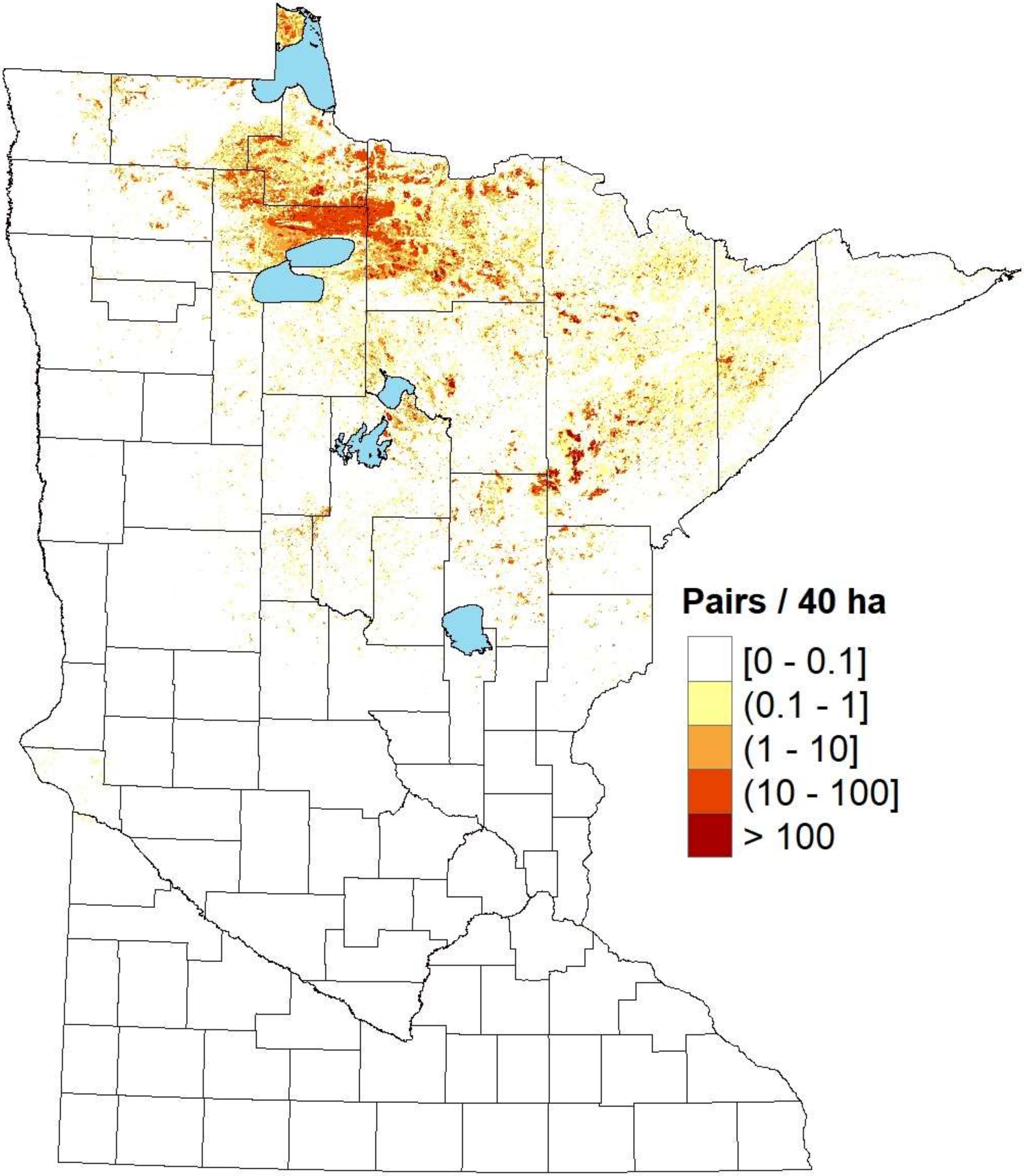

Pine Warbler *Setophaga pinus*

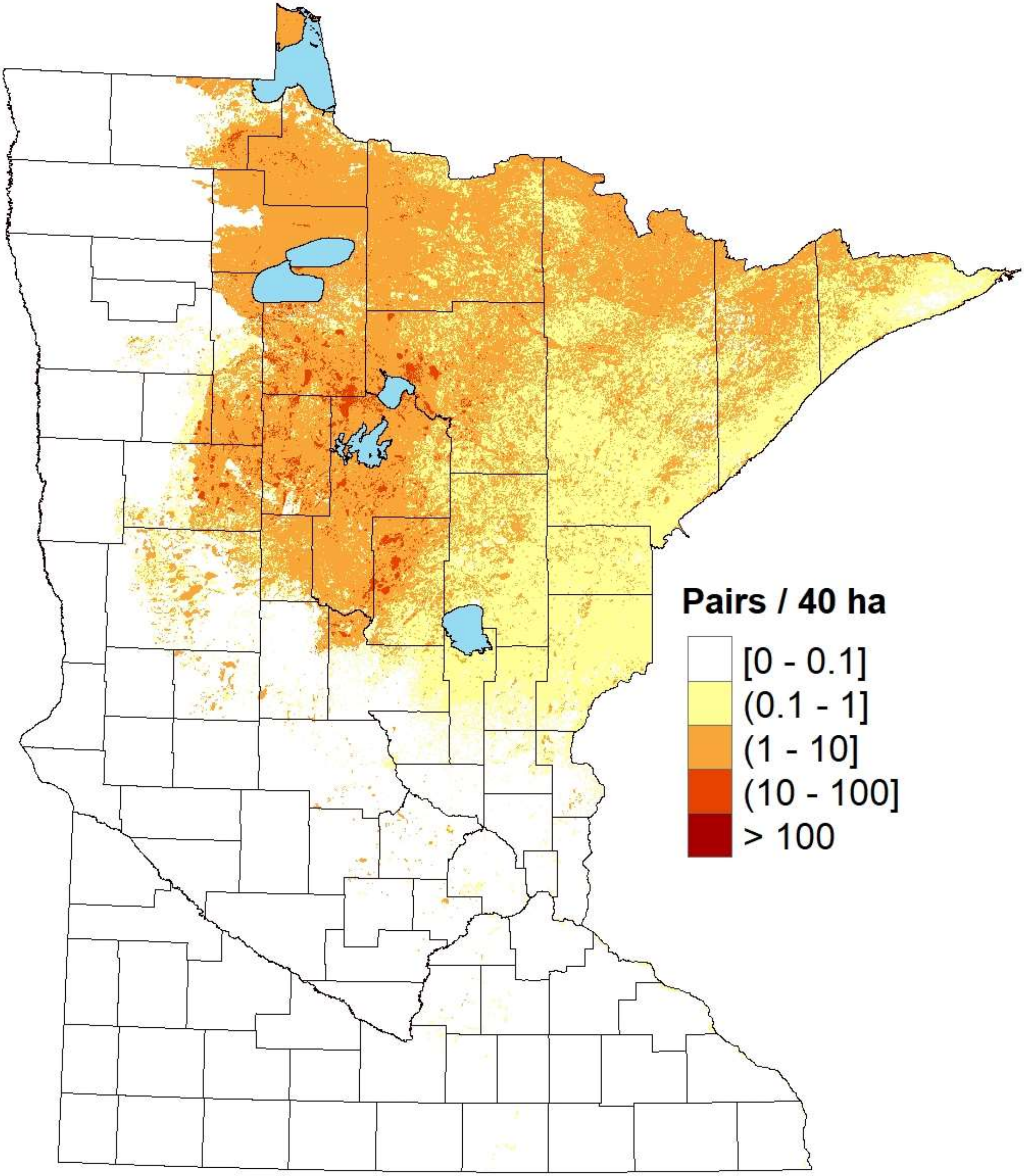

Purple Finch *Haemorhous purpureus*

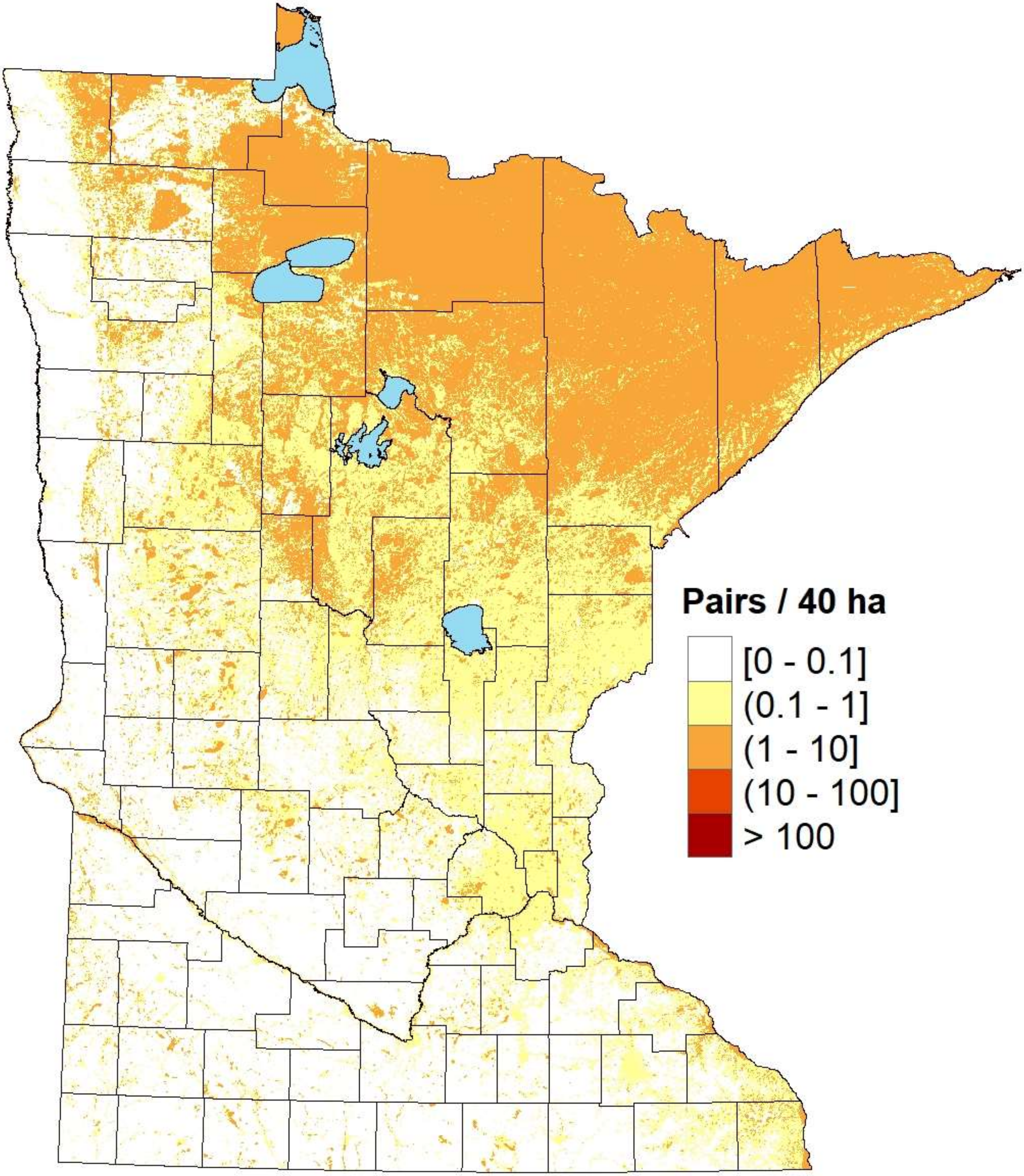

Red-eyed Vireo *Vireo olivaceus*

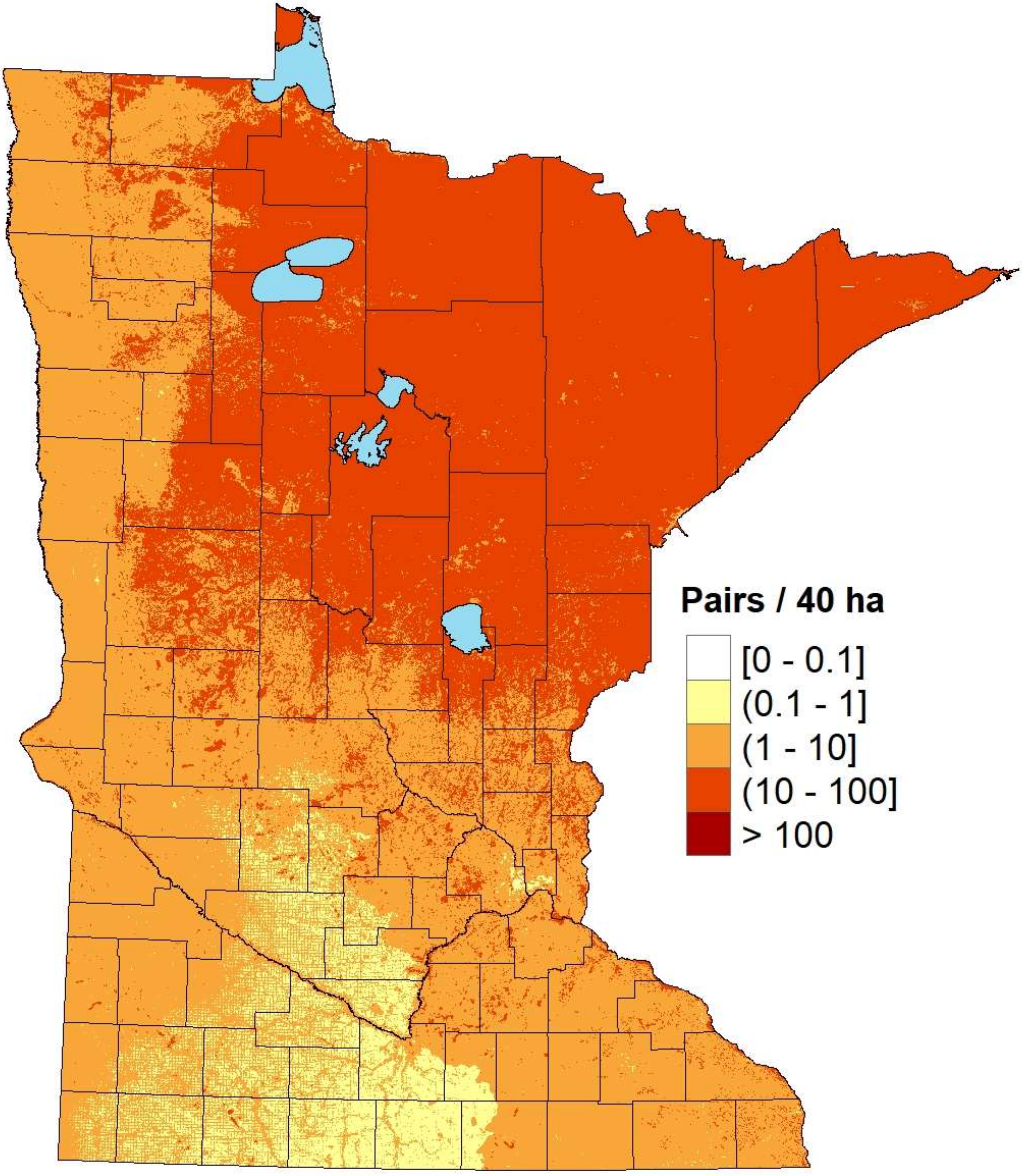

Red-winged Blackbird *Agelaius phoeniceus*

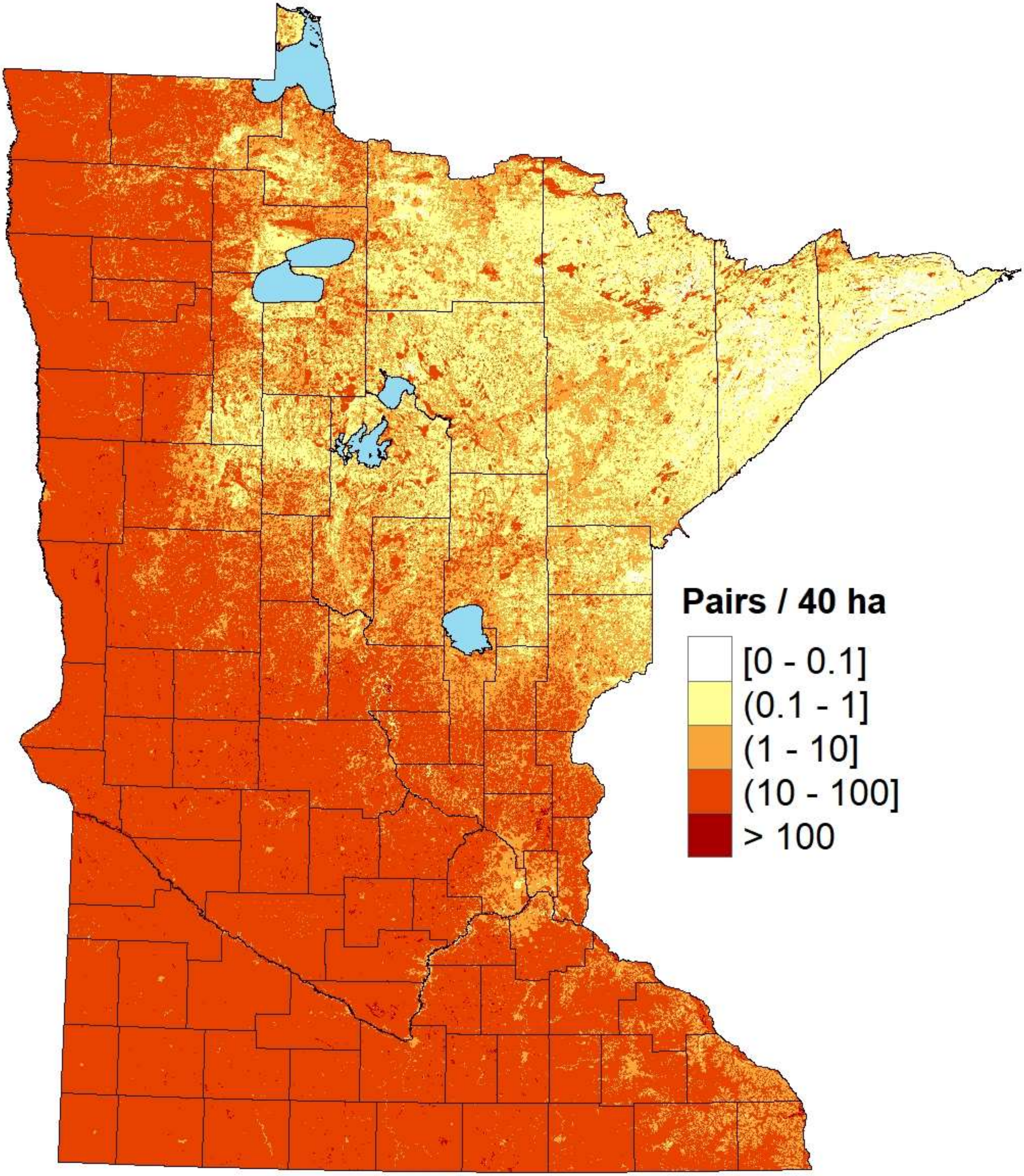

Ruby-crowned Kinglet *Regulus calendula*

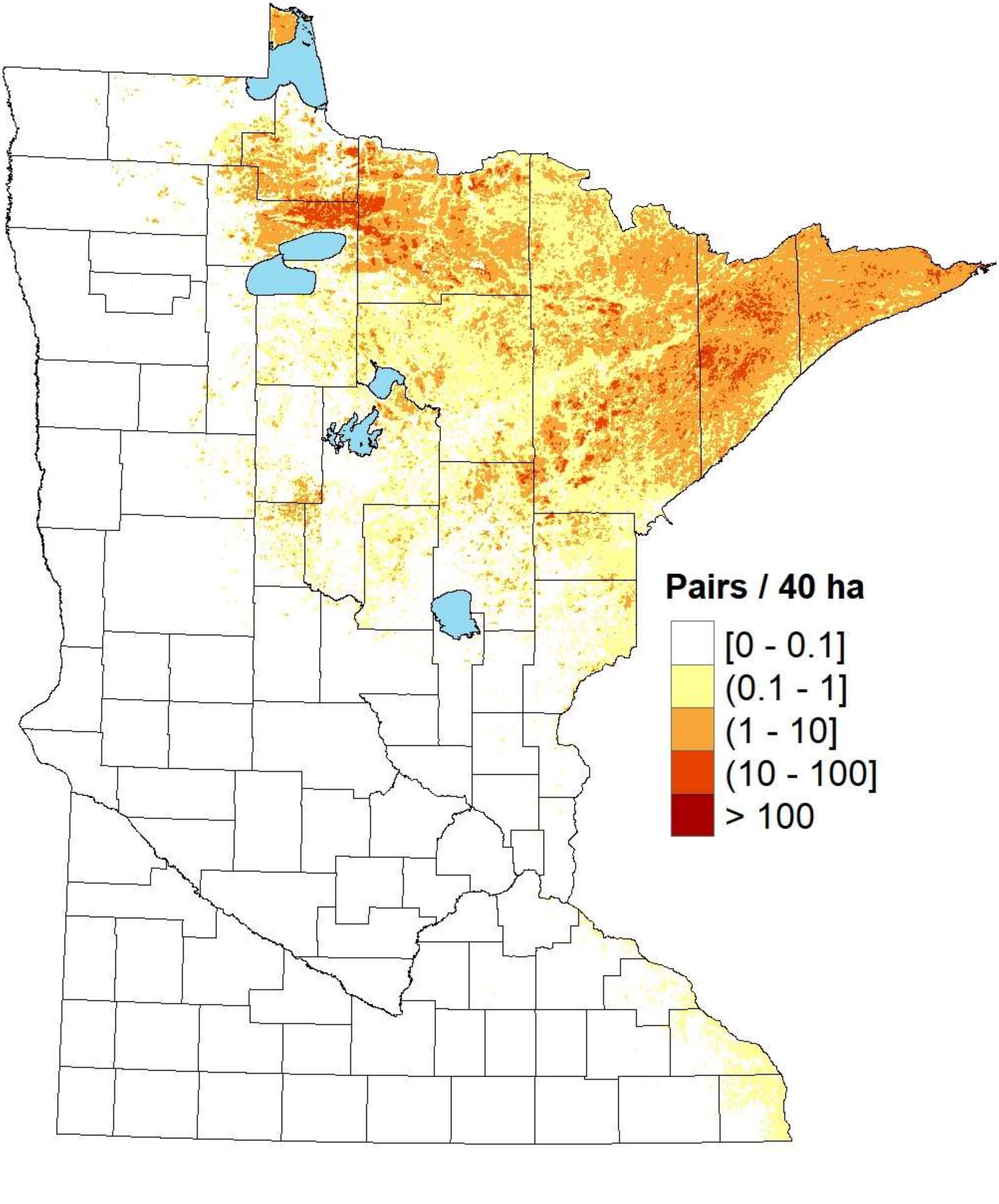

Savannah Sparrow *Passerculus sandwichensis*

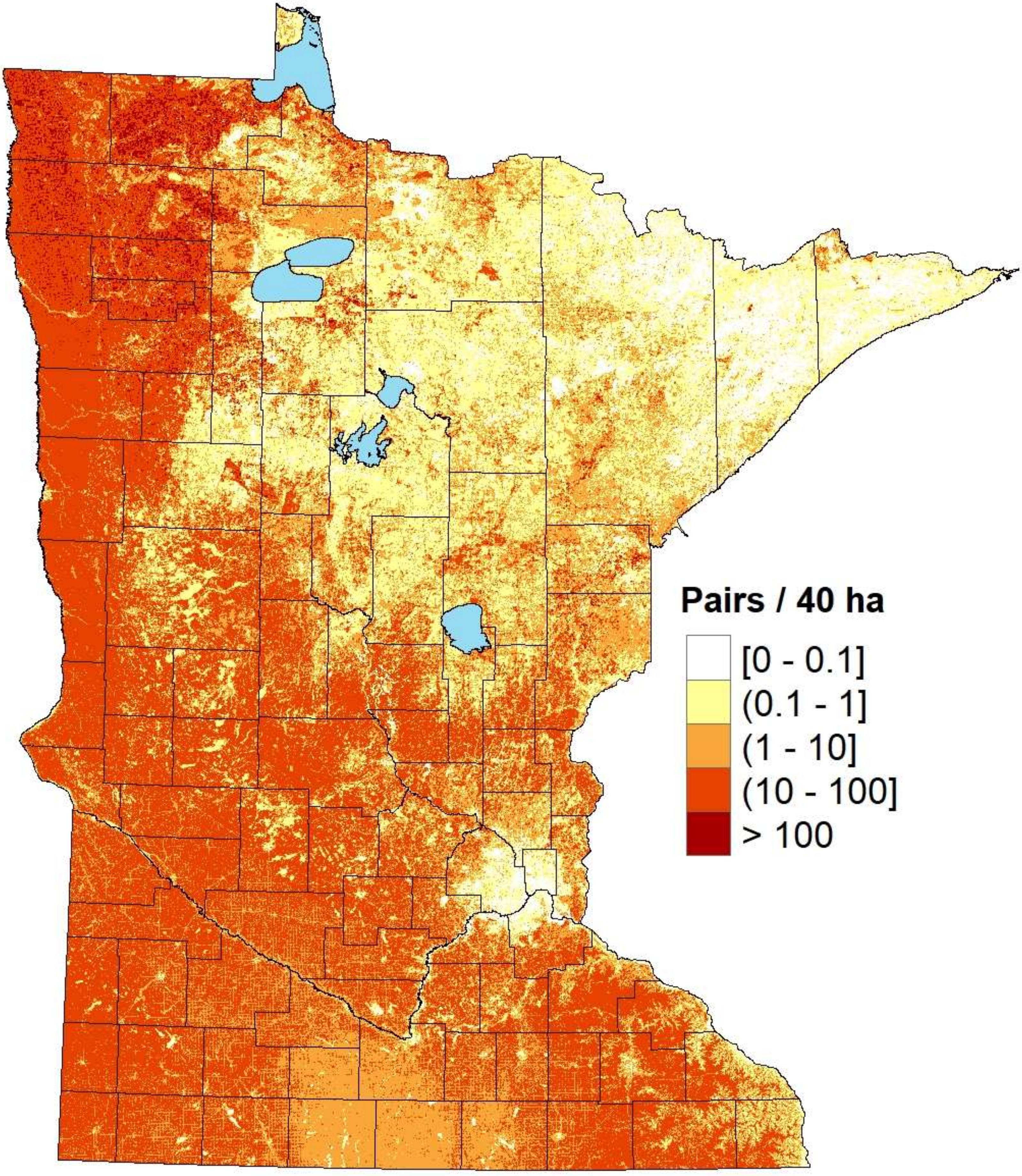

Scarlet Tanager *Piranga olivacea*

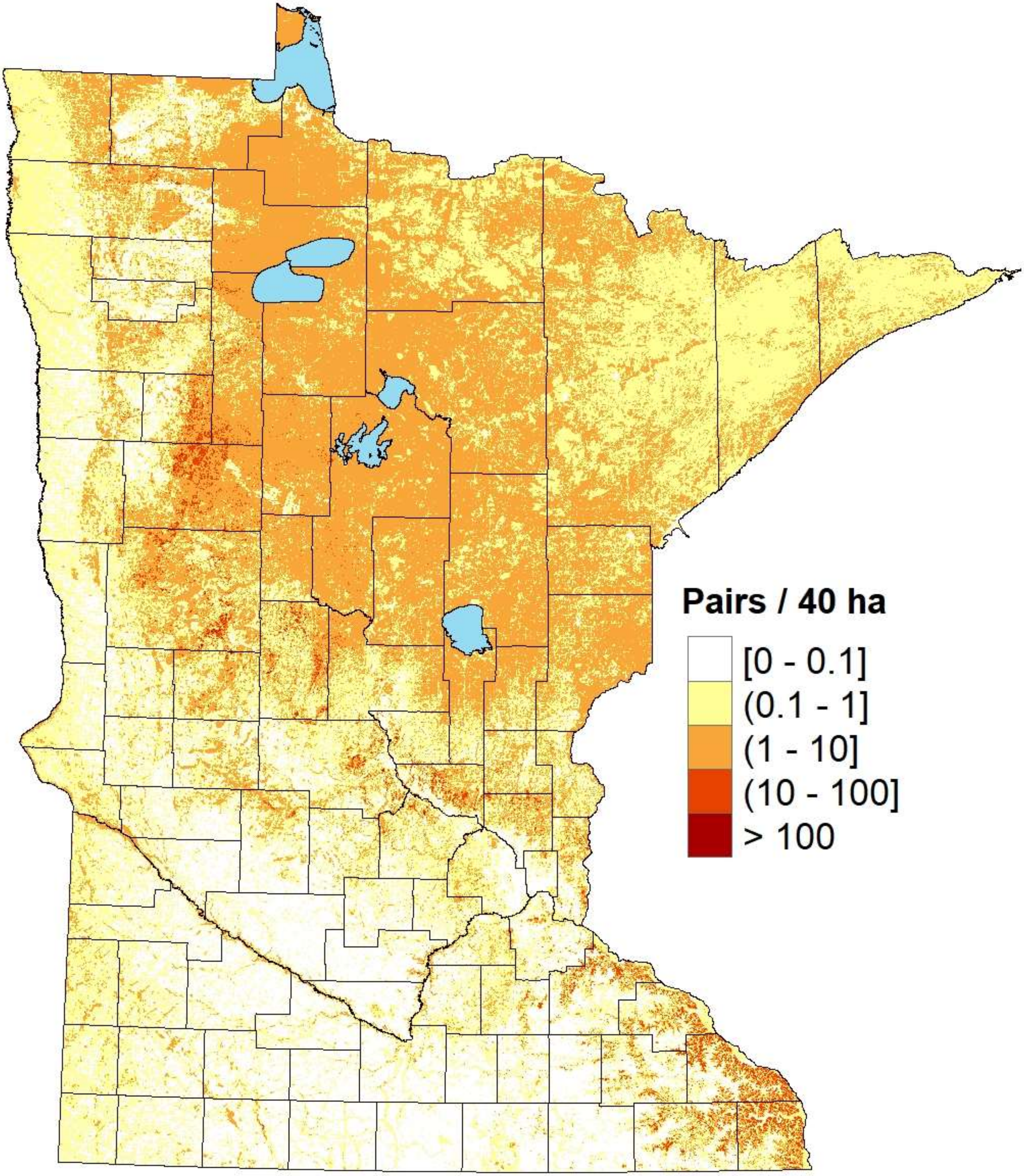

Sedge Wren *Cistothorus platensis*

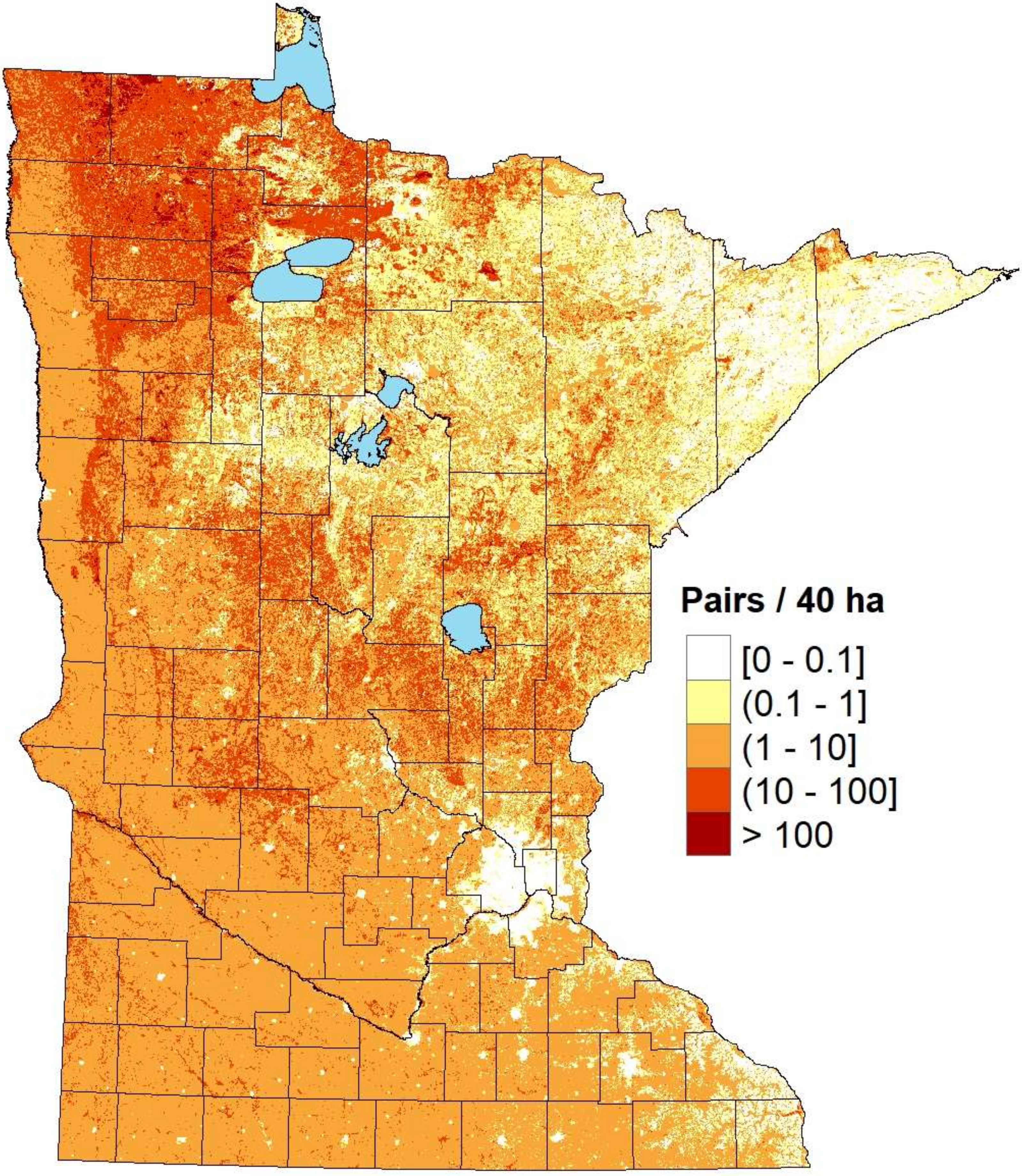

Song Sparrow *Melospiza melodia*

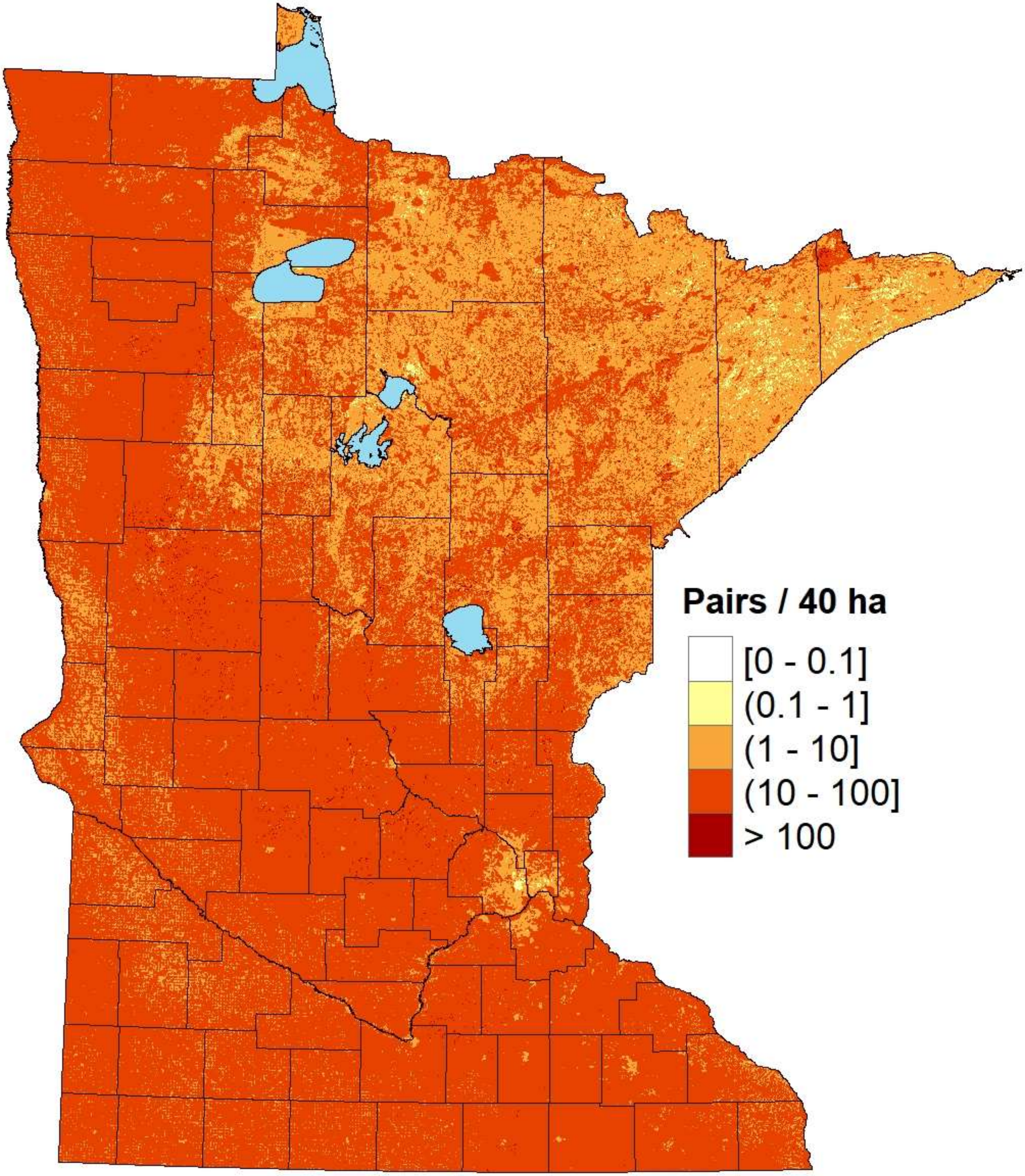

Swainson's Thrush *Catharus ustulatus*

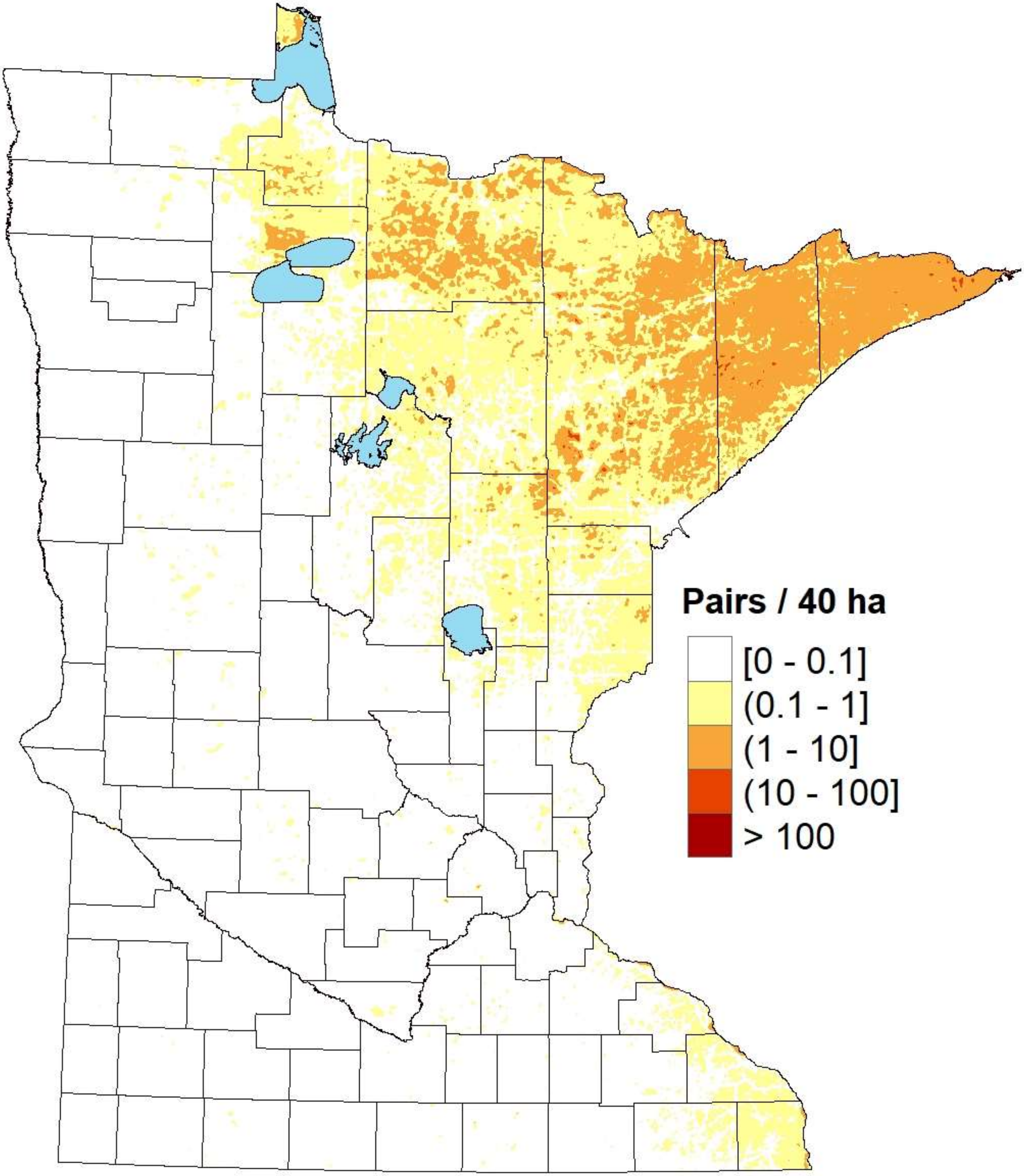

Swamp Sparrow *Melospiza georgiana*

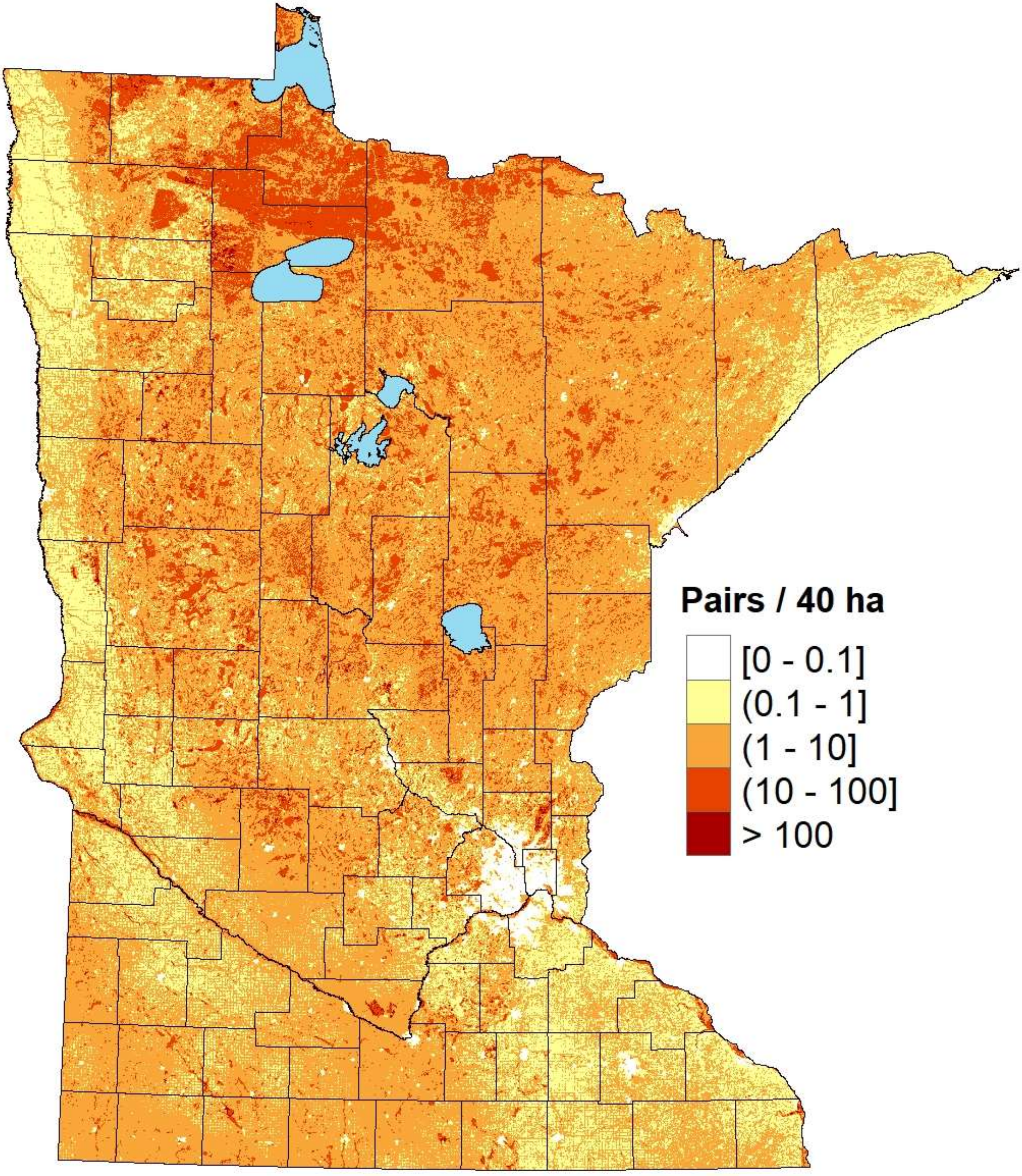

Tennessee Warbler *Leiothlypis peregrina*

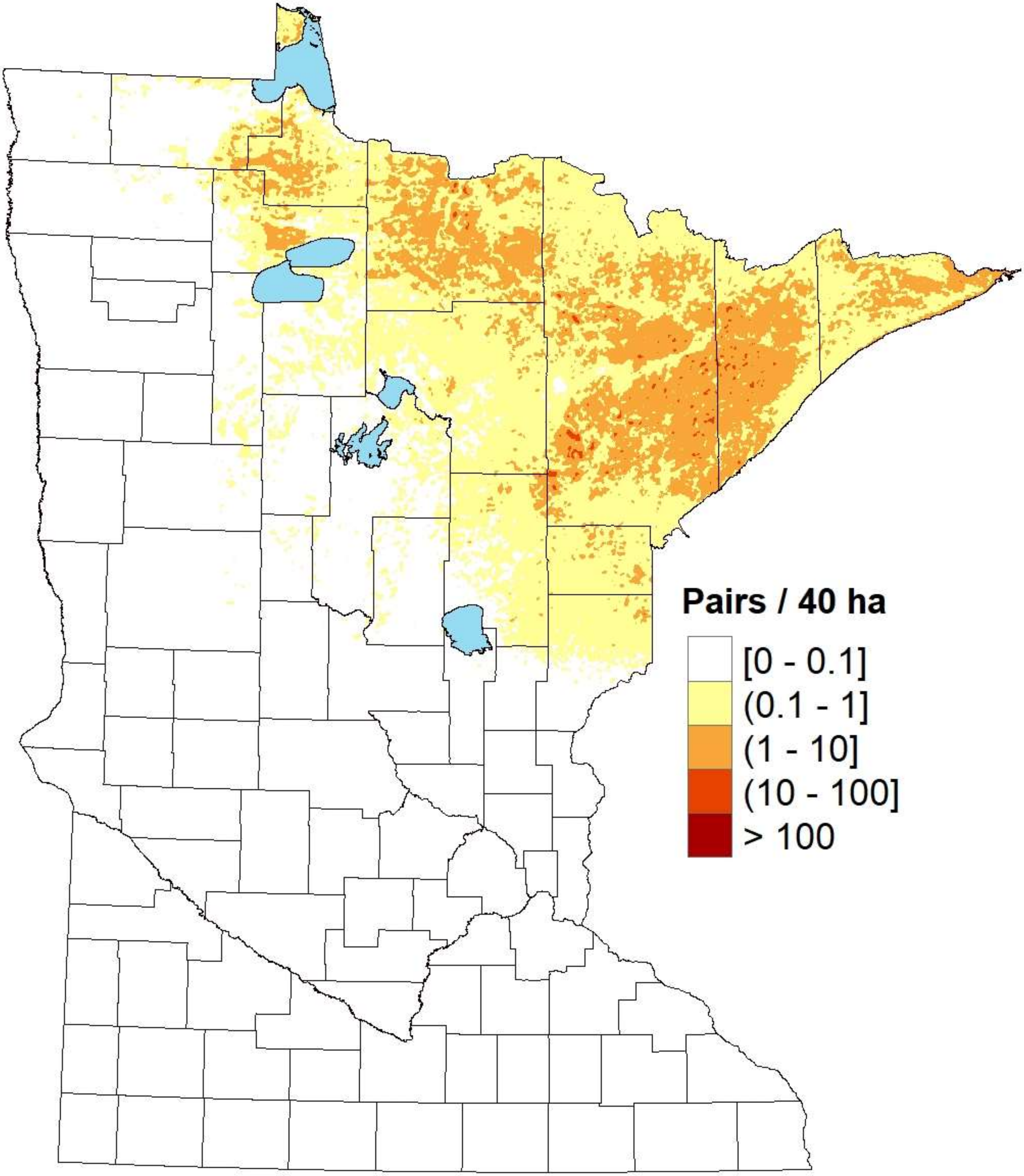

Veery *Catharus fuscescens*

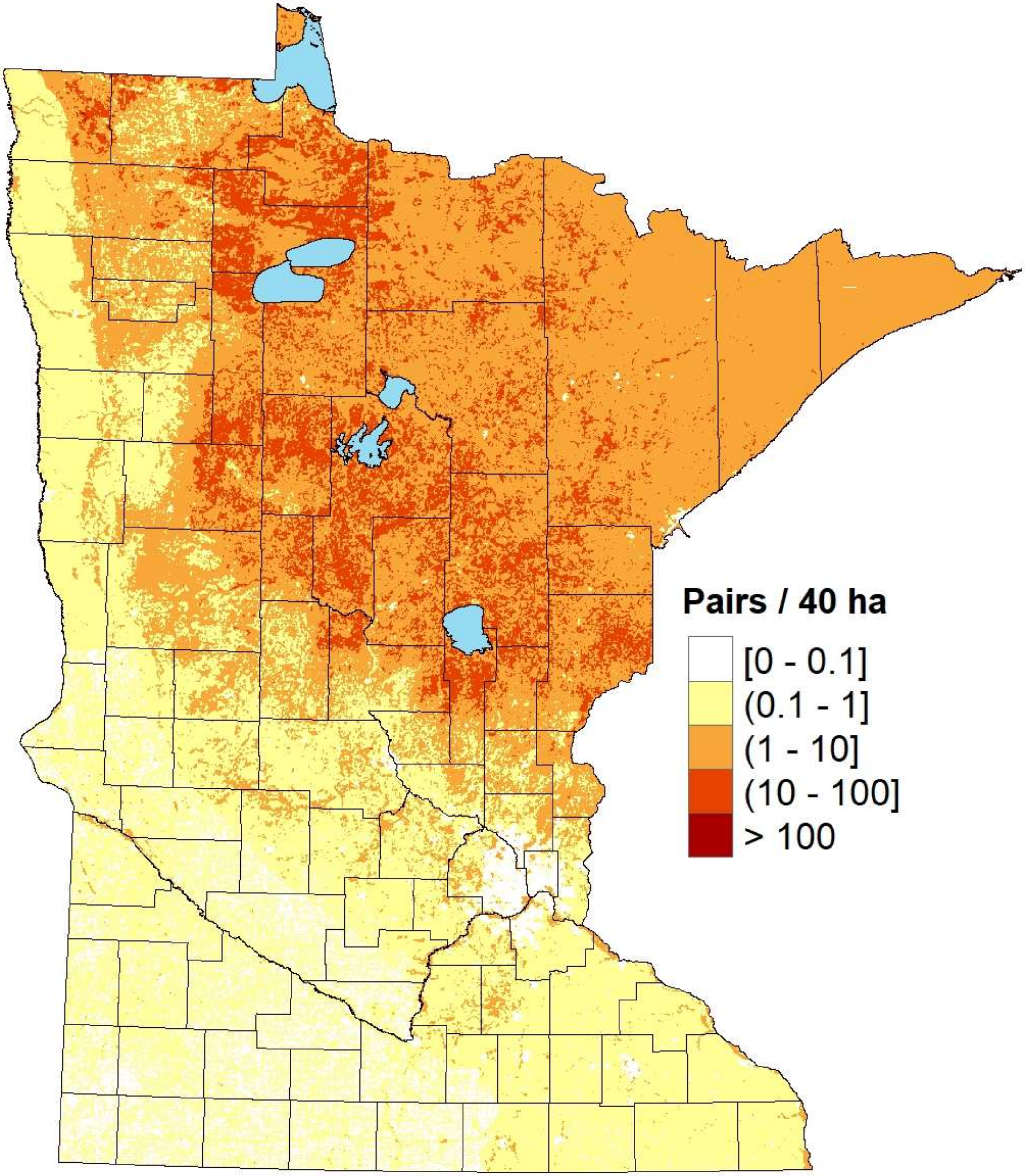

Vesper Sparrow *Pooecetes gramineus*

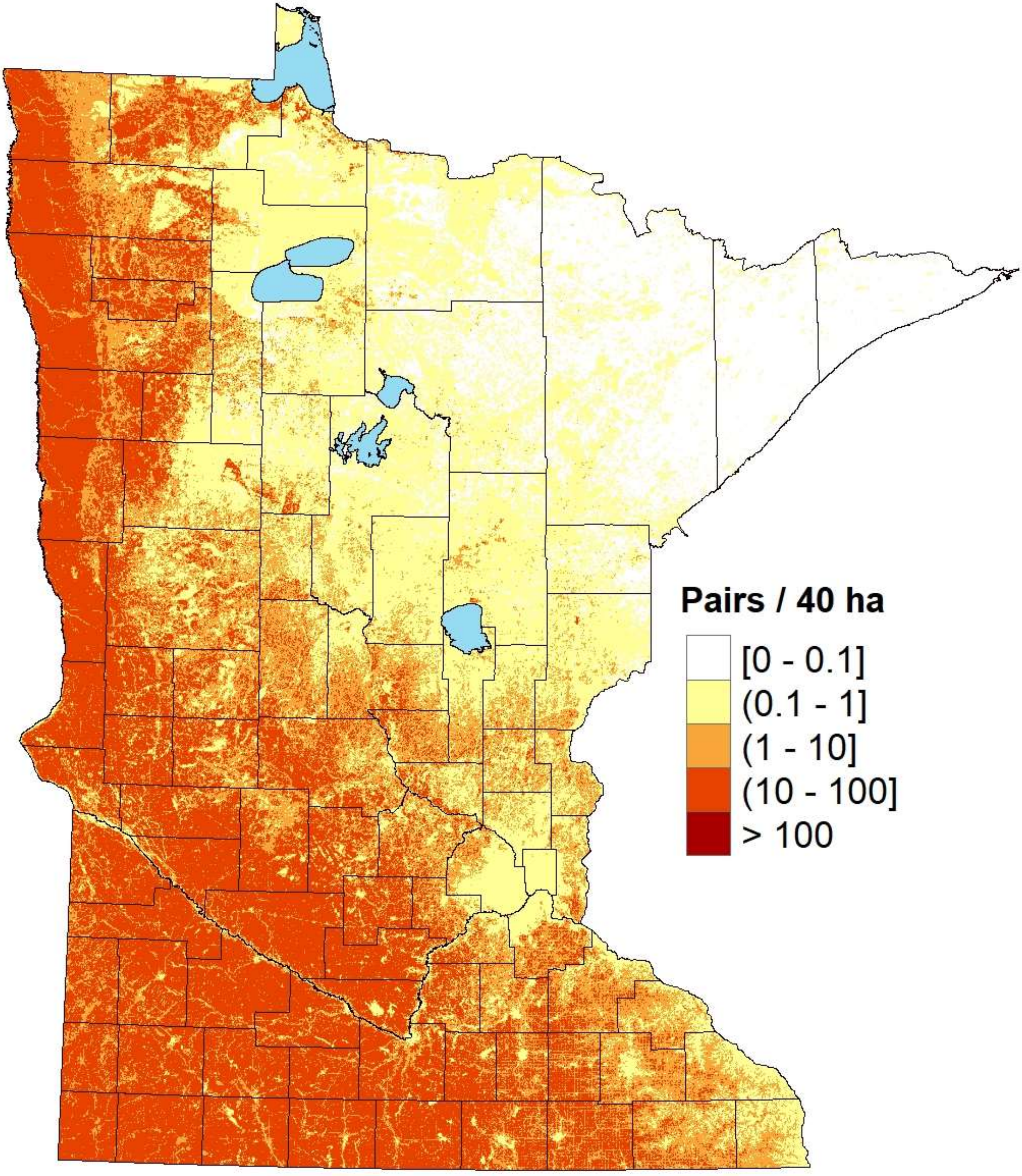

Warbling Vireo *Vireo gilvus*

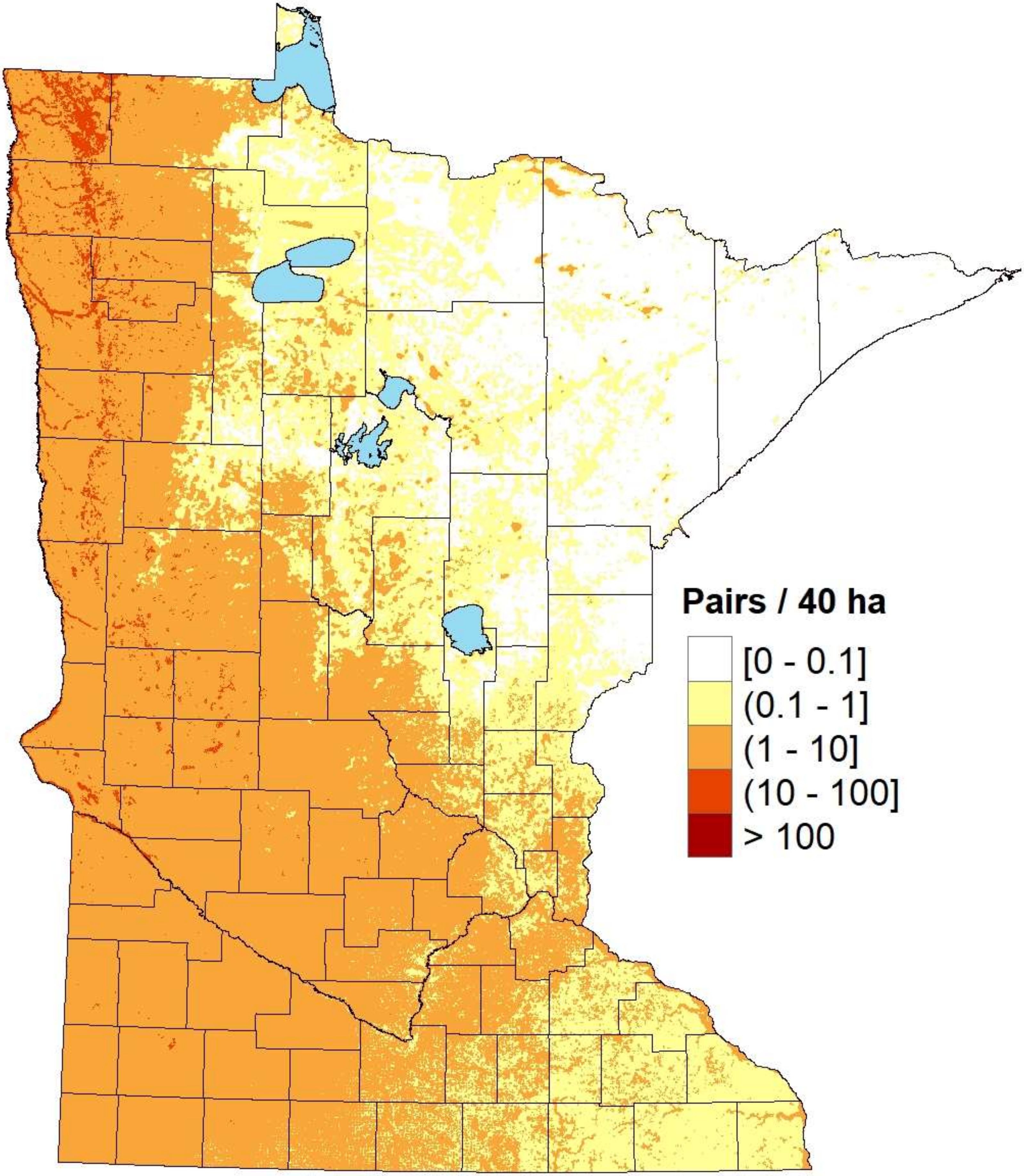

Western Meadowlark *Sturnella neglecta*

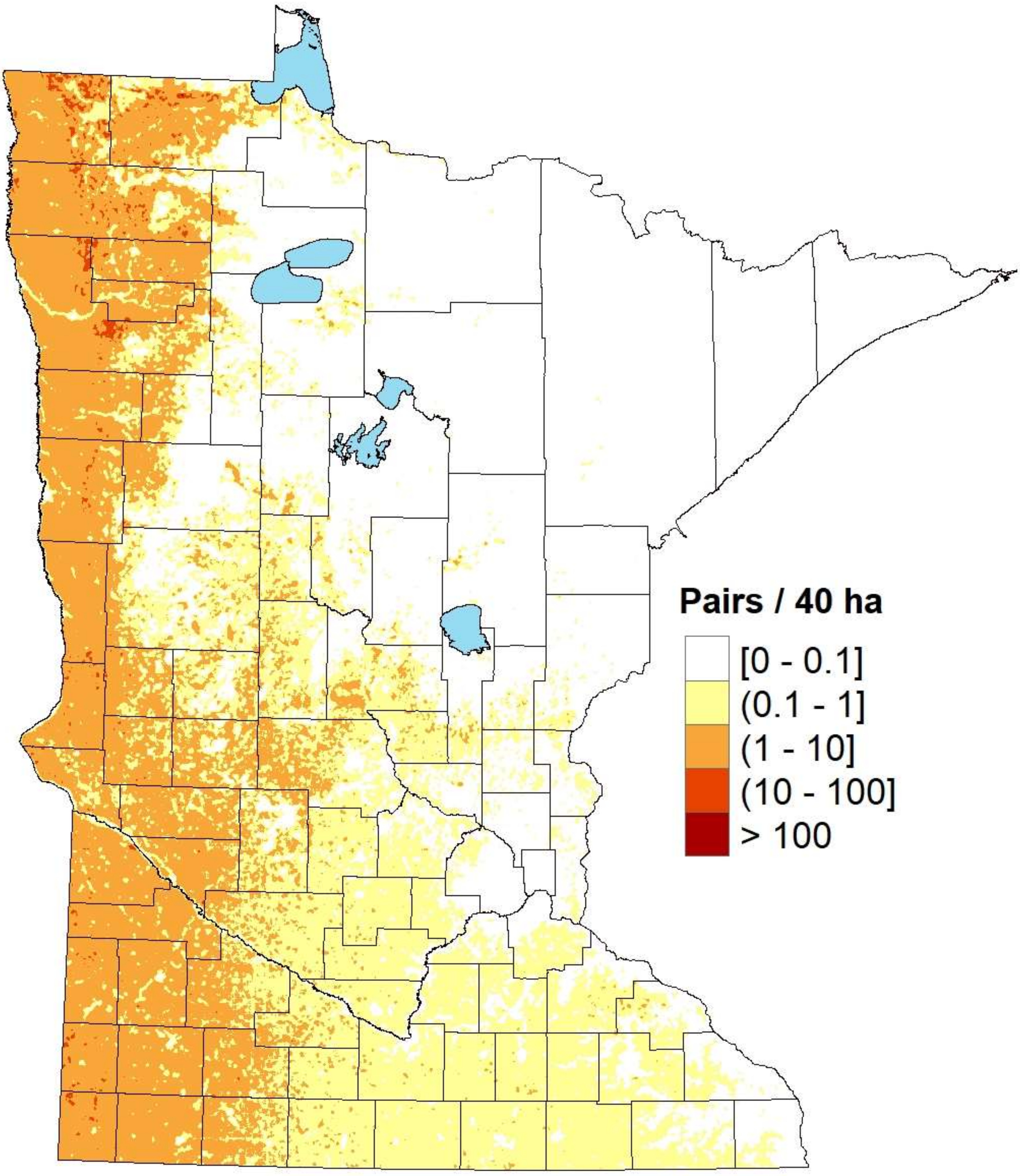

White-throated Sparrow *Zonotrichia albicollis*

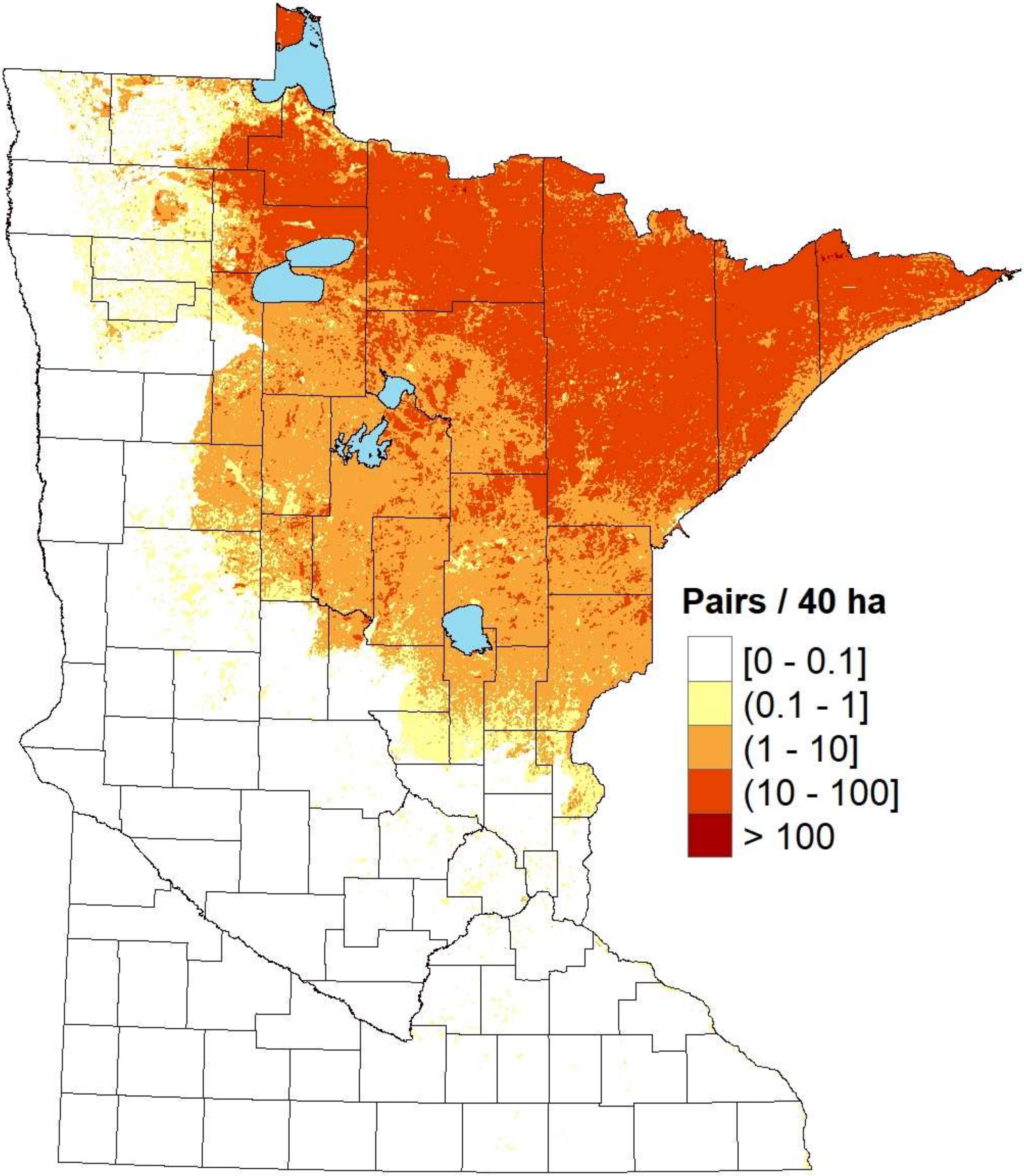

Winter Wren *Troglodytes hiemalis*

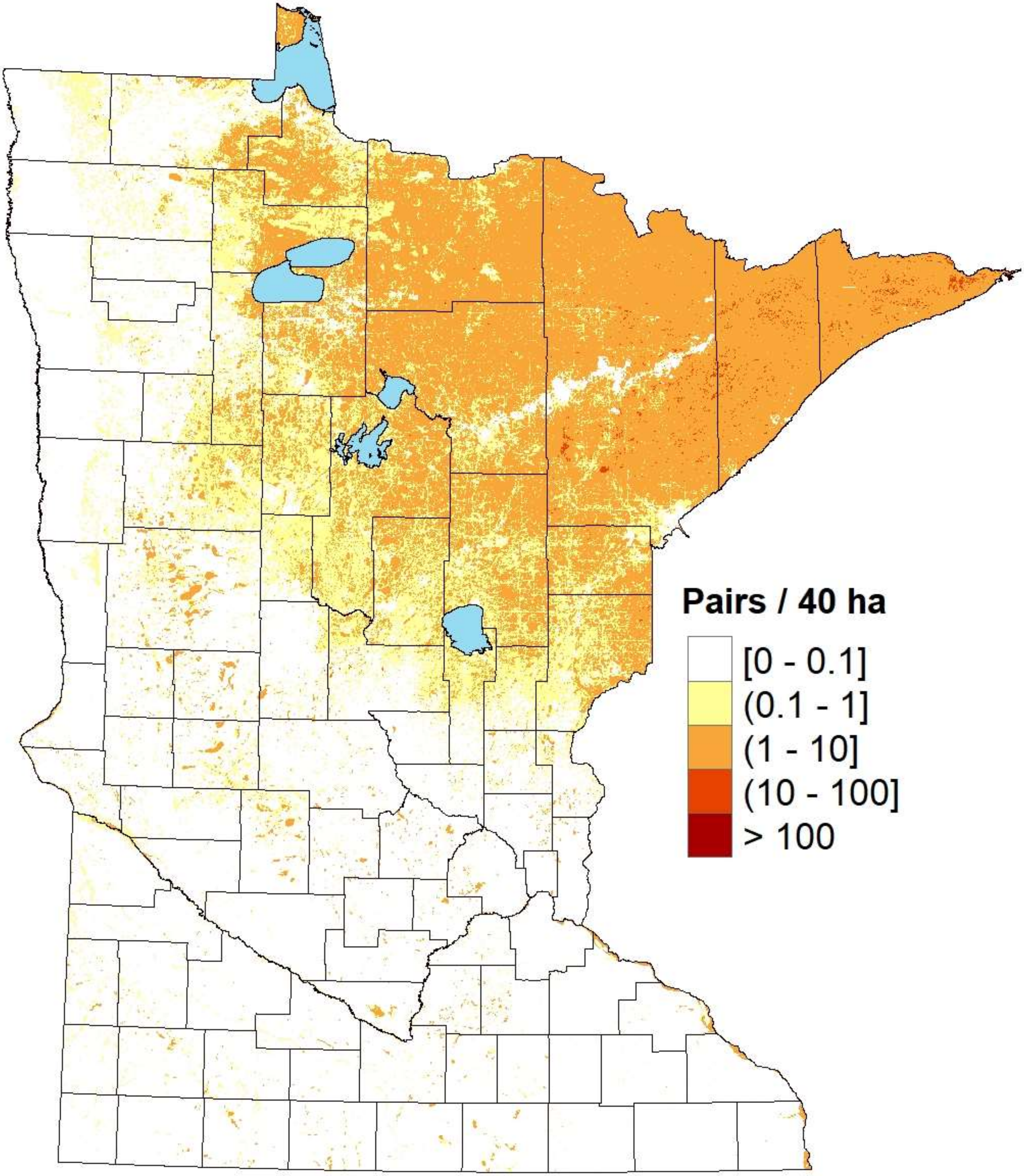

Yellow Warbler *Setophaga petechia*

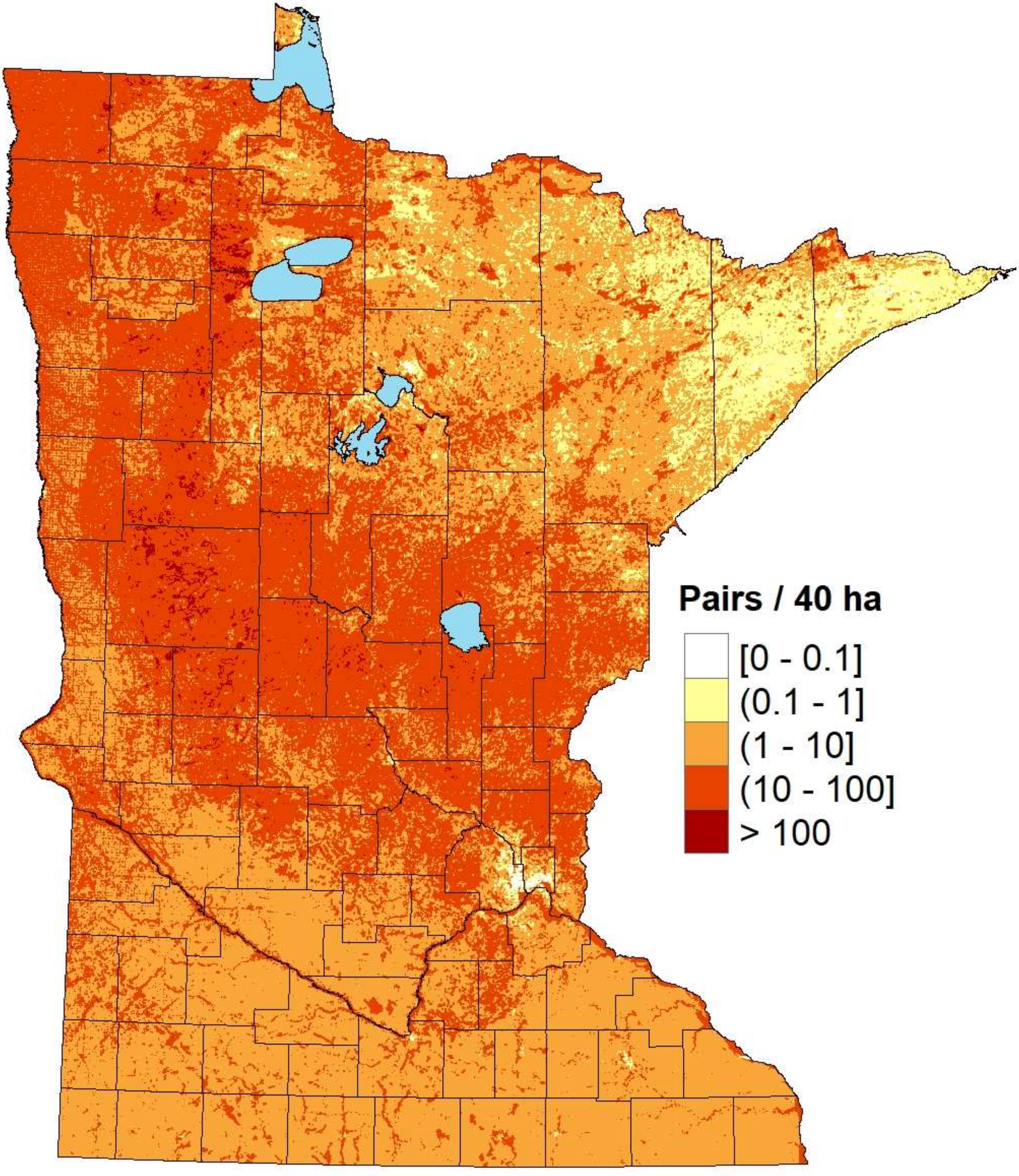

Yellow-bellied Flycatcher *Empidonax flaviventris*

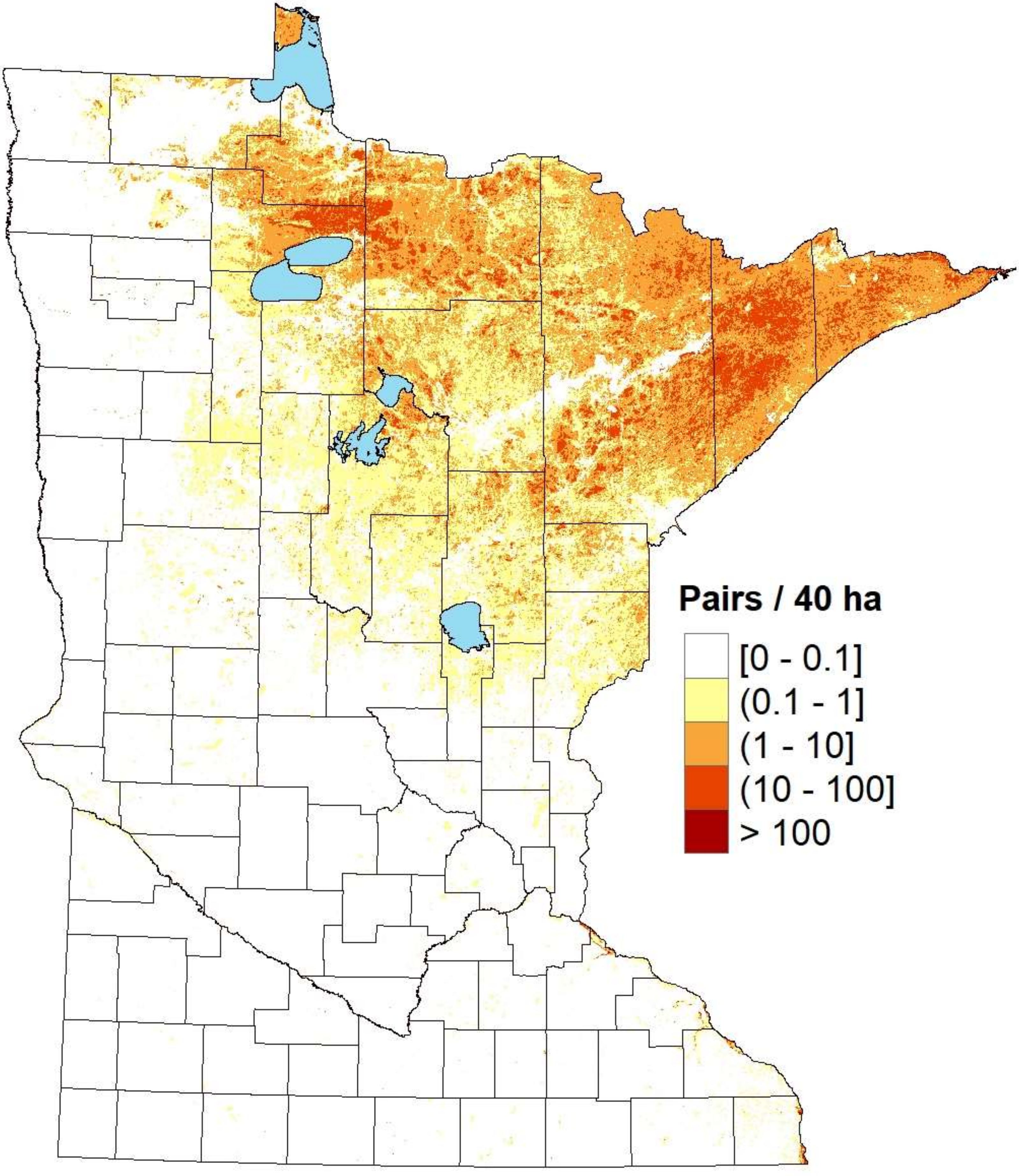

Yellow-headed Blackbird *Xanthocephalus xanthocephalus*

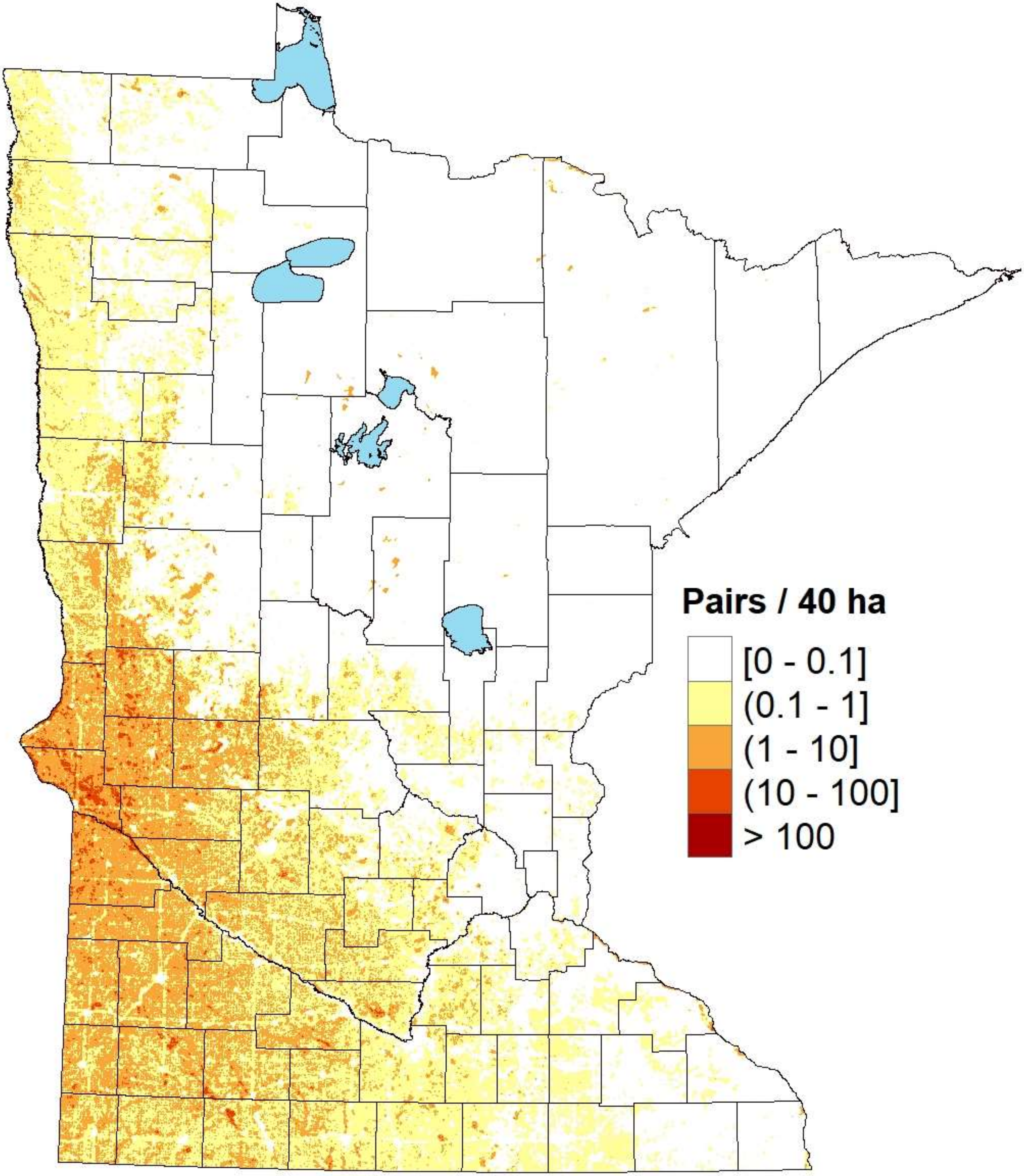

Yellow-rumped Warbler *Setophaga coronata*

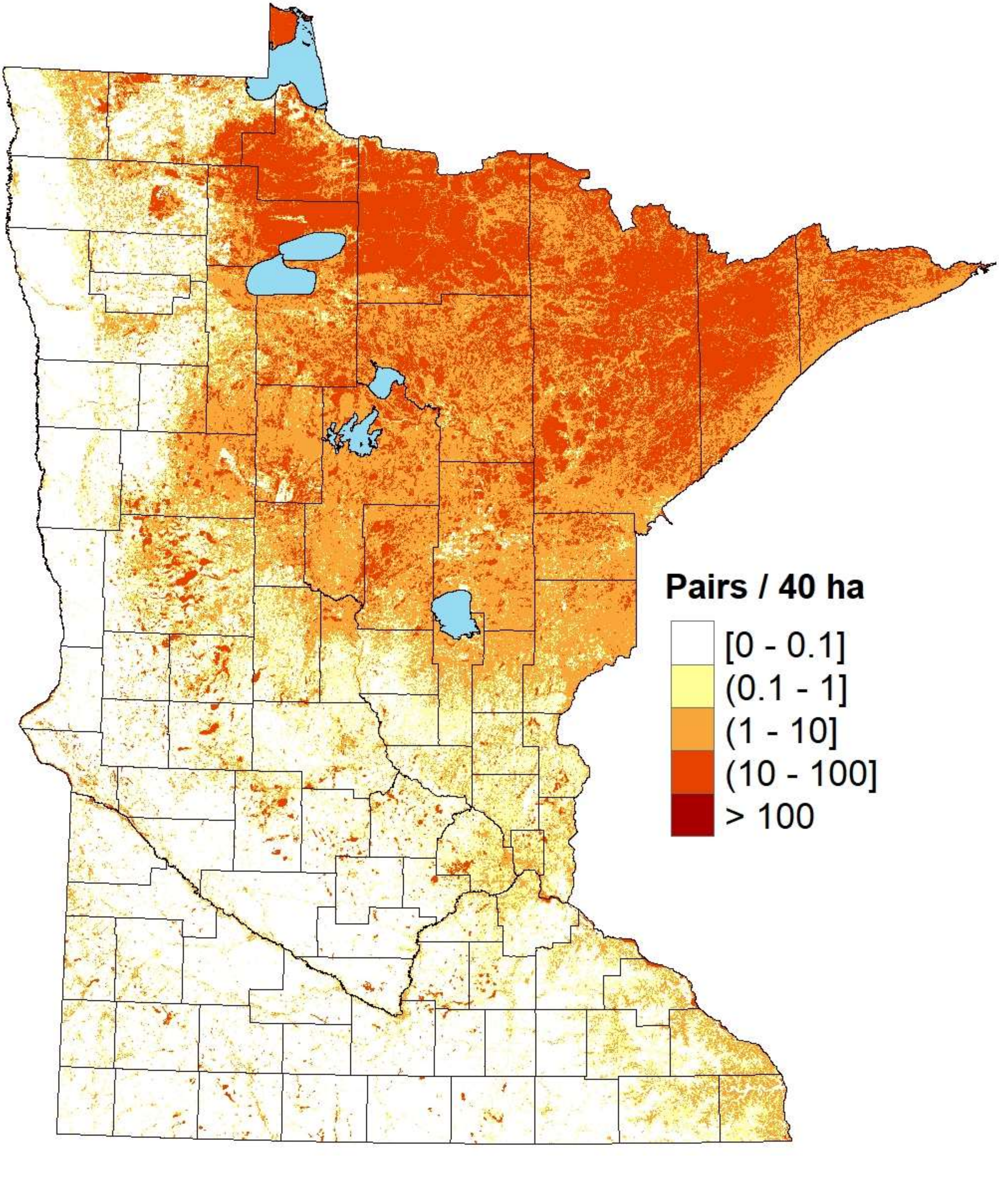

Yellow-throated Vireo *Vireo flavifrons*

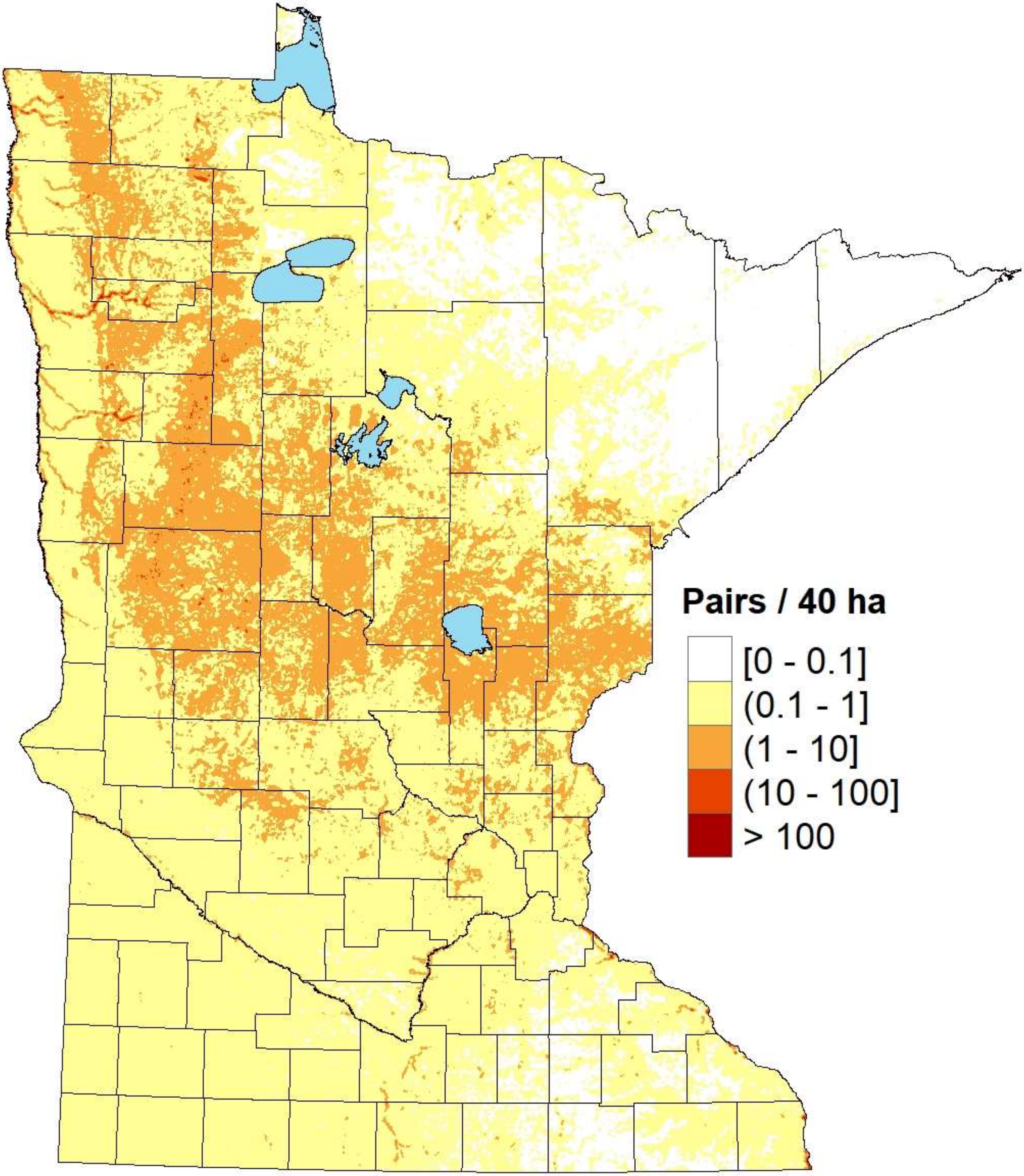

Maxent (33 species)

American kestrel *Falco sparverius*

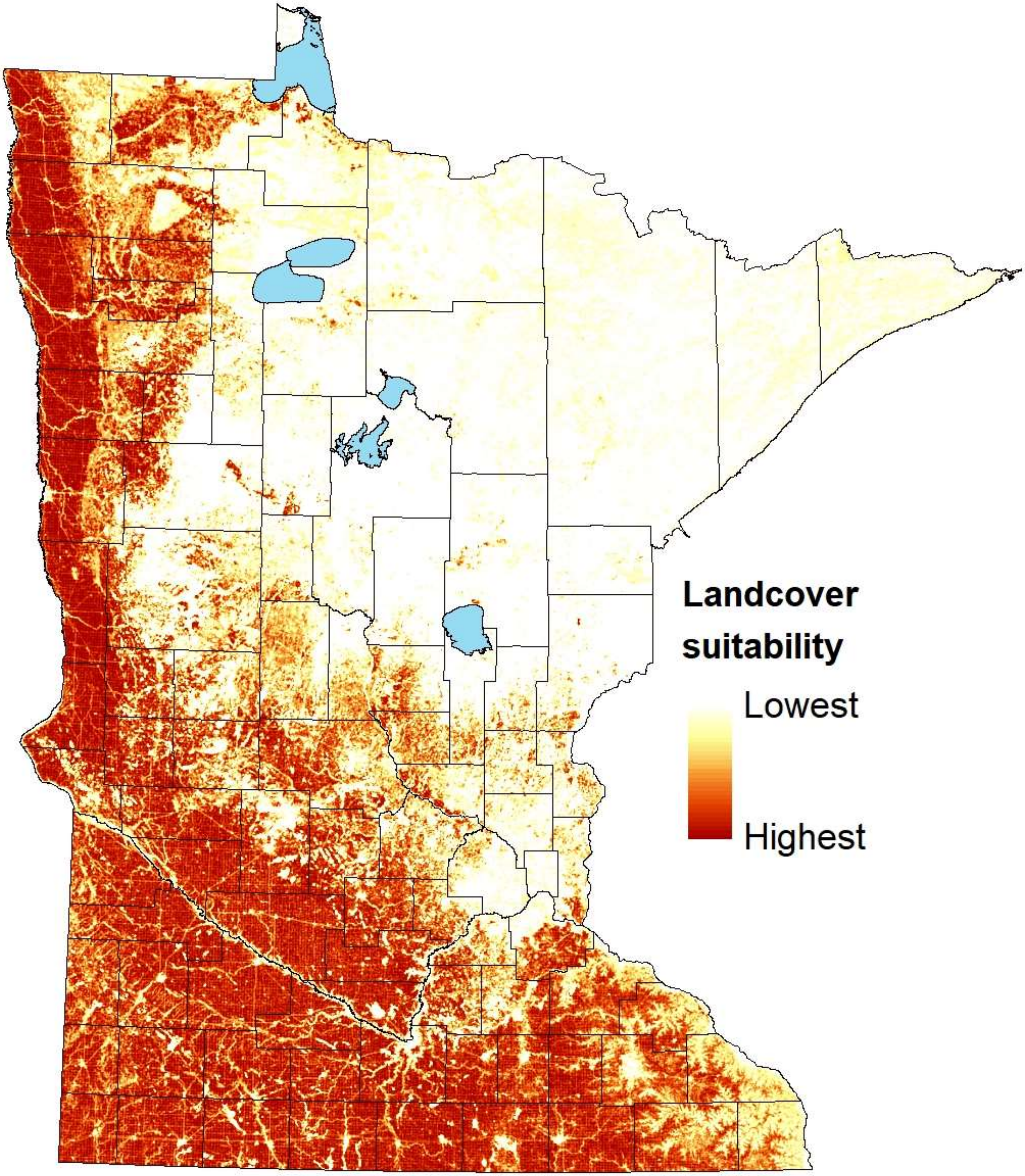

Bald Eagle *Haliaeetus leucocephalus*

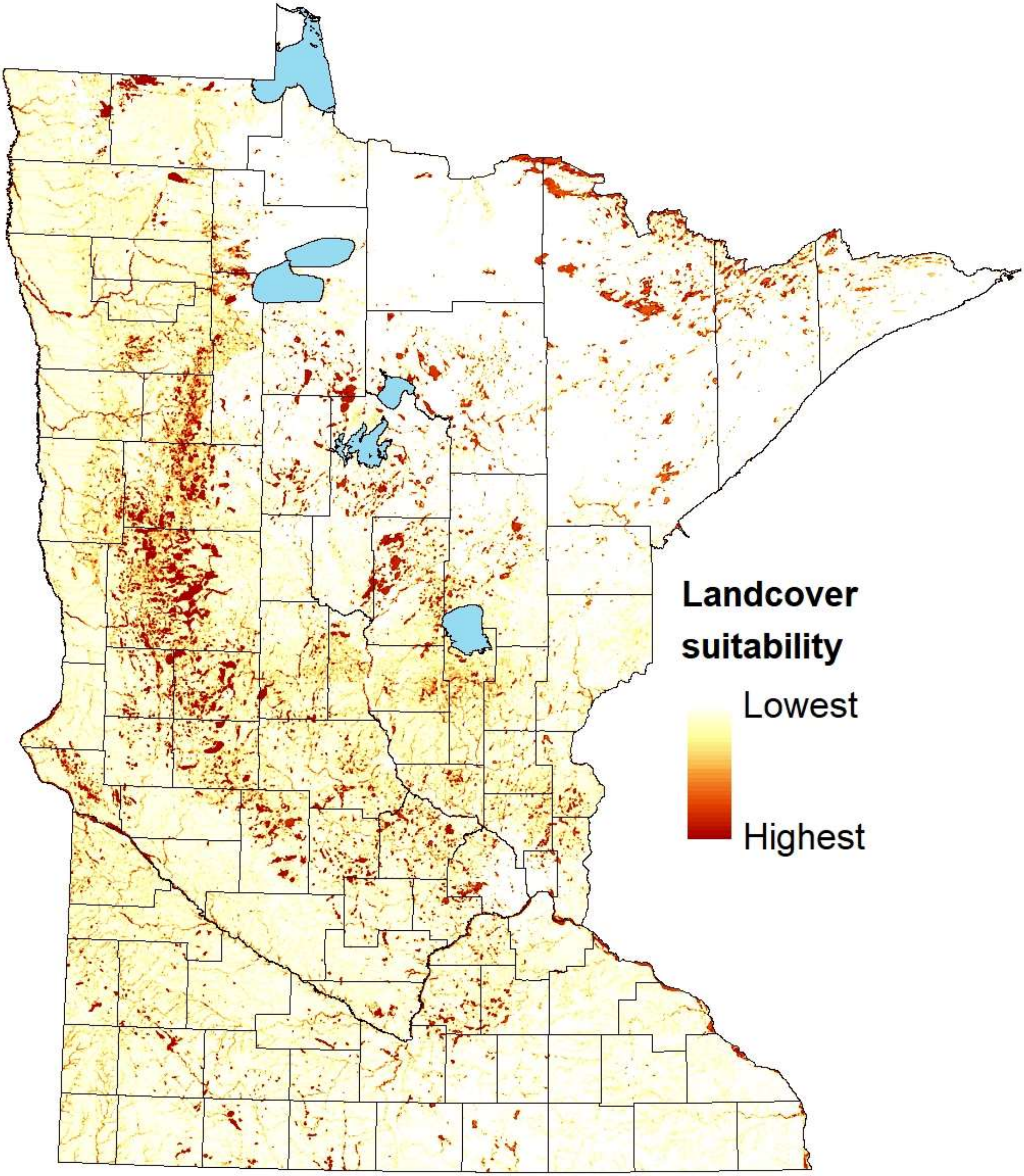

Bank Swallow *Riparia riparia*

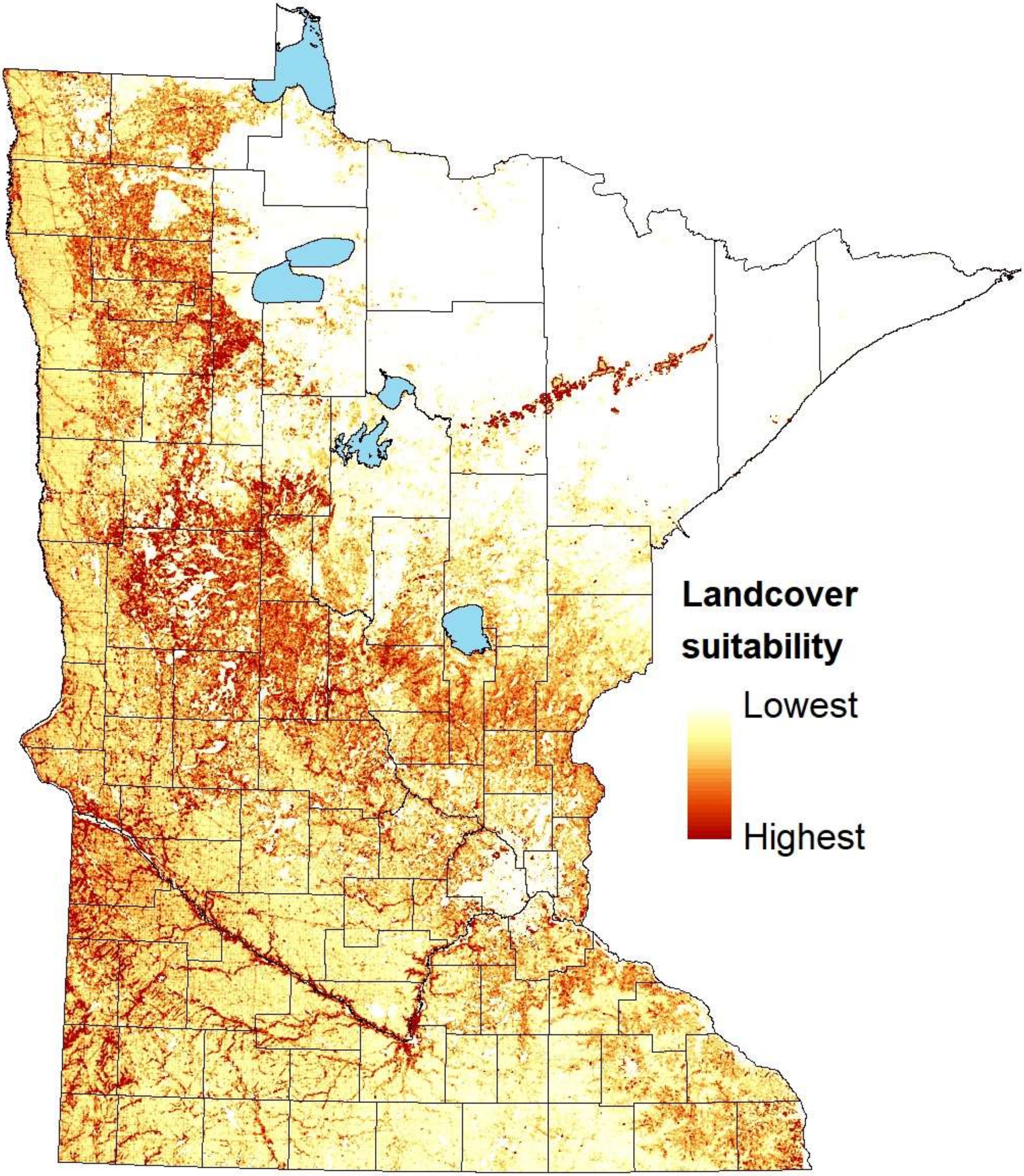

Barred Owl *Strix varia*

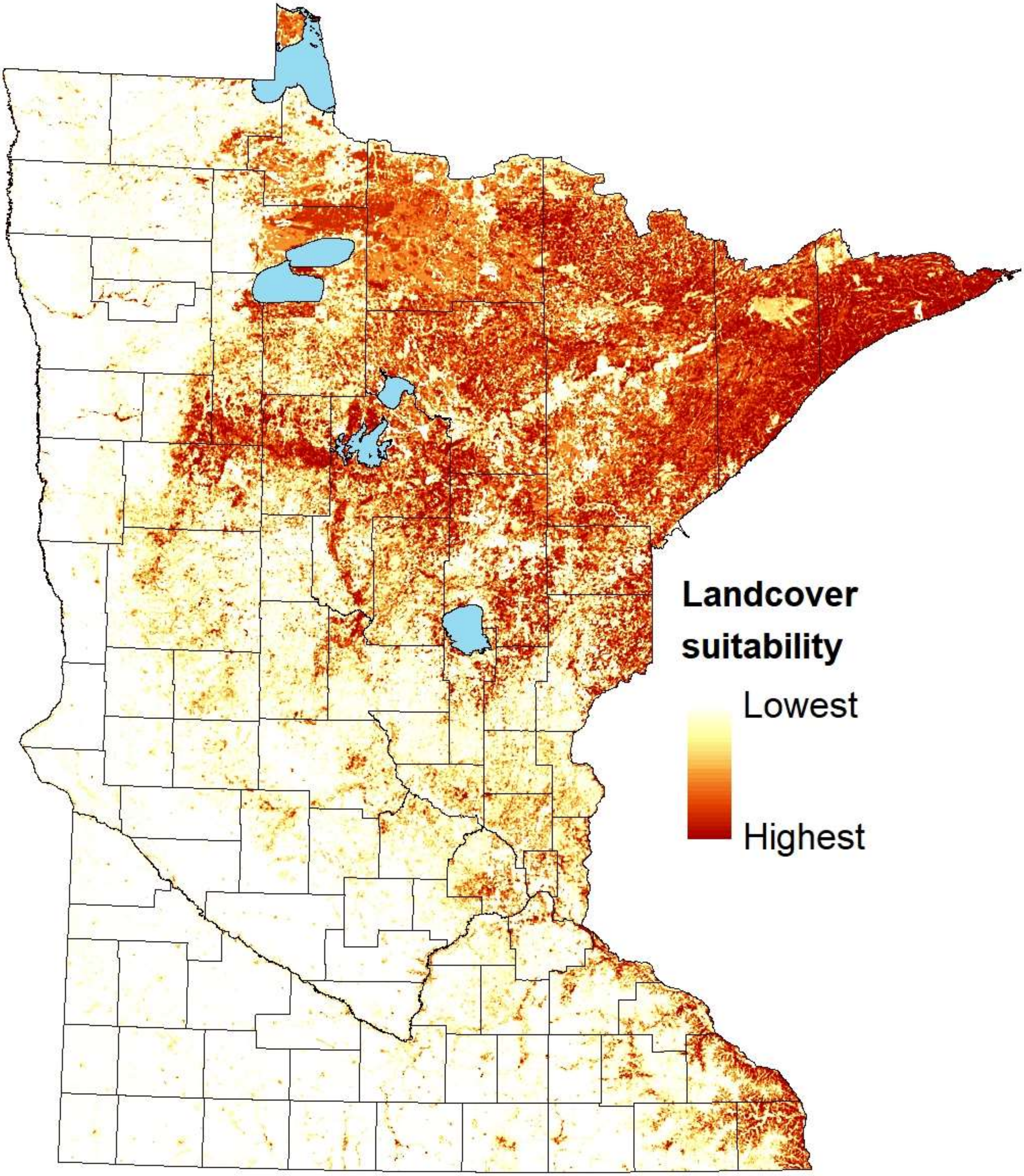

Black Tern *Chlidonias niger*

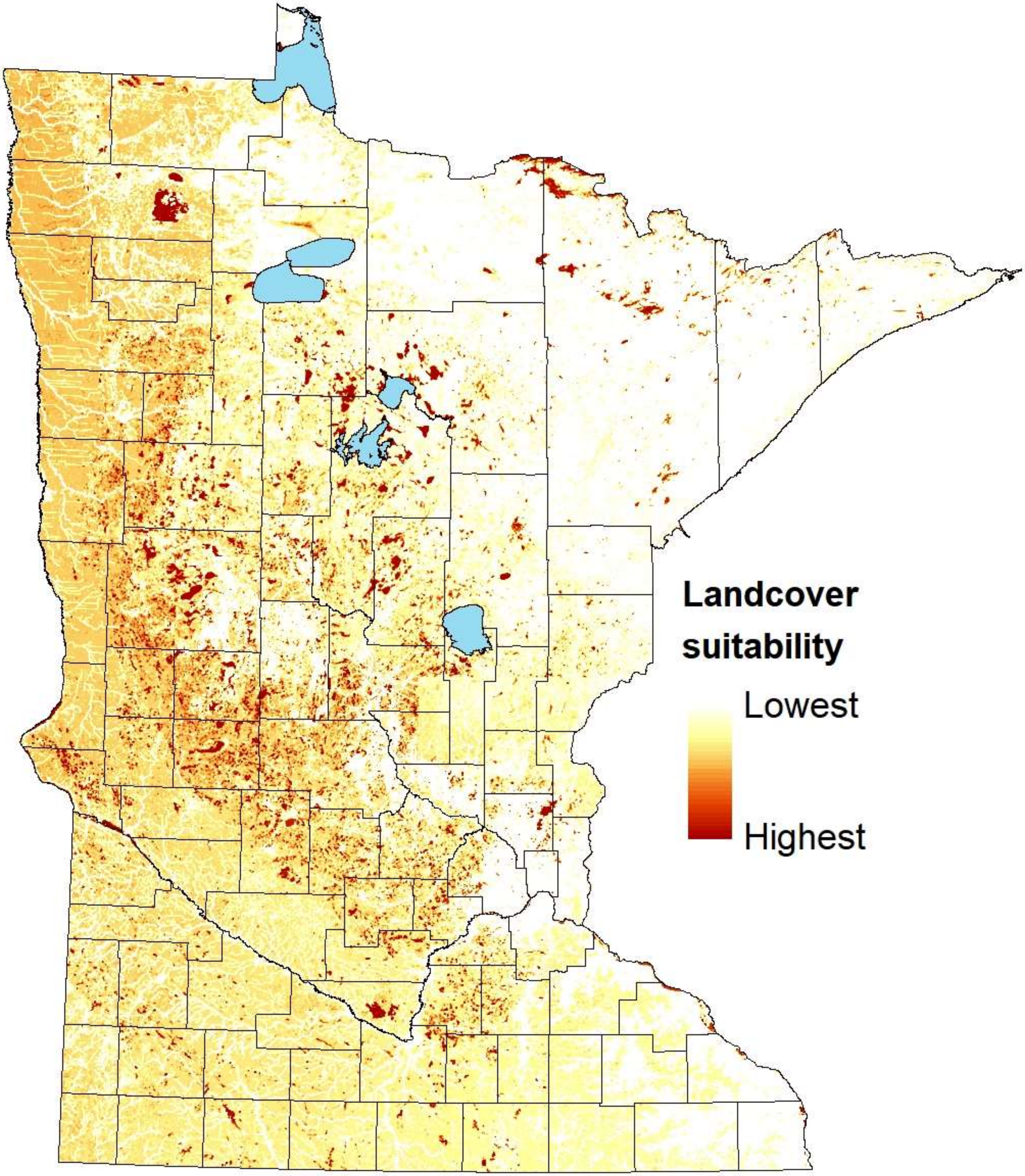

Blue-winged Warbler *Vermivora cyanoptera*

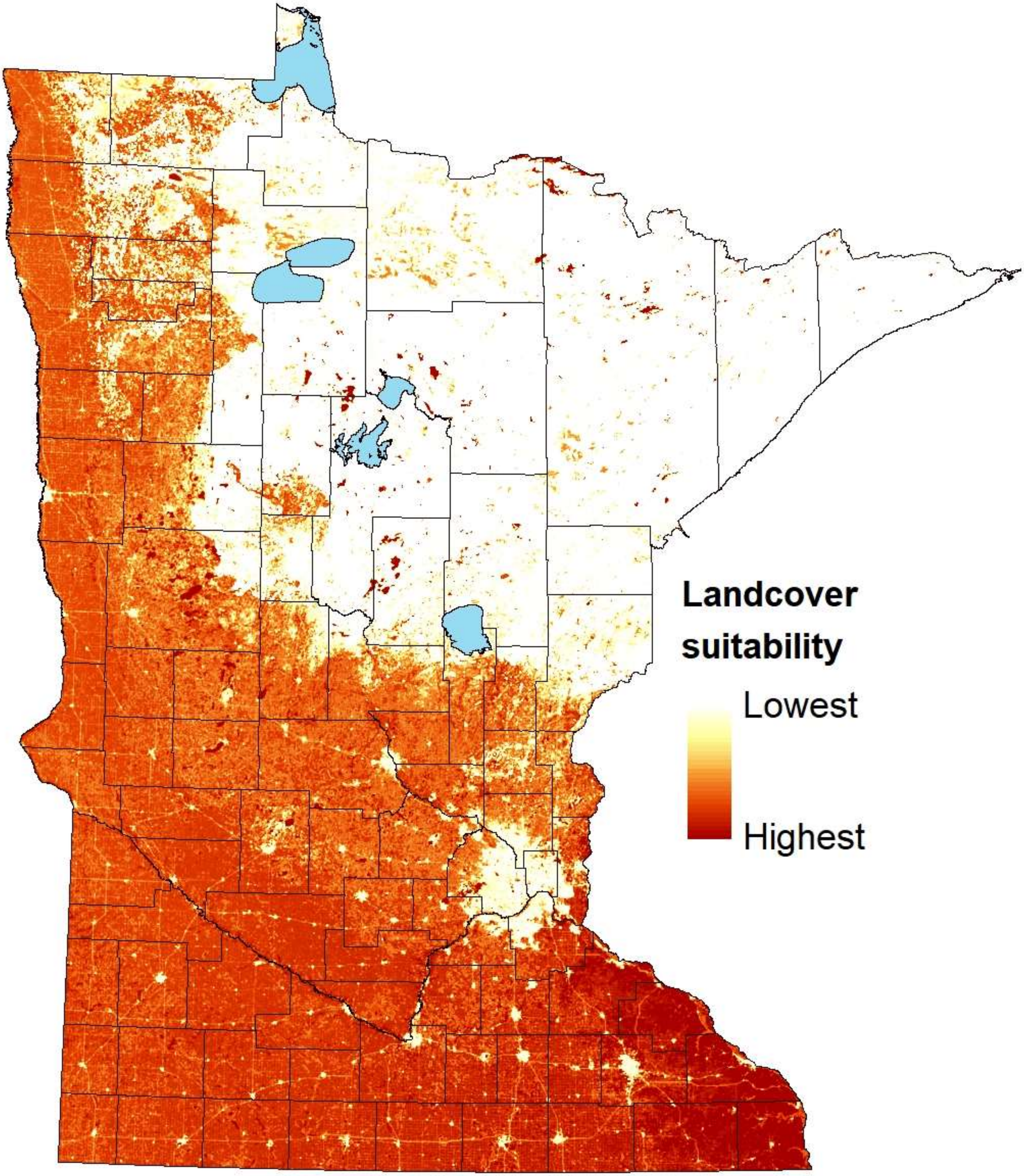

Broad-winged Hawk *Buteo platypterus*

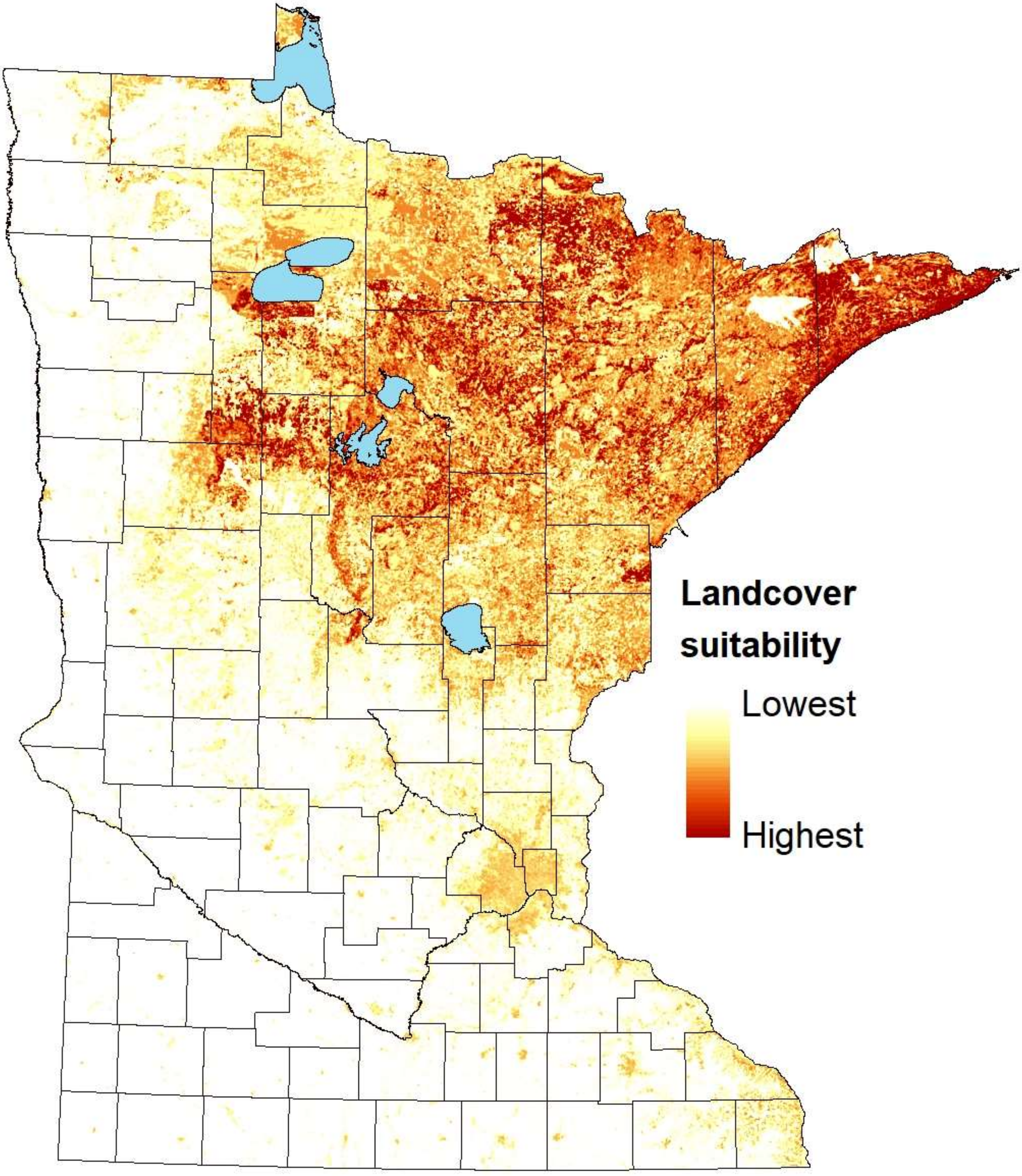

Cliff Swallow *Petrochelidon pyrrhonota*

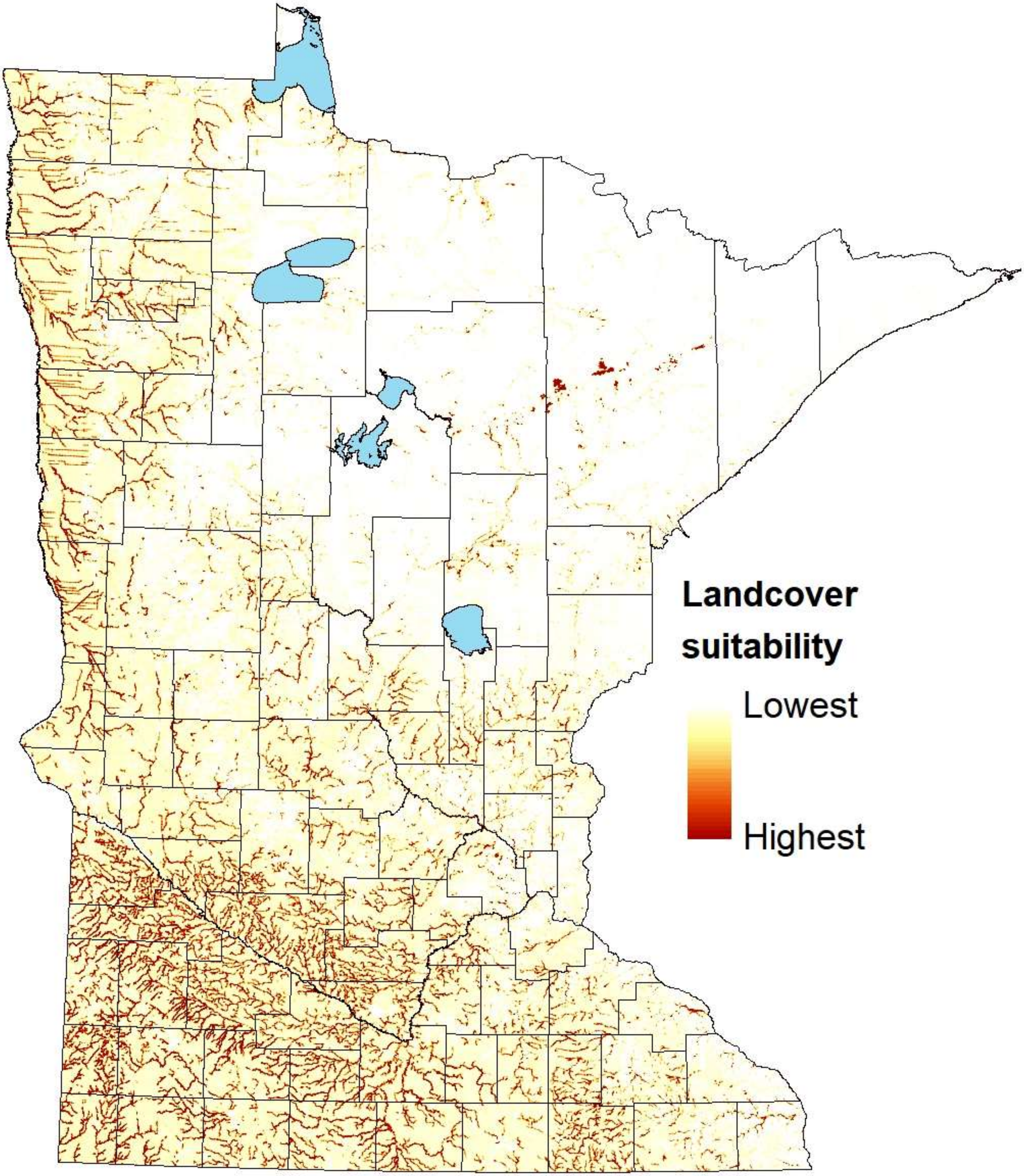

Common Goldeneye *Bucephala clangula*

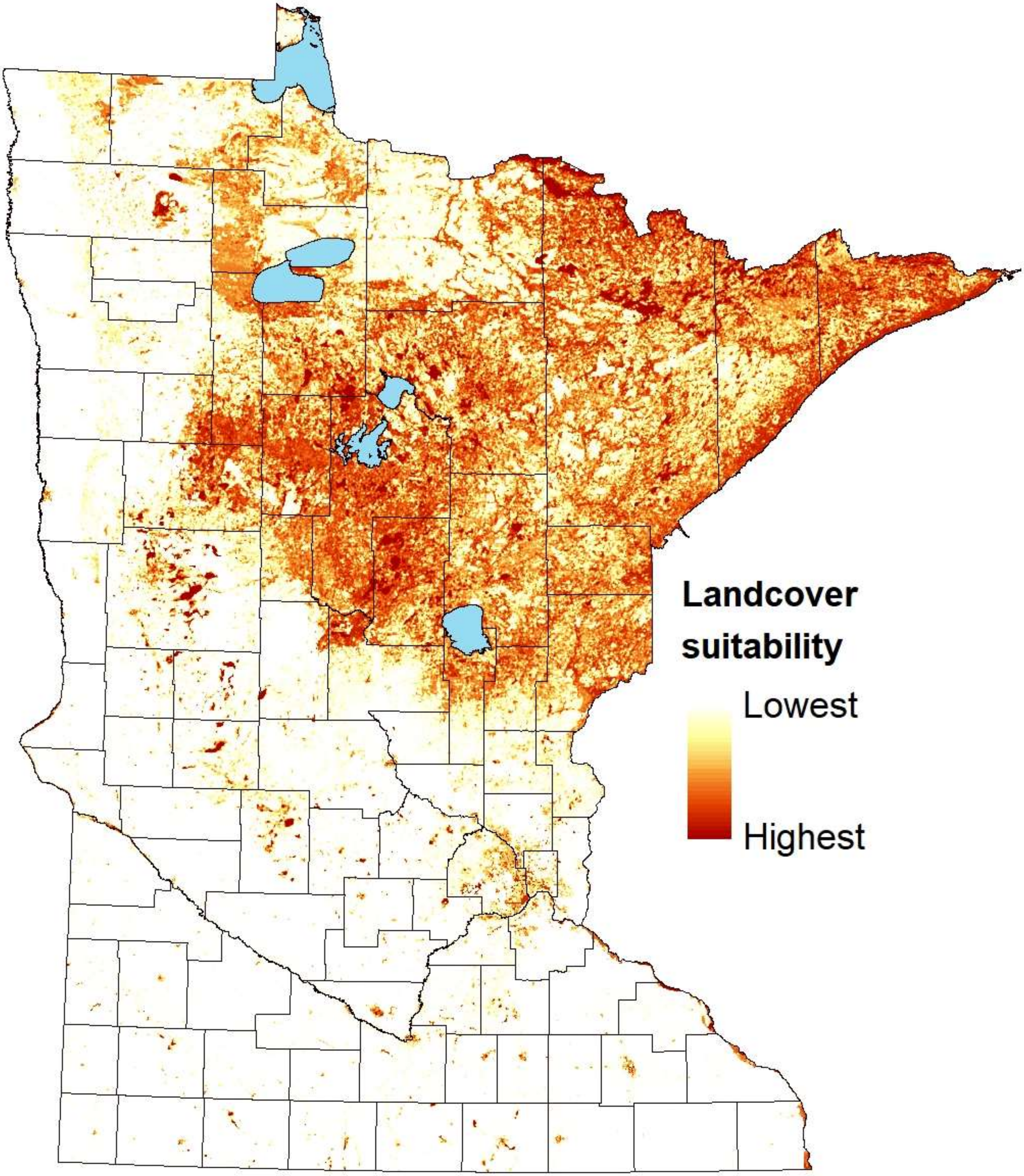

Common Loon *Gavia immer*

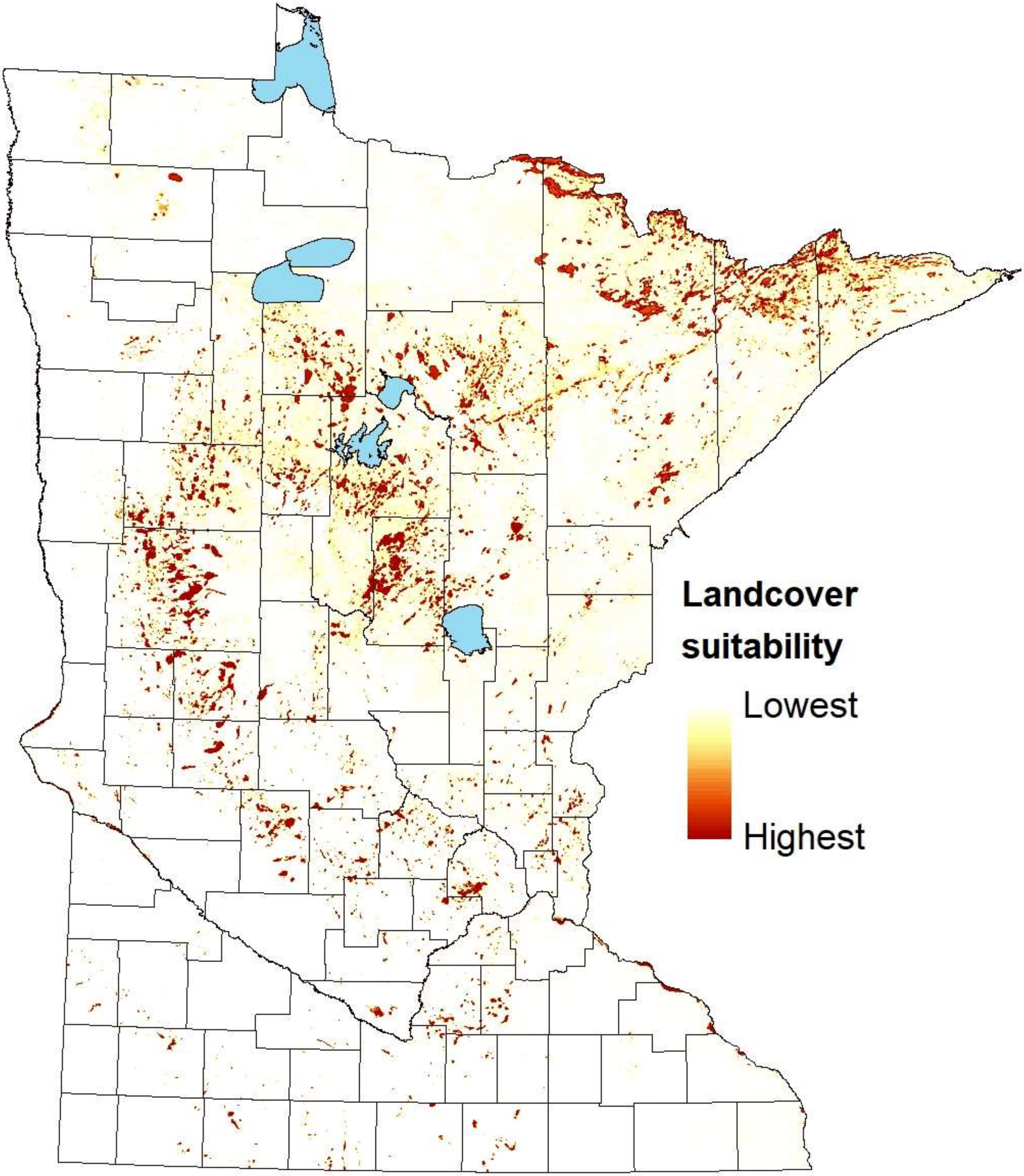

Cooper's Hawk *Accipiter cooperii*

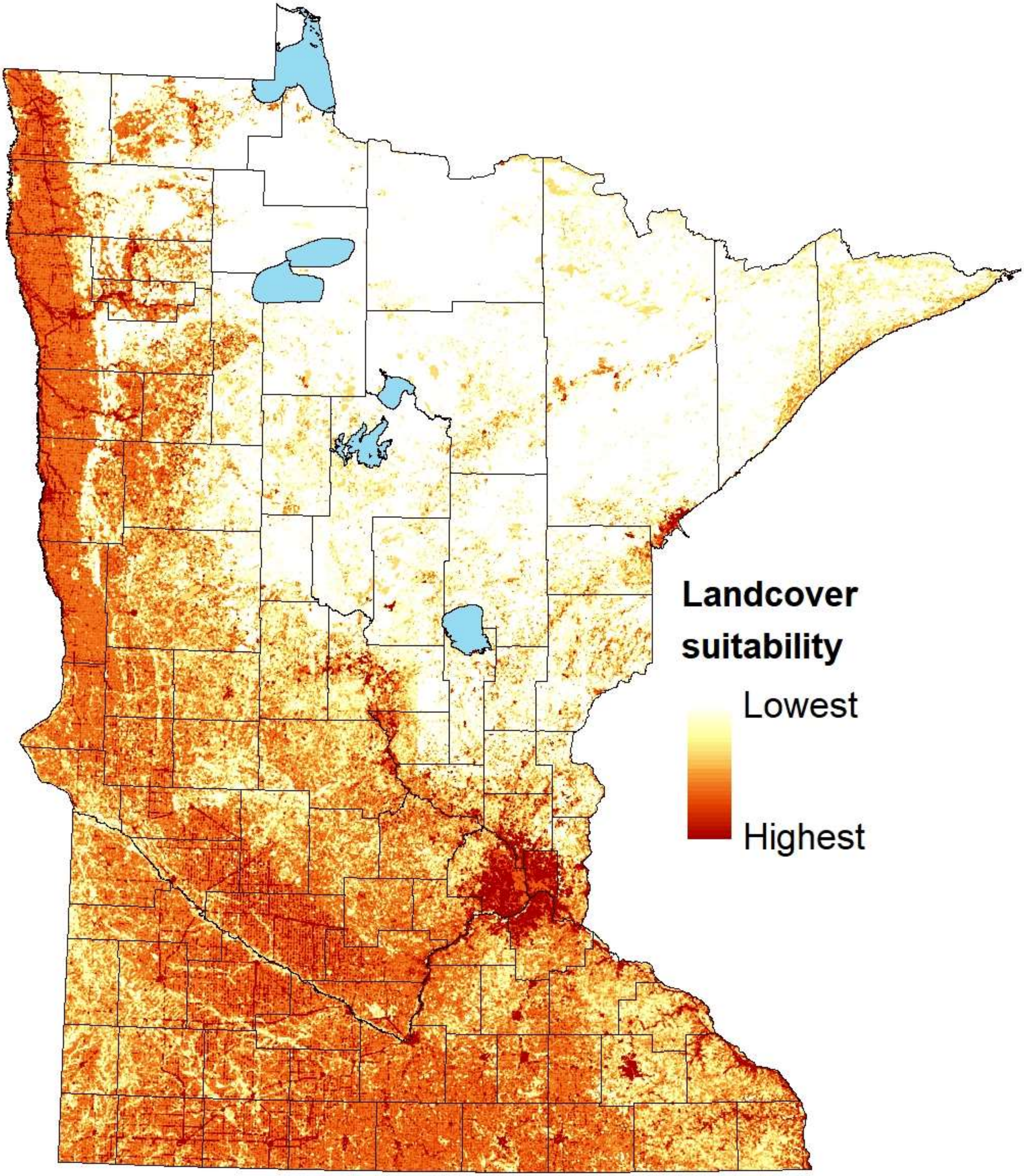

Eastern Towhee *Pipilo erythrophthalmus*

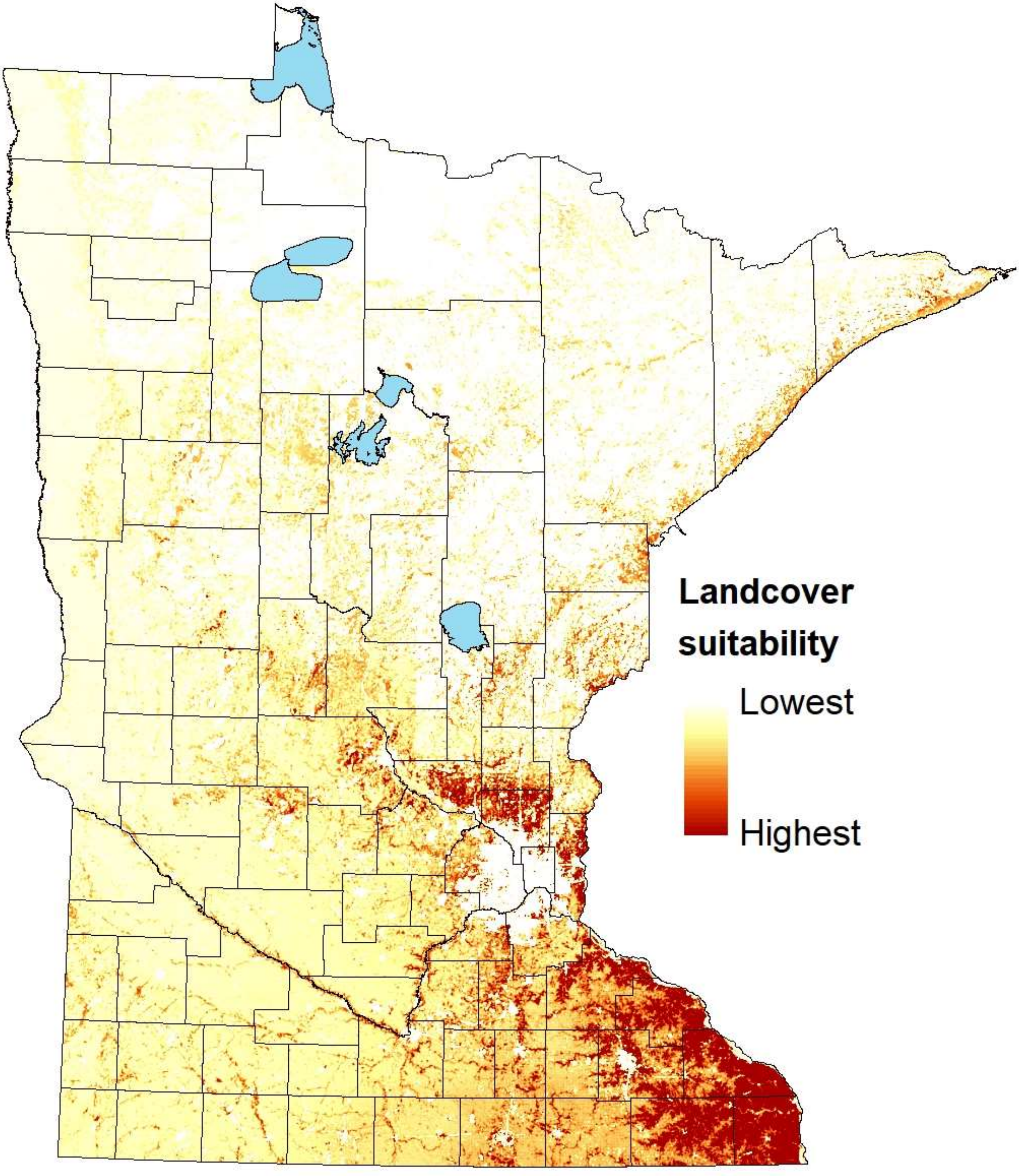

Gray Partridge *Perdix perdix*

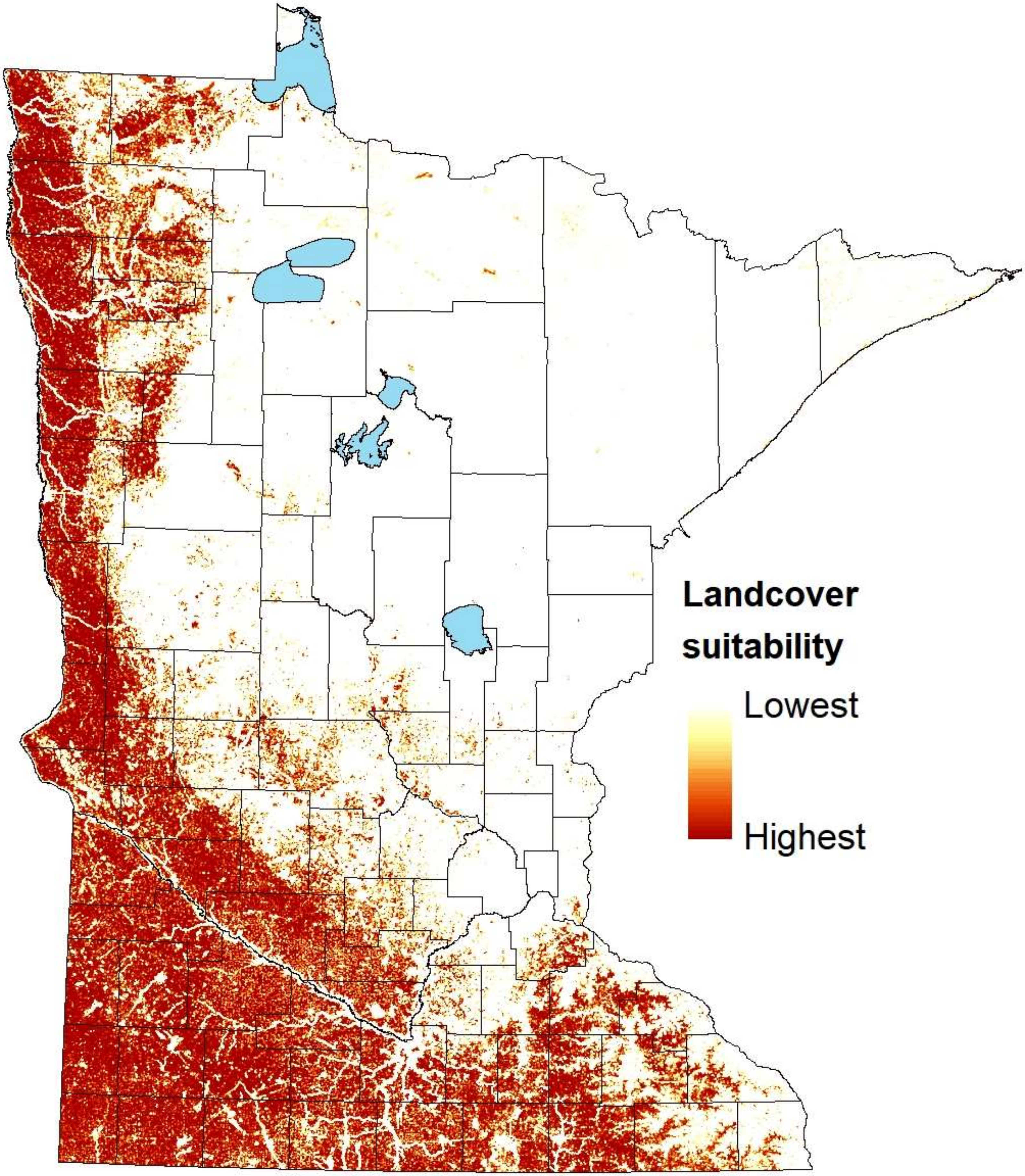

Henslow's Sparrow *Centronyx henslowii*

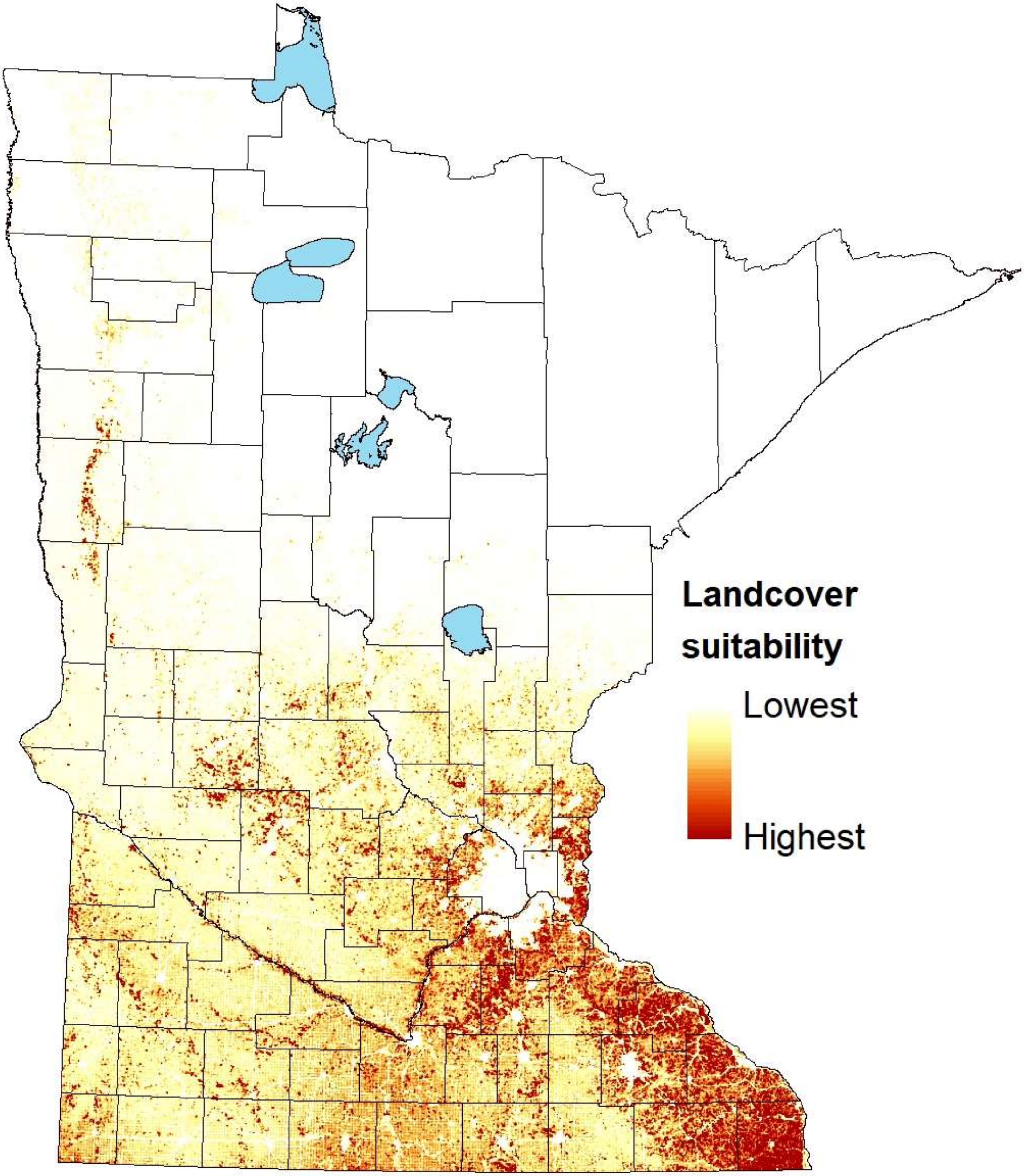

Lark Sparrow *Chondestes grammacus*

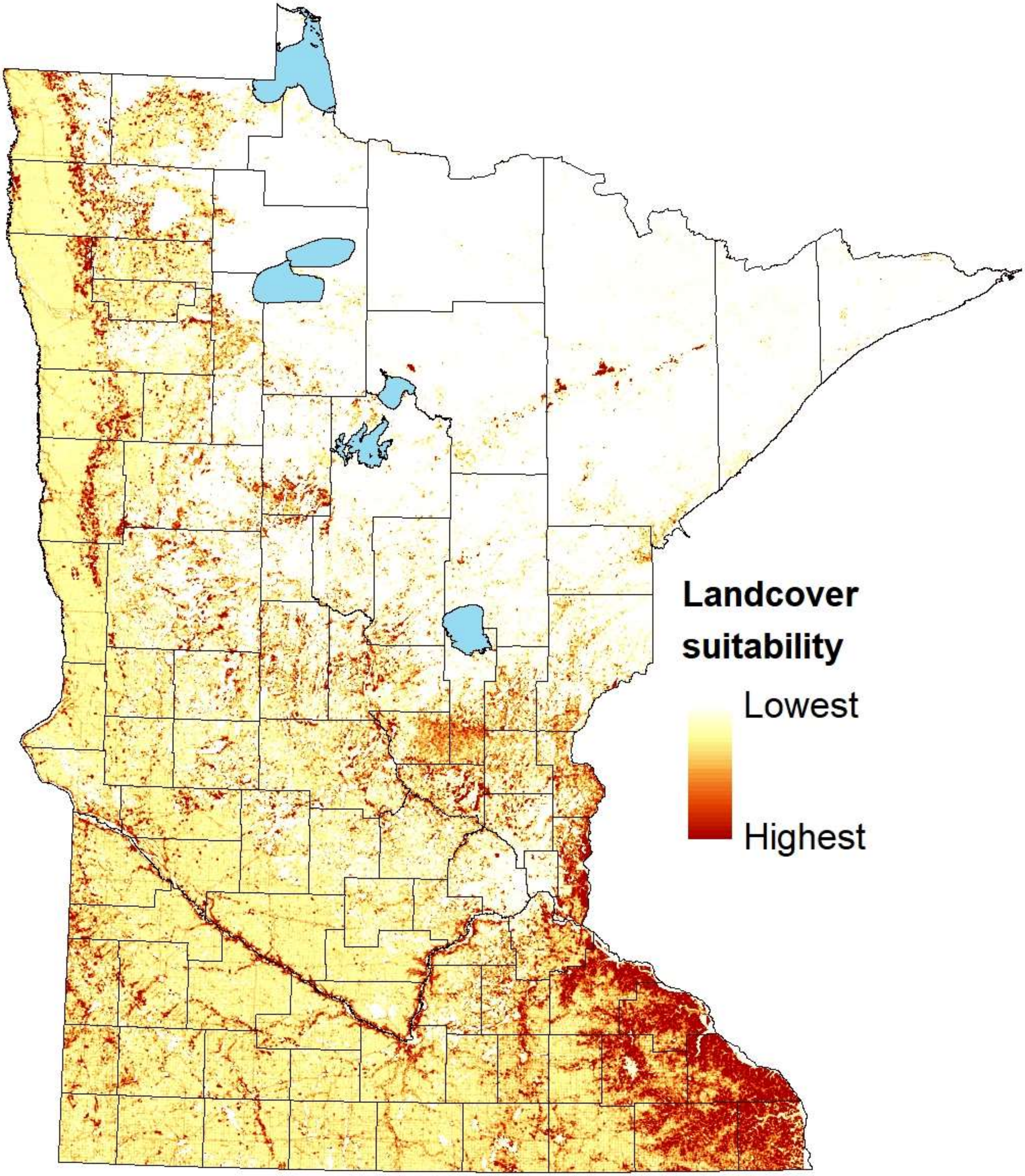

Loggerhead Shrike *Lanius ludovicianus*

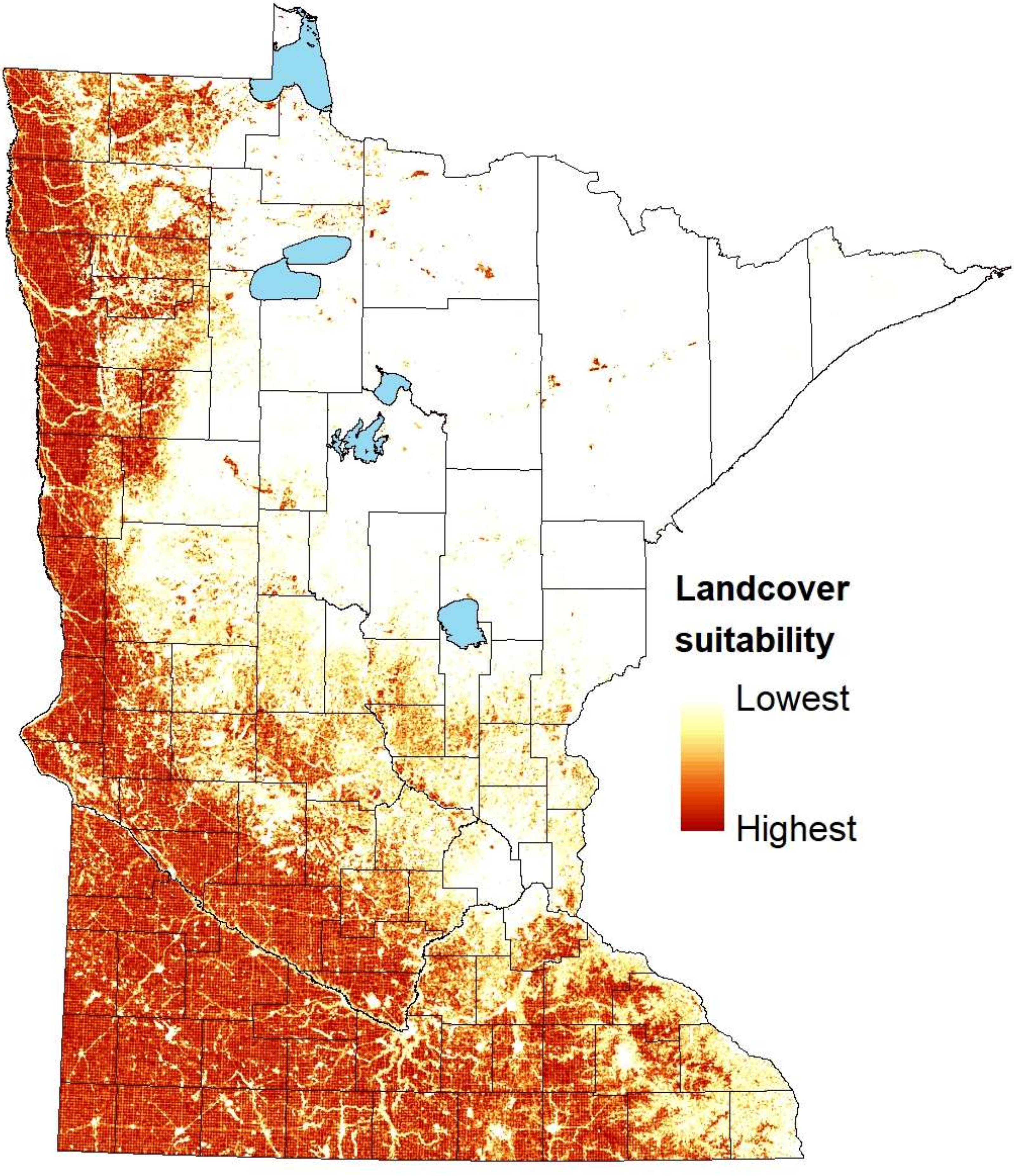

Merlin *Falco columbarius*

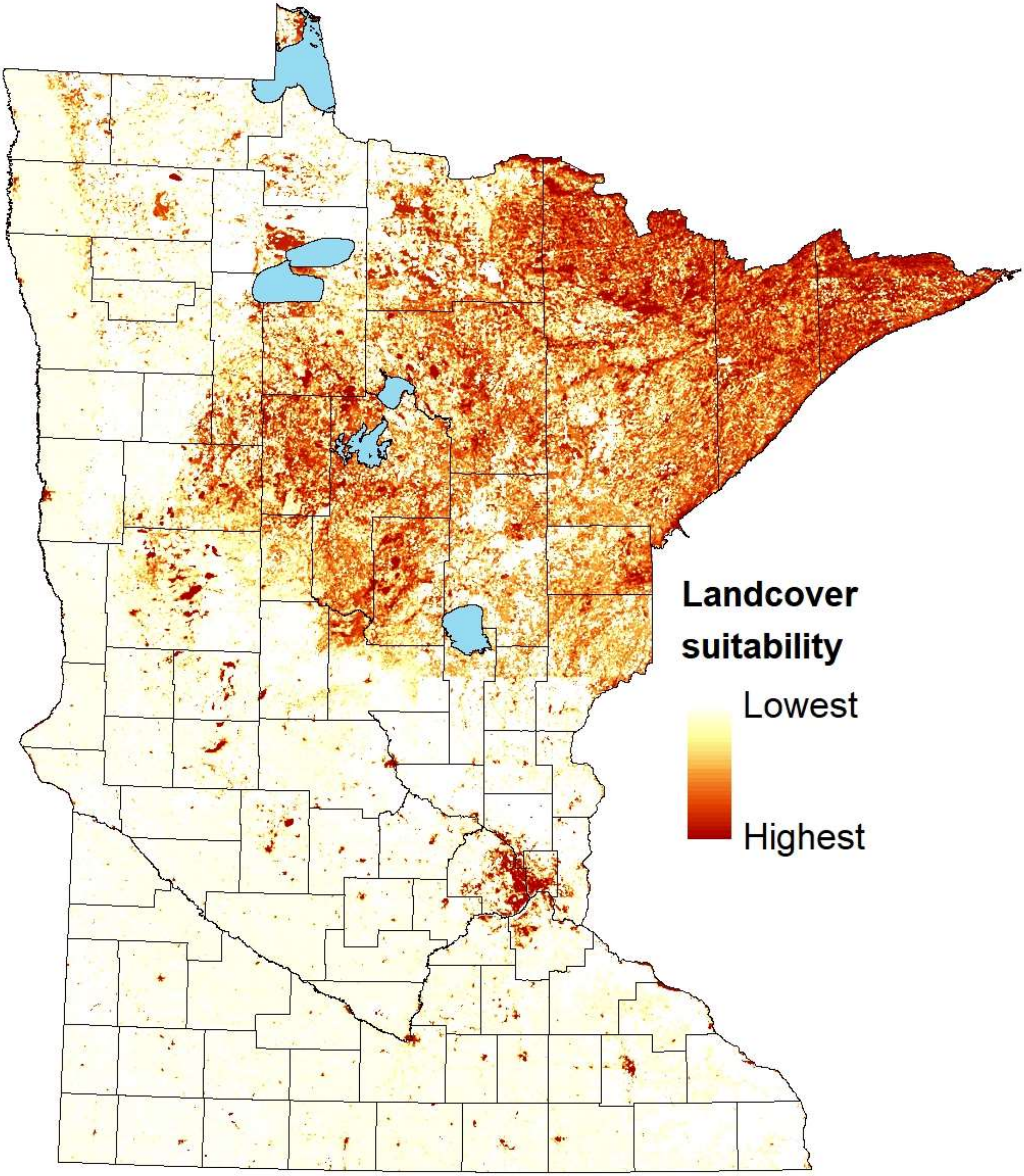

Northern Rough-winged Swallow *Stelgidopteryx serripennis*

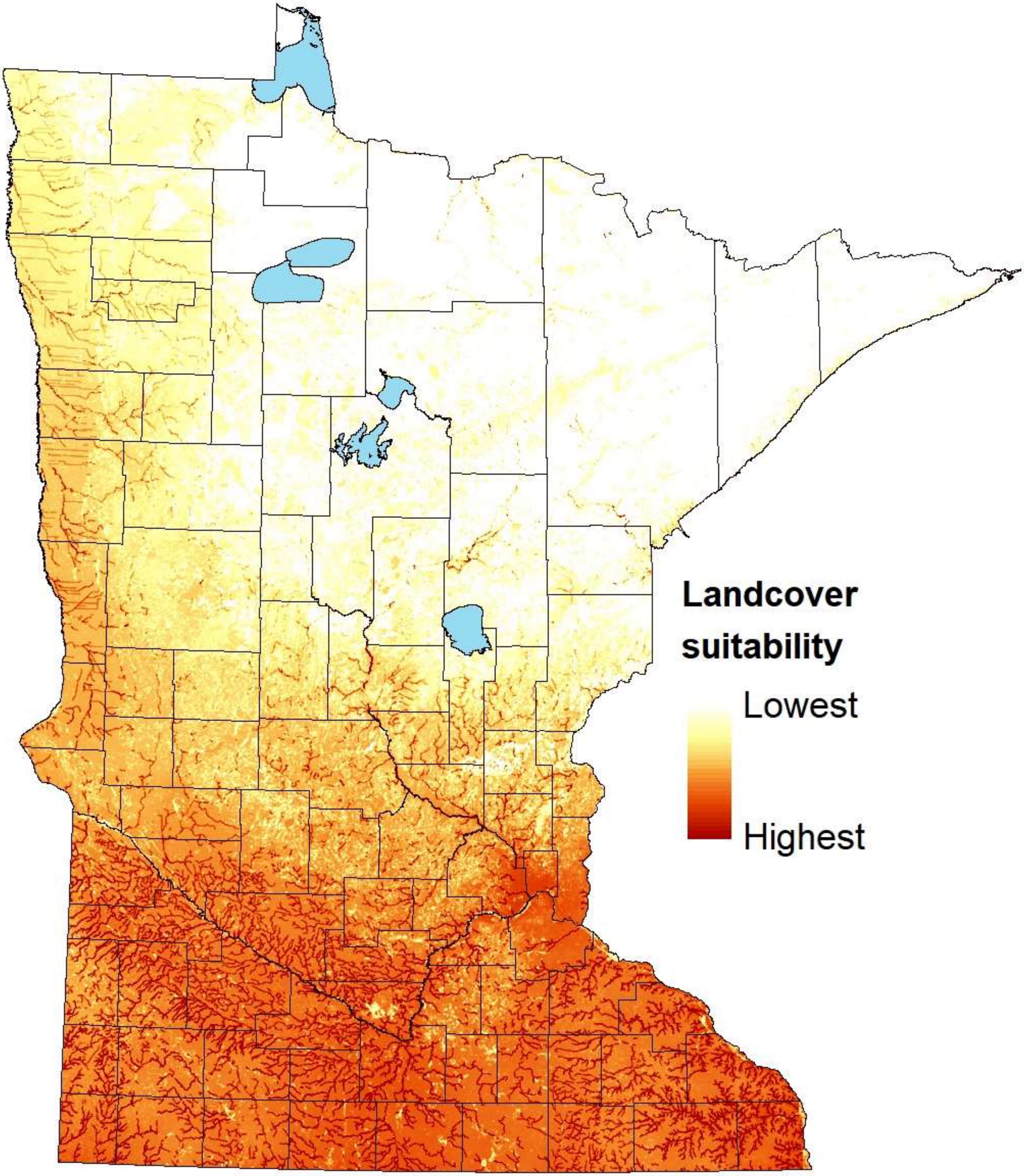

Orchard Oriole *Icterus spurius*

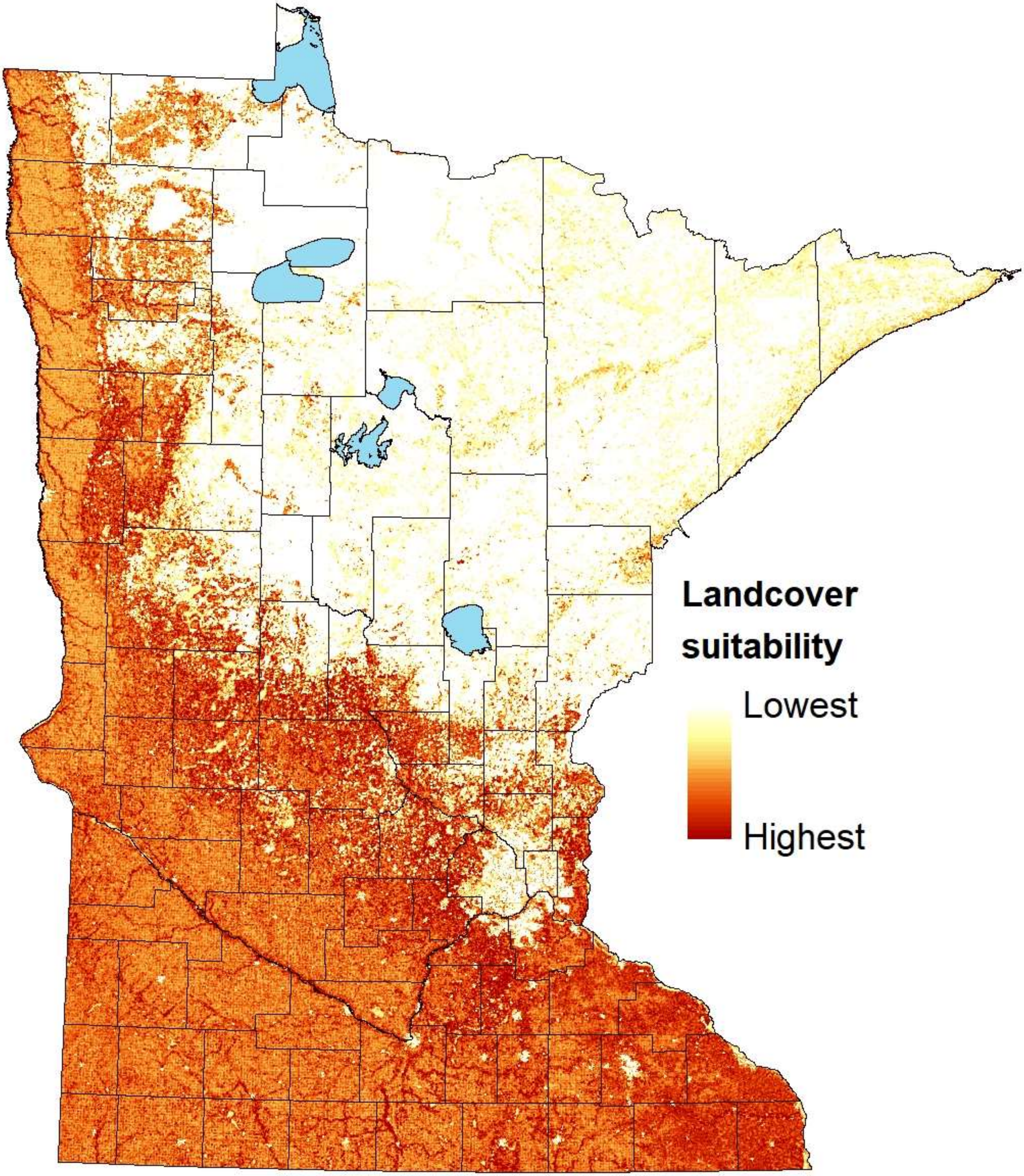

Osprey *Pandion haliaetus*

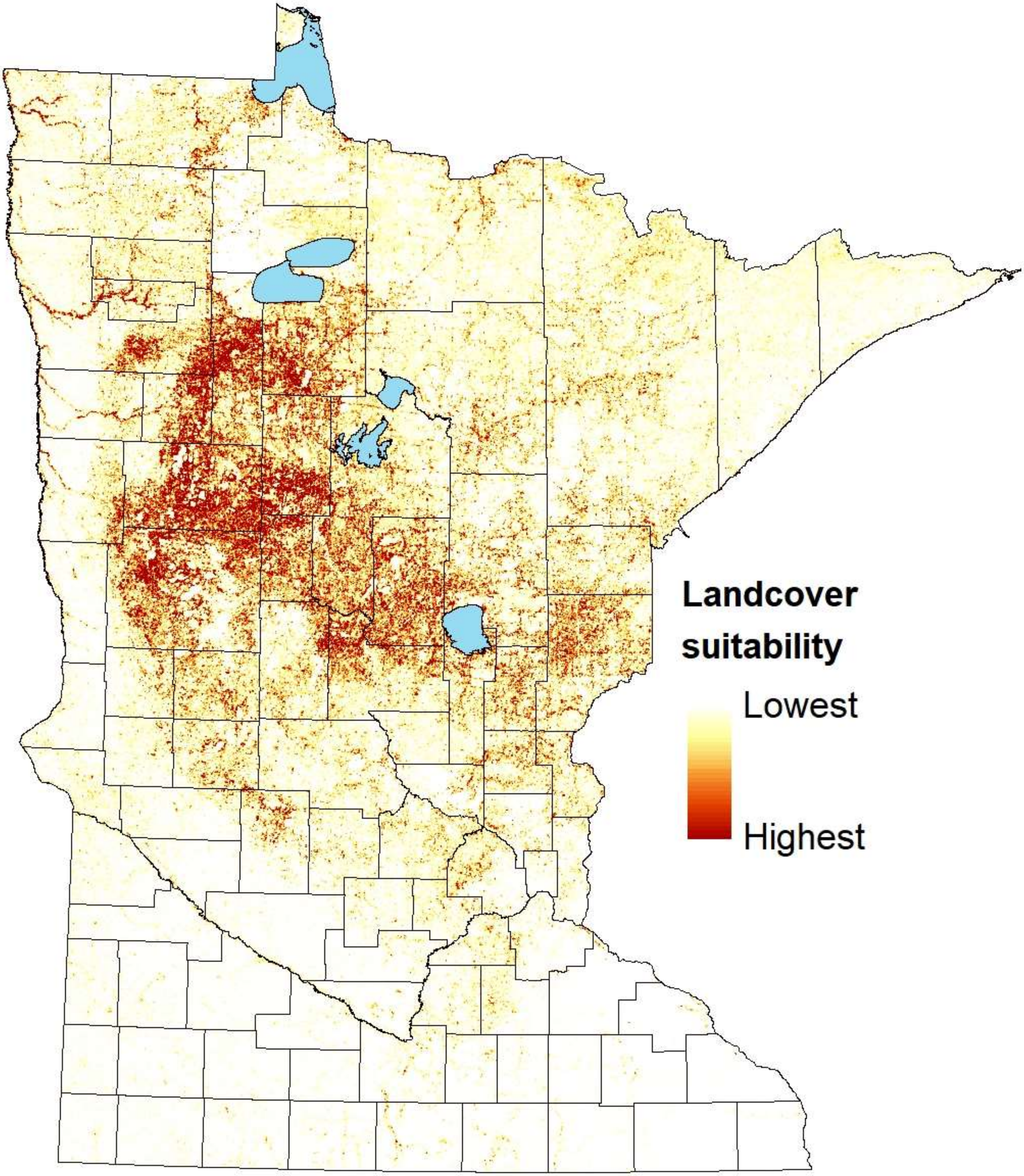

Pileated Woodpecker *Dryocopus pileatus*

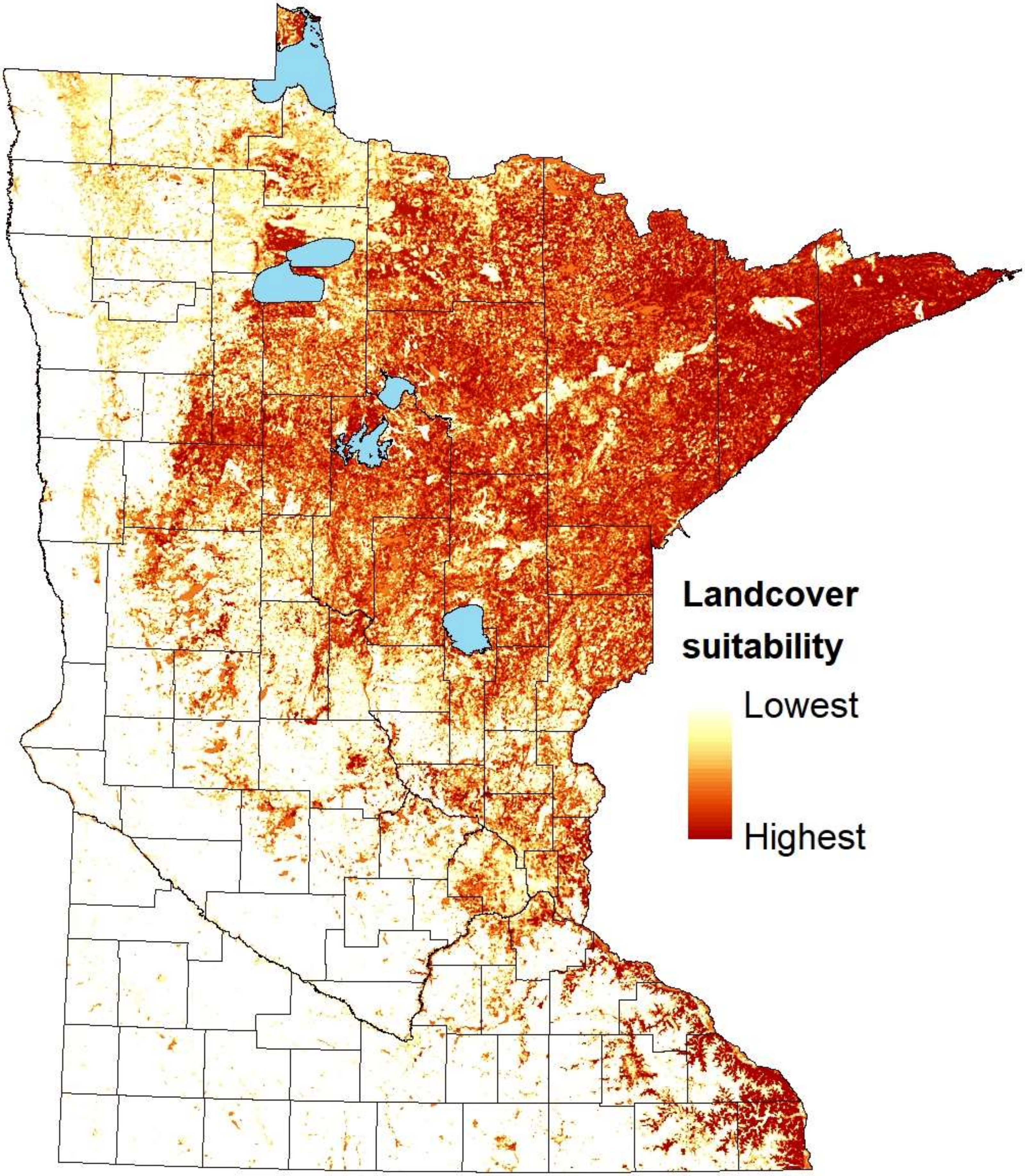

Purple Martin *Progne subis*

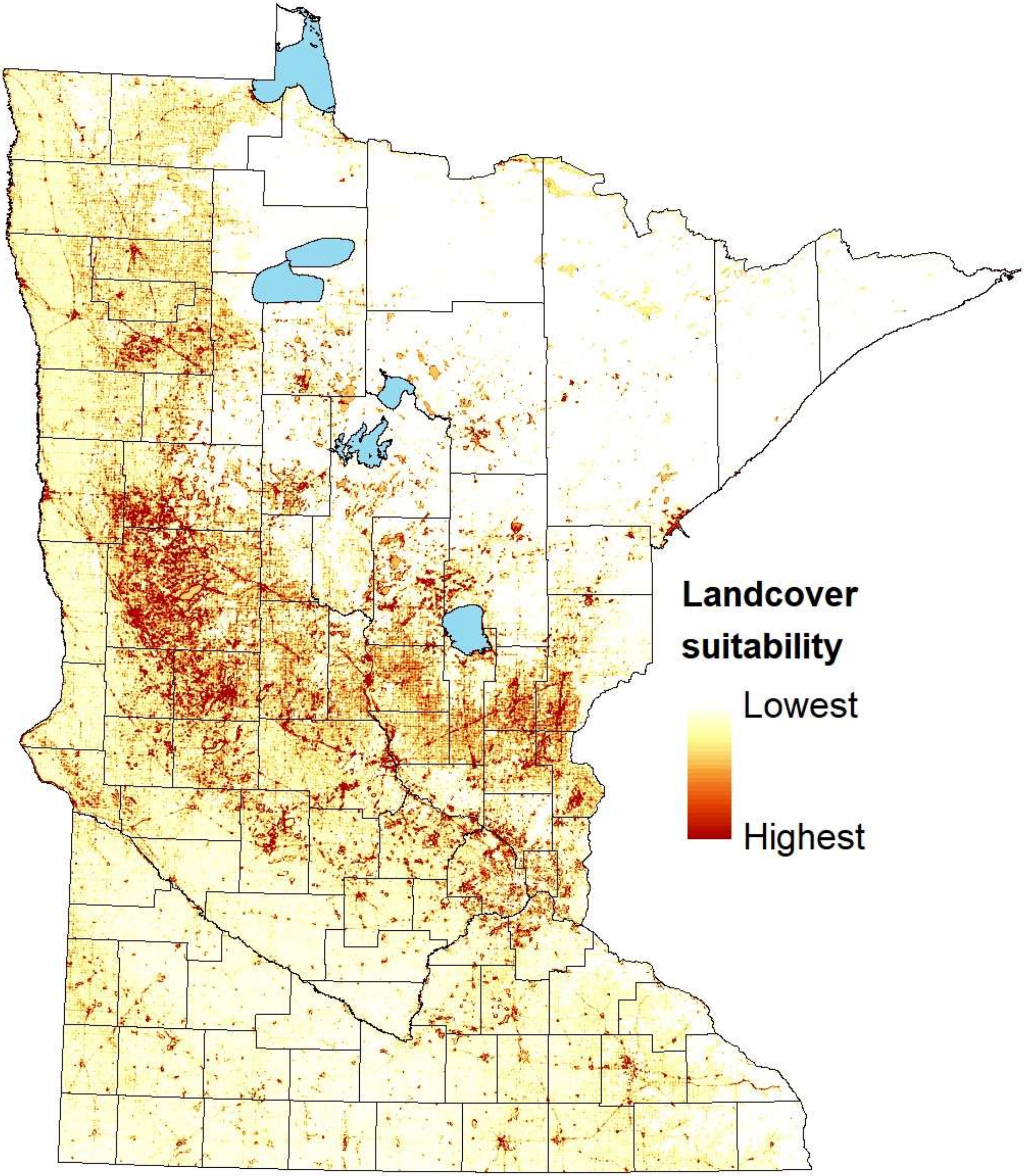

Red-headed Woodpecker *Melanerpes erythrocephalus*

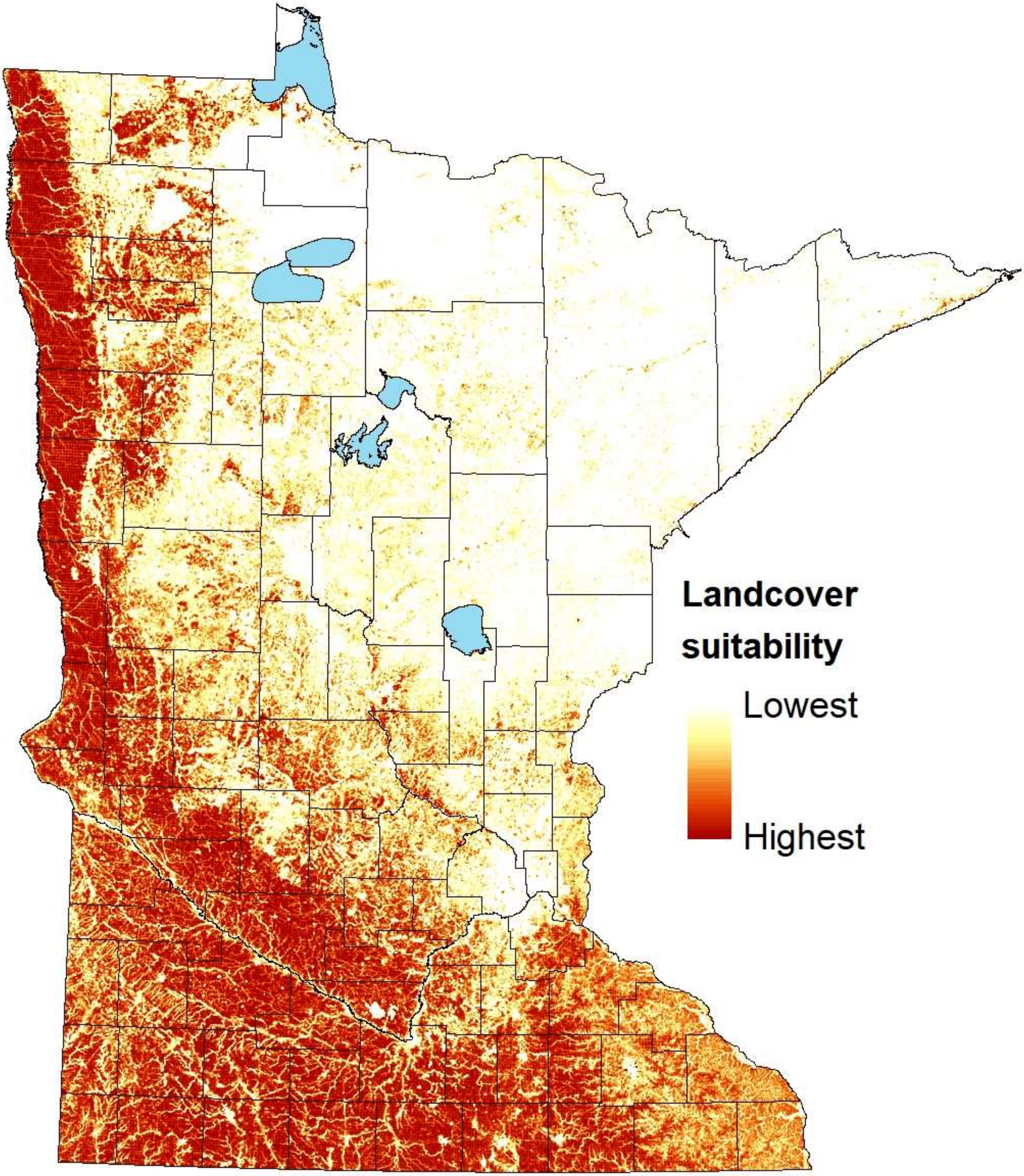

Red-necked Grebe *Podiceps grisegena*

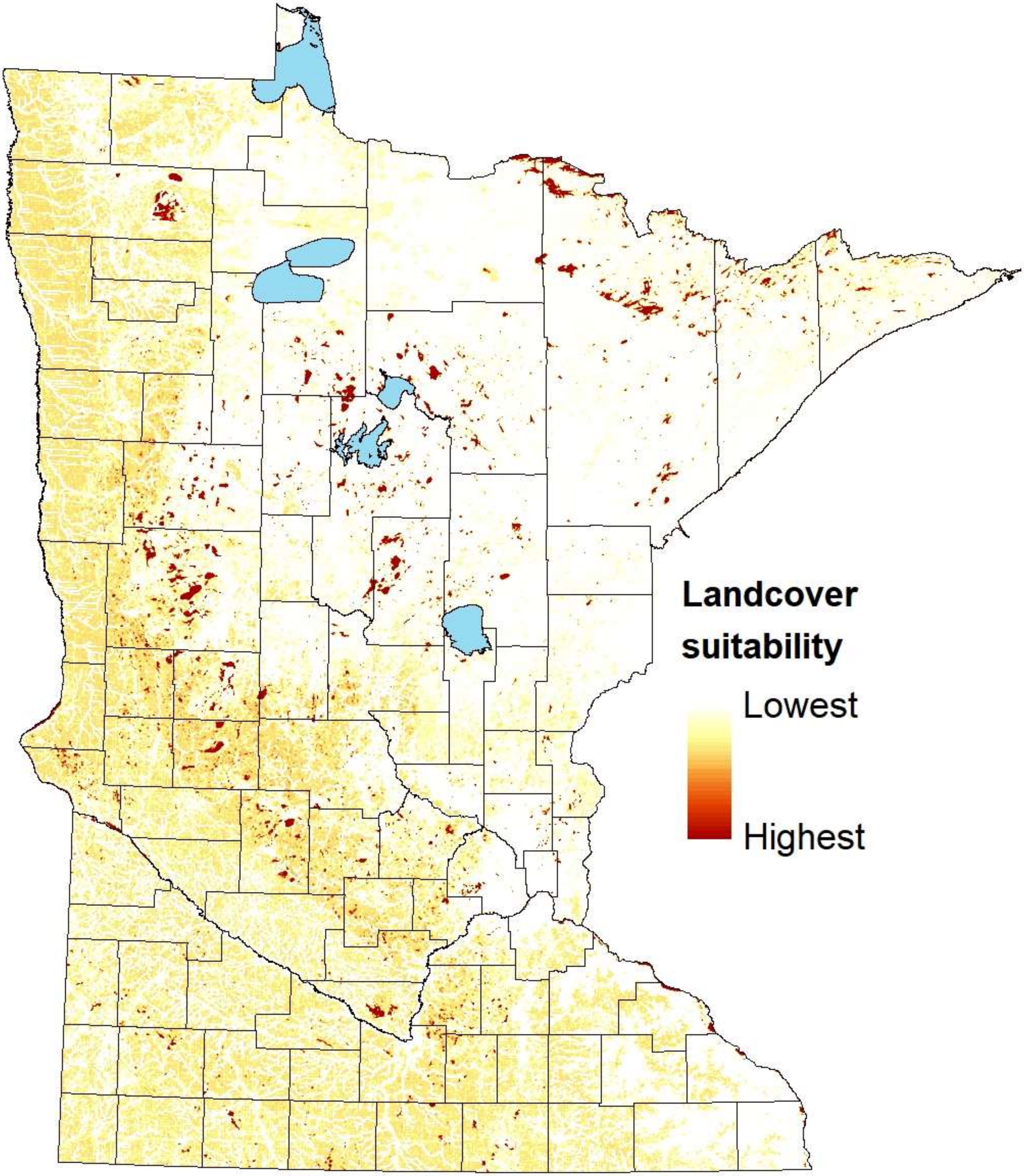

Ring-necked Duck *Aythya collaris*

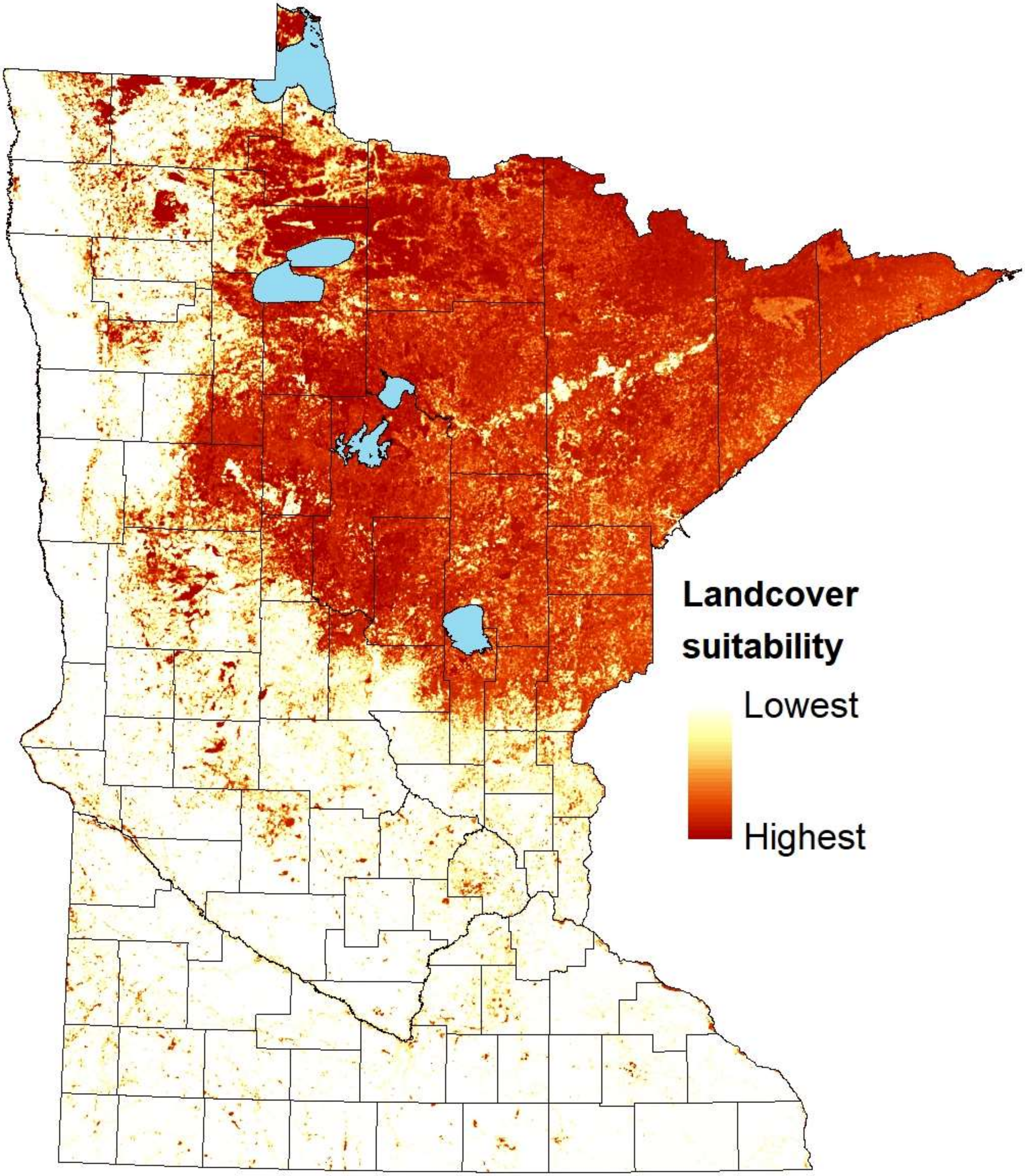

Rock Pigeon *Columba livia*

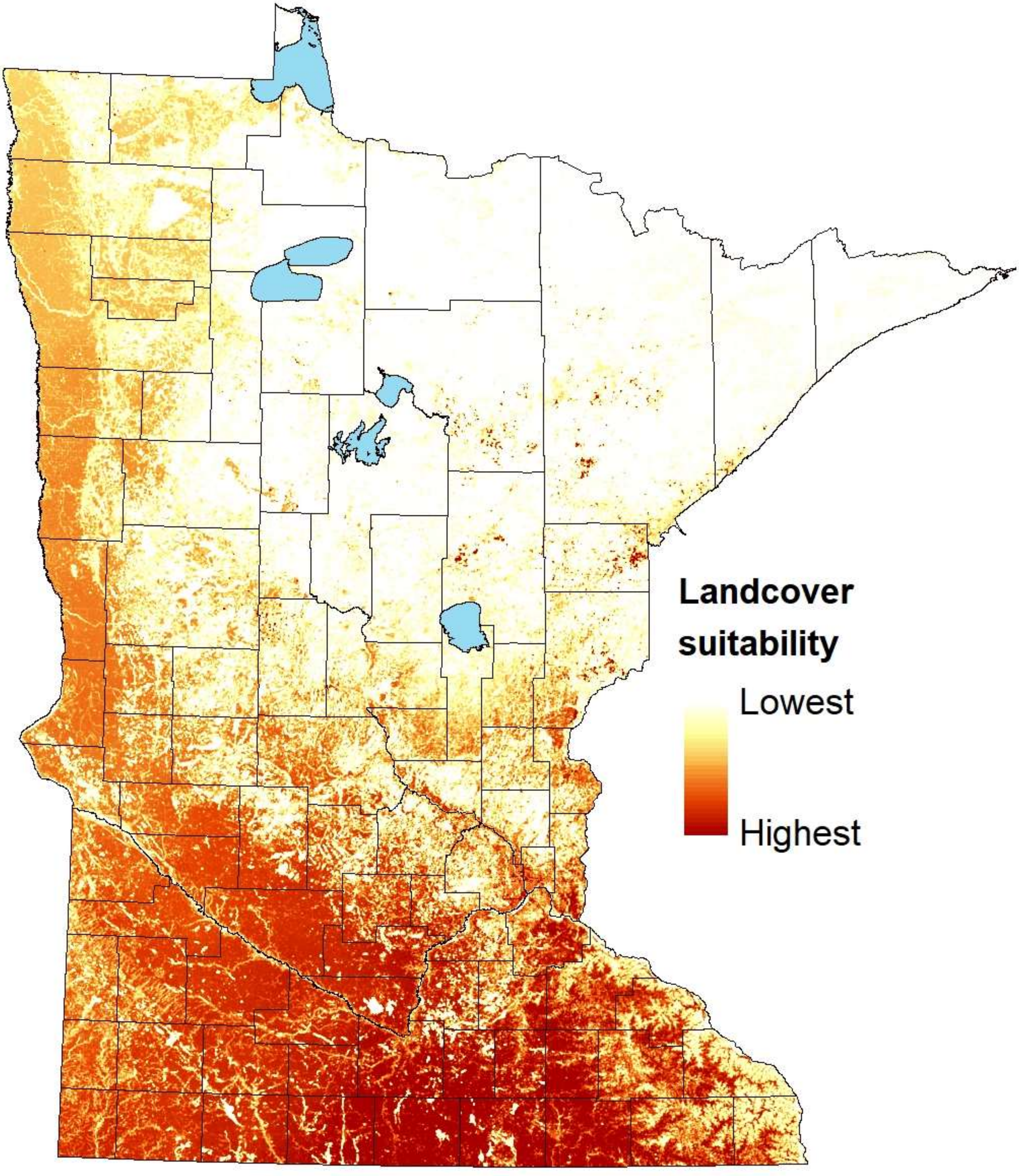

Ruffed Grouse *Bonasa umbellus*

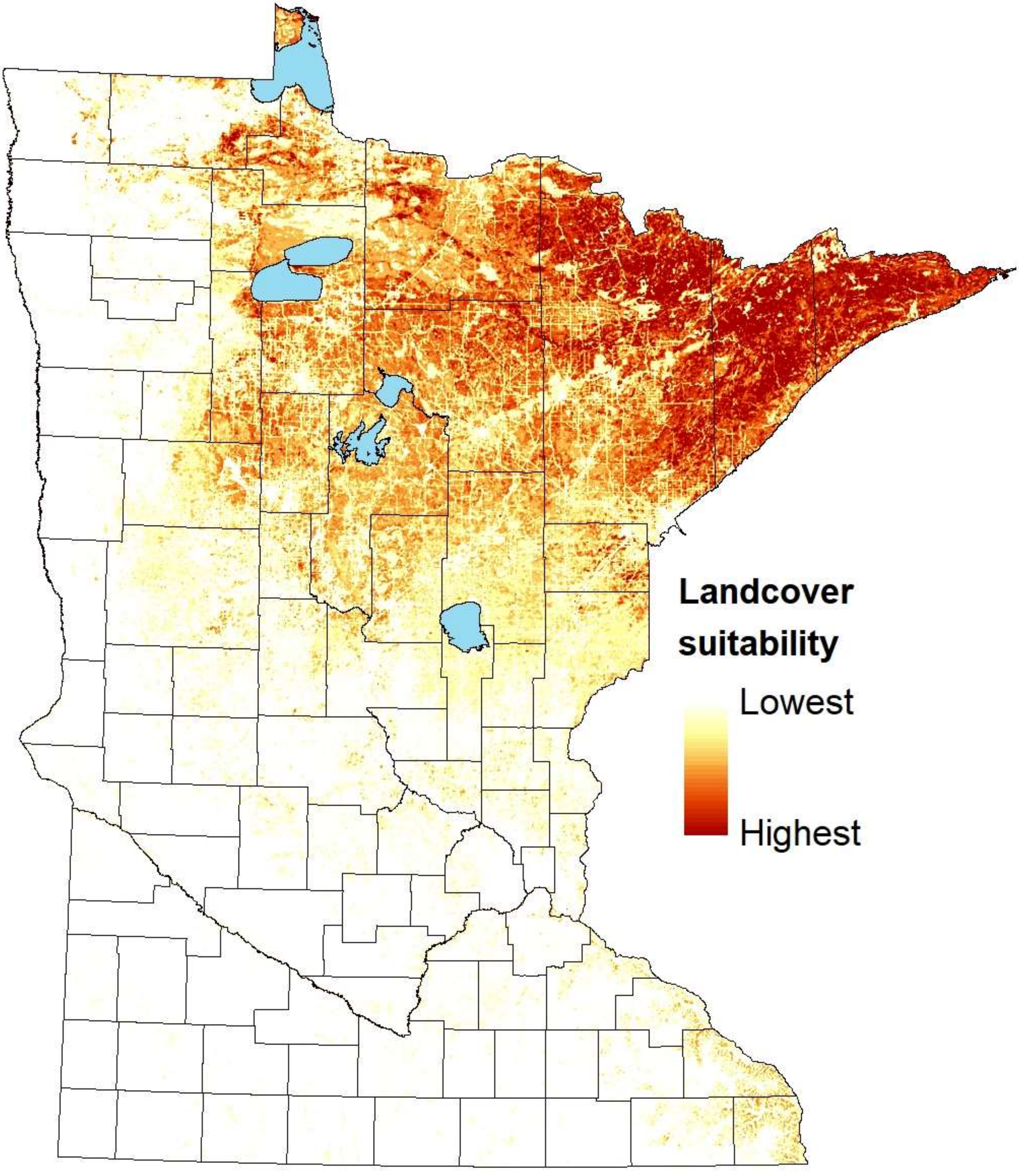

Trumpeter Swan *Cygnus buccinator*

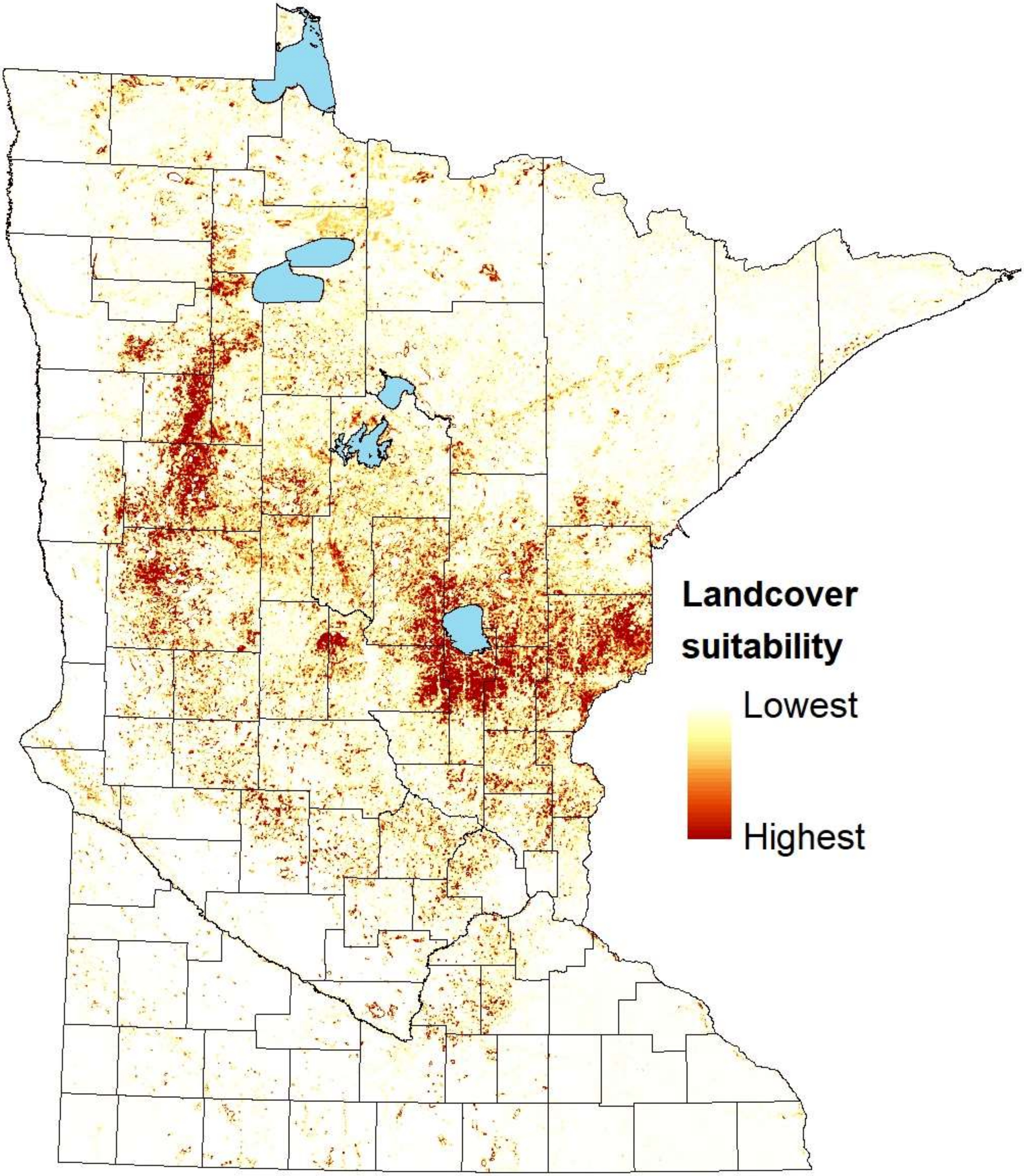

Tufted Titmouse *Baeolophus bicolor*

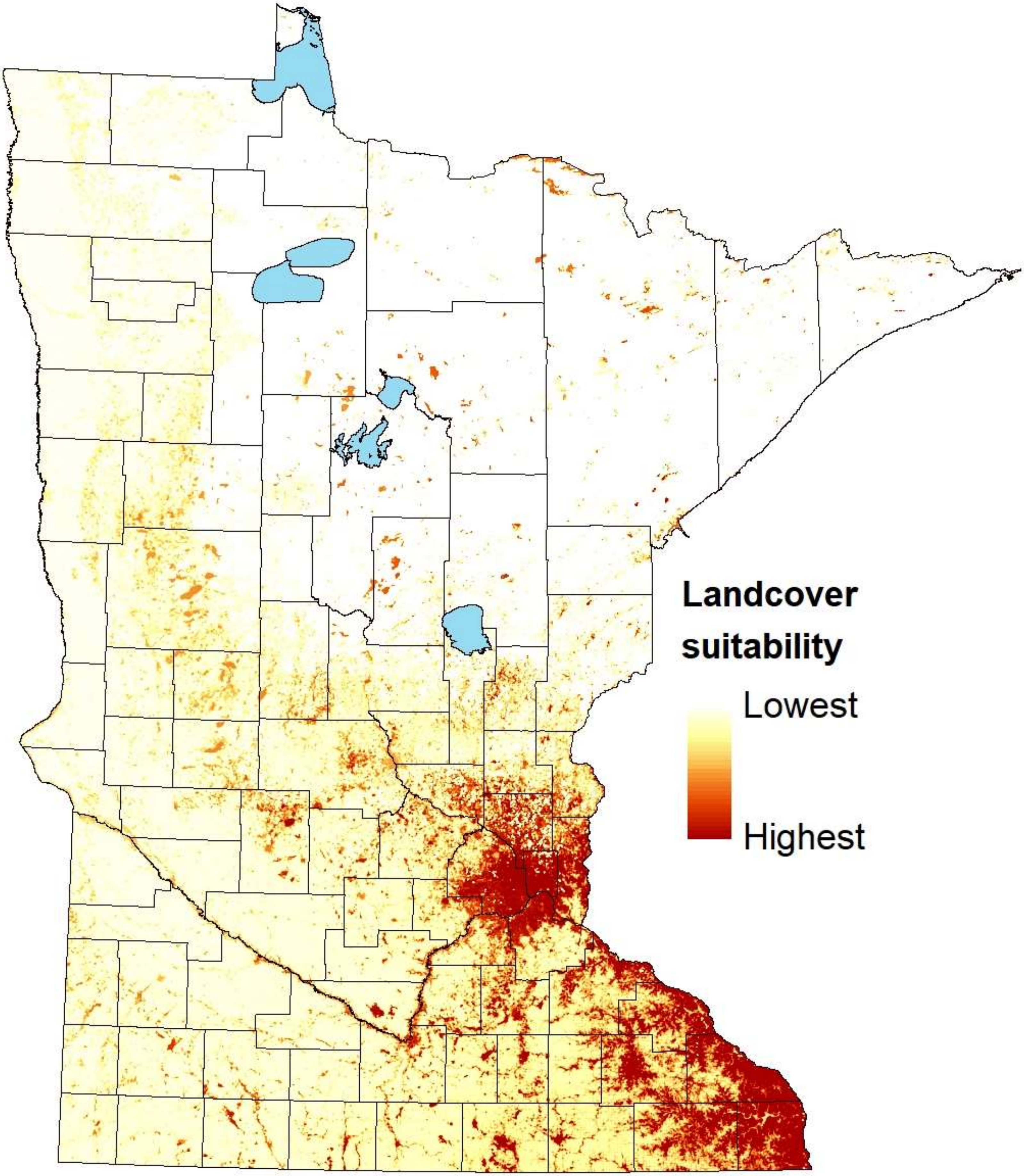

Western Kingbird *Tyrannus verticalis*

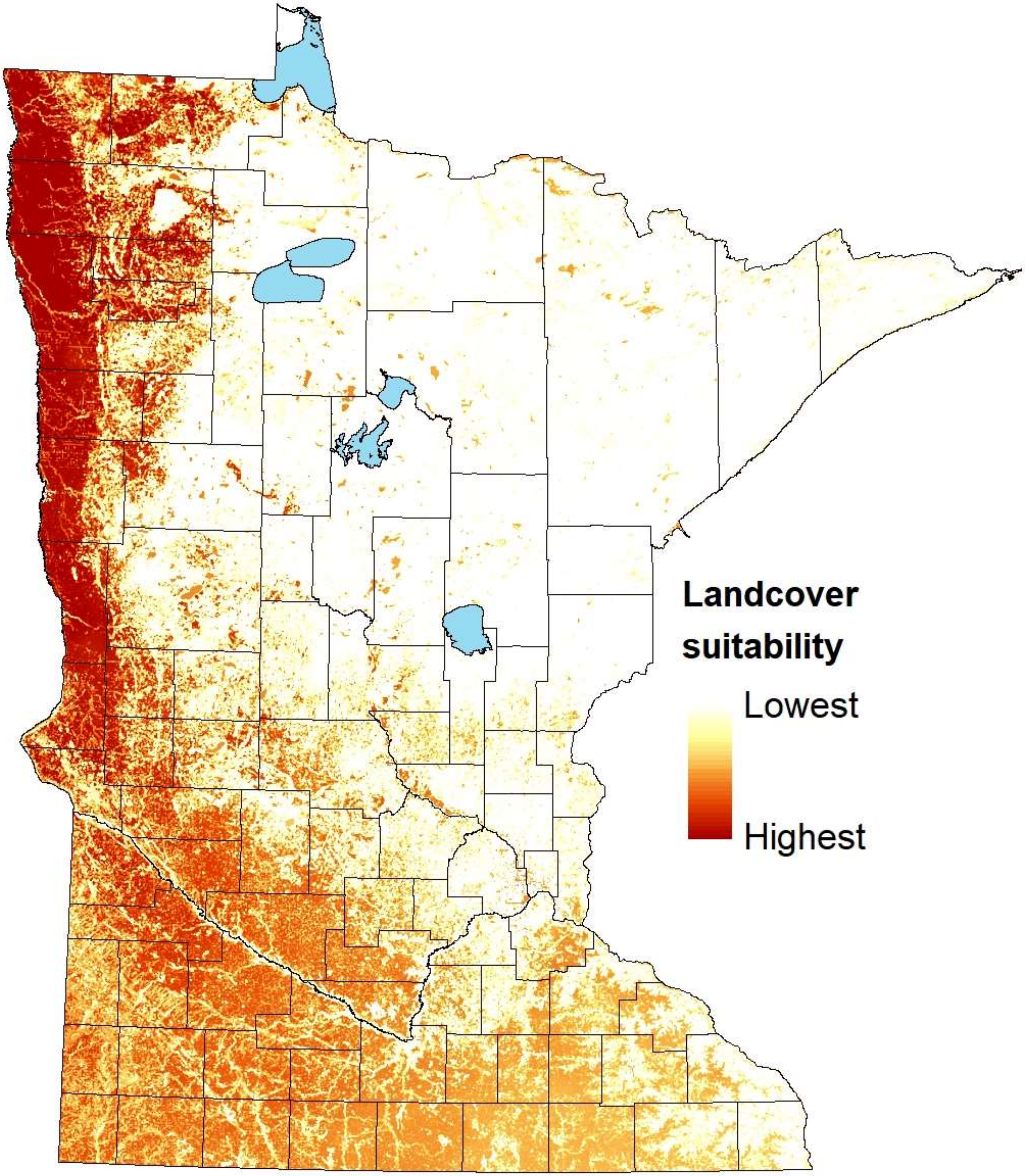

Wild Turkey *Meleagris gallopavo*

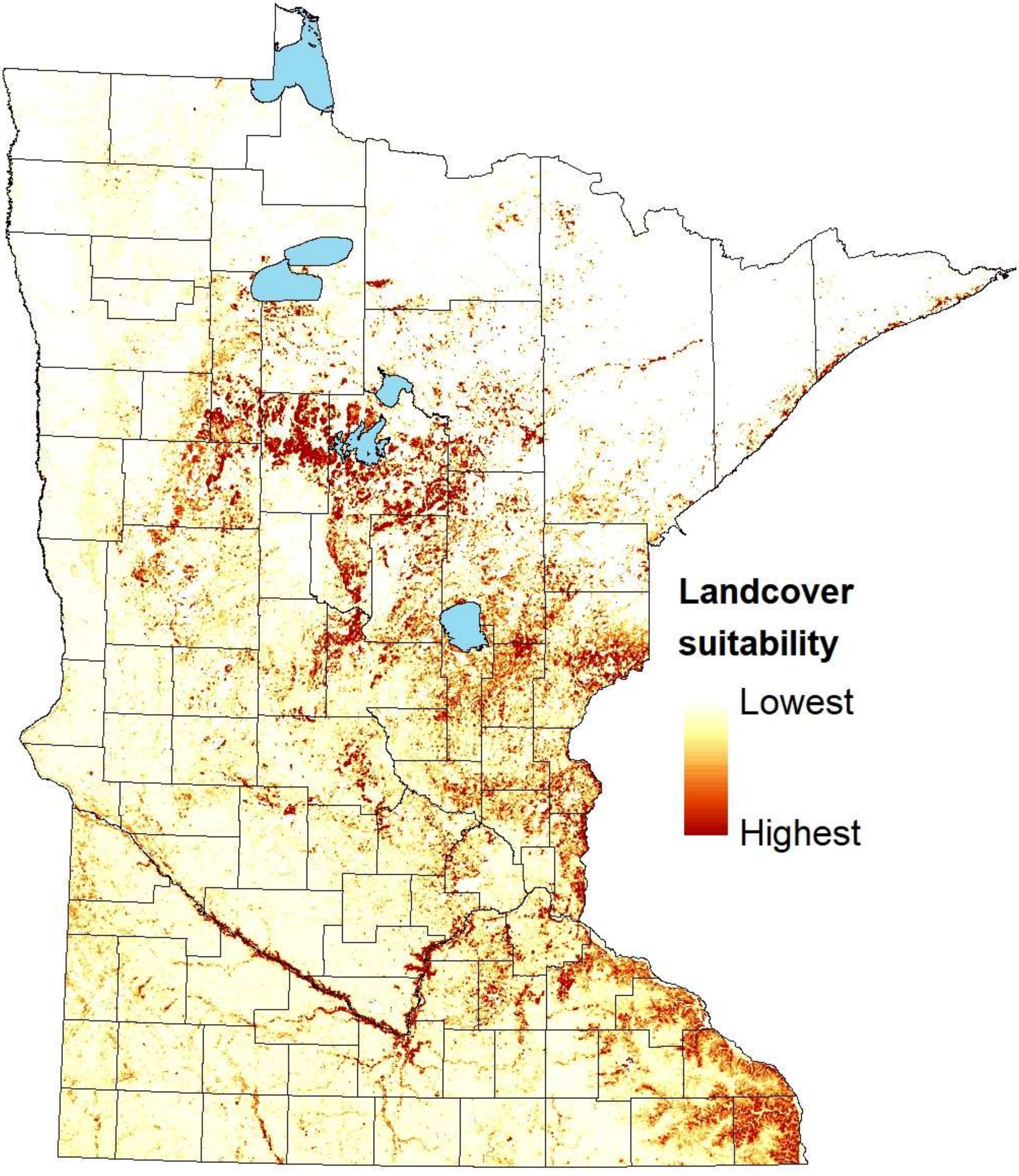

Wilson's Snipe *Gallinago delicata*

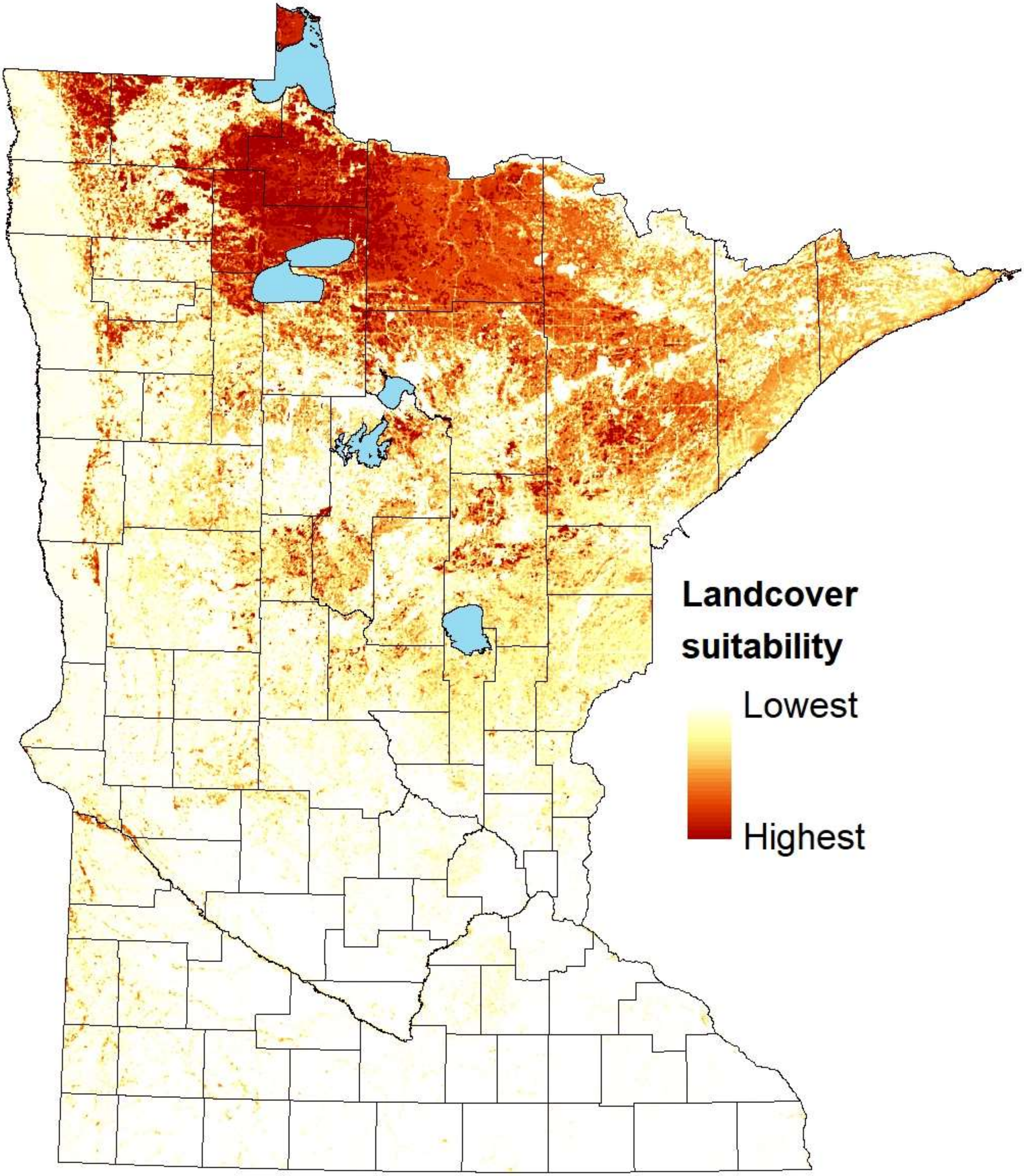

Wilson's Warbler *Cardellina pusilla*

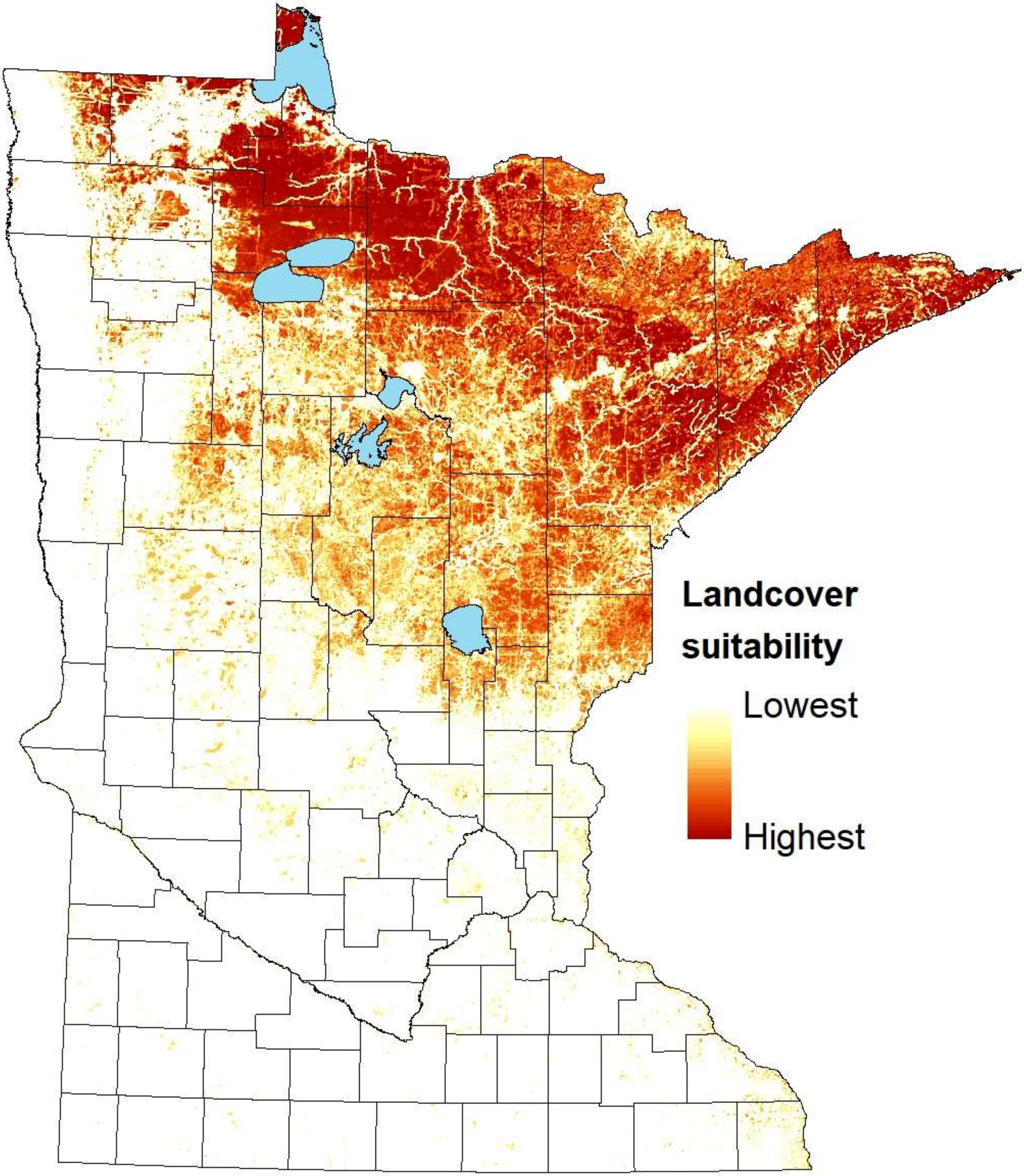

Supplement: Supplementary file 2 — Appendix S5: Species distribution maps for 136 species modeled as part of the Minnesota Breeding Bird Atlas. Species are grouped by model type. [file ECE3-16-e73808-s002.pdf]
